# Efficient Synthesis of Amides through Thioacids Reactions with In Situ Generated Formamidine

**DOI:** 10.1021/acs.joc.5c01644

**Published:** 2025-10-29

**Authors:** Chin-Ling Kuo, Yan-Jie Chen, Hsiang-Jou Chen, Ching-Ching Yu, Chien-Fu Liang

**Affiliations:** † Department of Chemistry, 34916 National Chung Hsing University, 145 Xingda Rd., South Dist., Taichung City 402202, Taiwan; ‡ Department of Chemistry, 34881 National Tsing Huw University,101 Section 2, Kuang Fu Road, Hsinchu 30013, Taiwan; § Institute of Biological Chemistry, Academia Sinica, 128, Academia Road Sec. 2, Nankang, Taipei 11529, Taiwan

## Abstract

A formamidine intermediate generated in situ was used as the nitrogen source in a reaction with thioacids to obtain amides. The synthetic protocol described in this paper can be used in the generation of various primary, secondary, and tertiary amides with organic thioacids used as substrates under the optimized conditions. Notably, this protocol uses readily available materials, is nearly solvent-free, supports gram-scale synthesis, and yields structurally diverse amide products with favorable efficiency.

## Graphical abstract

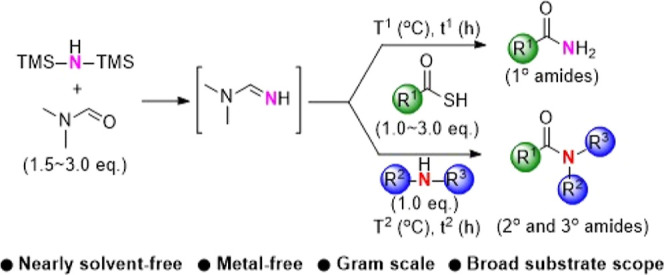

## Introduction

Amides synthesis is a fundamental organic reaction.^1^ Amides, that is, compounds containing a carbon–nitrogen (C–N) bond, are widely used as synthetic intermediates,^2^ natural products,^3^ polymer materials,^4^ and pharmaceuticals.^5^ Approximately 50% of drugs reported in the literature involve an amide bond formation (Figure 1a).^6^ Conventional methods for amide synthesis typically involve the coupling of activated carboxylic acid derivatives, such as acyl chlorides/anhydrides, with amines, or the condensation of carboxylic acid and amines by using stoichiometric amounts of coupling reagents.^7^ However, these methods using acyl halide/anhydride or coupling reagents conditions would lead to the generation of excessive undesired chemical wastes and are susceptible to moisture sensitivity.^7a^ In contrast to the carboxylic acids, thioacids exhibit unique reactivity and selectivity than their carboxylic acid counterparts do.^8^ The distinctive reactivities of thioacids have led to them being extensively used as mild acylating reagents in peptide and peptidomimetic synthesis as well as in the assembly of various bioconjugates.^8^ Thioacids have been employed to construct amide bonds through reactions with azides,^9^ isocyanates/isothiocyanates,^10^ isonitriles,^11^ nitroso compounds,^12^ thiocarbamates,^13^ aziridines,^14^ and amines^15^ (Figure 1b). Because of their effective reactivities for amide bond formation, the development of versatile synthetic methods for exploring the potential of thioacids is still highly desirable.

**Figure 1:**
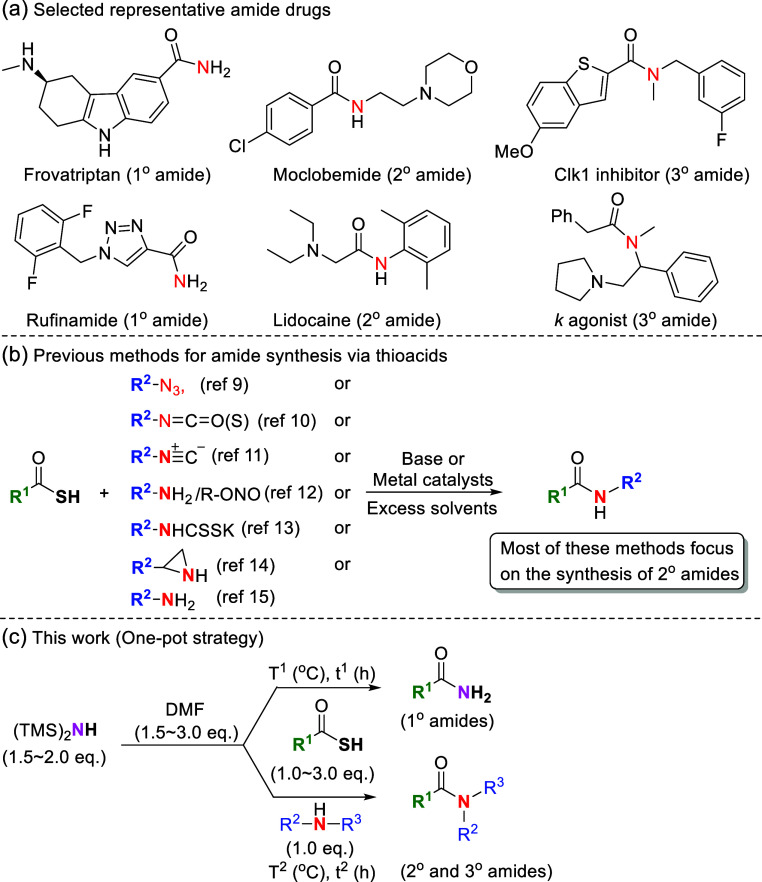
Selected representative amide drugs (a) and methods for the amide synthesis through thioacids (b,c).

Although the efficacy of the synthesis of amides from thioacids through reactions with diverse functional groups has been validated,^9–15^ most of the methods employed to do so require the use of environmentally unfriendly agents, such as transition metals, oxidant additives, corrosive reagents, and excess toxic solvents. Additionally, some methods involve tedious preparation processes for starting materials or have limited substrate scopes for primary and tertiary amides. These limitations highlight the need for novel sustainable synthetic methods that involve the use of thioacids under favorable conditions and that are scalable for industrial applications. We have previously used *N*,*N*-dimethylformamide (DMF) as a C1 source to synthesize *N*-sulfonylformamidines^16^ and 4-acyl-1,2,3-triazoles^17^ through an *N*,*N*-dimethyl formimidamide intermediate generated in situ. The findings indicate that although the synthesis of amides from thioacid is feasible, direct coupling of *N*,*N*-dimethyl formimidamide with thioacids remains a challenge. Herein, we present a green and convenient method involving the use of thioacids as acyl donors and introduce a novel chemical template as the amine source. This approach involves using an N,N-disubstituted formamidine intermediate generated in situ to synthesize diverse primary, secondary, and tertiary amides under metal-free, oxidant-free, and nearly solvent-free conditions (Figure 1c).

## Results and Discussion

To develop a straightforward process for the amidation of thioacids using *N*-formamidine generated in situ, a model study for primary amide formation was optimized. Thiobenzoic acid (**1a**) was reacted with stoichiometric amounts of hexamethyldisilazane (HMDS) and DMF under heating conditions (Table 1). Initially, the use of 2 equiv each of HMDS and DMF resulted in a 43% product yield (entry 1). However, the reaction resulted in yields of only 4.7% in the absence of DMF (2.0 equiv) conditions. Subsequently, 1.5 and 3 equiv of HMDS and DMF agents were used to determine the optimal quantities of these reactants (Table 1, entries 2 and 3, respectively). Notably, under entry 3 conditions, the reaction resulted in a yield of 74%. To determine the minimum effective quantities of HMDS and DMF, samples were prepared using 2, 1.5, and 1 equiv amounts, respectively, and the reactions were conducted again (Table 1, entries 5–8). The results revealed that the conditions in entry 6 represented the minimum HMDS quantity required to achieve optimal activity. Following the subsequent optimization of the reaction temperature, the results revealed that both higher and lower temperatures led to reduced yields (Table 1, entries 9–10). Reaction time was also evaluated, with durations ranging from 1 to 3 h (Table 1, entries 11 and 12). When the reaction time was less than 5 h, the reaction yields were not significantly improved.

**Table 1:**
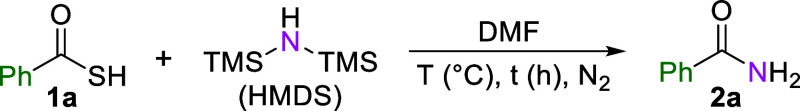
Optimization of the Reaction Conditions^a^

entry	HMDS (equiv)	DMF (equiv)	*T* (°C)	*T* (h)	yield (%)^b^
1	2	2	100	5	43 (4.7)^c^
2	1.5	1.5	100	5	60
3	3	3	100	5	74
4	3	3	100	7	66
5	3	2	100	5	54
6	2	3	100	5	80
7	1.5	3	100	5	70
8	1	3	100	5	35
9	2	3	100	5	71
10	2	3	100	5	75
11	2	3	100	3	78
12	2	3	100	1	73

a Reaction conditions: substrate **1a** (0.65 mmol, 1.0 equiv), HMDS, and DMF were stirred at heating conditions (oil bath) under a nitrogen atmosphere.

b Isolated yield.

c In the absence of DMF (2.0 equiv).

To broaden the scope of the primary amide formation, the optimal conditions for direct amidation were used to synthesize a series of functionalized primary amides through the treatment of HMDS and DMF agents with various structurally and electronically tuned organic thioacid molecules (**1a**–**1z**; Scheme 1).

**Scheme 1:**
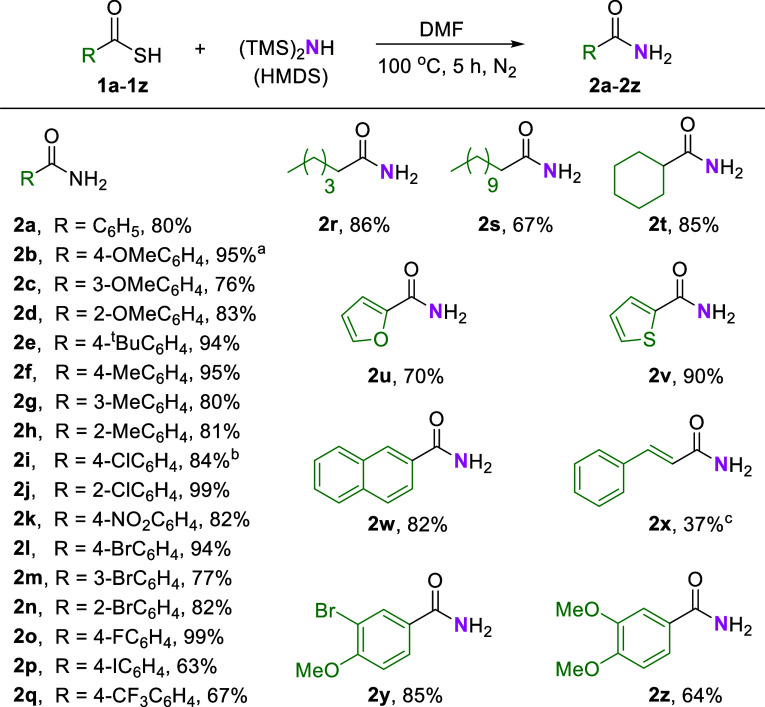
Primary Amide Formation by Using Diverse Thioacids^d^

a Stirred at 80 °C (oil bath) for 5 h.
b Stirred at 100 °C (oil bath) for 3 h.
c The reaction produced **2x** in 37%, including some unrecognized spots.
d Reaction conditions: Thioacid **1** (0.246–0.697 mmol, 1.0 equiv), HMDS (2.0 equiv), and DMF (3.0 equiv) were stirred at 100 °C (oil bath) for 5 h under a nitrogen atmosphere.

Thioacids containing electron-donating groups (methoxy, methyl, and *tert*-butyl, **1b**–**1h**/**1y**–**1z**) led to the formation of the desired products (**2b**–**2h**/**2y**–**2z**) in high yields (64%–95%). Additionally, reactions with thioacids bearing electron-withdrawing groups (e.g., F, Cl, Br, I, NO_2_, or CF_3_, **1i**–**1q**) achieved high yields (63%–99%). Notably, thioacids with ortho-substituted groups (**1d**, **1h**, **1j**, and **1n**) also produced the desired primary amides, namely, **2d**, **2h**, **2j**, and **2n**, at high yields of 83%, 81%, 99%, and 82%, respectively. Heteroatom- and naphthyl-containing thioacids (**1u**–**1w**) exhibited favorable tolerance under the reaction conditions, leading to high yields (70% to 90% yields) of the desired products. However, under optimized conditions, styryl thioacid (**1x**) produced **2x** in a yield of only 37%, along with some unrecognized byproducts. This reduced yield may be attributed to side reactions involving the α,β-unsaturated double bond, which could have reacted with HMDS or thioacid agents through conjugate addition.^18^ Additionally, thioacids with medium- and long-length alkyl chains as well as cyclohexyl thioacid systems produced favorable results, with yields of the corresponding products (**2r**–**2t**) ranging from 67% to 86%. Compared with previous methods for amidation using thioacids, which often involve cumbersome procedures, metal catalysts, and excessive organic solvents, the proposed method offers a greener, more efficient, and simpler synthetic process for primary amides.

Secondary amides have been studied across various research fields because of their diverse applications.^2–6^ In this study, to broaden the applicability of the formamidine intermediate in diverse amidation reactions, we evaluated its role in the synthesis of secondary amides. Building on our previous success in employing *N*,*N*-dimethylformimidamide intermediate as an amidinyl transfer reagent for *N*-arylformamidine synthesis,^16^ we investigated the reaction of *N*-arylformamidine with thioacids to produce secondary amides. First, amidation was performed using *N*-arylformamidine and a thiobenzoic acid (**1a**).

A systematic evaluation of the optimal reaction conditions (Table S1, Supporting Information) revealed that the reaction can proceed efficiently with amine used as the starting material through a sequential one-pot two-step process. In the first step, aniline (**3a**, 1.0 equiv) was reacted with stoichiometric amounts of HMDS (1.5 equiv) and DMF (1.5 equiv) under PPTS catalysis, solvent-free, and heating conditions to generate the *N*-substituted formamidine intermediate. Subsequently, amidation was performed by reacting this intermediate with thiobenzoic acid (**1a**, 3.0 equiv) in the presence of DBU (1.5 equiv) to yield the secondary amide (**4aa**) in 91% yield. To extend the scope of this study, the optimal conditions were applied for the synthesis of a series of functionalized secondary amides by reacting various amines (**3**) with thiobenzoic acid (**1a**) (Scheme 2). Under these conditions, various functionalized anilines (**3a**–**3u**, **3w**–**3x**) were converted into the corresponding secondary amide adducts (**4aa**–**4au**, **4aw**–**4ax**), with reaction yields of 54%–97%. The ease of this conversion and favorable yields of the aniline derivatives indicate that structural and electronic substituents on the phenyl group of the aniline derivatives did not significantly influence the reaction yields. In addition, amidation involving the more sterically hindered *o*-substituted aniline (**3j**) produced the amide product (**4aj**) with a reduced yield of 54%. Aniline containing a nitro group at the para position failed to yield the desired product (**4av**). Additionally, reactions involving cycloalkyl (**3y**), benzyl (**3b′**), short-length alkyl chain (**3c′**), and heteroaromatic (**3d′** and **3e′**) primary amines were investigated, and the results revealed that amidation of these amines proceeded efficiently, producing the corresponding amide adducts in acceptable yields (48–78%). However, compared with the amidation of short-length alkyl chain amines (**3c′**), the transformation of long- and medium-length alkyl chain amines (**3z** and **3a′**) was less efficient under the optimal conditions, resulting in relatively lower yields of products such as **4az** (22%) and **4aa′** (16%). In consideration of these promising results (Scheme 2), aniline (**3a**) was treated with various organic thioacids (**1b**–**1r**, **1t**–**1w**, and **1a’**–**1b′**) to further explore amide synthesis (Scheme 2). Aryl thioacids bearing electron-donating and electron-withdrawing groups on the phenyl ring successfully reacted with *N*-phenylformamidine generated in situ, yielding the corresponding amide products (**4ba**–**4ca**, **4ea**–**4ja**, **4la**–**4ra**, **4ta**–**4wa**, and **4a′a**–**4b′a**) in moderate to high yields. In addition, amidation involving the more sterically hindered *o*-substituted thioacid (**1d**) produced the amide product (**4da**) in a yield of only 15%. Aryl thioacid with a nitro group at the para position did not produce the desired product (**4ka**). Additionally, reactions involving alkyl (**1r** and **1b′**), cycloalkyl (**1t**), heteroaromatic (**1u** and **1v**), 2-naphthyl (**1w**), and phenylacetyl (**1a′**) thioacids demonstrated favorable tolerance to the reaction conditions. These substrates yielded the desired secondary amide products (**4ra**, **4ta**–**4wa**, and **4a′a**–**4b′a**) in yields between 53% and 87%.

**Scheme 2:**
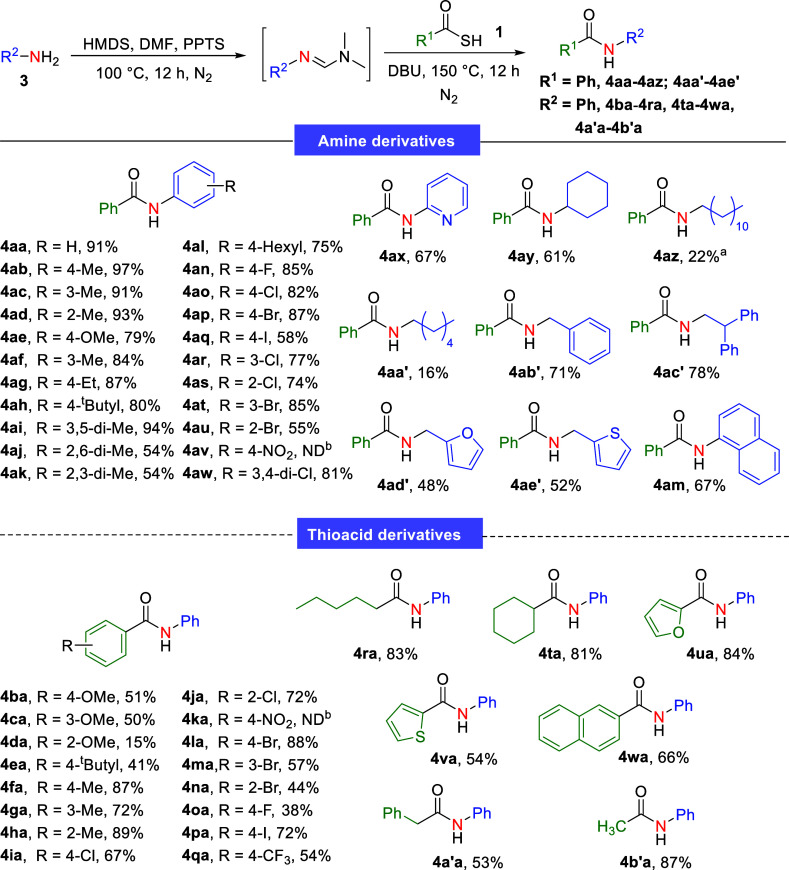
Scope of Secondary Amide Formation Using Diverse Amines and Thioacids^c^

a Amine (**3z**) was used 5.39 mmol in the reaction.
b ND = not detected.
c Reaction conditions: Amine **3** (1.07–5.39 mmol, 1.0 equiv), HMDS (1.5 equiv), DMF (1.5 equiv), and pyridinium *p*-toluenesulfonate (PPTS, 2 mol %) were stirred at 100 °C (oil bath) for 12 h under a nitrogen atmosphere. Subsequently, thioacid **1** (3.0 equiv) and DBU (1.5 equiv) were added to the reaction mixture and stirred at 150 °C (oil bath) for 12 h under a nitrogen atmosphere.

To further validate the practical utility of the formamidine intermediate in amidation reactions, we explored its application in the synthesis of tertiary amides. By using a secondary amine to generate the *N*,*N*-disubstituted formamidine intermediate in situ, amidation was performed under the aforementioned optimal conditions, and it resulted in the formation of tertiary amide derivatives. The results of these reactions are summarized in Scheme 3. In most cases, the transformation resulted in acceptable yields, with product yields of 31–91% (**4af’**–**4am′**, **4b’f’**). Specifically, tertiary amides such as *N*-benzyl-*N*-methyl (**4af′**, **4b’f’**), *N*-methyl-*N*-phenylethyl (**4ag′**), *N*-cyclohexyl-*N*-methyl (**4ah′**), morpholyl (**4ai′**), piperidinyl (**4aj′**), azepanyl (**4ak′**), *N*,*N*-dibutyl (**4al′**), and *N*,*N*-dihexyl (**4am′**) were synthesized in moderate to good yields under the optimized conditions. However, the amidation of isoindoline did not produce the desired product **4an’**.

**Scheme 3:**
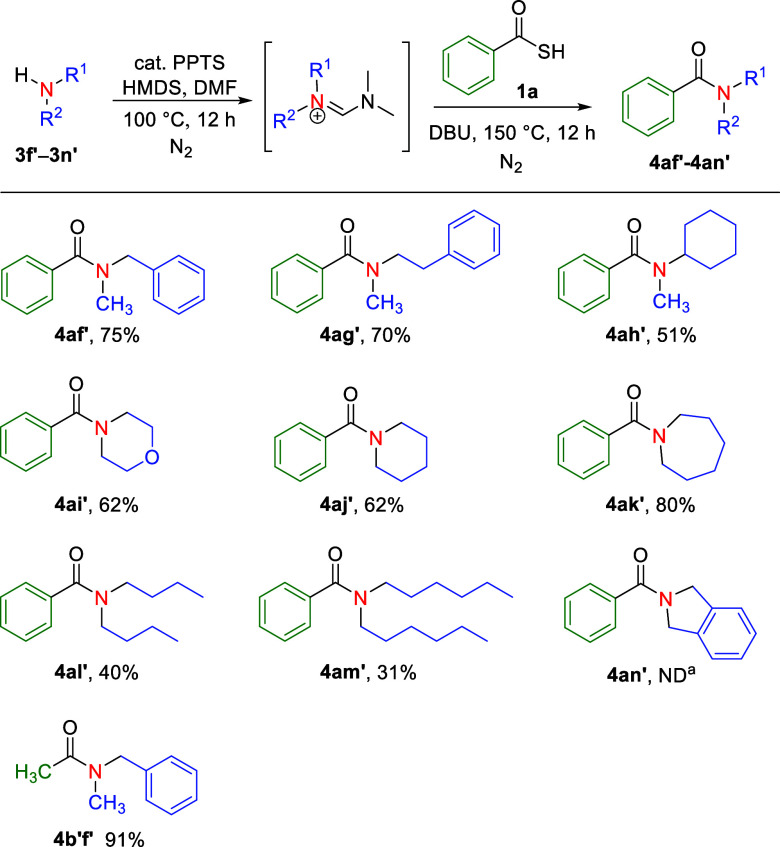
Scope of Tertiary Amide Formation Using Diverse Amines^b^

a ND = Not detected.
b Reaction conditions: Amine **3** (1.62–3.52 mmol, 1.0 equiv), HMDS (1.5 equiv), DMF (1.5 equiv), and PPTS (2 mol %) were stirred at 100 °C (oil bath) for 12 h under a nitrogen atmosphere. Subsequently, thiobenzoic acid **1a** (3.0 equiv) and DBU (1.5 equiv) were added to the reaction mixture and stirred at 150 °C (oil bath) for 12 h under a nitrogen atmosphere.

Therefore, the proposed method for primary, secondary, and tertiary amidation provides a green synthetic process for amide formation. For primary amidation, the reaction conditions are milder than those of the secondary and tertiary amidations, and the overall yields are good to excellent. In the subsequent phase of the study, thiobenzoic acids (**1a** and **1f**) were used to synthesize primary, secondary, and tertiary amide to demonstrate the scalability of the proposed synthetic method. To further validate its applicability, gram-scale syntheses were performed, and yields of 85%, 89%, and 72% were achieved for products **2f**, **4aa**, and **4af′**, respectively (Scheme 4).

**Scheme 4:**
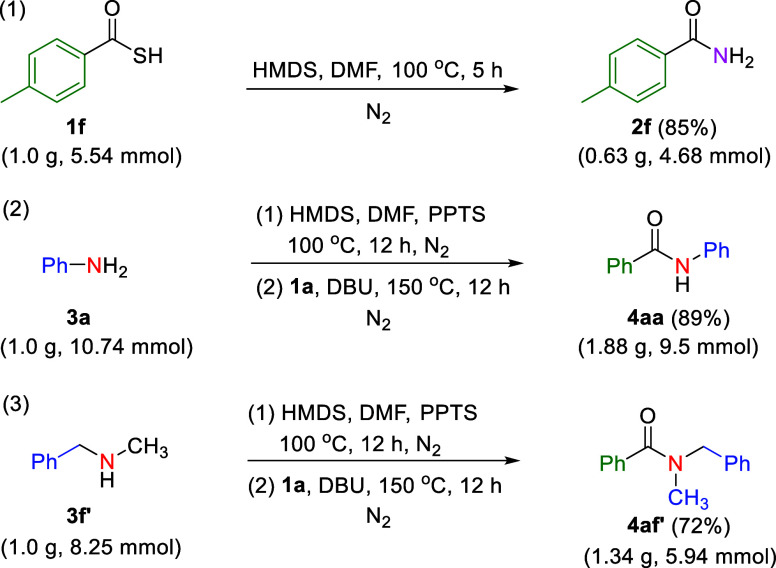
Gram-Scale Reactions

To elucidate the reaction mechanism, a series of control experiments were conducted (Scheme 5). First, thiobenzoic acid (**1a**) was converted into primary amide (**2a**) under HMDS/DMF-mediated conditions, with a yield of 80% (Scheme 5a). Notably, compound (**5**) was also obtained and characterized through column chromatography, with a yield of 85% (see the Supporting Information), indicating that HMDS can serve as a nitrogen source for primary amide generation. Next, *N*-phenylformamidine (**6a**) was used as the starting material and reacted with thiobenzoic acid (**1a**) in stoichiometric amounts with DBU and toluene. The reaction successfully resulted in a secondary amide (**4aa**), at a yield of 90% (Scheme 5b). However, the reaction only observed traces of byproduct **5** under the reaction conditions (Table 1, Supporting Information). Additionally, the radical trapping reagent (TEMPO) was introduced to the reaction system, which led to the formation of the product **2a** at a 71% yield (Scheme 5c). On the basis of the results of these control experiments and literature review,^9–15^ we identified plausible mechanisms for the synthesis of primary, secondary, and tertiary amides (Scheme 5d). One report suggested that DMF reacts with HMDS to generate *N*,*N*-dimethylformimidamide intermediate **A**,^16^ which then acts as a nitrogen source in amidation reactions. In the synthesis of primary amides, *N*,*N*-dimethylformimidamide intermediate **A** reacts with thiobenzoic acid under heating conditions, resulting in the formation of intermediate **B**. Intermediate **B** then undergoes rapid and spontaneous rearrangement through an intramolecular *S*,*N*-acyl transfer,^19^ yielding primary amide **2** and compound **5** (Scheme 5d). The mechanism for secondary amide synthesis may be similar to that of primary amides. In this case, *N*-substituted formamidine (**6**) is generated in situ and subsequently reacts with thioacid (**1**) in the presence of a base. Because of the pronounced S-nucleophilicity of thiolate (**8**), the reaction of **8** with *N*-substituted formamidine **6** was initially believed to involve *S*-nucleophilic addition to generate intermediate **C**. Subsequently, the *N*-substituted amino group of intermediate **C** undergoes rapid and spontaneous rearrangement through an intramolecular *S*,*N*-acyl transfer, leading to the formation of secondary amide **4** and compound **5** (Scheme 5d). Furthermore, the synthesis of tertiary amides from secondary amines may involve a mechanism similar to that of the formation of secondary amides (Scheme 5d).

**Scheme 5:**
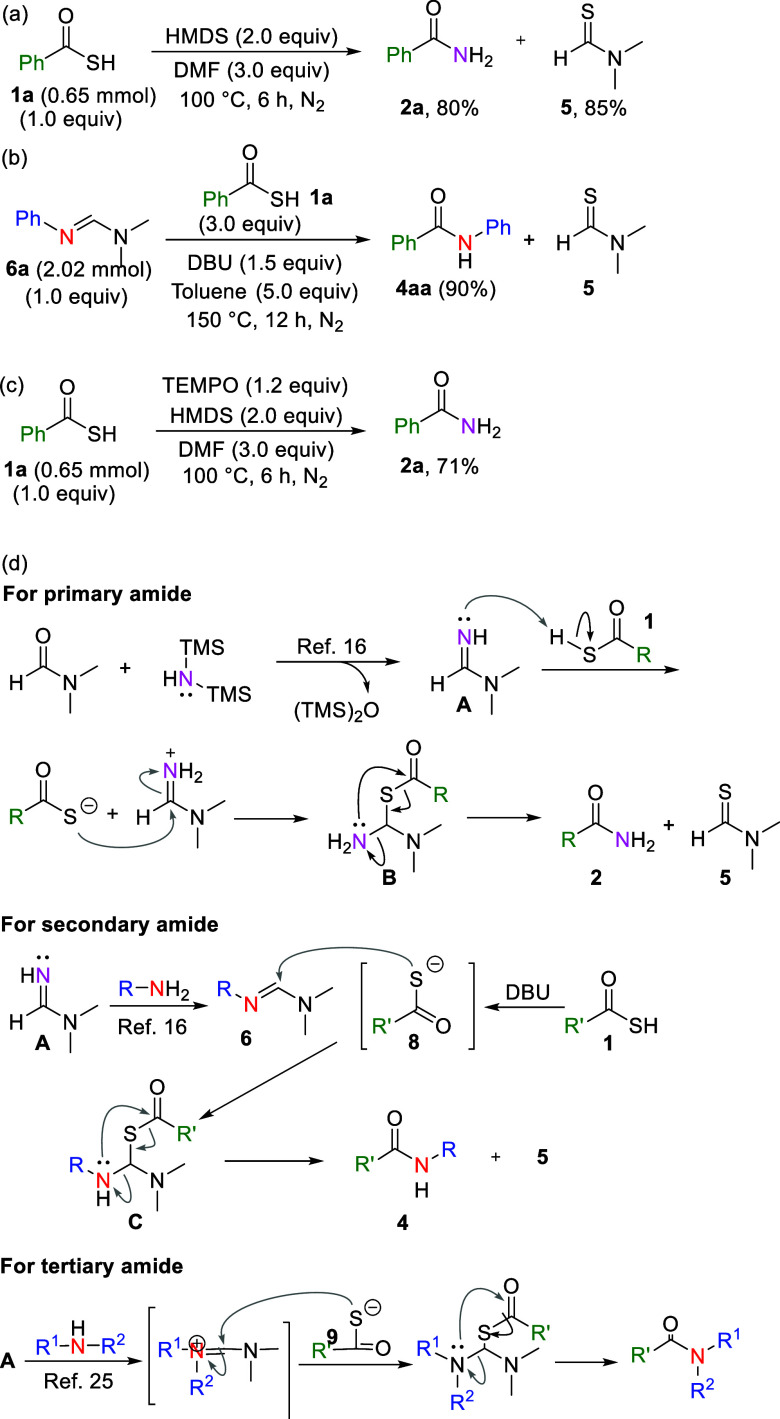
Control Experiments and Proposed Mechanisms

## Conclusions

In summary, we successfully synthesized primary, secondary, and tertiary amides by using readily available thioacids and formamidine intermediates generated in situ under metal-free, oxidant-free, and nearly solvent-free conditions. HMDS was reacted with DMF to generate the *N*,*N*-dimethylformimidamide intermediate in situ, which then served as a nitrogen source. Thereafter, the nucleophilic addition of thioacid to the *N*,*N*-dimethylformimidamide intermediate led to the generation of various primary amides. Moreover, the *N*,*N*-dimethylformimidamide intermediate was reacted with primary or secondary amines to generate N-substituted or N,N-disubstituted formamidines in situ, which subsequently underwent amidation with thioacids to, respectively, produce secondary or tertiary amides. This synthetic protocol is environmentally friendly (metal-fee, oxidant-free, and nearly solvent-free) and can be applied for the formation of various functionalized primary, secondary, and tertiary amides. Furthermore, the formamidine intermediate generated in situ can be used as a nitrogen source in diverse amidation reactions.

## Supplementary Material



## Data Availability

The data underlying this study are available in the published article and its Supporting Information.

## Notes

Detailed experimental procedures, characterization data of compounds, and NMR (^1^H, ^13^C, and ^19^F) spectra for all compounds (PDF)
