# Supplementary material for: Efficient Synthesis of Amides through Thioacids Reactions with In Situ Generated Formamidine
Source: J Org Chem. 2025 Oct 29;90(44):15578–84. doi: 10.1021/acs.joc.5c01644 (PMC12604045; doi:10.1021/acs.joc.5c01644)
Supplement: Supplementary file 1 [file jo5c01644_si_001.pdf]

Supporting Information

Efficient Synthesis of Amides Through Thioacids Reactions With In Situ Generated Formamidine

Chin-Ling Kuo,[#a] Yan-Jie Chen,[#ab] Hsiang-Jou, Chen,[a] Ching-Ching Yu,[*bc] Chien-Fu Liang[*a]

[a] Department of Chemistry, National Chung Hsing University, Taichung 402202, Taiwan.

[b] Department of Chemistry, National Tsing Hua University, Hsinchu 300, Taiwan.

[c] Institute of Biological Chemistry, Academia Sinica, 128, Academia Road Sec. 2, Nankang, Taipei 11529, Taiwan.

E-mail: lcf0201@dragon.nchu.edu.tw

E-mail: ccyu2@mx.nthu.edu.tw

Table of contents

1. General Information....................S2
2. Table S1: Optimized Conditions for Secondary Amide Bond Synthesis....................S3
3. General Procedure....................S4
4. Characterization Data....................S5
5. References....................S39
6. Copies of ^{1}H, ^{13}C and ^{19}F NMR Spectra of products....................S42

General Information

All reactions were performed under nitrogen atmosphere. Solvents were purified following standard literature procedures. ^{1}H NMR and ^{13}C NMR spectra were recorded on a Varian 400 MHz and Jeol 400 MHz NMR spectrometer with $CDCl_3$、CD_3OD、*d_6*-DMSO as the solvents. Chemical shifts were reported in parts per million (ppm) relative to residual solvent peak ($CDCl_3$ δ H = 7.26 ppm, δ C = 77.16 ppm; CD_3OD δ H = 3.31 ppm, δ C = 47.56 ppm; $(CD_3)_2SO$ δ H = 2.50 ppm, δ C = 39.52 ppm). TLC was performed on pre-coated glass plates of Silica Gel 60 F254 (0.25 mm, E. Merck); detection was performed by spraying with using a UV light and a solution of basic aqueous potassium permanganate ($KMnO_4$) and Phosphomolybdic acid (PMA) with heating. Flash column chromatography was carried out on Silica Gel 60 (230–400 mesh, E. Merck). High resolution mass spectrometry data were recorded on ESI spectrometer and EI spectrometer. Melting points were measured by Electrothermo (UK) "Mel-Temp 1101D" type melting point apparatus and are uncorrected. The following abbreviations were used to indicate multiplicities: s = singlet, d = doublet, t = triplet, q = quartet, quin. = quintet, dd = doublet of doublets, dt = doublet of triplets, td = triplet of doublets, ddd = doublet of doublet of doublets, br = broad, m = multiplet. Yields of products refer to chromatographically purified products unless otherwise stated. Thiobenzoic acid (**1a**) and thioacetic acid (**1b'**), and hexamethyldisilazane were purchase from Alfa Aesar or Acros Organics, and these reagents were used as received unless otherwise stated.

Table S1. Optimized Conditions for Secondary Amide Bond Synthesis

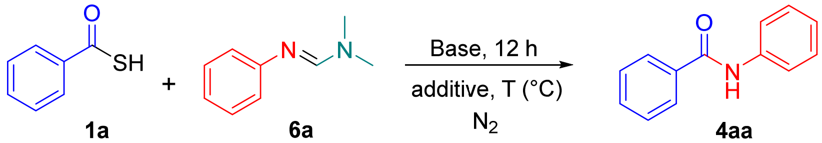

Entry[a]	**1a** (equiv)	Base	Solvent (equiv)	T (°C)	Yield (%)[b]
1	1.5	DBU	1,4-dioxane (5)	100	24%
2	1.5	DBU	DMF (5)	100	30%
3	1.5	DBU	Toluene (5)	100	31% (29%)[d]
4	1.5	DBU	Toluene (5)	130	41%
5	1.5	DBU	Toluene (5)	150	60%
6	1.5	DBU	Toluene (0.5 M)	150	17%[h]
7	1.5	–	–	150	32%
8	1.5	DBU	–	150	48%
9	1.5	–	Toluene (5)	150	50%
10	3.0	DBU	Toluene (5)	150	77%[g]
11	5.0	DBU	Toluene (5)	150	74%
12[c]	3.0	DBU	Toluene (5)	150	33%
13[d]	3.0	DBU	Toluene (5)	150	79%
14	3.0	NEt_3	Toluene (5)	150	56%
15	3.0	K_2CO_3	Toluene (0.85 M)	150	59%
16	3.0	Cs_2CO_3	Toluene (0.85 M)	150	35%
17	3.0	DMAP	Toluene (5)	150	56%
18	3.0	2,6-lutidine	Toluene (5)	150	68%
19[e]	3.0	DBU	Toluene (5)	150	90%
**20[f]**	**3.0**	**DBU**	**–**	**150**	**91%**

[a] Reaction conditions: Amidine **6a** (1.0 equiv, 50 mg, 0.34 mmol), Base (1.5 equiv) under nitrogen atmosphere. [b] Isolated yield. [c] Base (0.5 equiv). [d] Base (3.0 equiv). [e] Amidine (300 mg). [f] One-pot two steps: Aniline **3a** (300 mg, 3.22 mmol, 1.0 equiv), HMDS (1.5 equiv) and DMF(1.5 equiv) was reacted in the presence of PPTS (2 mole%) under 100 °C (oil bath) for 12 h. Then, the thiobenzoic **1a** (3.0 equiv) and DBU (1.5 equiv) were added to the reaction mixture and stirred for 12 h at 150 °C (oil bath). [g] The condition resulted in byproduct of *N*,*N*-dimethylbenzamide (**7a**) in 21% yield. [h] The reaction was also obtained *N*-phenylformamide (**9**) and *N*,*N*-dimethylbenzothioamide (**10**) byproducts, and the yield of these two byproducts was less than 10%.

The model of the secondary amides synthesis was optimized using thiobenzoic acid (**1a**) and *N*-phenylformamidine (**6a**) under different base, solvent, and reaction temperature conditions (Table S1). Initially, the reactivity of thiobenzoic acid (**1a**) reacting with *N*-phenylformamidine (**6a**) was investigated in the presence of DBU with solvents (including 1,4-dioxane, DMF, and Toluene) and reaction temperature (including 100 °C, 130 °C, and 150 °C) (entries 1−9). The results revealed that the reaction under stoichiometric amounts of toluene (5.0 equiv) and high temperature (150 °C) shown the temporarily optimal conditions (entry 6). Additionally, to determine the optimal base agent, reaction conditions were set according to the use of diver bases under using 3.0 equiv of thiobenzoic acid (**1a**), including DBU, NEt_3, K_2CO_3, Cs_2CO_3, DMAP, and 2,6-lutidine (entries 10−19). We found that the reaction could proceed favorably using 3.0 equiv of thiobenzoic acid with 1.5 equiv of DBU and 5.0 equiv of toluene at 150 °C to obtain a satisfactory yield of the desired product **4aa** (90%) (entry 19). Notably, we found that the reaction can proceed efficiently with amine used as the starting material

through a sequential one-pot two-step process. In the first step, aniline (**3a**, 1.0 equiv) was reacted with stoichiometric amounts of HMDS (1.5 equiv) and DMF (1.5 equiv) under PPTS catalysis, solvent-free and heating conditions to generate the *N*-substituted formamidine intermediate. Subsequently, amidation was performed by reacting this intermediate with thiobenzoic acid (**1a**, 3.0 equiv) in the presence of DBU (1.5 equiv) to yield the secondary amide (**4aa**) at a 91% yield (entry 20).

Experimental Procedures

General Procedure I: For the synthesis of thioacids[1]

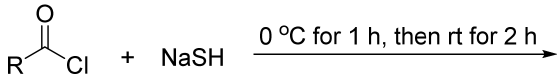

An oven-dried round-bottomed flask (50 mL) equipped with a stir bar was charged with sodium hydrosulfide hydrate (2.0 equiv, 10 mmol) and MeOH (6 mL). Acyl chloride (5 mmol, 1.0 equiv) was added dropwise to the reaction mixture with vigorous stirring at 0 °C, and maintained the temperature at 0 °C for 1 hour. Then, the solution was stirred at room temperature for 2 hours. After the indicated time, the reaction mixture was poured into water, acidized with 1 N aqueous HCl, extracted with EtOAc for three times and the combined organic layer was washed with brine, dried over $MgSO_4$, filtered and concentrated in rotary evaporators to produce the crude thioacids (**1b**–**1z** and **1a'**) as a yellow oil or solid (about 50-98 % purity, checked by ^1H NMR) and can be directly used to the amide formation without purification.

General procedure II: For the synthesis of primary amides (2a–2z)

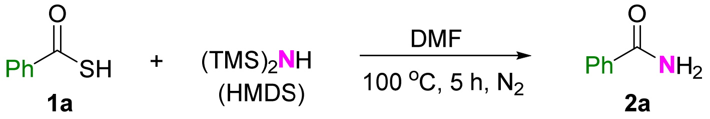

To a solution of thiobenzoic acid **1a** (90.0 mg, 0.65 mmol, 1.0 equiv) was added hexamethyldisilazane (1.30 mmol, 0.273 mL, 2.0 equiv) and *N,N*-dimethylformamide (1.95 mmol, 0.16 mL, 3.0 equiv). Then the resulting reaction mixture was stirred at 100 °C (oil bath) for 5 hours under nitrogen atmosphere. After the indicated time, the condenser tube was roughly washed by MeOH and DCM. After the solution was evaporated under reduced pressure, the crude products were purified by flash column chromatography to afford the desired product **2a** (81.9 mg, 80% yield) as a white solid. The products of **2b**–**2z** were synthesized by following general procedure II.

General procedure III: For the synthesis of secondary amides (4aa–4az, 4aa'–4ae', 4ba–4ra, 4ta–4wa, 4a'a–4b'a) and tertiary amides (4af'–4am', 4b'f')

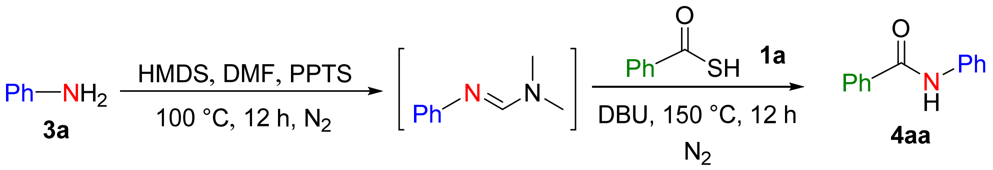

To a solution of the aniline **3a** (300 mg, 3.22 mmol, 1.0 equiv) and pyridinium *p*-toluenesulfonate (16.2 mg, 0.064 mmol, 2 mole %) was added *N*,*N*-dimethylformamide (4.83 mmol, 0.374 mL, 1.5 equiv) and hexamethyldisilazane (4.83 mmol, 1.03 mL, 1.5 equiv). Then the resulting reaction mixture was stirred at 100 °C (oil bath) for 12 hours under nitrogen atmosphere. Then, the thioacid (9.66 mmol, 1.14 mL, 3.0 equiv) and 1,8-diazabicyclo[5.4.0]undec-7-ene (4.83 mmol, 0.722 mL, 1.5 equiv) were added to the mixture and stirred at 150 °C (oil bath) for 12 hours under nitrogen atmosphere. The crude products were purified by flash column chromatography to afford the desired product **4aa** (577.5 mg, 91% yield) as a white solid. The products of secondary (**4ab–4az, 4aa'–4ae', 4ba–4ra, 4ta–4wa**, **4a'a–4b'a**) and tertiary amides (**4af'–4am', 4b'f'**) were synthesized by following general procedure III.

Characterization Data

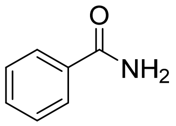

Benzamide (2a)[2]**:** Following the general procedure II, using the thiobenzoic acid **1a** (90.0 mg, 0.65 mmol), hexamethyldisilazane (0.273 mL, 1.30 mmol) and *N,N*-dimethylformamide (0.160 mL, 1.95 mmol). The crude product was then purified by column chromatography (gradient elution: 10% to 50% EtOAc in CH_2Cl_2) to afford compound **2a** (81.9 mg, 80% yield) as a white solid. ^1H NMR (400 MHz, $CDCl_3$) δ 7.82 (d, J = 7.0 Hz, 2H), 7.54 (t, 1H, J =7.4 Hz) 7.46 (t, 2H, J = 7.5 Hz), 6.09 (br, 1H), 5.76 (br, 1H); $^{13}C\{^1H\}$ NMR (100 MHz, $CDCl_3$) δ 169.9, 133.5, 132.1, 128.7, 127.5.

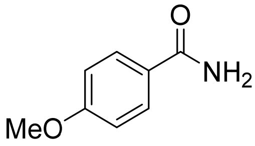

4-Methoxybenzamide (2b)[3,4]**:** Following the general procedure II, using the 4-methoxybenzothioic *S*-acid **1b** (85.0 mg, 0.505 mmol), hexamethyldisilazane (0.212 mL, 1.01 mmol) and *N,N*-dimethylformamide (0.118 mL, 1.52 mmol). The reaction mixture was stirred at 80 °C for 5 hours. The crude product was then purified by column

chromatography (gradient elution: 10% to 50% EtOAc in CH_2Cl_2) to afford compound **2b** (77.6 mg, 95% yield) as a white solid. ^{1}H NMR (400 MHz, $CDCl_3$) δ 7.78 (d, *J* = 8.9 Hz, 2H), 6.94 (d, *J* = 8.9 Hz, 2H), 5.68 (br, 2H), 3.86 (s, 3H); $^{13}C\{^1H\}$ NMR (100 MHz, $(CD_3)_2SO$) δ 167.5, 161.6, 129.4, 126.5, 113.4, 55.3.

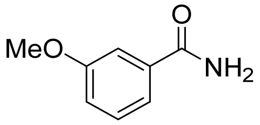

3-Methoxybenzamide (2c)[2,3]**:** Following the general procedure II, using the 3-methoxybenzothioic *S*-acid **1c** (90.8 mg, 0.54 mmol), hexamethyldisilazane (0.226 mL, 1.08 mmol) and *N,N*-dimethylformamide (0.126 mL, 1.62 mmol). The crude product was then purified by column chromatography (gradient elution: 10% to 50% EtOAc in CH_2Cl_2) to afford compound **2c** (61.6 mg, 76% yield) as a white solid. ^{1}H NMR (400 MHz, $CDCl_3$) δ 7.40 (s, 1H), 7.37 – 7.31 (m, 2H), 7.07 (td, *J* = 4.4, 2.4 Hz, 1H), 6.10 (br, 1H), 5.84 (br, 1H), 3.85 (s, 3H); $^{13}C\{^1H\}$ NMR (100 MHz, $(CD_3)_2SO$) δ 167.8, 159.2, 135.8, 129.4, 119.8, 117.1, 112.7, 55.2.

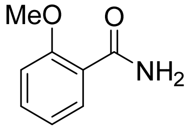

2-Methoxybenzamide (2d)[2]**:** Following the general procedure II, using the 2-methoxybenzothioic *S*-acid **1d** (72.5 mg, 0.43 mmol), hexamethyldisilazane (0.181 mL, 0.862 mmol) and *N,N*-dimethylformamide (0.101 mL, 1.29 mmol). The crude product was then purified by column chromatography (gradient elution: 10% to 50% EtOAc in CH_2Cl_2) to afford compound **2d** (53.8 mg, 83% yield) as a white solid. ^{1}H NMR (400 MHz, $CDCl_3$) δ 8.21 (dd, *J* = 7.8, 1.9 Hz, 1H), 7.72 (br, 1H), 7.51– 7.46 (m, 1H), 7.11 – 7.07 (m, 1H), 6.99 (d, *J* = 8.4 Hz, 1H), 5.78 (br, 1H), 3.98 (s, 3H); $^{13}C\{^1H\}$ NMR (100 MHz, $CDCl_3$) δ 167.3, 158.0, 133.5, 132.6, 121.3, 121.0, 111.5, 56.1.

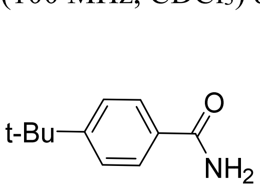

4-(*tert*-Butyl)benzamide (2e)[2]**:** Following the general procedure II, using the 4-(*tert*-butyl)benzothioic *S*-acid **1e** (74.0 mg, 0.38 mmol), hexamethyldisilazane (0.160 mL, 0.762 mmol) and *N,N*-dimethylformamide (0.0889 mL, 1.14 mmol). The crude product was then purified by column chromatography (gradient elution: 10% to 50% EtOAc in CH_2Cl_2) to afford compound **2e** (69.9 mg, 94% yield) as a white solid. ^{1}H NMR (400 MHz, $CDCl_3$) δ 7.75 (d, *J* = 8.4 Hz, 2H), 7.47 (d, *J* =8.4 Hz, 2H), 6.05 (br, 1H), 5.69

(br, 1H), 1.34 (s, 9H); $^{13}C\{^1H\}$ NMR (100 MHz, $CDCl_3$) δ 169.6, 155.7, 130.6, 127.4, 125.7, 35.1, 31.3.

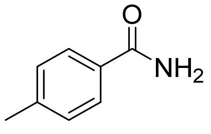

4-Methylbenzamide (2f)[2]: Following the general procedure II, using the 4-methylbenzothioic *S*-acid **1f** (90.4 mg, 0.583 mmol), hexamethyldisilazane (0.244 mL, 1.17 mmol) and *N,N*-dimethylformamide (0.136 mL, 1.75 mmol). The crude product was then purified by column chromatography (gradient elution: 10% to 50% EtOAc in CH_2Cl_2) to afford compound **2f** (76.8 mg, 95% yield) as a white solid. ^1H NMR (400 MHz, $CDCl_3$) δ 7.71 (d, *J* = 8.2 Hz, 2H), 7.25 (d, *J* = 7.1 Hz, 2H), 6.01 (br, 1H), 5.62 (br, 1H), 2.41 (s, 3H); $^{13}C\{^1H\}$ NMR (100 MHz, CDCl3) δ 169.6, 142.6, 130.6, 129.4, 127.5, 21.6.

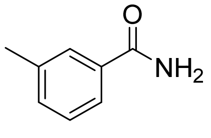

3-Methylbenzamide (2g)[2]: Following the general procedure II, using the 3-methylbenzothioic *S*-acid **1g** (72.0 mg, 0.473 mmol), hexamethyldisilazane (0.198 mL, 0.947 mmol) and *N,N*-dimethylformamide (0.11 mL, 1.42 mmol). The crude product was then purified by column chromatography (gradient elution: 10% to 50% EtOAc in CH_2Cl_2) to afford compound **2g** (50.8 mg, 80% yield) as a white solid. ^1H NMR (400 MHz, $CDCl_3$) δ 7.65 (s, 1H), 7.59 – 7.57 (m, 1H), 7.34 – 7.33 (m, 2H), 6.04 (br, 1H), 5.57 (br, 1H), 2.40 (s, 3H); $^{13}C\{^1H\}$ NMR (100 MHz, $CDCl_3$) δ 170.4, 138.5, 133.5, 132.7, 128.5, 128.2, 124.4, 21.4.

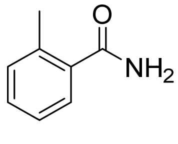

2-Methylbenzamide (2h)[2]: Following the general procedure II, using the 2-methylbenzothioic *S*-acid **1h** (79.1 mg, 0.52 mmol), hexamethyldisilazane (0.218 mL, 1.04 mmol) and *N,N*-dimethylformamide (0.121 mL, 1.56 mmol). The crude product was then purified by column chromatography (gradient elution: 10% to 50% EtOAc in CH_2Cl_2) to afford compound **2h** (57.2 mg, 81% yield) as a white solid. ^1H NMR (400 MHz, $CDCl_3$) δ 7.46 (d, *J* = 7.5 Hz, 1H), 7.34 (dt, *J* = 7.5, 1.2 Hz, 1H), 7.26 – 7.20 (m, 2H), 5.71 (br, 2H), 2.51 (s, 3H); $^{13}C\{^1H\}$ NMR (100 MHz, $CDCl_3$) δ 172.5, 136.4, 135.4, 131.3, 130.4, 127.1, 125.8, 20.1.

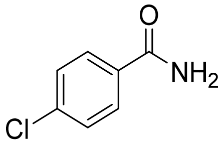

4-Chlorobenzamide (2i)[3,5]**:** Following the general procedure II, using the 4-chlorobenzothioic *S*-acid **1i** (93.7 mg, 0.543 mmol), hexamethyldisilazane (0.228 mL, 1.09 mmol) and *N,N*-dimethylformamide (0.127 mL, 1.63 mmol). The mixture was stirred at 100°C for 3 hours. The crude product was then purified by column chromatography (gradient elution: 10% to 50% EtOAc in CH_2Cl_2) to afford compound **2i** (71.2 mg, 84% yield) as a white solid. ^1H NMR (400 MHz, $CDCl_3$) δ 7.75 (d, J = 8.6 Hz, 2H), 7.43 (d, J = 8.5 Hz, 2H), 6.02 (br, 1H), 5.76 (br, 1H); $^{13}C\{^1H\}$ NMR (100 MHz, $(CD_3)_2SO$) δ 166.9, 136.1, 133.0, 129.4, 128.3.

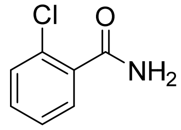

2-Chlorobenzamide (2j)[2]**:** Following the general procedure II, using the 2-chlorobenzothioic *S*-acid **1j** (90.0 mg, 0.521 mmol), hexamethyldisilazane (0.219 mL, 1.04 mmol) and *N,N*-dimethylformamide (0.122 mL, 1.56 mmol). The crude product was then purified by column chromatography (gradient elution: 10% to 50% EtOAc in CH_2Cl_2) to afford compound **2j** (82.9 mg, 99% yield) as a white solid. ^1H NMR (400 MHz, $CDCl_3$) δ 7.79 (dd, J = 7.4, 1.8 Hz, 1H), 7.44 – 7.32 (m, 3H), 6.37 (br, 1H), 6.20 (br, 1H); $^{13}C\{^1H\}$ NMR (100 MHz, $CDCl_3$) δ 168.6, 134.0, 131.9, 131.0, 130.7, 130.5, 127.3.

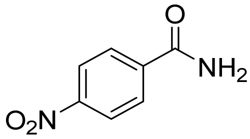

4-Nitrobenzamide (2k)[6,7]**:** Following the general procedure II, using the 4-Nitrobenzothioic *S*-acid **1k** (54.5 mg, 0.298 mmol), hexamethyldisilazane (0.125 mL, 0.595 mmol) and *N,N*-dimethylformamide (0.069 mL, 0.893mmol). The crude product was then purified by column chromatography (gradient elution: 10% to 50% EtOAc in CH_2Cl_2) to afford compound **2k** (44.5 mg, 82% yield) as a pale yellow solid. ^1H NMR (400 MHz, $CDCl_3$) δ 8.32 (d, J = 8.7 Hz, 2H), 7.98 (d, J =8.7 Hz, 2H), 6.13 (br, 1H), 5.83 (br, 1H); $^{13}C\{^1H\}$ NMR (100 MHz, $(CD_3)_2SO$) δ 166.2, 149.1, 140.0, 128.9, 123.5.

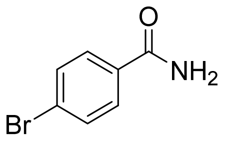

4-Bromobenzamide (2l)[3,5]**:** Following the general procedure II, using the 4-bromobenzothioic *S*-acid **1l** (53.4 mg, 0.246 mmol), hexamethyldisilazane (0.103 mL, 0.492 mmol) and *N,N*-dimethylformamide (0.057 mL, 0.738 mmol). The crude product was then purified by column chromatography (gradient elution: 10% to 50% EtOAc in CH_2Cl_2) to afford compound **2l** (46.4 mg, 94% yield) as a white solid. ^1H NMR (400 MHz, $CDCl_3$) δ 7.69 (d, J = 8.7 Hz, 2H), 7.59 (d, J = 8.6 Hz, 2H), 5.78 (br, 2H); $^{13}C\{^1H\}$ NMR (100 MHz, $(CD_3)_2SO$) δ 167.0, 133.4, 131.3, 129.6, 125.1.

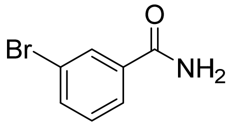

3-Bromobenzamide (2m)[4,8]**:** Following the general procedure II, using the 3-bromobenzothioic *S*-acid **1m** (76.0 mg, 0.35 mmol), hexamethyldisilazane (0.147 mL, 0.701 mmol) and *N,N*-dimethylformamide (0.082 mL, 1.05 mmol). The crude product was then purified by column chromatography (gradient elution: 10% to 50% EtOAc in CH_2Cl_2) to afford compound **2m** (54.1 mg, 77% yield) as a white solid. ^1H NMR (400 MHz, $CDCl_3$) δ 7.97 (s, 1H), 7.73 (d, J = 7.8 Hz, 1H), 7.66 (d, J = 8.0 Hz, 1H), 7.33 (t, J = 7.9 Hz, 1H), 6.06 (br, 1H), 5.84 (br, 1H); $^{13}C\{^1H\}$ NMR (100 MHz, $(CD_3)_2SO$) δ 166.3, 136.5, 134.0, 130.5, 130.2, 126.6, 121.6.

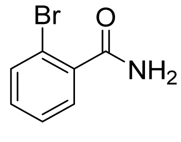

2-Bromobenzamide (2n)[2]**:** Following the general procedure II, using the 2-bromobenzothioic *S*-acid **1n** (80.0 mg, 0.369 mmol), hexamethyldisilazane (0.155 mL, 0.737 mmol) and *N,N*-dimethylformamide (0.086 mL, 1.11 mmol). The crude product was then purified by column chromatography (gradient elution: 10% to 50% EtOAc in CH_2Cl_2) to afford compound **2n** (60.2 mg, 82% yield) as white solid. ^1H NMR (400 MHz, $CDCl_3$) δ 7.64 – 7.60 (m, 2H), 7.39 – 7.35 (m, 1H), 7.32 – 7.27 (m, 1H), 6.18 (br, 2H); $^{13}C\{^1H\}$ NMR (101 MHz, $CDCl_3$) δ 169.5, 136.8, 133.7, 131.8, 130.0, 127.7, 119.3.

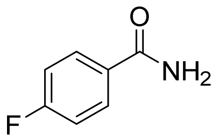

4-Fluorobenzamide (2o)[2,9,10]**:** Following the general procedure II, using the 4-fluorobenzothioic *S*-acid **1o** (52.8 mg, 0.338 mmol), hexamethyldisilazane (0.142 mL, 0.677 mmol) and *N,N*-dimethylformamide (0.079 mL, 1.02 mmol). The crude product was then purified by column chromatography (gradient elution: 10% to 50% EtOAc in CH_2Cl_2) to afford compound **2o** (46.8 mg, 99% yield) as a pale yellow solid. ^{1}H NMR (400 MHz, $CDCl_3$) δ 7.85 – 7.81 (m, 2H), 7.13 (t, $^3J_{H\text{-}H} = {}^3J_{H\text{-}F} = 8.6$ Hz, 2H), 5.92 (br, 2H); ^{13}C{^{1}H} NMR (100 MHz, CD_3OD) δ 169.7, 164.9 (d, $^1J_{CF} = 248$ Hz), 129.9 (d, $^3J_{CF} = 9.0$ Hz, 2C), 114.9 (d, $^2J_{CF} = 22.1$ Hz, 2C); ^{19}F (376 MHz, $CDCl_3$) −107.34 (tt, J = 8.3 Hz, 5.3 Hz).

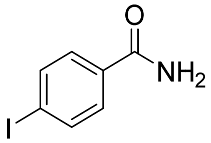

4-Iodobenzamide (2p)[11,12]**:** Following the general procedure II, using the 4-iodobenzothioic *S*-acid **1p** (81.3 mg, 0.308 mmol), hexamethyldisilazane (0.130 mL, 0.616 mmol) and *N,N*-dimethylformamide (0.072 mL, 0.924 mmol). The crude product was then purified by column chromatography (gradient elution: 10% to 50% EtOAc in CH_2Cl_2) to afford compound **2p** (48.0 mg, 63% yield) as a white solid. ^{1}H NMR (400 MHz, $(CD_3)_2SO$) δ 8.02 (br, 1H), 7.84 (d, J = 8.4 Hz, 2H), 7.64 (d, J = 8.4 Hz, 2H), 7.42 (br, 1H); ^{13}C{^{1}H} NMR (100 MHz, $(CD_3)_2SO$) δ 167.2, 137.1, 133.8, 129.5, 98.9

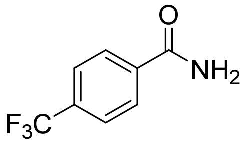

4-(Trifluoromethyl)benzamide (2q)[6,10,13]**:** Following the general procedure II, using the 4-(trifluoromethyl)benzothioic *S*-acid **1q** (85.0 mg, 0.412 mmol), hexamethyldisilazane (0.173 mL, 0.824 mmol) and *N,N*-dimethylformamide (0.096 mL, 1.24 mmol). The crude product was then purified by column chromatography (gradient elution: 10% to 50% EtOAc in CH_2Cl_2) to afford compound **2q** (52.0 mg, 67% yield) as a white solid. ^{1}H NMR (400 MHz, $CDCl_3$) δ 7.93 (d, J = 8.2 Hz, 2H), 7.73 (d, J = 8.2 Hz, 2H), 6.10 (br, 1H), 5.75 (br, 1H); ^{13}C{^{1}H} NMR (100 MHz, $(CD_3)_2SO$) δ 166.7, 138.1, 131.2 (q, $^2J_{CF} = 31.9$ Hz), 128.4 (CH×2), 125.3 (q, $^3J_{CF} = 3.0$ Hz, 2C), 123.9 (q, $^1J_{CF} = 271$ Hz); ^{19}F (376 MHz, $CDCl_3$) δ −60.3 (s, 3F).

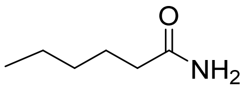

Hexanamide (2r)[14]**:** Following the general procedure II, using the hexanethioic *S*-acid **1r** (79.0 mg, 0.598 mmol), hexamethyldisilazane (0.251 mL, 1.20 mmol) and *N,N*-dimethylformamide (0.140 mL, 1.79 mmol). The crude product was then purified by column chromatography (gradient elution: 10% to 50% EtOAc in CH_2Cl_2) to afford compound **2r** (59.4 mg, 86% yield) as a white solid. ^{1}H NMR (400 MHz, $CDCl_3$) δ 5.44 (br, 2H), 2.22 (t, J = 7.6 Hz, 2H), 1.68–1.60 (m, 2H), 1.36–1.30 (m, 4H), 0.90 (t, J = 7.0 Hz, 3H); ^{13}C{^{1}H} NMR (100 MHz, $CDCl_3$) δ176.3, 36.0, 31.5, 25.3, 22.5, 14.0.

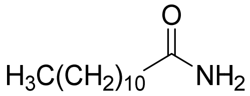

Dodecanamide (2s)[15]**:** Following the general procedure II, using the thiododecanoic acid **1s** (66.5 mg, 0.307 mmol), hexamethyldisilazane (0.129 mL, 0.614 mmol) and *N,N*-dimethylformamide (0.072 mL, 0.922 mmol). The crude product was then purified by column chromatography (gradient elution: 10% to 50% EtOAc in CH_2Cl_2) to afford compound **2s** (40.8 mg, 67% yield) as a white solid. ^{1}H NMR (400 MHz, $CDCl_3$) δ 5.31 (br, 2H), 2.24–2.20 (m, 2H), 1.65-1.58 (m, 2H), 1.35–1.26 (m, 16H), 0.88 (t, J = 6.9 Hz, 3H). ^{13}C{^{1}H} NMR (100 MHz, $CDCl_3$) δ 175.9, 36.1, 32.0, 29.7, 29.6, 29.5, 29.4, 25.7, 22.9, 14.3.

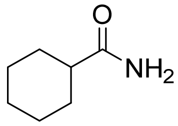

Cyclohexanecarboxamide (2t)[16]**:** Following the general procedure II, using the cyclohexanecarbothioic *S*-acid **1t** (53.2 mg, 0.369 mmol), hexamethyldisilazane (0.155 mL, 0.738 mmol) and *N,N*-dimethylformamide (0.086 mL, 1.11 mmol). The crude product was then purified by column chromatography (gradient elution: 10% to 50% EtOAc in CH_2Cl_2) to afford compound **2t** (39.8 mg, 85% yield) as a white solid. ^{1}H NMR (400 MHz, $CDCl_3$) δ 5.41 (br, 2H), 2.15 (tt, J = 11.7, 3.5 Hz, 1H), 1.93–1.89 (m, 2H), 1.82–1.78 (m, 2H), 1.69–1.67 (m, 1H), 1.47–1.38 (m, 2H), 1.34–1.20 (m, 3H). ^{13}C{^{1}H} NMR (100 MHz, $CDCl_3$) δ 178.9, 44.9, 29.8, 25.9, 25.8.

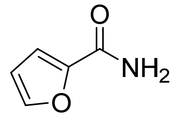

Furan-2-carboxamide (2u)[4]: Following the general procedure II, using the furan-2-carbothioic *S*-acid **1u** (89.3 mg, 0.697 mmol), hexamethyldisilazane (0.292 mL, 1.39 mmol) and *N,N*-dimethylformamide (0.163 mL, 2.09 mmol). The crude product was then purified by column chromatography (gradient elution: 10% to 50% EtOAc in CH_2Cl_2) to afford compound **2u** (54.3 mg, 70% yield) as a white solid. ^{1}H NMR (400 MHz, $CDCl_3$) δ 7.47–7.46 (m, 1H), 7.16 (dd, *J* = 3.5, 0.8 Hz, 1H), 6.52–6.51 (m, 1H), 6.29 (br, 1H), 5.91(br, 1H) ppm. ^{13}C{^{1}H} NMR (100 MHz, $CDCl_3$) δ 160.4, 147.6, 144.5, 115.3, 112.4 ppm.

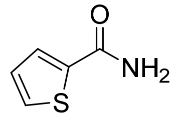

Thiophene-2-carboxamide (2v)[2]: Following the general procedure II, using the thiophene-2-carbothioic *S*-acid **1v** (68.5 mg, 0.475 mmol), hexamethyldisilazane (0.199 mL, 0.950 mmol) and *N,N*-dimethylformamide (0.111 mL, 1.43 mmol). The crude product was then purified by column chromatography (gradient elution: 10% to 50% EtOAc in CH_2Cl_2) to afford compound **2v** (54.4 mg, 90% yield) as a white solid. ^{1}H NMR (400 MHz, $(CD_3)_2SO$) δ 7.95 (s, 1H), 7.74–7.73 (m, 2H), 7.37 (s, 1H), 7.14–7.11 (m, 1H); ^{13}C{^{1}H} NMR (100 MHz, $(CD_3)_2SO$) δ 162.9, 140.3, 131.0, 128.6, 127.9.

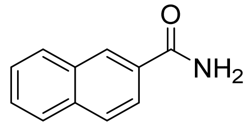

2-Naphthamide (2w)[6,13]: Following the general procedure II, using the naphthalene-2-carbothioic thioacid **1w** (89.0 mg, 0.472 mmol), hexamethyldisilazane (0.198 mL, 0.944 mmol) and *N,N*-dimethylformamide (0.110 mL, 1.42 mmol). The crude product was then purified by column chromatography (gradient elution: 10% to 50% EtOAc in CH_2Cl_2) to afford compound **2w** (65.8 mg, 82% yield) as a white solid. ^{1}H NMR (400 MHz, $CDCl_3$) δ 8.35 (s, 1H), 7.92(dd, *J* = 14.9, 6.0 Hz, 2H), 7.86 (dd, *J* =8.6, 1.8 Hz, 2H), 7.61–7.54 (m, 2H), 6.19 (br, 1H), 5.73 (br, 1H); ^{13}C{^{1}H} NMR (100 MHz, $(CD_3)_2SO$) δ 168.0, 134.2, 132.2, 131.7, 128.9, 127.8, 127.6, 126.6, 124.4.

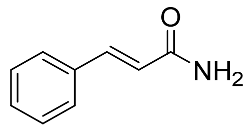

Cinnamamide (2x)[2]**:** Following the general procedure II, using the thiolocinnamic acid **1x** (77.4 mg, 0.471 mmol), hexamethyldisilazane (0.198 mL, 0.942 mmol) and *N,N*-dimethylformamide (0.110 mL, 1.41 mmol). The crude product was then purified by column chromatography (gradient elution: 10% to 50% EtOAc in CH_2Cl_2) to afford compound **2x** (25.3 mg, 37% yield) as a white solid. ^{1}H NMR (400 MHz, $CDCl_3$) δ 7.65 (d, J = 15.7 Hz, 1H), 7.53 – 7.50 (m, 2H), 7.41 – 7.37 (m, 3H), 6.46 (d, J = 15.7 Hz, 1H), 5.60 (br, 2H); $^{13}C\{^1H\}$ NMR (100 MHz, $CDCl_3$) δ 168.0, 142.6, 134.6, 130.1, 129.0, 128.1, 119.7.

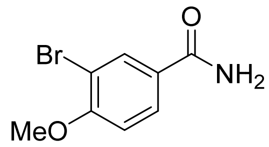

3-Bromo-4-methoxybenzamide (2y)[8,17]**:** Following the general procedure II, using the 3-bromo-4-methoxybenzothioic *S*-acid **1y** (94.6 mg, 0.345 mmol), hexamethyldisilazane (0.145 mL, 0.690 mmol) and *N,N*-dimethylformamide (0.081 mL, 1.04 mmol). The crude product was then purified by column chromatography (gradient elution: 10% to 50% EtOAc in CH_2Cl_2) to afford compound **2y** (67.2 mg, 85% yield) as a white solid. ^{1}H NMR (400 MHz, $CDCl_3$) *δ*: 8.01 (s, 1H), 7.78 (d, J = 8.2 Hz, 1H), 6.93 (d, J = 8.2 Hz, 1H), 5.84 (br, 2H), 3.95 (s, 3H); $^{13}C\{^1H\}$ NMR (100 MHz, $(CD_3)_2SO$) δ 166.2, 157.6, 132.2, 128.8, 127.7, 112.0, 110.2, 56.5.

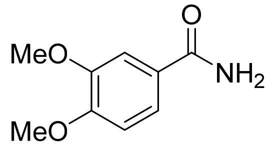

3,4-Dimethoxybenzamide(2z)[10]: Following the general procedure II, using the 3,4-dimethoxybenzenecarbothioic *S*-acid **1z** (92.5 mg, 0.465 mmol), hexamethyldisilazane (0.195 mL, 0.930 mmol) and *N,N*-dimethylformamide (0.108 mL, 1.39 mmol). The crude product was then purified by column chromatography (gradient elution: 10% to 50% EtOAc in CH_2Cl_2) to afford compound **2z** (53.5 mg, 64% yield) as a white solid. ^{1}H NMR (400 MHz, $CDCl_3$) δ 7.46 (d, J = 1.96 Hz, 1H), 7.32 (dd, J = 8.3, 2.1 Hz, 1H), 6.87 (d, J = 8.3 Hz, 1H), 5.88 (br, 2H), 3.93 (s, 3H), 3.92 (s, 2H). $^{13}C\{^1H\}$ NMR (100 MHz, $CDCl_3$) δ 169.2, 152.3, 149.1, 126.1, 120.2, 110.9, 110.4, 56.2.

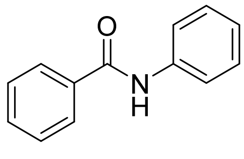

***N*-Phenylbenzamide (4aa)**[18]**:** Following the general procedure III, using the aniline **3a** (300.0 mg, 3.22 mmol), pyridinium *p*-toluenesulfonate (16.2 mg, 0.06 mmol), *N*,*N*-dimethylformamide (0.374 mL, 4.83 mmol), hexamethyldisilazane (1.026 mL, 3.22 mmol), thiobenzoic acid **1a** (1.141 mL, 9.66 mmol) and 1,8-diazabicyclo[5.4.0]undec-7-ene (0.722 mL, 4.83 mmol). The crude product was then purified by column chromatography (Hexane:EtOAc = 5:1) to afford compound **4aa** (577.5 mg, 91% yield) as a white solid. ^{1}H NMR (400 MHz, $CDCl_3$) δ 7.87 (d, *J* = 7.1 Hz, 2H), 7.84 (s, 1H), 7.65 (d, *J* = 8.2 Hz, 2H), 7.56 (t, *J* = 7.3 Hz, 1H), 7.49 (t, *J* = 7.3 Hz, 2H), 7.38 (t, *J* = 7.9 Hz, 2H), 7.16 (t, *J* = 7.4 Hz, 1H); ^{13}C{^{1}H} NMR (100 MHz, $CDCl_3$) δ 165.9, 138.0, 135.1, 132.0, 129.3 (CH×2), 129.0 (CH×2), 127.1 (CH×2), 124.7, 120.3 (CH×2).

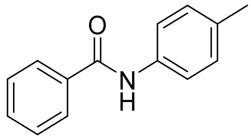

***N*-(p-Tolyl)benzamide (4ab)**[19a]**:** Following the general procedure III, using the *p*-toluidine **3b** (300.0 mg, 2.80 mmol), pyridinium *p*-toluenesulfonate (14.1 mg, 0.06 mmol), *N*,*N*-dimethylformamide (0.325 mL, 4.20 mmol), hexamethyldisilazane (0.892 mL, 4.20 mmol), thiobenzoic acid **1a** (0.992 mL, 8.40 mmol) and 1,8-Diazabicyclo[5.4.0]undec-7-ene (0.628 mL, 4.20 mmol). The crude product was then purified by column chromatography (Hexane:EtOAc = 5:1) to afford compound **4ab** (571.0 mg, 97% yield) as a white solid. ^{1}H NMR (400 MHz, $CDCl_3$) δ 8.12 (s, 1H), 7.84 (d, *J* = 7.2 Hz, 2H), 7.56 – 7.46 (m, 3H), 7.41 (t, *J* = 7.5 Hz, 2H), 7.13 (d, *J* = 8.3 Hz, 2H), 2.33 (s, 3H); ^{13}C{^{1}H} NMR (100 MHz, $CDCl_3$) δ 166.0, 135.5, 135.1, 134.3, 131.7, 129.6 (CH×2), 128.8 (CH×2), 127.2 (CH×2), 120.6 (CH×2), 21.0.

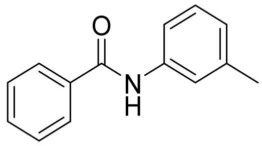

***N*-(m-Tolyl)benzamide (4ac)**[18]**:** Following the general procedure III, using the *m*-toluidine **3c** (300.0 mg, 2.80 mmol), pyridinium *p*-toluenesulfonate (14.1 mg, 0.06 mmol), *N*,*N*-dimethylformamide (0.325 mL, 4.20 mmol), hexamethyldisilazane (0.892 mL, 4.20 mmol), thiobenzoic acid **1a** (0.992 mL, 8.40 mmol) and 1,8-

Diazabicyclo[5.4.0]undec-7-ene (0.628 mL, 4.20 mmol). The crude product was then purified by column chromatography (Hexane:EtOAc = 5:1) to afford compound **4ac** (537.0 mg, 91% yield) as a yellow solid. ^1H NMR (400 MHz, $CDCl_3$) δ 8.42 (s, 1H), 7.84 (d, J = 7.3 Hz, 2H), 7.56 – 7.42 (m, 3H), 7.37 (t, J = 7.6 Hz, 2H), 7.19 (t, J = 7.8 Hz, 1H), 6.94 (d, J = 7.5 Hz, 1H), 2.29 (s, 3H); $^{13}C\{^1H\}$ NMR (100 MHz, $CDCl_3$) δ 166.2, 138.9, 138.0, 135.0, 131.7, 128.8, 128.6 (CH×2), 127.2 (CH×2), 125.4, 121.3, 117.7, 21.5.

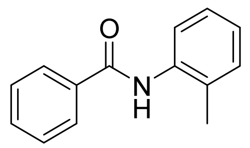

***N*-(o-Tolyl)benzamide (4ad)**[18]**:** Following the general procedure III, using the *o*-toluidine **3d** (300.0 mg, 2.80 mmol), pyridinium *p*-toluenesulfonate (14.1 mg, 0.06 mmol), *N*,*N*-dimethylformamide (0.325 mL, 4.20 mmol), hexamethyldisilazane (0.892 mL, 4.20 mmol), thiobenzoic acid **1a** (0.992 mL, 8.40 mmol) and 1,8-Diazabicyclo[5.4.0]undec-7-ene (0.628 mL, 4.20 mmol). The crude product was then purified by column chromatography (Hexane:EtOAc = 5:1) to afford compound **4ad** (553.0 mg, 93% yield) as a white solid. ^1H NMR (400 MHz, $CDCl_3$) δ 7.92 (s, 1H), 7.87 (d, J = 7.3 Hz, 2H), 7.83 (d, J = 7.8 Hz, 1H), 7.54 (t, J = 7.4 Hz, 1H), 7.46 (t, J = 7.5 Hz, 2H), 7.25 – 7.16 (m, 2H), 7.12 (t, J = 7.4 Hz, 1H), 2.30 (s, 3H); $^{13}C\{^1H\}$ NMR (100 MHz, $CDCl_3$) δ 166.0, 135.8, 134.9, 131.8, 130.6, 130.1, 128.8 (CH×2), 127.2 (CH×2), 126.8, 125.6, 123.7, 17.9.

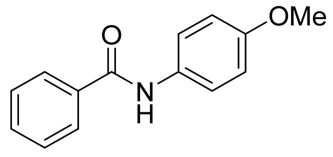

***N*-(4-Methoxyphenyl)benzamide (4ae)**[18]**:** Following the general procedure III, using the 4-methoxyaniline **3e** (300.0 mg, 2.44 mmol), pyridinium *p*-toluenesulfonate (12.2 mg, 0.05 mmol), *N*,*N*-dimethylformamide (0.283 mL, 3.65 mmol), hexamethyldisilazane (0.776 mL, 3.65 mmol), thiobenzoic acid **1a** (0.863 mL, 7.31 mmol) and 1,8-Diazabicyclo[5.4.0]undec-7-ene (0.546 mL, 3.65 mmol). The crude product was then purified by column chromatography (Hexane:EtOAc = 5:1) to afford compound **4ae** (439.0 mg, 79% yield) as a yellow solid. ^1H NMR (400 MHz, $CDCl_3$) δ 7.86 (d, J = 7.2 Hz, 2H), 7.83 (s, 1H), 7.58 – 7.51 (m, 3H), 7.46 (t, J = 7.4 Hz, 2H), 6.90 (d, J = 9.0 Hz, 2H), 3.81 (s, 3H); $^{13}C\{^1H\}$ NMR (100 MHz, $CDCl_3$) δ 165.8, 156.8, 135.2, 131.8, 131.1, 128.9 (CH×2), 127.1 (CH×2), 122.3 (CH×2), 114.4 (CH×2), 55.6.

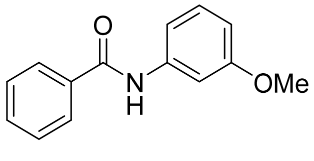

***N*-(3-Methoxyphenyl)benzamide (4af)**[20]**:** Following the general procedure III, using the 3-methoxyaniline **3f** (300.0 mg, 2.44 mmol), pyridinium *p*-toluenesulfonate (12.2 mg, 0.05 mmol), *N*,*N*-dimethylformamide (0.283 mL, 3.65 mmol), hexamethyldisilazane (0.776 mL, 3.65 mmol), thiobenzoic acid **1a** (0.863 mL, 7.31 mmol) and 1,8-Diazabicyclo[5.4.0]undec-7-ene (0.546 mL, 3.65 mmol). The crude product was then purified by column chromatography (Hexane:EtOAc = 5:1) to afford compound **4af** (464.0 mg, 84% yield) as a white solid. ^{1}H NMR (400 MHz, $CDCl_3$) δ 8.04 (s, 1H), 7.86 (d, J = 7.1 Hz, 2H), 7.54 (t, J = 7.4 Hz, 1H), 7.50 – 7.42 (m, 3H), 7.25 (t, J = 8.2 Hz, 1H), 7.12 (d, J = 8.0, 1H), 6.71 (d, J = 8.2, 1H), 3.81 (s, 3H); ^{13}C{^{1}H} NMR (100 MHz, $CDCl_3$) δ 166.0, 160.3, 139.3, 135.1, 132.0, 129.8, 129.0 (CH×2), 127.1 (CH×2), 112.5, 110.7, 106.0, 55.4.

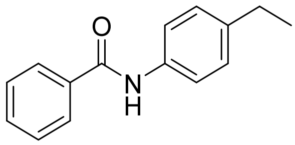

***N*-(4-Ethylphenyl)benzamide (4ag)**[21]**:** Following the general procedure III, using the 4-ethylaniline **3g** (300.0 mg, 2.48 mmol), pyridinium *p*-toluenesulfonate (12.4 mg, 0.05 mmol), *N*,*N*-dimethylformamide (0.288 mL, 3.71 mmol), hexamethyldisilazane (0.789 mL, 3.71 mmol), thiobenzoic acid **1a** (0.877 mL, 7.43 mmol) and 1,8-Diazabicyclo[5.4.0]undec-7-ene (0.555 mL, 3.71 mmol). The crude product was then purified by column chromatography (Hexane:EtOAc = 5:1) to afford compound **4ag** (484.0 mg, 87% yield) as a white solid. ^{1}H NMR (400 MHz, $CDCl_3$) δ 8.21 (s, 1H), 7.84 (d, J = 7.4 Hz, 2H), 7.56 (d, J = 8.3 Hz, 2H), 7.49 (t, J = 7.4 Hz, 1H), 7.40 (t, J = 7.6 Hz, 2H), 7.16 (d, J = 8.3 Hz, 2H), 2.63 (q, J = 7.6 Hz, 2H), 1.24 (t, J = 7.6 Hz, 3H); ^{13}C{^{1}H} NMR (100 MHz, $CDCl_3$) δ 166.0, 140.7, 135.7, 135.1, 131.7, 128.7 (CH×2), 128.4 (CH×2), 127.2 (CH×2), 120.7 (CH×2), 28.4, 15.7.

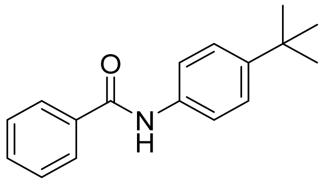

***N*-(4-(tert-Butyl)phenyl)benzamide (4ah)**[22]**:** Following the general procedure III,

using the 4-*tert*-butylaniline **3h** (300.0 mg, 2.01 mmol), pyridinium *p*-toluenesulfonate (10.1 mg, 0.04 mmol), *N*,*N*-dimethylformamide (0.233 mL, 3.02 mmol), hexamethyldisilazane (0.640 mL, 3.02 mmol), thiobenzoic acid **1a** (0.712 mL, 6.03 mmol) and 1,8-Diazabicyclo[5.4.0]undec-7-ene (0.451 mL, 3.02 mmol). The crude product was then purified by column chromatography (Hexane:EtOAc = 5:1) to afford compound **4ah** (406.0 mg, 80% yield) as a yellow solid. ^{1}H NMR (400 MHz, $CDCl_3$) δ 8.43 (s, 1H), 7.84 (d, *J* = 8.0 Hz, 2H), 7.60 (d, *J* = 7.7 Hz, 2H), 7.48 (t, *J* = 7.4 Hz, 1H), 7.41 – 7.30 (m, 4H), 1.33 (s, 9H); ^{13}C{^{1}H} NMR (100 MHz, $CDCl_3$) δ 166.2, 147.5, 135.5, 135.1, 131.6, 128.6 (CH×2), 127.2 (CH×2), 125.8 (CH×2), 120.5 (CH×2), 34.4, 31.4 (CH×3).

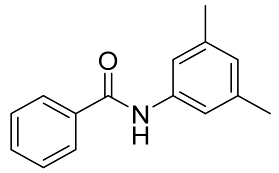

***N*-(3,5-Dimethylphenyl)benzamide (4ai)**[23]**:** Following the general procedure III, using the 3,5-dimethylaniline **3i** (300.0 mg, 2.48 mmol), pyridinium *p*-toluenesulfonate (12.4 mg, 0.05 mmol), *N*,*N*-dimethylformamide (0.288 mL, 3.71 mmol), hexamethyldisilazane (0.789 mL, 3.71 mmol), thiobenzoic acid **1a** (0.877 mL, 7.43 mmol) and 1,8-Diazabicyclo[5.4.0]undec-7-ene (0.555 mL, 3.71 mmol). The crude product was then purified by column chromatography (Hexane:EtOAc = 5:1) to afford compound **4ai** (526.0 mg, 94% yield) as a white solid. ^{1}H NMR (400 MHz, $CDCl_3$) δ 8.19 (s, 1H), 7.85 (d, *J* = 7.7 Hz, 2H), 7.49 (t, *J* = 7.4 Hz, 1H), 7.40 (t, *J* = 7.5 Hz, 2H), 7.29 (s, 2H), 6.77 (s, 1H), 2.27 (s, 6H); ^{13}C{^{1}H} NMR (100 MHz, $CDCl_3$) δ 166.0, 138.7 (CH×2), 137.9, 135.1, 131.7, 128.7 (CH×2), 127.2 (CH×2), 126.3, 118.3 (CH×2), 21.4 (CH×2).

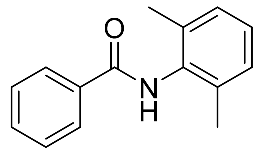

***N*-(2,6-Dimethylphenyl)benzamide (4aj)**[24]**:** Following the general procedure III, using the 2,6-dimethylaniline **3j** (300.0 mg, 2.48 mmol), pyridinium *p*-toluenesulfonate (12.4 mg, 0.05 mmol), *N*,*N*-dimethylformamide (0.288 mL, 3.71 mmol), hexamethyldisilazane (0.789 mL, 3.71 mmol), thiobenzoic acid **1a** (0.877 mL, 7.43 mmol) and 1,8-Diazabicyclo[5.4.0]undec-7-ene (0.555 mL, 3.71 mmol). The crude product was then purified by column chromatography (Hexane:EtOAc = 5:1) to afford

compound **4aj** (299.0 mg, 54% yield) as a white solid. ^{1}H NMR (400 MHz, $CDCl_3$) δ 7.92 (d, *J* = 7.0 Hz, 2H), 7.57 (t, *J* = 7.4 Hz, 1H), 7.53 – 7.44 (m, 3H), 7.18 – 7.09 (m, 3H), 2.28 (s, 6H); ^{13}C{^{1}H} NMR (100 MHz, $CDCl_3$) δ 166.0, 135.7 (CH×2), 134.6, 134.0, 131.9, 128.9 (CH×2), 128.4 (CH×2), 127.6, 127.4 (CH×2), 18.6 (CH×2).

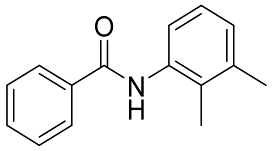

***N*-(2,3-Dimethylphenyl)benzamide (4ak)[25]:** Following the general procedure III, using the 2,3-dimethylaniline **3k** (300.0 mg, 2.48 mmol), pyridinium *p*-toluenesulfonate (12.4 mg, 0.05 mmol), *N*,*N*-dimethylformamide (0.288 mL, 3.71 mmol), hexamethyldisilazane (0.789 mL, 3.71 mmol), thiobenzoic acid **1a** (0.877 mL, 7.43 mmol) and 1,8-Diazabicyclo[5.4.0]undec-7-ene (0.555 mL, 3.71 mmol). The crude product was then purified by column chromatography (Hexane:EtOAc = 5:1) to afford compound **4ak** (299.0 mg, 54% yield) as a white solid. ^{1}H NMR (400 MHz, $CDCl_3$) δ 7.89 (d, *J* = 7.5 Hz, 2H), 7.77 (s, 1H), 7.61–7.45 (m, 4H), 7.14 (t, *J* = 7.7 Hz, 1H), 7.06 (d, *J* = 7.5 Hz, 1H), 2.32 (s, 3H), 2.21 (s, 3H); ^{13}C{^{1}H} NMR (100 MHz, $CDCl_3$) δ 166.1, 137.6, 135.5, 135.1, 131.9, 129.8, 128.9 (CH×2), 127.7, 127.3 (CH×2), 126.1, 122.4, 20.7, 14.0.

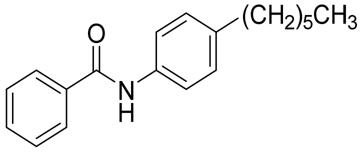

***N*-(4-Hexylphenyl)benzamide (4al):** Following the general procedure III, using the 4-hexylaniline **3l** (300.0 mg, 1.69 mmol), pyridinium *p*-toluenesulfonate (8.5 mg, 0.03 mmol), *N*,*N*-dimethylformamide (0.197 mL, 2.54 mmol), hexamethyldisilazane (0.539 mL, 2.54 mmol), thiobenzoic acid **1a** (0.600 mL, 5.08 mmol) and 1,8-Diazabicyclo[5.4.0]undec-7-ene (0.379 mL, 2.54 mmol). The crude product was then purified by column chromatography (Hexane:EtOAc = 5:1) to afford compound **4al** (357.0 mg, 75% yield) as a white solid, m.p. 115-116°C. ATR-IR (cm^{-1})：3340, 2960, 2922, 2849, 1651, 1597, 1514, 1410, 1320, 1260, 822. ^{1}H NMR (400 MHz, $CDCl_3$) δ 7.98 (s, 1H), 7.85 (d, *J* = 7.6 Hz, 2H), 7.54 (d, *J* = 7.6 Hz, 2H), 7.51 (d, *J* = 7.8 Hz, 1H), 7.44 (t, *J* = 7.4 Hz, 2H), 7.16 (d, *J* = 7.9 Hz, 2H), 2.59 (t, *J* = 7.6 Hz, 2H), 1.61 (quin., *J* = 7.9 Hz, 2H), 1.40 – 1.24 (m, 6H), 0.89 (d, *J* = 6.6 Hz, 3H); ^{13}C{^{1}H} NMR (100 MHz, $CDCl_3$) δ 165.9, 139.5, 135.7, 135.2, 131.8, 129.0 (CH×2), 128.8 (CH×2), 127.2

(CH×2), 120.5 (CH×2), 35.5, 31.8, 31.6, 29.0, 22.7, 14.2. HRMS (EI) m/z : $[M]^+$ calcd. for $C_{19}H_{23}NO$: 281.1780, found: 281.1788.

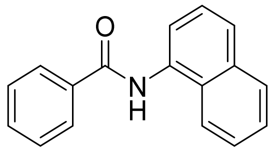

***N*-(Naphthalen-1-yl)benzamide (4am)**[24]**:** Following the general procedure III, using the 1-naphthylamine **3m** (300.0 mg, 2.10 mmol), pyridinium *p*-toluenesulfonate (10.5 mg, 0.04 mmol), *N*,*N*-dimethylformamide (0.243 mL, 3.14 mmol), hexamethyldisilazane (0.667 mL, 3.14 mmol), thiobenzoic acid **1a** (0.742 mL, 6.29 mmol) and 1,8-Diazabicyclo[5.4.0]undec-7-ene (0.470 mL, 3.14 mmol). The crude product was then purified by column chromatography (Hexane:EtOAc = 5:1) to afford compound **4am** (345.0 mg, 67% yield) as a pale-purple solid. ^{1}H NMR (400 MHz, $(CD_3)_2SO$) δ 10.44 (s, 1H), 8.11 (d, *J* = 7.3 Hz, 2H), 8.03 – 7.95 (m, 2H), 7.88 (d, *J* = 8.0 Hz, 1H), 7.66 – 7.51 (m, 8H); ^{13}C{^{1}H} NMR (100 MHz, $CDCl_3$) δ 166.6, 134.8, 134.2, 132.5, 131.9, 128.82 (CH×2), 128.78, 127.8, 127.3 (CH×2), 126.4, 126.3, 126.1, 125.8, 121.7, 121.1.

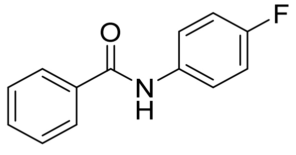

***N*-(4-Fluorophenyl)benzamide (4an)**[24]**:** Following the general procedure III, using the 4-fluoroaniline **3n** (300.0 mg, 2.70 mmol), pyridinium *p*-toluenesulfonate (13.6 mg, 0.05 mmol), *N*,*N*-dimethylformamide (0.314 mL, 4.05 mmol), hexamethyldisilazane (0.860 mL, 4.05 mmol), thiobenzoic acid **1a** (0.957 mL, 8.10 mmol) and 1,8-Diazabicyclo[5.4.0]undec-7-ene (0.605 mL, 4.05 mmol). The crude product was then purified by column chromatography (Hexane:EtOAc = 5:1) to afford compound **4an** (497.0 mg, 85% yield) as a white solid. ^{1}H NMR (400 MHz, $(CD_3)_2SO$) δ 10.32 (s, 1H), 7.97 (d, *J* = 6.6 Hz, 2H), 7.91–7.71 (m, 2H), 7.65–7.47 (m, 2H), 7.19 (t, *J* = 8.1 Hz, 2H); ^{13}C{^{1}H} NMR (100 MHz, $(CD_3)_2SO$) δ 165.5, 159.5 (d, J_{C-F} = 240.3 Hz), 135.6, 134.9, 131.6, 128.4 (CH×2), 127.6 (CH×2), 122.2 (d, J_{C-F} = 7.8 Hz), 115.3 (d, J_{C-F} = 22.2 Hz); ^{19}F NMR (376 MHz, $CDCl_3$) δ -118.8 (s, C-F).

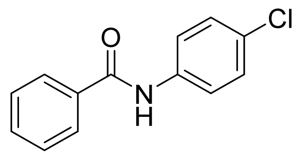

***N*-(4-Chlorophenyl)benzamide (4ao)**[18]**:** Following the general procedure III, using the 4-chloroaniline **3o** (300.0 mg, 2.35 mmol), pyridinium *p*-toluenesulfonate (11.8 mg, 0.05 mmol), *N*,*N*-dimethylformamide (0.273 mL, 3.53 mmol), hexamethyldisilazane (0.749 mL, 3.53 mmol), thiobenzoic acid **1a** (0.833 mL, 7.05 mmol) and 1,8-Diazabicyclo[5.4.0]undec-7-ene (0.527 mL, 3.53 mmol). The crude product was then purified by column chromatography (Hexane:EtOAc = 5:1) to afford compound **4ao** (445.0 mg, 82% yield) as a yellow solid. ^{1}H NMR (400 MHz, $CDCl_3$) δ 7.86 (d, *J* = 7.2 Hz, 2H), 7.80 (s, 1H), 7.60 (d, *J* = 8.8 Hz, 2H), 7.56 (d, *J* = 7.3 Hz, 1H), 7.50 (t, *J* = 7.4 Hz, 2H), 7.34 (d, *J* = 8.8 Hz, 2H); $^{13}C\{^1H\}$ NMR (100 MHz, $(CD_3)_2SO$) δ 165.7, 138.2, 134.7, 131.7, 128.5 (CH×2), 128.4 (CH×2), 127.7 (CH×2), 127.3, 121.8 (CH×2).

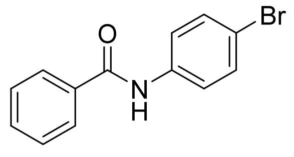

***N*-(4-Bromophenyl)benzamide (4ap)**[24]**:** Following the general procedure III, using the 4-bromoaniline **3p** (300.0 mg, 1.74 mmol), pyridinium *p*-toluenesulfonate (8.8 mg, 0.03 mmol), *N*,*N*-dimethylformamide (0.203 mL, 2.62 mmol), hexamethyldisilazane (0.556 mL, 2.62 mmol), thiobenzoic acid **1a** (0.618 mL, 5.23 mmol) and 1,8-Diazabicyclo[5.4.0]undec-7-ene (0.391 mL, 2.62 mmol). The crude product was then purified by column chromatography (Hexane:EtOAc = 5:1) to afford compound **4ap** (421.0 mg, 87% yield) as a yellow solid. ^{1}H NMR (400 MHz, $(CD_3)_2SO$) δ 10.37 (s, 1H), 7.96 (d, *J* = 7.1 Hz, 2H), 7.79 (d, *J* = 8.8 Hz, 2H), 7.66–7.47 (m, 5H); $^{13}C\{^1H\}$ NMR (100 MHz, $(CD_3)_2SO$) δ 165.7, 138.6, 134.7, 131.7, 131.4 (CH×2), 128.4 (CH×2), 127.7 (CH×2), 122.2 (CH×2), 115.3.

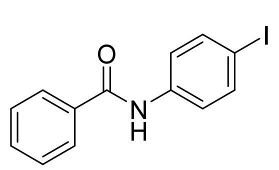

***N*-(4-Iodophenyl)benzamide (4aq)**[26] Following the general procedure III, using the 4-iodoaniline **3q** (300.0 mg, 1.37 mmol), pyridinium *p*-toluenesulfonate (6.9 mg, 0.03 mmol), *N*,*N*-dimethylformamide (0.159 mL, 2.05 mmol), hexamethyldisilazane (0.436

mL, 2.05 mmol), thiobenzoic acid **1a** (0.485 mL, 4.11 mmol) and 1,8-Diazabicyclo[5.4.0]undec-7-ene (0.307 mL, 2.05 mmol). The crude product was then purified by column chromatography (Hexane:EtOAc = 5:1) to afford compound **4aq** (255.0 mg, 58% yield) as a white solid. ^{1}H NMR (400 MHz, $(CD_3)_2SO$) δ 10.36 (s, 1H), 7.94 (d, *J* = 7.3 Hz, 2H), 7.72–7.50 (m, 7H); ^{13}C{^{1}H} NMR (100 MHz, $(CD_3)_2SO$) δ 165.6, 139.1, 137.3 (CH×2), 134.7, 131.7, 128.4 (CH×2), 127.7 (CH×2), 122. 5 (CH×2), 87.3.

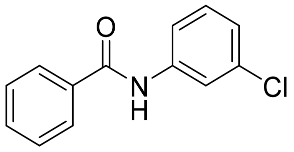

***N*-(3-Chlorophenyl)benzamide (4ar)**[18]**:** Following the general procedure III, using the 3-chloroaniline **3r** (300.0 mg, 2.35 mmol), pyridinium *p*-toluenesulfonate (11.8 mg, 0.05 mmol), *N*,*N*-dimethylformamide (0.273 mL, 3.53 mmol), hexamethyldisilazane (0.749 mL, 3.53 mmol), thiobenzoic acid **1a** (0.833 mL, 7.05 mmol) and 1,8-Diazabicyclo[5.4.0]undec-7-ene (0.527 mL, 3.53 mmol). The crude product was then purified by column chromatography (Hexane:EtOAc = 5:1) to afford compound **4ar** (420.0 mg, 77% yield) as a white solid. ^{1}H NMR (400 MHz, $CDCl_3$) δ 8.32 (s, 1H), 7.81 (d, *J* = 7.3 Hz, 2H), 7.75 (s, 1H), 7.53–7.45 (m, 2H), 7.39 (t, *J* = 7.6 Hz, 2H), 7.21 (t, *J* = 8.1 Hz, 1H), 7.09 (dd, *J* = 8.0, 1.0 Hz, 1H); ^{13}C{^{1}H} NMR (100 MHz, $CDCl_3$) δ 166.3, 139.2, 134.7, 134.5, 132.1, 130.0, 128.8 (CH×2), 127.2 (CH×2), 124.7, 120.7, 118.6.

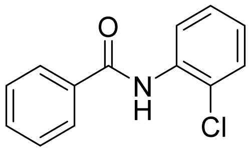

***N*-(2-Chlorophenyl)benzamide (4as)**[18]**:** Following the general procedure III, using the 2-chloroaniline **3s** (300.0 mg, 2.35 mmol), pyridinium *p*-toluenesulfonate (11.8 mg, 0.05 mmol), *N*,*N*-dimethylformamide (0.273 mL, 3.53 mmol), hexamethyldisilazane (0.749 mL, 3.53 mmol), thiobenzoic acid **1a** (0.833 mL, 7.05 mmol) and 1,8-Diazabicyclo[5.4.0]undec-7-ene (0.527 mL, 3.53 mmol). The crude product was then purified by column chromatography (Hexane:EtOAc = 5:1) to afford compound **4as** (401.0 mg, 74% yield) as a white solid. ^{1}H NMR (400 MHz, $CDCl_3$) δ 8.56 (dd, *J* = 8.3, 1.5 Hz, 1H), 8.47 (s, 1H), 7.95–7.79 (m, 2H), 7.57 (t, *J* = 7.3 Hz, 1H), 7.54–7.47 (m, 2H), 7.40 (dd, *J* = 8.0, 1.5 Hz, 1H), 7.35–7.29 (m, 1H), 7.11–7.04 (m, 1H); ^{13}C{^{1}H}

NMR (100 MHz, $CDCl_3$) δ 165.4, 134.8, 134.7, 132.3, 129.13, 129.05 (CH×2), 128.0, 127.2 (CH×2), 124.9, 123.2, 121.6.

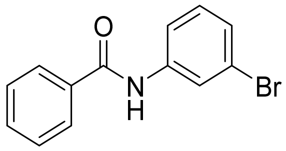

***N*-(3-Bromophenyl)benzamide (4at)**[27]**:** Following the general procedure III, using the 3-bromoaniline **3t** (300.0 mg, 1.74 mmol), pyridinium *p*-toluenesulfonate (8.8 mg, 0.03 mmol), *N*,*N*-dimethylformamide (0.203 mL, 2.62 mmol), hexamethyldisilazane (0.556 mL, 2.62 mmol), thiobenzoic acid **1a** (0.618 mL, 5.23 mmol) and 1,8-Diazabicyclo[5.4.0]undec-7-ene (0.391 mL, 2.62 mmol). The crude product was then purified by column chromatography (Hexane:EtOAc = 5:1) to afford compound **4at** (410.0 mg, 85% yield) as a white solid. ^{1}H NMR (400 MHz, $CDCl_3$) δ 8.30 (s, 1H), 7.89 (s, 1H), 7.81 (d, J = 7.5 Hz, 2H), 7.57–7.47 (m, 2H), 7.40 (t, J = 7.6 Hz, 2H), 7.24 (d, J = 8.0 Hz, 1H), 7.16 (t, J = 8.0 Hz, 1H); ^{13}C{^{1}H} NMR (100 MHz, $CDCl_3$) δ 166.2, 139.3, 134.5, 132.1, 130.4, 128.8 (CH×2), 127.6, 127.2 (CH×2), 123.5, 122.7, 119.0.

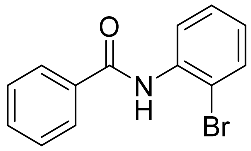

***N*-(2-Bromophenyl)benzamide (4au)**[18]**:** Following the general procedure III, using the 2-bromoaniline **3u** (300.0 mg, 1.74 mmol), pyridinium *p*-toluenesulfonate (8.8 mg, 0.03 mmol), *N*,*N*-dimethylformamide (0.203 mL, 2.62 mmol), hexamethyldisilazane (0.556 mL, 2.62 mmol), thiobenzoic acid **1a** (0.618 mL, 5.23 mmol) and 1,8-Diazabicyclo[5.4.0]undec-7-ene (0.391 mL, 2.62 mmol). The crude product was then purified by column chromatography (Hexane:EtOAc = 5:1) to afford compound **4au** (264.0 mg, 55% yield) as a yellow solid. ^{1}H NMR (400 MHz, $CDCl_3$) δ 8.55 (dd, J = 8.3, 1.5 Hz, 1H), 8.48 (s, 1H), 7.98–7.91 (m, 2H), 7.62–7.54 (m, 2H), 7.51 (t, J = 7.3 Hz, 2H), 7.41–7.32 (m, 1H), 7.04–6.99 (m, 1H); ^{13}C{^{1}H} NMR (100 MHz, $CDCl_3$) δ 165.3, 135.9, 134.6, 132.32, 132.26, 129.0 (CH×2), 128.6, 127.2 (CH×2), 125.4, 121.9, 113.9.

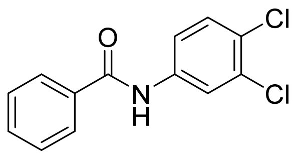

***N*-(3,4-Dichlorophenyl)benzamide (4aw)[24]:** Following the general procedure III, using the 3,4-dichloroaniline **3w** (300.0 mg, 1.85 mmol), pyridinium *p*-toluenesulfonate (9.3 mg, 0.04 mmol), *N*,*N*-dimethylformamide (0.215 mL, 2.78 mmol), hexamethyldisilazane (0.590 mL, 2.78 mmol), thiobenzoic acid **1a** (0.656 mL, 5.55 mmol) and 1,8-Diazabicyclo[5.4.0]undec-7-ene (0.415 mL, 2.78 mmol). The crude product was then purified by column chromatography (Hexane:EtOAc = 5:1) to afford compound **4aw** (397.0 mg, 81% yield) as a white solid. ^{1}H NMR (400 MHz, $CDCl_3$) δ 8.06 (s, 1H), 7.87 (s, 1H), 7.82 (d, *J* = 7.7 Hz, 2H), 7.55 (t, *J* = 7.2 Hz, 1H), 7.50–7.41 (m, 3H), 7.37 (d, *J* = 8.6 Hz, 1H); ^{13}C{^{1}H} NMR (100 MHz, $CDCl_3$) δ 166.0, 137.5, 134.3, 133.0, 132.4, 130.7, 129.0 (CH×2), 127.9, 127.2 (CH×2), 122.1, 119.7.

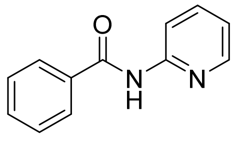

***N*-(Pyridin-2-yl)benzamide (4ax)[28]:** Following the general procedure III, using the 2-aminopyridine **3x** (300.0 mg, 3.19 mmol), pyridinium *p*-toluenesulfonate (16.0 mg, 0.06 mmol), *N*,*N*-dimethylformamide (0.370 mL, 4.78 mmol), hexamethyldisilazane (1.015 mL, 4.78 mmol), thiobenzoic acid **1a** (1.130 mL, 9.56 mmol) and 1,8-Diazabicyclo[5.4.0]undec-7-ene (0.715 mL, 4.78 mmol). The crude product was then purified by column chromatography (Hexane:EtOAc = 5:1) to afford compound **4ax** (426.0 mg, 67% yield) as a yellow liquid. ^{1}H NMR (400 MHz, $CDCl_3$) δ 9.53 (s, 1H), 8.40 (d, *J* = 8.4 Hz, 1H), 8.02 (dd, *J* = 4.9, 0.8 Hz, 1H), 7.91 (d, *J* = 7.7 Hz, 2H), 7.70 (td, *J* = 8.3, 1.7 Hz, 1H), 7.51 (t, *J* = 7.4 Hz, 1H), 7.42 (t, *J* = 7.6 Hz, 2H), 6.99–6.92 (m, 1H); ^{13}C{^{1}H} NMR (100 MHz, $CDCl_3$) δ 166.3, 151.9, 147.6, 138.4, 134.4, 132.0, 128.6 (CH×2), 127.4 (CH×2), 119.7, 114.5.

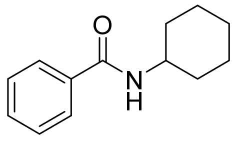

***N*-Cyclohexylbenzamide (4ay)[29]:** Following the general procedure III, using the cyclohexanamine **3y** (300.0 mg, 3.03 mmol), pyridinium *p*-toluenesulfonate (15.2 mg, 0.06 mmol), *N*,*N*-dimethylformamide (0.351 mL, 4.54 mmol), hexamethyldisilazane (0.964 mL, 4.54 mmol), thiobenzoic acid **1a** (1.072 mL, 9.08 mmol) and 1,8-Diazabicyclo[5.4.0]undec-7-ene (0.678 mL, 4.54 mmol). The crude product was then

purified by column chromatography (Hexane:EtOAc = 5:1) to afford compound **4ay** (373.0 mg, 61% yield) as a white solid. ^{1}H NMR (400 MHz, $CDCl_3$) δ 7.74 (d, *J* = 7.8 Hz, 2H), 7.42 (t, *J* = 7.1 Hz, 1H), 7.34 (t, *J* = 7.5 Hz, 2H), 6.39 (s, 1H), 4.02–3.85 (m, 1H), 2.04–1.89 (m, 2H), 1.76–1.65 (m, 2H), 1.64–1.55 (m, 1H), 1.43–1.06 (m, 5H); ^{13}C{^{1}H} NMR (100 MHz, $CDCl_3$) δ 166.7, 135.2, 131.2, 128.5 (CH×2), 127.0 (CH×2), 48.8, 33.2 (CH×2), 25.6, 25.0 (CH×2).

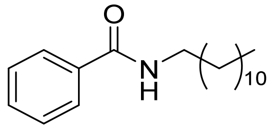

***N*-Dodecylbenzamide (4az)[30]:** Following the general procedure III, using the dodecylamine **3z** (1000.0 mg, 5.39 mmol), pyridinium *p*-toluenesulfonate (27.1 mg, 0.11 mmol), *N*,*N*-dimethylformamide (0.627 mL, 8.09 mmol), hexamethyldisilazane (1.719 mL, 8.09 mmol), thiobenzoic acid **1a** (1.912 mL, 16.18 mmol) and 1,8-Diazabicyclo[5.4.0]undec-7-ene (1.210 mL, 8.09 mmol). The crude product was then purified by column chromatography (Hexane:EtOAc = 5:1) to afford compound **4az** (344.0 mg, 22% yield) as a yellow solid. ^{1}H NMR (400 MHz, $CDCl_3$) δ 7.77 (d, *J* = 7.6 Hz, 2H), 7.42 (t, *J* = 7.3 Hz, 1H), 7.34 (t, *J* = 7.5 Hz, 2H), 6.79 (s, 1H), 3.46–3.27 (m, 2H), 1.62–1.51 (m, 2H), 1.40–1.17 (m, 18H), 0.86 (t, *J* = 6.7 Hz, 3H); ^{13}C{^{1}H} NMR (100 MHz, $CDCl_3$) δ 167.7, 134.9, 131.2, 128.4 (CH×2), 127.0 (CH×2), 40.2, 31.9, 29.69 (CH×2), 29.67, 29.64, 29.61, 29.39, 29.38, 27.1, 22.7, 14.1.

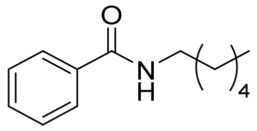

***N*-Hexylbenzamide (4aa')[31]:** Following the general procedure III, using the *n*-hexylamine **3a'** (300.0 mg, 2.96 mmol), pyridinium *p*-toluenesulfonate (14.9 mg, 0.06 mmol), *N*,*N*-dimethylformamide (0.344 mL, 4.45 mmol), hexamethyldisilazane (0.944 mL, 4.45 mmol), thiobenzoic acid **1a** (1.050 mL, 8.89 mmol) and 1,8-Diazabicyclo[5.4.0]undec-7-ene (0.665 mL, 4.45 mmol). The crude product was then purified by column chromatography (Hexane:EtOAc = 5:1) to afford compound **4aa'** (99.0 mg, 16% yield) as a brown liquid. ^{1}H NMR (400 MHz, $CDCl_3$) δ 7.76 (d, *J* = 7.1 Hz, 2H), 7.41 (t, *J* = 7.4 Hz, 1H), 7.32 (t, *J* = 6.7 Hz, 2H), 6.95 (s, 1H), 3.35 (td, *J* = 7.1, 6.0 Hz, 2H), 1.61–1.47 (m, 2H), 1.39–1.15 (m, 6H), 0.84 (m, 3H); ^{13}C{^{1}H} NMR (100 MHz, $CDCl_3$) δ 167.7, 134.9, 131.2, 128.4 (CH×2), 127.0 (CH×2), 40.2, 31.5, 29.6, 26.7, 22.6, 14.0.

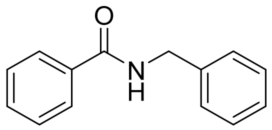

***N*-Benzylbenzamide (4ab')**[29]**:** Following the general procedure III, using the benzylamine **3b'** (300.0 mg, 2.80 mmol), pyridinium *p*-toluenesulfonate (14.1 mg, 0.06 mmol), *N*,*N*-dimethylformamide (0.325 mL, 4.20 mmol), hexamethyldisilazane (0.892 mL, 4.20 mmol), thiobenzoic acid **1a** (0.992 mL, 8.40 mmol) and 1,8-Diazabicyclo[5.4.0]undec-7-ene (0.628 mL, 4.20 mmol). The crude product was then purified by column chromatography (Hexane:EtOAc = 5:1) to afford compound **4ab'** (418.0 mg, 71% yield) as a white solid. ^{1}H NMR (400 MHz, $CDCl_3$) δ 7.80 (d, J = 7.7 Hz, 2H), 7.47 (t, J = 7.4 Hz, 1H), 7.37 (t, J = 7.6 Hz, 2H), 7.33–7.24 (m, 5H), 7.03 (s, 1H), 4.57 (d, J = 5.8 Hz, 2H); ^{13}C{^{1}H} NMR (100 MHz, $CDCl_3$) δ 167.6, 138.4, 134.4, 131.5, 128.7 (CH×2), 128.5 (CH×2), 127.8 (CH×2), 127.5, 127.1 (CH×2), 44.0.

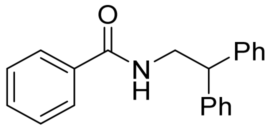

***N*-(2,2-Diphenylethyl)benzamide (4ac')**[32]**:** Following the general procedure III, using the 2,2-diphenylethylamine **3c'** (300.0 mg, 1.52 mmol), pyridinium *p*-toluenesulfonate (7.6 mg, 0.03 mmol), *N*,*N*-dimethylformamide (0.177 mL, 2.28 mmol), hexamethyldisilazane (0.484 mL, 2.28 mmol), thiobenzoic acid **1a** (0.539 mL, 4.56 mmol) and 1,8-Diazabicyclo[5.4.0]undec-7-ene (0.341 mL, 2.28 mmol). The crude product was then purified by column chromatography (Hexane:EtOAc = 5:1) to afford compound **4ac'** (357.0 mg, 78% yield) as a white solid. ^{1}H NMR (400 MHz, $CDCl_3$) δ 7.62 (d, J = 7.6 Hz, 2H), 7.45 (t, J = 7.3 Hz, 1H), 7.38–7.29 (m, 12H), 6.41 (s, 1H), 4.39 (t, J = 7.9 Hz, 1H), 4.11 (m, 2H); ^{13}C{^{1}H} NMR (100 MHz, $CDCl_3$) δ 167.6, 141.9 (CH×2), 134.6, 131.4, 128.8 (CH×4), 128.5 (CH×2), 128.1 (CH×4), 126.9 (CH×4), 50.5, 44.3.

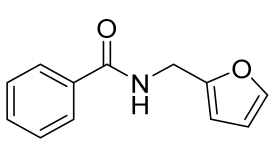

***N*-(Furan-2-ylmethyl)benzamide (4ad')**[33]**:** Following the general procedure III, using the furfurylamine **3d'** (300.0 mg, 3.09 mmol), pyridinium *p*-toluenesulfonate (15.5 mg, 0.06 mmol), *N*,*N*-dimethylformamide (0.359 mL, 4.63 mmol), hexamethyldisilazane (0.984 mL, 4.63 mmol), thiobenzoic acid **1a** (1.095 mL, 9.27 mmol) and 1,8-Diazabicyclo[5.4.0]undec-7-ene (0.693 mL, 4.63 mmol). The crude product was then purified by column chromatography (Hexane:EtOAc = 5:1) to afford compound **4ad'**

(298.3 mg, 48% yield) as a brown solid. ^{1}H NMR (400 MHz, $CDCl_3$) δ 7.78 (d, J = 7.2 Hz, 2H), 7.44 (t, J = 7.4 Hz, 1H), 7.37–7.29 (m, 3H), 7.14 (s, 1H), 6.28 (dd, J = 3.1, 1.9 Hz, 1H), 6.21 (d, J = 2.8 Hz, 1H), 4.56 (d, J = 5.6 Hz, 2H); ^{13}C{^{1}H} NMR (100 MHz, $CDCl_3$) δ 167.57, 151.4, 142.1, 134.1, 131.5, 128.4 (CH×2), 127.1 (CH×2), 110.5, 107.5, 36.9.

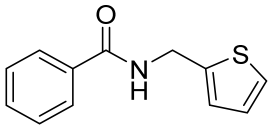

***N*-(Thiophen-2-ylmethyl)benzamide (4ae')[33]:** Following the general procedure III, using the 2-thiophenemethylamine **3e'** (300.0 mg, 2.65 mmol), pyridinium *p*-toluenesulfonate (13.3 mg, 0.05 mmol), *N*,*N*-dimethylformamide (0.308 mL, 3.98 mmol), hexamethyldisilazane (0.844 mL, 3.98 mmol), thiobenzoic acid **1a** (0.939 mL, 7.95 mmol) and 1,8-Diazabicyclo[5.4.0]undec-7-ene (0.594 mL, 3.98 mmol). The crude product was then purified by column chromatography (Hexane:EtOAc = 5:1) to afford compound **4ae'** (297.5 mg, 52% yield) as a pale-brown solid. ^{1}H NMR (400 MHz, $CDCl_3$) δ 7.78 (d, J = 7.2 Hz, 2H), 7.47 (t, J = 7.4 Hz, 1H), 7.38 (t, J = 7.6 Hz, 2H), 7.21 (dd, J = 5.1, 1.1 Hz, 1H), 7.05–6.87 (m, 3H), 4.76 (d, J = 5.7 Hz, 2H); ^{13}C{^{1}H} NMR (100 MHz, $CDCl_3$) δ 167.4, 141.0, 134.2, 131.6, 128.6 (CH×2), 127.1 (CH×2), 127.0, 126.2, 125.3, 38.8.

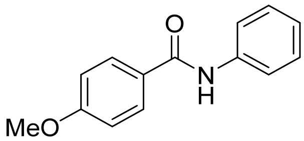

4-Methoxy-*N*-phenylbenzamide (4ba)[27]: Following the general procedure III, using the aniline **3a** (100.0 mg, 1.07 mmol), pyridinium *p*-toluenesulfonate (5.4 mg, 0.02 mmol), *N*,*N*-dimethylformamide (0.125 mL, 1.61 mmol), hexamethyldisilazane (0.342 mL, 1.61 mmol), 4-methoxybenzothioic *S*-acid **1b** (541.9 mg, 3.22 mmol) and 1,8-Diazabicyclo[5.4.0]undec-7-ene (0.241 mL, 1.61 mmol). The crude product was then purified by column chromatography (Hexane:EtOAc = 5:1) to afford compound **4ba** (124.0 mg, 51% yield) as a white solid. ^{1}H NMR (400 MHz, $CDCl_3$) δ 7.84 (d, J = 8.7 Hz, 2H), 7.79 (s, 1H), 7.63 (d, J = 8.3 Hz, 2H), 7.36 (t, J = 7.3 Hz, 2H), 7.14 (t, J = 7.4 Hz, 1H), 6.97 (d, J = 7.5 Hz, 2H), 3.87 (s, 3H); ^{13}C{^{1}H} NMR (100 MHz, $CDCl_3$) δ 165.4, 162.6, 138.3, 129.2 (CH×2), 129.0 (CH×2), 127.3, 124.5, 120.3 (CH×2), 114.1 (CH×2), 55.6.

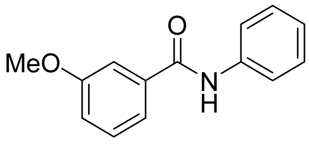

3-Methoxy-*N*-phenylbenzamide (4ca)[34]**:** Following the general procedure III, using the aniline **3a** (100.0 mg, 1.07 mmol), pyridinium *p*-toluenesulfonate (5.4 mg, 0.02 mmol), *N*,*N*-dimethylformamide (0.125 mL, 1.61 mmol), hexamethyldisilazane (0.342 mL, 1.61 mmol), 3-methylbenzothioic *S*-acid **1c** (541.9 mg, 3.22 mmol) and 1,8-Diazabicyclo[5.4.0]undec-7-ene (0.241 mL, 1.61 mmol). The crude product was then purified by column chromatography (Hexane:EtOAc = 5:1) to afford compound **4ca** (119.0 mg, 50% yield) as a white solid. ^{1}H NMR (400 MHz, $CDCl_3$) δ 8.45 (s, 1H), 7.66 (d, J = 7.6 Hz, 2H), 7.42–7.22 (m, 5H), 7.13 (t, J = 7.1 Hz, 1H), 7.01 (d, J = 7.9 Hz, 1H), 3.75 (s, 3H); $^{13}C\{^1H\}$ NMR (100 MHz, $CDCl_3$) δ 166.1, 159.8, 138.1, 136.4, 129.6, 129.0 (CH×2), 124.6, 120.6 (CH×2), 119.0, 118.0, 112.5, 55.3.

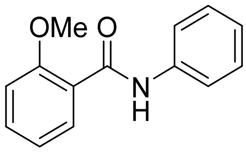

2-Methoxy-*N*-phenylbenzamide (4da)[34]**:** Following the general procedure III, using the aniline **3a** (100.0 mg, 1.07 mmol), pyridinium *p*-toluenesulfonate (5.4 mg, 0.02 mmol), *N*,*N*-dimethylformamide (0.125 mL, 1.61 mmol), hexamethyldisilazane (0.342 mL, 1.61 mmol), 2-methylbenzothioic *S*-acid **1d** (541.9 mg, 3.22 mmol) and 1,8-Diazabicyclo[5.4.0]undec-7-ene (0.241 mL, 1.61 mmol). The crude product was then purified by column chromatography (Hexane:EtOAc = 5:1) to afford compound **4da** (36.2 mg, 15% yield) as orange oil. ^{1}H NMR (400 MHz, $CDCl_3$) δ 9.82 (br s, 1H), 8.29 (dd, J = 7.8, 1.9 Hz, 1H), 7.72–7.64 (m, 2H), 7.58–7.45 (m, 1H), 7.41–7.32 (m, 2H), 7.18–7.09 (m, 2H), 7.03 (d, J = 8.5 Hz, 1H), 4.05 (s, 3H); $^{13}C\{^1H\}$ NMR (100 MHz, $CDCl_3$) δ 163.3, 157.3, 138.5, 133.4, 132.6, 129.1, 124.3, 121.8, 120.5, 111.6, 56.3.

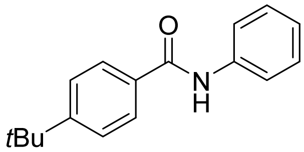

4-(*tert*-Butyl)-*N*-phenylbenzamide (4ea)[35]**:** Following the general procedure III, using the aniline **3a** (100.0 mg, 1.07 mmol), pyridinium *p*-toluenesulfonate (5.4 mg, 0.02 mmol), *N*,*N*-dimethylformamide (0.125 mL, 1.61 mmol), hexamethyldisilazane (0.342 mL, 1.61 mmol), 4-(*tert*-butyl)benzothioic *S*-acid **1e** (626.0 mg, 3.22 mmol) and 1,8-Diazabicyclo[5.4.0]undec-7-ene (0.241 mL, 1.61 mmol). The crude product was then

purified by column chromatography (Hexane:EtOAc = 5:1) to afford compound **4ea** (110.0 mg, 41% yield) as a white solid. ^{1}H NMR (400 MHz, $CDCl_3$) δ 8.18 (s, 1H), 7.80 (d, J = 8.2 Hz, 2H), 7.66 (d, J = 7.9 Hz, 2H), 7.43 (d, J = 8.2 Hz, 2H), 7.33 (t, J = 7.8 Hz, 2H), 7.13 (t, J = 7.4 Hz, 1H), 1.34 (s, 9H); ^{13}C{^{1}H} NMR (100 MHz, $CDCl_3$) δ 166.3, 155.1, 138.3, 132.0, 128.9 (CH×2), 127.2 (CH×2), 125.5 (CH×2), 124.3, 120.7 (CH×2), 34.9, 31.1 (CH×3).

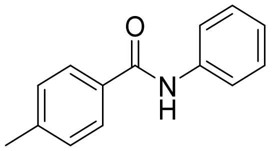

4-Methyl-*N*-phenylbenzamide (4fa)[19b]**:** Following the general procedure III, using the aniline **3a** (100.0 mg, 1.07 mmol), pyridinium *p*-toluenesulfonate (5.4 mg, 0.02 mmol), *N*,*N*-dimethylformamide (0.125 mL, 1.61 mmol), hexamethyldisilazane (0.342 mL, 1.61 mmol), 4-methylbenzothioic *S*-acid **1f** (489.8 mg, 3.22 mmol) and 1,8-Diazabicyclo[5.4.0]undec-7-ene (0.241 mL, 1.61 mmol). The crude product was then purified by column chromatography (Hexane:EtOAc = 5:1) to afford compound 4**fa** (197.0 mg, 87% yield) as a white solid. ^{1}H NMR (400 MHz, $CDCl_3$) δ 7.87 (s, 1H), 7.74 (d, J = 8.0 Hz, 2H), 7.62 (d, J = 8.1 Hz, 2H), 7.34 (t, J = 7.8 Hz, 2H), 7.24 (d, J = 6.9 Hz, 2H), 7.12 (t, J = 7.4 Hz, 1H), 2.40 (s, 3H); ^{13}C{^{1}H} NMR (100 MHz, $CDCl_3$) δ 166.0, 142.3, 138.2, 132.2, 129.4 (CH×2), 129.1 (CH×2), 127.2 (CH×2), 124.5, 120.5 (CH×2), 21.6.

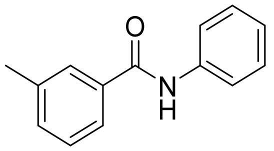

3-Methyl-*N*-phenylbenzamide (4ga)[27]**:** Following the general procedure III, using the aniline **3a** (100.0 mg, 1.07 mmol), pyridinium *p*-toluenesulfonate (5.4 mg, 0.02 mmol), *N*,*N*-dimethylformamide (0.125 mL, 1.61 mmol), hexamethyldisilazane (0.342 mL, 1.61 mmol), 3-methylbenzothioic *S*-acid **1g** (489.8 mg, 3.22 mmol) and 1,8-Diazabicyclo[5.4.0]undec-7-ene (0.241 mL, 1.61 mmol). The crude product was then purified by column chromatography (Hexane:EtOAc = 5:1) to afford compound **4ga** (163.0 mg, 72% yield) as a yellow solid. ^{1}H NMR (400 MHz, $CDCl_3$) δ 8.32 (s, 1H), 7.69–7.64 (m, 3H), 7.62 (d, J = 7.1 Hz, 1H), 7.38–7.26 (m, 4H), 7.13 (t, J = 7.4 Hz, 1H), 2.35 (s, 3H); ^{13}C{^{1}H} NMR (100 MHz, $CDCl_3$) δ 166.4, 138.5, 138.2, 135.0, 132.5, 129.0 (CH×2), 128.5, 128.0, 124.5, 124.2, 120.5 (CH×2), 21.4.

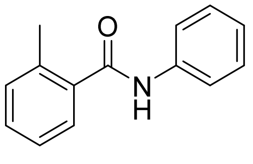

2-Methyl-*N*-phenylbenzamide (4ha)[27]**:** Following the general procedure III, using the aniline **3a** (100.0 mg, 1.07 mmol), pyridinium *p*-toluenesulfonate (5.4 mg, 0.02 mmol), *N*,*N*-dimethylformamide (0.125 mL, 1.61 mmol), hexamethyldisilazane (0.342 mL, 1.61 mmol), 2-methylbenzothioic *S*-acid **1h** (489.8 mg, 3.22 mmol) and 1,8-Diazabicyclo[5.4.0]undec-7-ene (0.241 mL, 1.61 mmol). The crude product was then purified by column chromatography (Hexane:EtOAc = 5:1) to afford compound **4ha** (202.0 mg, 89% yield) as a white solid. ^{1}H NMR (400 MHz, $CDCl_3$) δ 8.06 (s, 1H), 7.60 (d, *J* = 7.5 Hz, 2H), 7.40–7.28 (m, 4H), 7.23–7.11 (m, 3H), 2.42 (s, 3H); ^{13}C{^{1}H} NMR (100 MHz, $CDCl_3$) δ 168.4, 138.2, 136.4, 136.2, 131.1, 130.1, 129.0 (CH×2), 126.7, 125.8, 124.4, 120.1 (CH×2), 19.8.

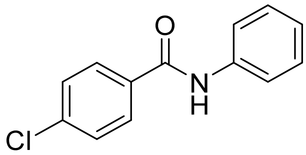

4-Chloro-*N*-phenylbenzamide (4ia)[19a]**:** Following the general procedure III, using the aniline **3a** (100.0 mg, 1.07 mmol), pyridinium *p*-toluenesulfonate (5.4 mg, 0.02 mmol), *N*,*N*-dimethylformamide (0.125 mL, 1.61 mmol), hexamethyldisilazane (0.342 mL, 1.61 mmol), 4-chlorobenzothioic *S*-acid **1i** (556.1 mg, 3.22 mmol) and 1,8-Diazabicyclo[5.4.0]undec-7-ene (0.241 mL, 1.61 mmol). The crude product was then purified by column chromatography (Hexane:EtOAc = 5:1) to afford compound **4ia** (167.0 mg, 67% yield) as a white solid. ^{1}H NMR (400 MHz, $(CD_3)_2SO$) δ 10.28 (s, 1H), 7.95 (d, *J* = 8.5 Hz, 2H), 7.74 (d, *J* = 7.7 Hz, 2H), 7.57 (d, *J* = 8.5 Hz, 2H), 7.32 (t, *J* = 7.9 Hz, 2H), 7.07 (t, *J* = 7.4 Hz, 1H); ^{13}C{^{1}H} NMR (100 MHz, $(CD_3)_2SO$) δ 164.4, 139.0, 136.4, 133.7, 129.6 (CH×2), 128.6 (CH×2), 128.5 (CH×2), 123.8, 120.4 (CH×2).

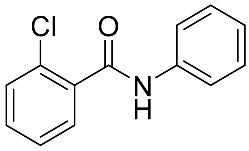

2-Chloro-*N*-phenylbenzamide (4ja)[36]**:** Following the general procedure III, using the aniline **3a** (100.0 mg, 1.07 mmol), pyridinium *p*-toluenesulfonate (5.4 mg, 0.02 mmol), *N*,*N*-dimethylformamide (0.125 mL, 1.61 mmol), hexamethyldisilazane (0.342 mL, 1.61 mmol), 2-chlorobenzothioic *S*-acid **1j** (556.1 mg, 3.22 mmol) and 1,8-

Diazabicyclo[5.4.0]undec-7-ene (0.241 mL, 1.61 mmol). The crude product was then purified by column chromatography (Hexane:EtOAc = 5:1) to afford compound **4ja** (180.0 mg, 72% yield) as a white solid. ^{1}H NMR (400 MHz, $CDCl_3$) δ 8.30 (s, 1H), 7.60 (d, J = 7.9 Hz, 2H), 7.56 (d, J = 7.5 Hz, 1H), 7.38–7.28 (m, 4H), 7.24 (t, J = 7.2 Hz, 1H), 7.13 (t, J = 7.3 Hz, 1H); ^{13}C{^{1}H} NMR (100 MHz, $CDCl_3$) δ 164.9, 137.7, 135.3, 131.4, 130.7, 130.2, 129.8, 129.0 (CH×2), 127.0, 124.7, 120.3 (CH×2).

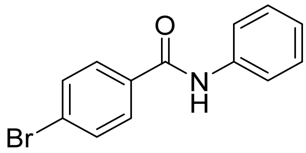

4-Bromo-*N*-phenylbenzamide (4la)[19a]**:** Following the general procedure III, using the aniline **3a** (100.0 mg, 1.07 mmol), pyridinium *p*-toluenesulfonate (5.4 mg, 0.02 mmol), *N*,*N*-dimethylformamide (0.125 mL, 1.61 mmol), hexamethyldisilazane (0.342 mL, 1.61 mmol), 4-bromobenzothioic *S*-acid **1l** (699.3 mg, 3.22 mmol) and 1,8-Diazabicyclo[5.4.0]undec-7-ene (0.241 mL, 1.61 mmol). The crude product was then purified by column chromatography (Hexane:EtOAc = 5:1) to afford compound **4la** (260.4 mg, 88% yield) as a yellow solid. ^{1}H NMR (400 MHz, $(CD_3)_2SO$) δ 10.31 (s, 1H), 7.92 (d, J = 8.2 Hz, 2H), 7.78 (d, J = 8.3 Hz, 2H), 7.74 (d, J = 8.1 Hz, 2H), 7.36 (t, J = 7.6 Hz, 2H), 7.11 (t, J = 7.2 Hz, 1H); ^{13}C{^{1}H} NMR (100 MHz, $(CD_3)_2SO$) δ 164.5, 139.0, 134.0, 131.4 (CH×2), 129.8 (CH×2), 128.6 (CH×2), 125.3, 123.8, 120.4 (CH×2).

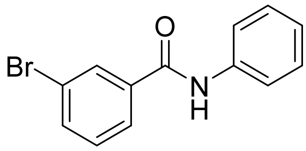

3-Bromo-*N*-phenylbenzamide (4ma)[37]**:** Following the general procedure III, using the aniline **3a** (100.0 mg, 1.07 mmol), pyridinium *p*-toluenesulfonate (5.4 mg, 0.02 mmol), *N*,*N*-dimethylformamide (0.125 mL, 1.61 mmol), hexamethyldisilazane (0.342 mL, 1.61 mmol), 3-bromobenzothioic *S*-acid **1m** (699.3 mg, 3.22 mmol) and 1,8-Diazabicyclo[5.4.0]undec-7-ene (0.241 mL, 1.61 mmol). The crude product was then purified by column chromatography (Hexane:EtOAc = 5:1) to afford compound **4ma** (170.0 mg, 57% yield) as a white solid. ^{1}H NMR (400 MHz, $CDCl_3$) δ 8.07 (s, 1H), 7.97 (s, 1H), 7.75 (d, J = 6.8 Hz, 1H), 7.70–7.57 (m, 3H), 7.42–7.27 (m, 3H), 7.15 (t, J = 6.9 Hz, 1H); ^{13}C{^{1}H} NMR (100 MHz, $CDCl_3$) δ 164.6, 137.7, 137.0, 134.8, 130.41, 130.37, 129.2 (CH×2), 125.8, 125.0, 123.0, 120.6 (CH×2).

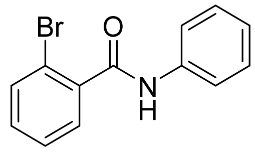

2-Bromo-*N*-phenylbenzamide (4na)[27]**:** Following the general procedure III, using the aniline **3a** (100.0 mg, 1.07 mmol), pyridinium *p*-toluenesulfonate (5.4 mg, 0.02 mmol), *N*,*N*-dimethylformamide (0.125 mL, 1.61 mmol), hexamethyldisilazane (0.342 mL, 1.61 mmol), 2-bromobenzothioic *S*-acid **1n** (699.3 mg, 3.22 mmol) and 1,8-Diazabicyclo[5.4.0]undec-7-ene (0.241 mL, 1.61 mmol). The crude product was then purified by column chromatography (Hexane:EtOAc = 5:1) to afford compound **4na** (130.0 mg, 44% yield) as a white solid. ^{1}H NMR (400 MHz, $CDCl_3$) δ 8.09 (s, 1H), 7.61 (d, J = 7.6 Hz, 2H), 7.56 (dd, J = 7.9, 1.1 Hz, 1H), 7.51 (dd, J = 7.5, 1.8 Hz, 1H), 7.38–7.28 (m, 3H), 7.25 (td, J = 7.7, 1.8 Hz, 1H), 7.15 (t, J = 7.4 Hz, 1H); ^{13}C{^{1}H} NMR (100 MHz, $CDCl_3$) δ 165.8, 137.8, 137.7, 133.5, 131.5, 129.6 (CH×2), 129.1, 127.6, 124.8, 120.2 (CH×2), 119.4.

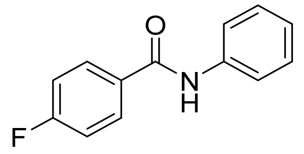

4-Fluoro-*N*-phenylbenzamide (4oa)[19a]**:** Following the general procedure III, using the aniline **3a** (100.0 mg, 1.07 mmol), pyridinium *p*-toluenesulfonate (5.4 mg, 0.02 mmol), *N*,*N*-dimethylformamide (0.125 mL, 1.61 mmol), hexamethyldisilazane (0.342 mL, 1.61 mmol), 4-fluorobenzothioic *S*-acid **1o** (503.1 mg, 3.22 mmol) and 1,8-Diazabicyclo[5.4.0]undec-7-ene (0.241 mL, 1.61 mmol). The crude product was then purified by column chromatography (Hexane:EtOAc = 5:1) to afford compound **4oa** (87.4 mg, 38% yield) as a white solid. ^{1}H NMR (400 MHz, $(CD_3)_2SO$) δ 10.26 (s, 1H), 8.05 (dd, J = 8.3, 5.7 Hz, 2H), 7.78 (d, J = 8.0 Hz, 2H), 7.41–7.31 (m, 4H), 7.10 (t, J = 7.3 Hz, 1H); ^{13}C{^{1}H} NMR (100 MHz, $(CD_3)_2SO$) δ 165.3 (d, $J_{C\text{-}F}$ = 248.6 Hz), 164.4, 139.1, 131.4, 130.4 (d, $J_{C\text{-}F}$ = 8.8 Hz, 2C), 128.6 (CH×2), 123.7, 120.4 (CH×2), 115.4 (d, $J_{C\text{-}F}$ = 21.7 Hz, 2C); ^{19}F NMR (376 MHz, $CDCl_3$) δ -108.7 (s, C-F).

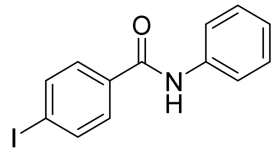

4-Iodo-*N*-phenylbenzamide (4pa)[19a]**:** Following the general procedure III, using the aniline **3a** (100.0 mg, 1.07 mmol), pyridinium *p*-toluenesulfonate (5.4 mg, 0.02 mmol),

N,*N*-dimethylformamide (0.125 mL, 1.61 mmol), hexamethyldisilazane (0.342 mL, 1.61 mmol), 4-iodobenzothioic *S*-acid **1p** (850.7 mg, 3.22 mmol) and 1,8-Diazabicyclo[5.4.0]undec-7-ene (0.241 mL, 1.61 mmol). The crude product was then purified by column chromatography (Hexane:EtOAc = 5:1) to afford compound **4pa** (251.2 mg, 72% yield) as a yellow solid. ^{1}H NMR (400 MHz, $(CD_3)_2SO$) δ 10.28 (s, 1H), 7.91 (d, J = 8.5 Hz, 2H), 7.80–7.72 (m, 4H), 7.35 (t, J = 7.9 Hz, 2H), 7.10 (t, J = 7.4 Hz, 1H); ^{13}C{^{1}H} NMR (100 MHz, $(CD_3)_2SO$) δ 164.8, 139.0, 137.2 (CH×2), 134.3, 129.6 (CH×2), 128.6 (CH×2), 123.8, 120.4 (CH×2), 99.2.

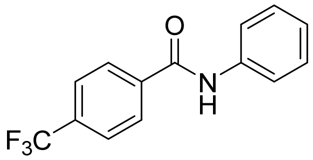

***N*-Phenyl-4-(trifluoromethyl)benzamide (4qa)**[34]**:** Following the general procedure III, using the aniline **3a** (100.0 mg, 1.07 mmol), pyridinium *p*-toluenesulfonate (5.4 mg, 0.02 mmol), *N*,*N*-dimethylformamide (0.125 mL, 1.61 mmol), hexamethyldisilazane (0.342 mL, 1.61 mmol), 4-(trifluoromethyl)benzothioic *S*-acid **1q** (664.2 mg, 3.22 mmol) and 1,8-Diazabicyclo[5.4.0]undec-7-ene (0.241 mL, 1.61 mmol). The crude product was then purified by column chromatography (Hexane:EtOAc = 5:1) to afford compound **4qa** (153.0 mg, 54% yield) as a white solid. ^{1}H NMR (400 MHz, $(CD_3)_2SO$) δ 10.47 (s, 1H), 8.16 (d, J = 8.1 Hz, 2H), 7.90 (d, J = 8.1 Hz, 2H), 7.80 (d, J = 8.2 Hz, 2H), 7.37 (t, J = 7.7 Hz, 2H), 7.13 (t, J = 7.3 Hz, 1H); ^{13}C{^{1}H} NMR (100 MHz, $(CD_3)_2SO$) δ 164.4, 138.9, 138.8, 131.41 (q, J_{C-F} = 31.9 Hz), 128.7 (CH×2), 128.6 (CH×2), 125.36 (q, J_{C-F} = 4.0 Hz), 124.0, 124.0 (q, J_{C-F} = 271 Hz), 120.5 (CH×2); ^{19}F NMR (376 MHz, $(CD_3)_2SO$) δ -61.3 (s, C-F).

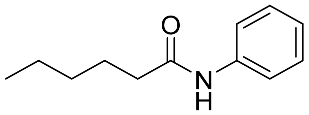

***N*-Phenylhexanamide (4ra)**[19a]**:** Following the general procedure III, using the aniline **3a** (100.0 mg, 1.07 mmol), pyridinium *p*-toluenesulfonate (5.4 mg, 0.02 mmol), *N*,*N*-dimethylformamide (0.125 mL, 1.61 mmol), hexamethyldisilazane (0.342 mL, 1.61 mmol), hexanethioic *S*-acid **1r** (425.9 mg, 3.22 mmol) and 1,8-Diazabicyclo[5.4.0]undec-7-ene (0.241 mL, 1.61 mmol). The crude product was then purified by column chromatography (Hexane:EtOAc = 5:1) to afford compound **4ra** (170.0 mg, 83% yield) as a white solid. ^{1}H NMR (400 MHz, $CDCl_3$) δ 8.56 (s, 1H), 7.63 (d, J = 8.0 Hz, 2H), 7.32 (t, J = 7.8 Hz, 2H), 7.13 (t, J = 7.4 Hz, 1H), 2.40 (t, J = 7.6 Hz, 2H), 1.76 (quin., J = 7.2 Hz, 2H), 1.43–1.29 (m, 4H), 0.94 (t, J = 6.9 Hz, 3H);

$^{13}C\{^1H\}$ NMR (100 MHz, $CDCl_3$) δ 172.5, 138.3, 128.8 (CH×2), 124.1, 120.3 (CH×2), 37.5, 31.4, 25.5, 22.4, 13.9.

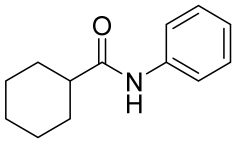

***N*-Phenylcyclohexanecarboxamide (4ta)[27]:** Following the general procedure III, using the aniline **3a** (100.0 mg, 1.07 mmol), pyridinium *p*-toluenesulfonate (5.4 mg, 0.02 mmol), *N*,*N*-dimethylformamide (0.125 mL, 1.61 mmol), hexamethyldisilazane (0.342 mL, 1.61 mmol), cyclohexanecarbothioic *S*-acid **1t** (464.6 mg, 3.22 mmol) and 1,8-Diazabicyclo[5.4.0]undec-7-ene (0.241 mL, 1.61 mmol). The crude product was then purified by column chromatography (Hexane:EtOAc = 5:1) to afford compound **4ta** (177.0 mg, 81% yield) as a white solid. ^1H NMR (400 MHz, $CDCl_3$) δ 7.71 (s, 1H), 7.55 (d, *J* = 7.8 Hz, 2H), 7.28 (t, *J* = 7.9 Hz, 2H), 7.07 (t, *J* = 7.4 Hz, 1H), 2.25 (tt, *J* = 11.8, 3.4 Hz, 1H), 1.92 (d, *J* = 13.4 Hz, 2H), 1.87–1.76 (m, 2H), 1.76–1.63 (m, 1H), 1.63–1.44 (m, 2H), 1.37–1.15 (m, 3H); $^{13}C\{^1H\}$ NMR (100 MHz, $CDCl_3$) δ 174.9, 138.3, 129.0 (CH×2), 124.1, 120.0 (CH×2), 46.5, 29.7 (CH×2), 25.7 (CH×3).

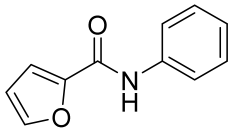

***N*-Phenylfuran-2-carboxamide (4ua)[19a]:** Following the general procedure III, using the aniline **3a** (100.0 mg, 1.07 mmol), pyridinium *p*-toluenesulfonate (5.4 mg, 0.02 mmol), *N*,*N*-dimethylformamide (0.125 mL, 1.61 mmol), hexamethyldisilazane (0.342 mL, 1.61 mmol), furan-2-carbothioic *S*-acid **1u** (412.8 mg, 3.22 mmol) and 1,8-Diazabicyclo[5.4.0]undec-7-ene (0.241 mL, 1.61 mmol). The crude product was then purified by column chromatography (Hexane:EtOAc = 5:1) to afford compound **4ua** (170.0 mg, 84% yield) as a pale-brown solid. ^1H NMR (400 MHz, $CDCl_3$) δ 8.27 (s, 1H), 7.67 (d, *J* = 8.0 Hz, 2H), 7.45 (s, 1H), 7.33 (t, *J* = 7.9 Hz, 2H), 7.20 (d, *J* = 3.5 Hz, 1H), 7.12 (t, *J* = 7.4 Hz, 1H), 6.55–6.47 (m, 1H); $^{13}C\{^1H\}$ NMR (100 MHz, $CDCl_3$) δ 156.3, 147.8, 144.3, 137.5, 129.1, 124.5, 120.1 (CH×2), 115.2, 112.6 (CH×2).

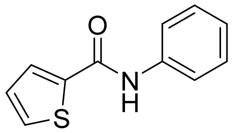

***N*-Phenylthiophene-2-carboxamide (4va)[19a]:** Following the general procedure III, using the aniline **3a** (100.0 mg, 1.07 mmol), pyridinium *p*-toluenesulfonate (5.4 mg, 0.02 mmol), *N*,*N*-dimethylformamide (0.125 mL, 1.61 mmol), hexamethyldisilazane

(0.342 mL, 1.61 mmol), thiophene-2-carbothioic *S*-acid **1v** (464.6 mg, 3.22 mmol) and 1,8-Diazabicyclo[5.4.0]undec-7-ene (0.241 mL, 1.61 mmol). The crude product was then purified by column chromatography (Hexane:EtOAc = 5:1) to afford compound **4va** (119.0 mg, 54% yield) as a brown solid. ^{1}H NMR (400 MHz, $CDCl_3$) δ 7.86 (s, 1H), 7.67–7.57 (m, 3H), 7.53 (d, *J* = 4.8 Hz, 1H), 7.34 (t, *J* = 7.2 Hz, 2H), 7.18–7.06 (m, 2H); ^{13}C{^{1}H} NMR (100 MHz, $CDCl_3$) δ 160.2, 139.4, 137.7, 130.9, 129.2 (CH×2), 128.6, 128.0, 124.8, 120.4 (CH×2).

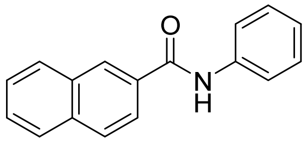

***N*-Phenyl-2-naphthamide (4wa)**[27]**:** Following the general procedure III, using the aniline **3a** (100.0 mg, 1.07 mmol), pyridinium *p*-toluenesulfonate (5.4 mg, 0.02 mmol), *N*,*N*-dimethylformamide (0.125 mL, 1.61 mmol), hexamethyldisilazane (0.342 mL, 1.61 mmol), naphthalene-2-carbothioic *S*-acid **1w** (616.4 mg, 3.22 mmol) and 1,8-Diazabicyclo[5.4.0]undec-7-ene (0.241 mL, 1.61 mmol). The crude product was then purified by column chromatography (Hexane:EtOAc = 5:1) to afford compound **4wa** (176.0 mg, 66% yield) as a orange solid. ^{1}H NMR (400 MHz, $CDCl_3$) δ 8.39 (s, 1H), 8.01–7.93 (m, 4H), 7.91 (d, *J* = 7.5 Hz, 1H), 7.70 (d, *J* = 8.1 Hz, 2H), 7.64–7.54 (m, 2H), 7.41 (t, *J* = 7.7 Hz, 2H), 7.18 (t, *J* = 7.4 Hz, 1H); ^{13}C{^{1}H} NMR (100 MHz, $CDCl_3$) δ 166.0, 138.1, 135.0, 132.7, 132.3, 129.3 (CH×2), 129.1, 128.9, 128.0, 127.9, 127.7, 127.1, 124.7, 123.7, 120.4 (CH×2).

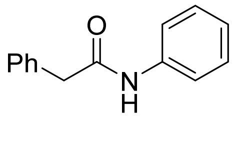

***N*,2-Diphenylacetamide (4a'a)**[34]**:** Following the general procedure III, using the aniline **3a** (100.0 mg, 1.07 mmol), pyridinium *p*-toluenesulfonate (5.4 mg, 0.02 mmol), *N*,*N*-dimethylformamide (0.125 mL, 1.61 mmol), hexamethyldisilazane (0.342 mL, 1.61 mmol), 2-phenylethanethioic thioacid **1a'** (490.3 mg, 3.22 mmol) and 1,8-Diazabicyclo[5.4.0]undec-7-ene (0.241 mL, 1.61 mmol). The crude product was then purified by column chromatography (Hexane:EtOAc = 5:1) to afford compound **4a'a** (121.0 mg, 53% yield) as a white solid. ^{1}H NMR (400 MHz, $CDCl_3$) δ 7.66 (s, 1H), 7.43 (d, *J* = 8.0 Hz, 2H), 7.39–7.20 (m, 7H), 7.07 (t, *J* = 7.4 Hz, 1H), 3.67 (s, 2H); ^{13}C{^{1}H} NMR (100 MHz, $CDCl_3$) δ 169.6, 137.8, 134.6, 129.5 (CH×2), 129.2 (CH×2), 129.0 (CH×2), 127.6, 124.5, 120.1 (CH×2), 44.7.

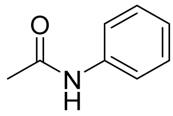

***N*-Phenylacetamide (4b'a)**[27]**:** Following the general procedure III, using the aniline **3a** (100.0 mg, 1.07 mmol), pyridinium *p*-toluenesulfonate (5.4 mg, 0.02 mmol), *N*,*N*-dimethylformamide (0.125 mL, 1.61 mmol), hexamethyldisilazane (0.342 mL, 1.61 mmol), thioacetic acid **1b'** (245.2 mg, 3.22 mmol) and 1,8-Diazabicyclo[5.4.0]undec-7-ene (0.241 mL, 1.61 mmol). The crude product was then purified by column chromatography (Hexane:EtOAc = 5:1) to afford compound **4b'a** (127.0 mg, 87% yield) as an orange solid. ^{1}H NMR (400 MHz, $CDCl_3$) δ 7.97 (s, 1H), 7.52 (d, J = 8.2 Hz, 2H), 7.30 (t, J = 7.8 Hz, 2H), 7.10 (t, J = 7.3 Hz, 1H), 2.16 (s, 3H); ^{13}C{^{1}H} NMR (100 MHz, $CDCl_3$) δ 169.0, 138.1, 129.0 (CH×2), 124.4, 120.2 (CH×2), 24.5.

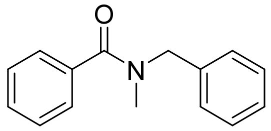

***N*-Benzyl-*N*-methylbenzamide (4af')**[26]**:** Following the general procedure III, using the *n*-benzylmethylamine **3f'** (300.0 mg, 2.48 mmol), pyridinium *p*-toluenesulfonate (12.4 mg, 0.05 mmol), *N*,*N*-dimethylformamide (0.288 mL, 3.71 mmol), hexamethyldisilazane (0.789 mL, 3.71 mmol), thiobenzoic acid **1a** (0.877 mL, 7.43 mmol) and 1,8-Diazabicyclo[5.4.0]undec-7-ene (0.555 mL, 3.71 mmol). The crude product was then purified by column chromatography (Hexane:EtOAc = 5:1) to afford compound **4af'** (416.0 mg, 75% yield) as an orange liquid. ^{1}H NMR (400 MHz, $CDCl_3$) δ 7.33–7.00 (m, 9H), 6.95 (s, 1H), 4.56 (s, 1H), 4.27 (s, 1H), 2.80 (s, 1.5H), 2.60 (s, 1.5H); ^{13}C{^{1}H} NMR (100 MHz, $CDCl_3$) δ 172.2, 171.5, 136.9, 136.5, 136.1 (CH×2), 129.5, 128.7, 128.6, 128.3 (CH×2), 128.1, 127.4 (CH×3), 126.9, 126.7 (CH×2), 55.0, 50.7, 36.9, 33.0.

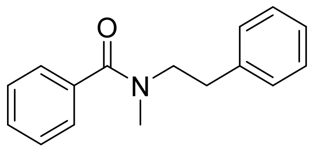

N-Methyl-N-(2-phenylethyl)benzamide (4ag'): Following the general procedure III, using the *N*-Methyl-phenethylamine **3g'** (300.0 mg, 2.22 mmol), pyridinium *p*-toluenesulfonate (11.2 mg, 0.0440 mmol), *N*,*N*-dimethylformamide (0.258 mL, 3.33 mmol), hexamethyldisilazane (0.707 mL, 3.33 mmol), thiobenzoic acid **1a** (0.881 mL, 6.66 mmol) and 1,8-Diazabicyclo[5.4.0]undec-7-ene (0.498 mL, 3.33 mmol). The crude product was then purified by column chromatography (Hexane:EtOAc = 5:1) to

afford compound **4ag'** (374.6 mg, 70% yield) as pale yellow oil. ATR-IR (cm^{-1})：3240, 3056, 2923, 2856, 1619, 1425, 1283, 1220, 1132, 789, 708, 637. ; ^1H NMR (400 MHz, $CDCl_3$) δ 7.37–6.97 (m, 10H), 3.78 (s, 1H), 3.46 (s, 1H), 3.14 (s, 1.5H), 3.00 (s, 1H), 2.82 (s, 2.5H); $^{13}C\{^1H\}$ NMR (100 MHz, $CDCl_3$) δ172.3, 171.4, 139.2, 138.0, 136.7, 129.5, 129.3, 129.0, 128.9, 128.7, 128.4, 127.0, 126.7, 126.5, 53.1, 49.8, 38.4, 34.9, 33.6, 33.2. HRMS (EI) m/z : $[M]^+$ calcd. for $C_{16}H_{17}NO$: 239.1310, found: 239.1314.

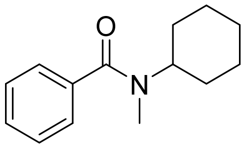

***N*-Cyclohexyl-*N*-methylbenzamide (4ah')**: Following the general procedure III, using the Methylcyclohexylamine **3h'** (300.0 mg, 2.65 mmol), pyridinium *p*-toluenesulfonate (13.3 mg, 0.053 mmol), *N,N*-dimethylformamide (0.308 mL, 3.98 mmol), hexamethyldisilazane (0.844 mL, 3.98 mmol), thiobenzoic acid **1a** (1.05 mL, 7.95 mmol) and 1,8-Diazabicyclo[5.4.0]undec-7-ene (0.594 mL, 3.98 mmol). The crude product was then purified by column chromatography (Hexane:EtOAc = 5:1) to afford compound **4ah'** (291.9 mg, 51% yield) as a white solid; m.p. 86 – 90°C；ATR-IR (cm^{-1})：3068, 3027, 3000, 2980, 2951, 2851, 1610, 1430, 1326, 1256, 1070, 898, 729, 205, 638. ; ^1H NMR (400 MHz, $CDCl_3$) δ 7.38 (br, 5H), 4.53(br, 0.5H), 3.45(br, 0.5H), 2.97(s, 2H), 2.78(s, 2H), 1.80–1.63(m, 4H), 1.56–1.47 (m, 4H), 1.05(br, 3H); $^{13}C\{^1H\}$ NMR (100 MHz, $CDCl_3$) δ 171.8, 137.3, 129.2, 128.5, 126.8, 126.2, 58.3, 52.8, 32.1, 30.9, 29.7, 27.5, 25.6, 25.2. HRMS (EI) m/z : $[M]^+$ calcd. for $C_{14}H_{19}NO$: 217.1466, found: 217.1476.

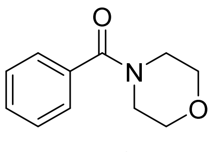

Morpholino(phenyl)methanone (4ai')[29]: Following the general procedure III, using the morpholine **3i'** (300.0 mg, 3.44 mmol), pyridinium *p*-toluenesulfonate (17.3 mg, 0.07 mmol), *N,N*-dimethylformamide (0.400 mL, 5.17 mmol), hexamethyldisilazane (1.097 mL, 5.17 mmol), thiobenzoic acid **1a** (1.220 mL, 10.33 mmol) and 1,8-Diazabicyclo[5.4.0]undec-7-ene (0.772 mL, 5.17 mmol). The crude product was then purified by column chromatography (Hexane:EtOAc = 5:1) to afford compound **4ai'** (408.3 mg, 62% yield) as a brown liquid. ^1H NMR (400 MHz, $CDCl_3$) δ 7.40–7.35 (m, 5H), 3.86–3.30 (m, 8H); $^{13}C\{^1H\}$ NMR (100 MHz, $CDCl_3$) δ 170.5, 135.3, 129.9, 128.6 (CH×2), 127.1 (CH×2), 66.9 (CH×2), 48.2, 42.5.

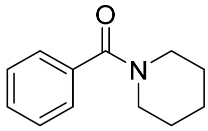

Phenyl(piperidin-1-yl)methanone (4aj')[28]**:** Following the general procedure III, using the piperidine **3j'** (300.0 mg, 3.52 mmol), pyridinium *p*-toluenesulfonate (17.7 mg, 0.07 mmol), *N*,*N*-dimethylformamide (0.409 mL, 5.28 mmol), hexamethyldisilazane (1.122 mL, 5.28 mmol), thiobenzoic acid **1a** (1.248 mL, 10.57 mmol) and 1,8-Diazabicyclo[5.4.0]undec-7-ene (0.790 mL, 5.28 mmol). The crude product was then purified by column chromatography (Hexane:EtOAc = 5:1) to afford compound **4aj'** (416.0 mg, 62% yield) as a brown liquid. ^{1}H NMR (400 MHz, $CDCl_3$) δ 7.40–7.34 (m, 5H), 3.70 (s, 2H), 3.33 (s, 2H), 1.58 (m, 6H); ^{13}C{^{1}H} NMR (100 MHz, $CDCl_3$) δ 170.4, 136.6, 129.5, 128.5 (CH×2), 126.9 (CH×2), 48.9, 43.2, 26.6, 25.7, 24.7.

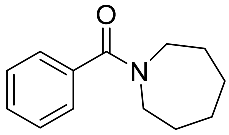

(Hexahydro-1*H*-azepin-1-yl)phenylmethanone (4ak'): Following the general procedure III, using the Dibutylamine **3k'** (300.0 mg, 3.02 mmol), pyridinium *p*-toluenesulfonate (15.2 mg, 0.0605 mmol), *N*,*N*-dimethylformamide (0.351mL, 4.54 mmol), hexamethyldisilazane (0.964 mL, 4.54 mmol), thiobenzoic acid **1a** (1.2 mL, 9.07 mmol) and 1,8-Diazabicyclo[5.4.0]undec-7-ene (0.678 mL, 4.54 mmol). The crude product was then purified by column chromatography (Hexane:EtOAc = 5:1) to afford compound **4ak'** (487.7 mg, 80% yield) as a brown oil. ATR-IR (cm^{-1})：3059, 3028, 2930, 2862, 1619, 1463, 1401, 1299, 1247, 1047, 1020, 696. ; ^{1}H NMR (400 MHz, $CDCl_3$) δ 7.37 (s, 5H), 3.67 (t, J = 5.8 Hz, 2H), 3.36 (t, J = 5.6 Hz, 2H), 1.85 – 1.80 (m, 2H), 1.65 – 1.59 (m, 6H); ^{13}C{^{1}H} NMR (100 MHz, $CDCl_3$) δ 171.6, 137.3, 129.0, 128.3, 126.4, 49.7, 46.3, 29.5, 27.8, 27.2, 26.4. HRMS (EI) m/z : [M]$^+$ calcd. for $C_{13}H_{17}NO$: 203.1310, found: 203.1315.

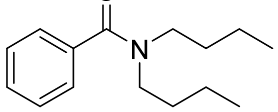

***N*,*N*-Dibutylbenzamide (4al')**[38]: Following the general procedure III, using the Dibutylamine **3l'** (300.0 mg, 2.32 mmol), pyridinium *p*-toluenesulfonate (11.8 mg, 0.0464 mmol), *N*,*N*-dimethylformamide (0.270 mL, 3.48 mmol), hexamethyldisilazane (0.739 mL, 3.48 mmol), thiobenzoic acid **1a** (0.922 mL, 6.96 mmol) and 1,8-Diazabicyclo[5.4.0]undec-7-ene (0.521 mL, 3.48 mmol). The crude product was then purified by column chromatography (Hexane:EtOAc = 5:1) to afford compound **4al'** (258.9 mg, 40% yield) as a pale yellow oil. ^{1}H NMR (400 MHz,

$CDCl_3$) δ 7.39–7.32 (m, 5H), 3.48 (s, 2H), 3.18 (s, 2H), 1.65 (br, 2H), 1.48 (br, 2H), 1.41 (br, 2H), 1.13 (br, 2H), 0.97 (br, 3H), 0.78 (br, 3H); $^{13}C\{^1H\}$ NMR (100 MHz, $CDCl_3$) δ 171.7, 137.4, 129.0, 128.4, 126.5, 48.8, 44.5, 30.8, 29.7, 20.3, 19.8, 14.0, 13.6.

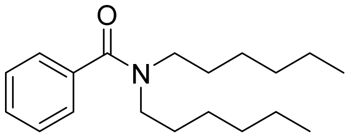

***N*,*N*-Dihexylbenzamide (4am')**[39]: Following the general procedure III, using the Dihexylamine **3m'** (300.0 mg, 1.62 mmol), pyridinium *p*-toluenesulfonate (8.14 mg, 0.0324 mmol), *N*,*N*-dimethylformamide (0.188 mL, 2.43 mmol), hexamethyldisilazane (0.516 mL, 2.43 mmol), thiobenzoic acid **1a** (0.643 mL, 4.86 mmol) and 1,8-Diazabicyclo[5.4.0]undec-7-ene (0.363 mL, 2.43 mmol). The crude product was then purified by column chromatography (Hexane:EtOAc = 5:1) to afford compound **4am'** (144.4 mg, 31% yield) as brown oil. ^1H NMR (400 MHz, $CDCl_3$) δ 7.38–7.32 (m, 5H), 3.47 (br, 2H), 3.16 (br, 2H), 1.63 (br, 2H), 1.47 (br, 2H), 1.34 (s, 6H), 1.20 (br, 2H), 1.11 (br, 4H), 0.90 (br, 3H), 0.82 (br, 3H); $^{13}C\{^1H\}$ NMR (100 MHz, $CDCl_3$) δ 171.7, 137.5, 129.1, 128.4, 126.5, 49.1, 44.8, 31.7, 31.3, 28.7, 27.6, 26.8, 26.2, 22.7, 22.5, 14.1, 14.0.

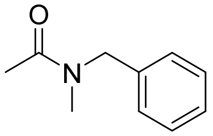

***N*-Benzyl-*N*-methylacetamide (4b'f')**[42]: Following the general procedure III, using the *n*-benzylmethylamine **3f'** (300.0 mg, 2.48 mmol), pyridinium *p*-toluenesulfonate (12.4 mg, 0.05 mmol), *N*,*N*-dimethylformamide (0.288 mL, 3.71 mmol), hexamethyldisilazane (0.789 mL, 3.71 mmol), thioacetic acid **1b'** (0.526 mL, 7.43 mmol) and 1,8-Diazabicyclo[5.4.0]undec-7-ene (0.555 mL, 3.71 mmol). The crude product was then purified by column chromatography to afford compound **4b'f'** (369.9 mg, 91% yield) as brown oil. ^1H NMR (400 MHz, $CDCl_3$) ^1H NMR (400 MHz, $CDCl_3$) δ 7.40–7.21 (m, 8H), 7.20–14 (m, 2H), 4.58 (s, 2H), 4.52 (s, 2H), 2.94 (s, 3H), 2.92 (s, 3H), 2.16 (s, 3H), 2.15 (s, 3H); $^{13}C\{^1H\}$ NMR (100 MHz, $CDCl_3$) δ 170.3, 170.0, 136.7, 135.9, 128.2, 127.9, 127.3, 126.9, 126.6, 125.7, 53.4, 49.8, 34.8, 32.9, 21.0, 20.7.

Byproducts (5 and **7a)**：

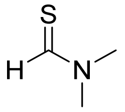

***N*,*N*-Dimethylmethanethioamide (5)**[40]**:** Following the general procedure II, the crude

product was then purified by column chromatography (gradient elution: 10% to 50% EtOAc in CH_2Cl_2) to afford byproduct **5** (49.2 mg, 85% yield) as a light yellow oil. ^{1}H NMR (400 MHz, $CDCl_3$) δ 9.20 (s, 1H), 3.29 (d, *J* = 10.6 Hz, 6H); ^{13}C{^{1}H} NMR (100 MHz, $CDCl_3$) δ 188.3, 45.4, 37.4.

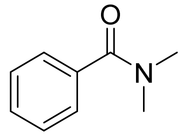

***N,N*-Dimethylbenzamide (7a)**[41]**:** ^{1}H NMR (400 MHz, $CDCl_3$) δ 7.41–7.37 (m, 5H), 3.10 (s, 3H), 2.97 (s, 3H),; ^{13}C{^{1}H} NMR (100 MHz, $CDCl_3$) δ 171.8, 136.4, 129.6, 128.4 (CH×2), 127.1 (CH×2), 39.7, 35.4.

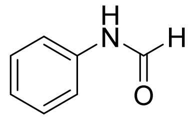

***N*-Phenylformamide (9)**[43]**:** ^{1}H NMR (400 MHz, $CDCl_3$; Mixture of rotamers is observed. Ratio: 1.00/1.24) δ 8.70 (d, *J* = 11.3 Hz, 1H) (major), 8.55 (brs, 1H), 8.36 (s, 1H) (minor), 7.64 (brs, 1H)7.54 (d, *J* = 7.8 Hz, 2H), 7.34 (d, *J* = 7.3 Hz, 4H), 7.22 – 7.05 (m, 4H); ^{13}C{^{1}H} NMR (100 MHz, $CDCl_3$) δ 162.9, 159.3, 137.0, 136.8, 129.9 (CH×2), 129.2 (CH×2), 125.4, 124.9, 120.1 (CH×2), 118.9 (CH×2).

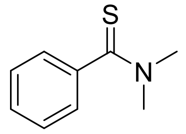

***N,N*-dimethylbenzothioamide (10)**[44]**:**^{1}H NMR (400 MHz, $CDCl_3$) δ 7.36 – 7.15 (m, 5H), 3.53 (s, 3H), 3.08 (s, 3H); ^{13}C{^{1}H} NMR (100 MHz, $CDCl_3$) δ 200.9, 143.2, 128.4, 128.1 (CH×2), 125.5 (CH×2), 44.0, 43.1.

References

1. Wang, R.; Xie, K.-J.; Fu, Q.; Wu, M.; Pan, G.-F.; Lou, D.-W.; Liang, F.-S. *Org. Lett.* **2022**, *24*, 2020–2024.
2. Yang, F.-L.; Zhu, X.; Rao, D.-K.; Cao, X.-N.; Li, K.; Xu, Y.; Hao. X.-Q.; Song, M.-P. *RSC Adv.* **2016**, *6*, 37093−37098.
3. Chouhan, K. K.; Chowdhurya, D.; Mukherjee, A. *Org. Biomol. Chem.* **2023**, *21*, 2429−2439.
4. Ghosh,S.; Jana, C. K. *Org. Lett.* **2016**, *18*, 5788–5791.
5. Sharma, A. K.; Joshi, H.; Bhaskar, R.; Singh, A. K. *Dalton Trans.* **2019**, *48*, 10962−10970.

6. Qi, X.; Ai, H.-J.; Cai, C.-X.; Peng, J.-B.; Ying, J.; Wu, X.-F. *Eur. J. Org. Chem.* **2017**, *48*, 7222−7225.
7. Kuwabara, J.; Sawada, Y.; Yoshimatsu, M. *Org. Lett.* **2018**, *20*, 1130–1133.
8. Ma, C.; Fan, G.; Wu, P.; Li, Z.; Zhou, Y.; Ding, Q.; Zhang, W. *Org. Biomol. Chem.* **2017**, *15*, 9889–9894.
9. González-Fernández, R.; Crochet, P.; Cadierno, V.; Menéndez, M. I.; López, R. *Chem. Eur. J.* **2017**, *23*, 15210–15221.
10. Garduño, J. A.; Arévalo, A.; Flores-Alamoa, M.; García, J. J. *Catal. Sci. Technol.* **2018**, *8*, 2606–2616.
11. Wang, R.; Chen, Y.; Fei, B.; Hu, J.; Chen, J.; Luo, Y.; Xia, Y. *Org. Lett.* **2023**, *25*, 2970–2974
12. Senthamarai, T.; Chandrashekhar, V. G.; Rockstroh, N.; Rabeah, J.; Bartling, S.; Jagadeesh, R. V.; Beller, M. *Chem.* **2021**, *8*, 508–531.
13. Chen, B.; Zhang, L.; Luo, H.; Huang, L.; He P.; Xue, G.; Liang, H.; Dai, W. *JACS Au.* **2023**, *3*, 476–487.
14. Sun, C.; Qua, P.; Li, F. *Catal. Sci. Technol.* **2014**, *4*, 988–996.
15. Zhang, Z.; Zheng, D.; Wan, Y.; Zhang, G.; Bi, J.; Liu, Q.; Liu, T.; Shi, L. *J. Org. Chem.* **2018**, *83*, 1369–1376.
16. Bai, J.; Li, S.; Zhu, R.; Li, Y.; Li, W. *J. Org. Chem.* **2023**, *88*, 3714–3723.
17. Shimokawa S.; Kawagoe, Y.; Moriyama, K.; Togo, H. *Org. Lett.* **2016**, *18*, 784–787.
18. Teng, Q.-H.; Sun, G.-X.; Huang, F.-P.; Wang, K.; Lianga, F.-P. *Adv. Synth. Catal.* **2023**, *365*, 307–311.
19. (a)Yu, H.; Zhang, X.; Li, L.; Luo, H.; Che, G. *Org. Chem. Front.* **2023**, *10*, 686–698; (b)Li, L.-J.; Zhou, Z.-Q.; Liu, Z.-K.; He, Y.-Y.; Jia, F.-C.; Hu, X.-Q. *Chem. Commun.* **2023**, *59*, 438–441.
20. Pandey, K.; Muthyala, M. K.; Choudhary, S.; Kumar, A. *RSC Adv.* **2015**, *5*, 13797–13804.
21. Yu, S.; Song, K. H.; Lee, S. *Asian J. Org.Chem.* **2019**, *8*, 1613–1616.
22. Bradley, R. D.; Bahamonde, A. *Org. Lett.* **2022**, *24*, 7134−7139.
23. Magyar, C. L.; Wall, T. J.; Davies, S. B.; Campbell, M. V.; Barna, H. A.; Smith, S. R.; Savich, C. J.; Mosey, R. A. *Org. Biomol. Chem.* **2019**, *17*, 7995–8000.
24. Rajan, I. A. P. S.; Subramani, M.; Pushparathinam, G.; Rajendran, S. *Asian J. Org. Chem.* **2022**, *11*, e202200378.
25. Du, J.; Luo, K.; Zhang, X. *RSC Adv.* **2014**, *4*, 54539–54546.
26. Zhang, J.; Hou, Y.; Ma,Y.; Szostak, M. *J. Org. Chem.* **2019**, *84*, 338−345.
27. Bai, J.; Li, S.; Zhu, R.; Li, Y.; Li, W. *J. Org. Chem.* **2023**, *88*, 3714−3723.
28. Fan, W.; Yang, Y.; Lei, J.; Jiang, Q.; Zhou, W. *J. Org. Chem.* **2015**, *80*, 8782−8789.

29. Pan, B.; Huang, D.-M.; Sun, H.-T.; Song, S.-N.; Su, X.-B. *J. Org. Chem.* **2023**, *88*, 2832−2840.
30. Liu, Y.; Shi, S.; Achtenhagen, M.; Liu, R.; Szostak, M. *Org. Lett.* **2017**, *19*, 1614−1617.
31. Iqbal, N.; Cho, E. J. *J. Org. Chem.* **2016**, *81*, 1905−1911.
32. Otsuka, R.; Maruhashi, K.; Ohwada, T. *Synthesis* **2018**, *50*, 2041−2057.
33. Wang, W.-Q.; Yuan, Y.; Miao, Y.; Yu, B.-Y.; Wang, H.-J.; Wang, Z.-Q.; Sang, W.; Chen, C.; Verpoort, F. *Appl. Organomet. Chem.* **2019**, *34*, e5323.
34. Ling, L.; Chen, C.; Luo, M.; Zeng, X. *Org. Lett.* **2019**, *21*, 1912−1916.
35. Fang, W.-Y.; Huang, Y.-M.; Leng, J.; Qin, H.-L. *Asian J. Org. Chem.* **2018**, *7*, 751–756.
36. Teng, Q.-H.; Sun, G.-X.; Huang, F.-P.; Wang, K.; Liang, F.-P. *Adv. Synth. Catal.* **2023**, *365*, 307–311.
37. Kumar, S.; Vanjari, R.; Guntreddi, T.; Singh, K. N. *RSC Adv.* **2015**, *5*, 9920–9924.
38. Zhu, M.; Fujita, K.-i.; Yamaguchi, R. *J. Org. Chem.* **2012**, *77*, 9102–9109.
39. Pathak, U.; Bhattacharyya, S.; Pandey, L. K.; Mathur, S.; Jain, R. *RSC Adv.* **2014**, *4*, 3900–3903
40. Shibahara, F.; Sugiura, R.; Murai, T. *Org. Lett.* **2009**, *11*, 3064–3067.
41. Lai, M.; Wu, Z.; Su, F.; Yu, Y.; Jing, Y.; Kong, J.; Wang, Z.; Wang, S.; Zhao, M. *Eur. J. Org. Chem.* **2020**, *2*, 198–208.
42. Fu, R. Z.; Yang, Y.; Chen, Z. K.; Lai, W. C.; Ma, Y. F.; Wang, Q.; Yuan, R. X. *Tetrahedron* **2014**, *70*, 9492–9499.
43. Leong, B.-X.; Teo, Y.-C.; Condamines, C.; Yang, M.-C.; Su, M.-D.; So, C.-W. *ACS Catal.* **2020**, *10*, 14824–14833.
44. Kumar, S.; Vanjari, R.; Guntreddi, T.; Singh, K. N. *Tetrahedron* **2016**, *72*, 2012–2017.

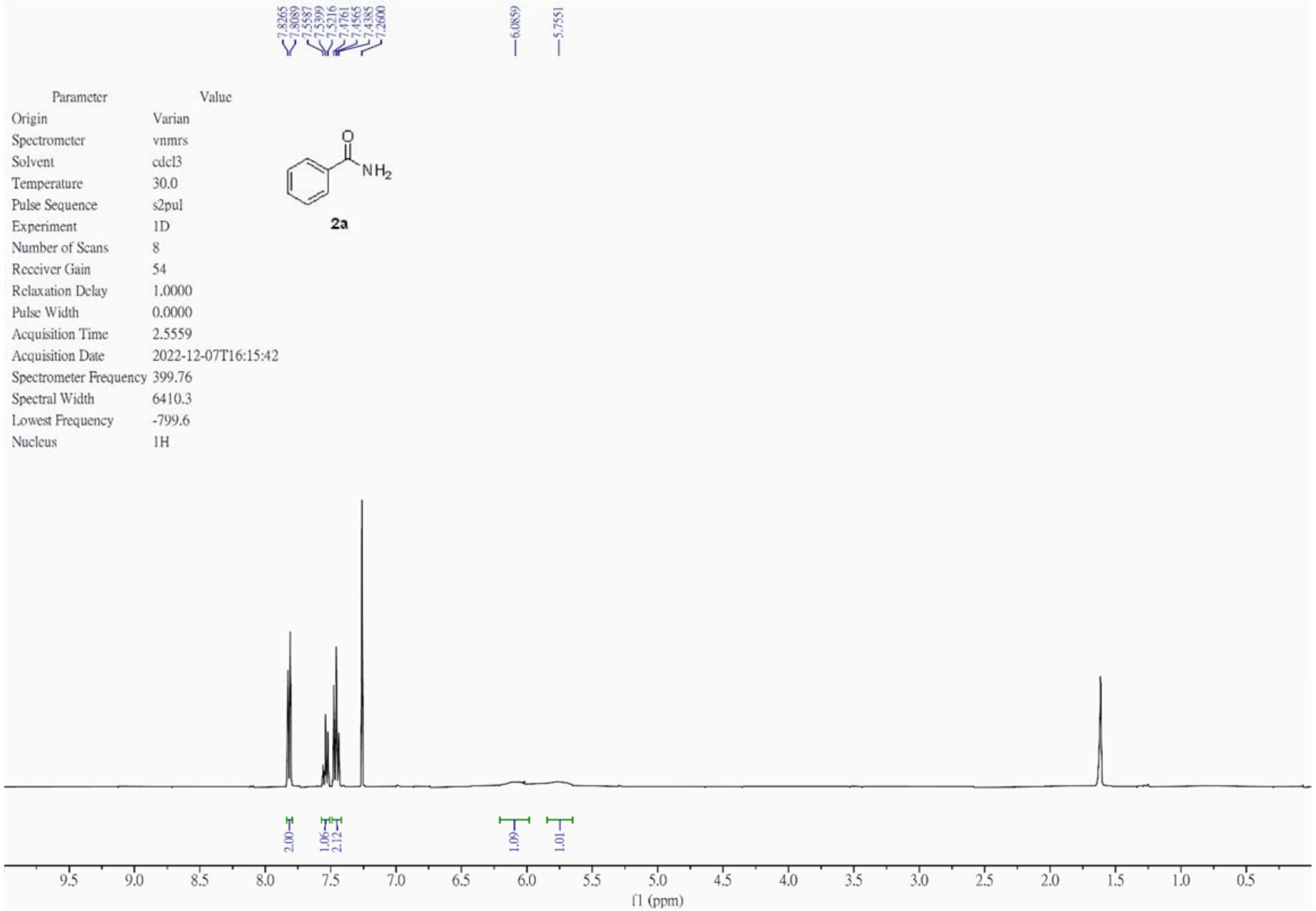

2a ^{1}H NMR spectrum (400 MHz in $CDCl_3$)

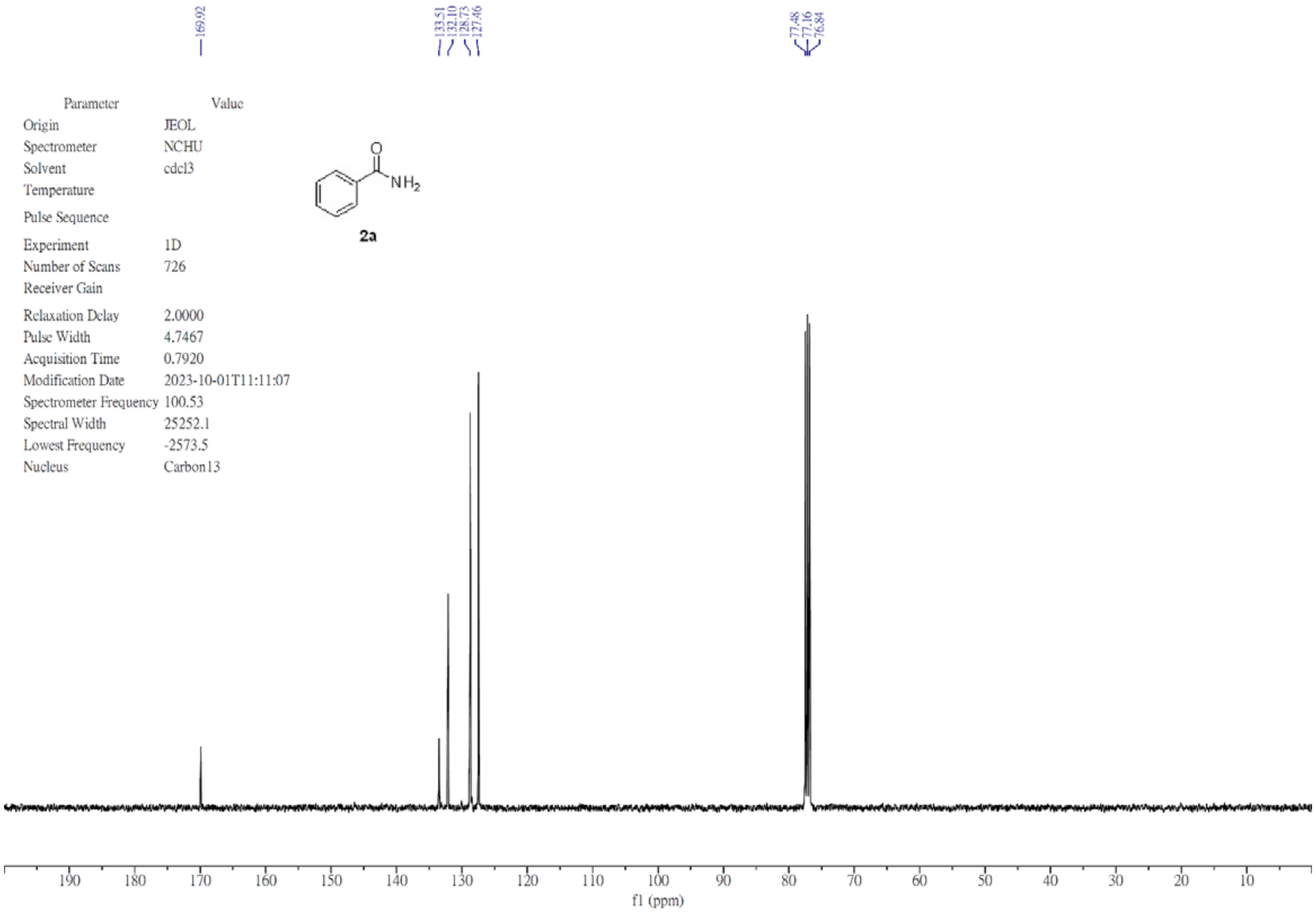

2a $^{13}C\{^{1}H\}$ NMR spectrum (100 MHz in $CDCl_3$)

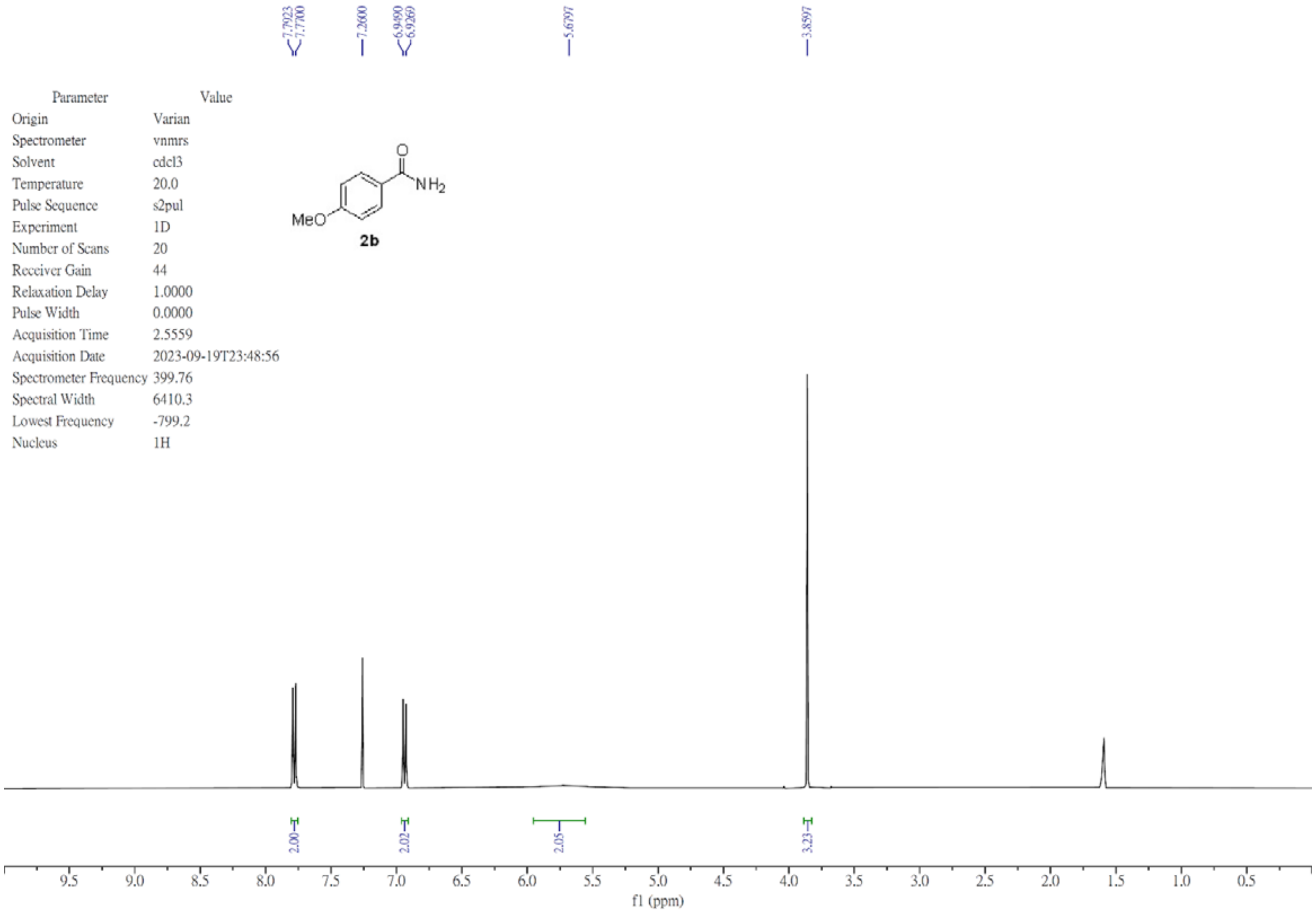

2b ^{1}H NMR spectrum (400 MHz in $CDCl_3$)

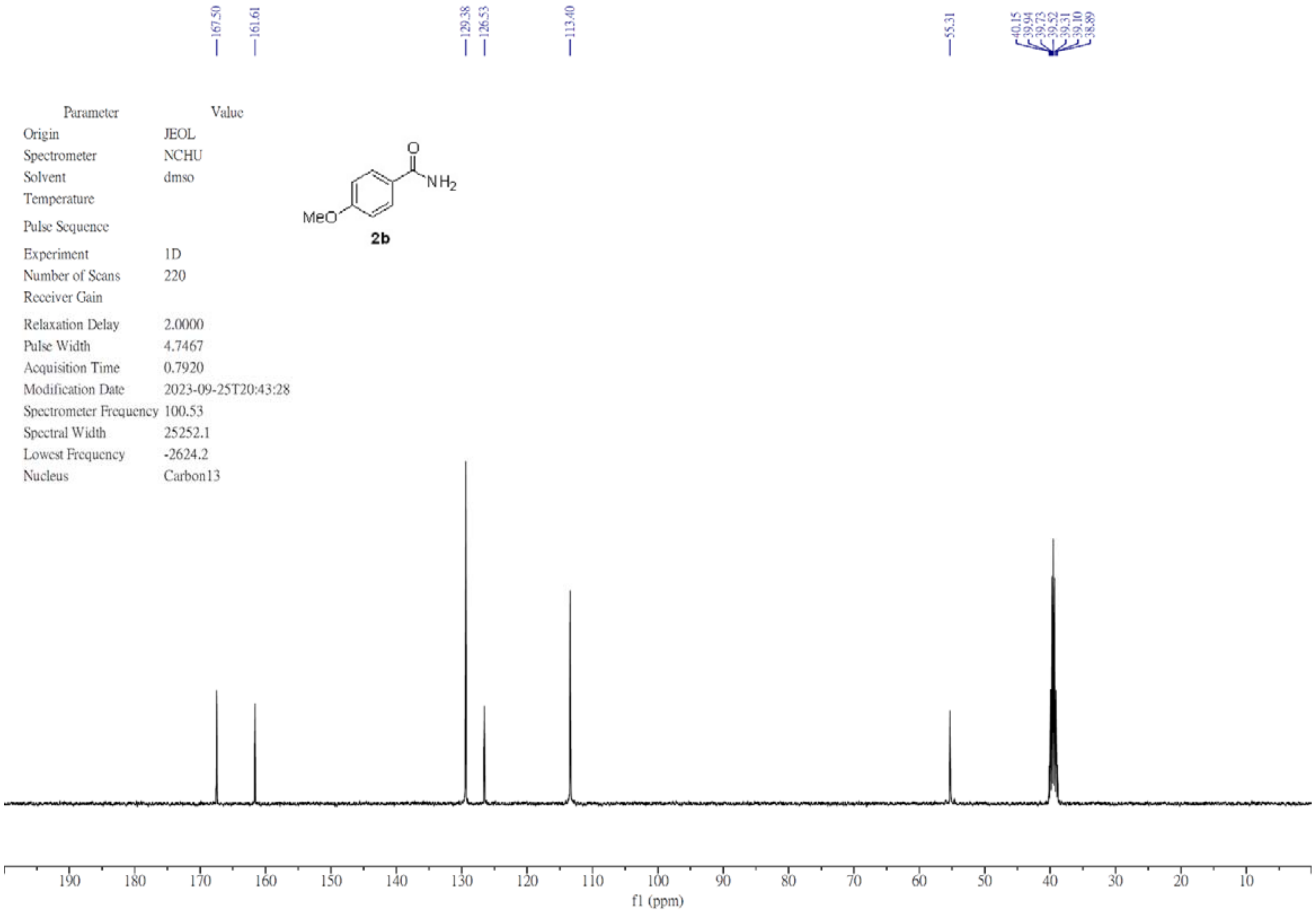

2b $^{13}C\{^{1}H\}$ NMR spectrum (100 MHz in $(CD_3)_2SO$)

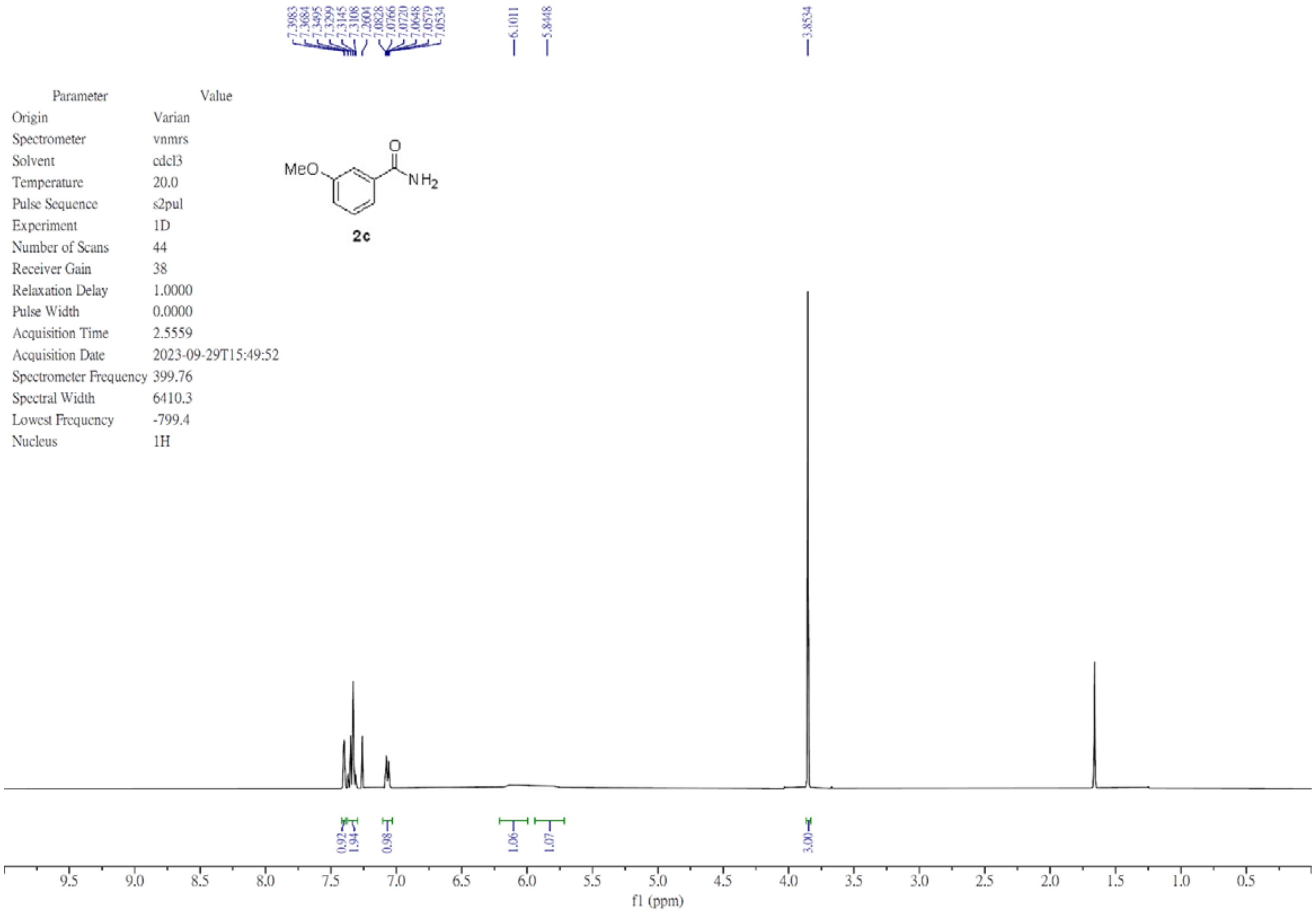

2c ^{1}H NMR spectrum (400 MHz in $CDCl_3$)

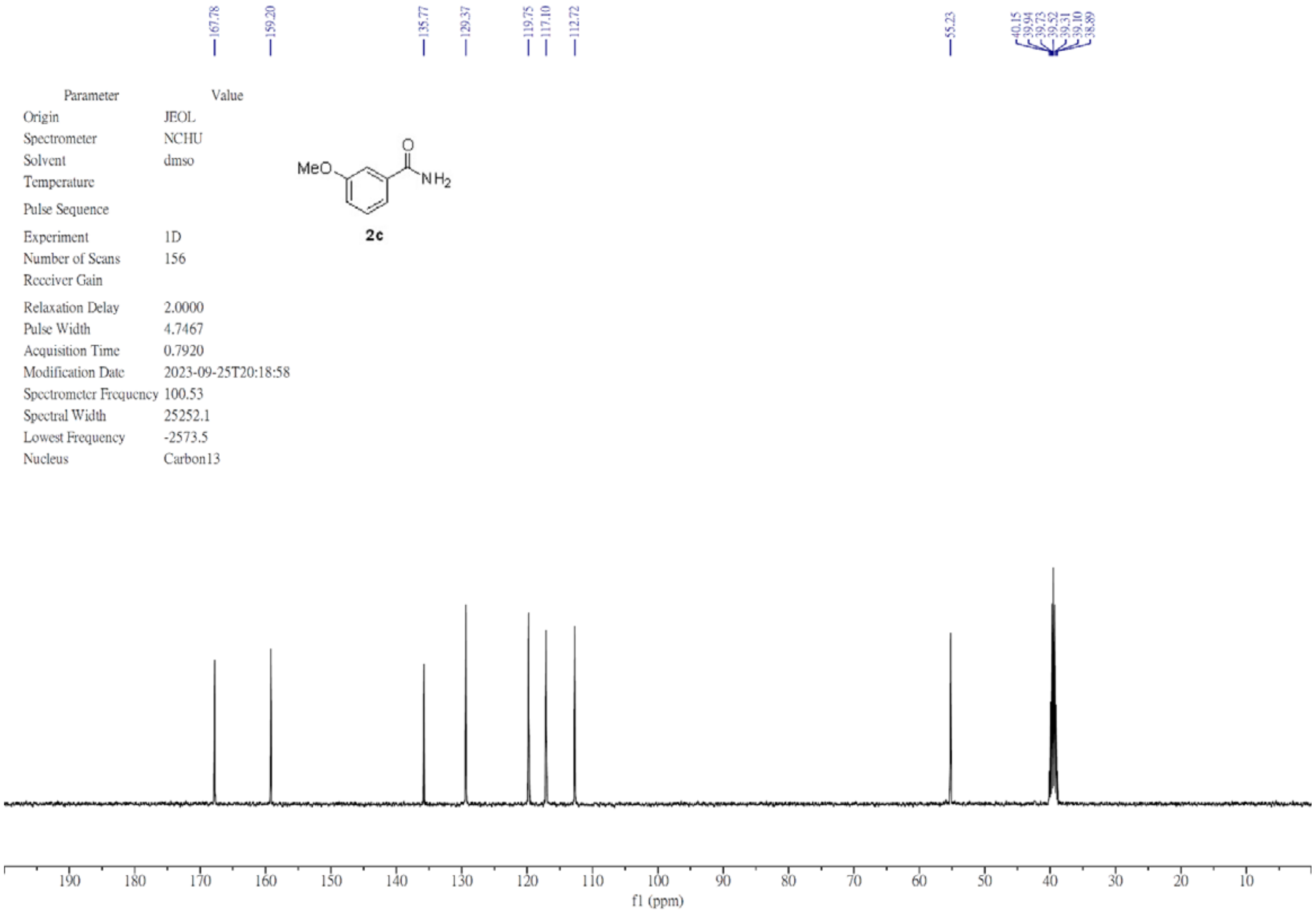

Parameter	Value
Origin	JEOL
Spectrometer	NCHU
Solvent	dmso
Temperature	
Pulse Sequence	
Experiment	1D
Number of Scans	156
Receiver Gain	
Relaxation Delay	2.0000
Pulse Width	4.7467
Acquisition Time	0.7920
Modification Date	2023-09-25T20:18:58
Spectrometer Frequency	100.53
Spectral Width	25252.1
Lowest Frequency	-2573.5
Nucleus	Carbon13

2c

2c $^{13}C\{^{1}H\}$ NMR spectrum (100 MHz in $(CD_3)_2SO$)

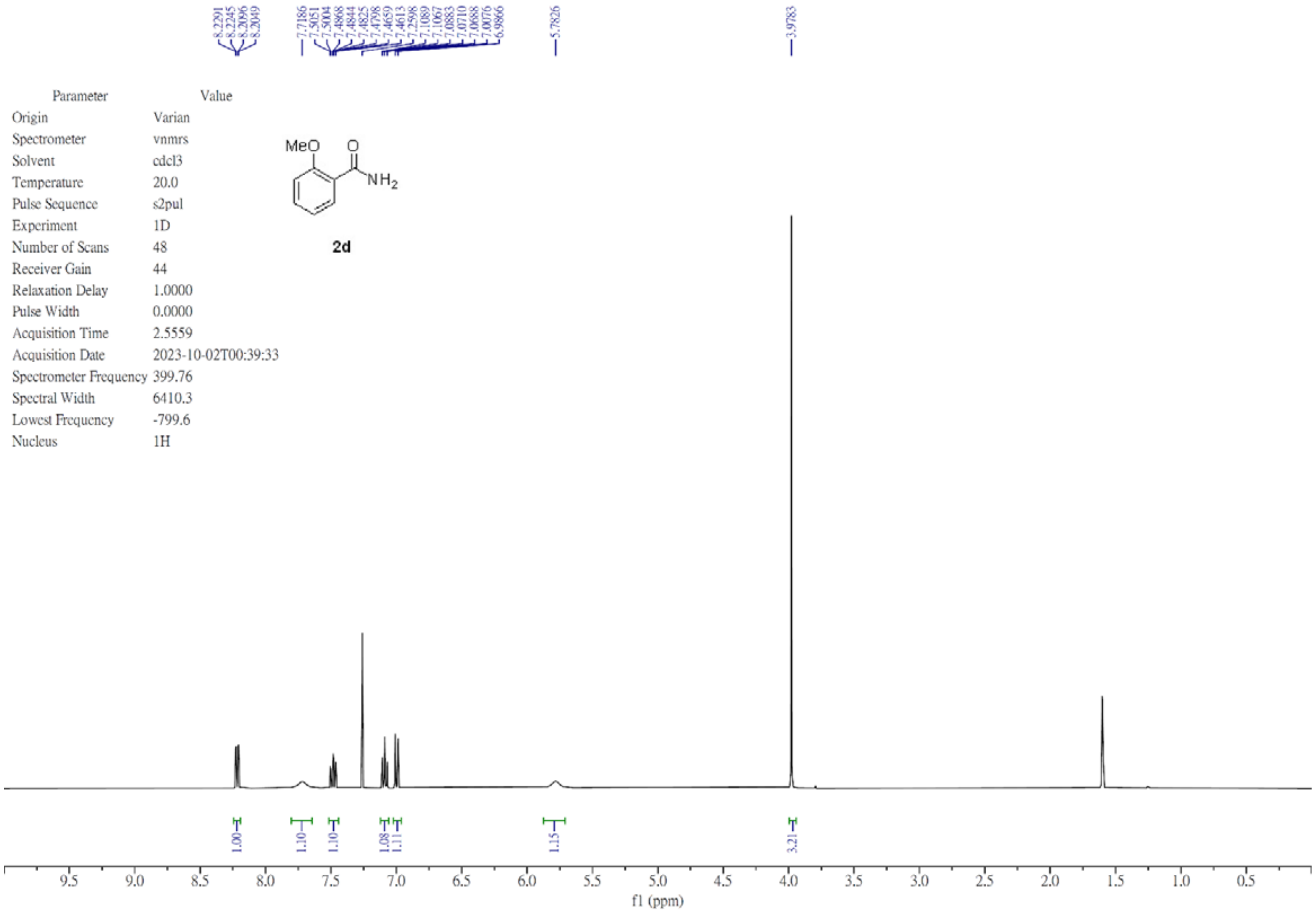

2d ^{1}H NMR spectrum (400 MHz in $CDCl_3$)

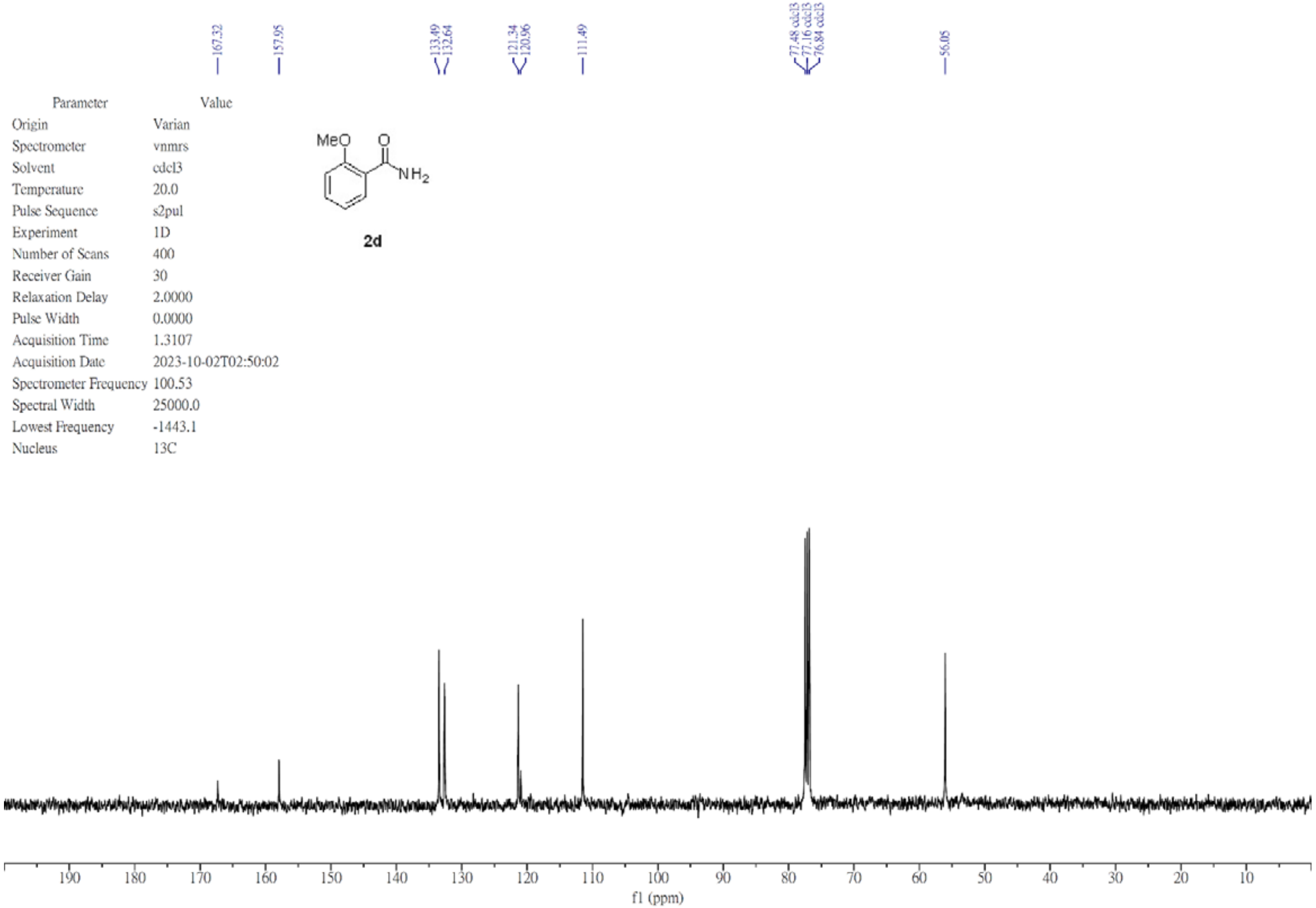

2d $^{13}C\{^{1}H\}$ NMR spectrum (100 MHz in $CDCl_3$)

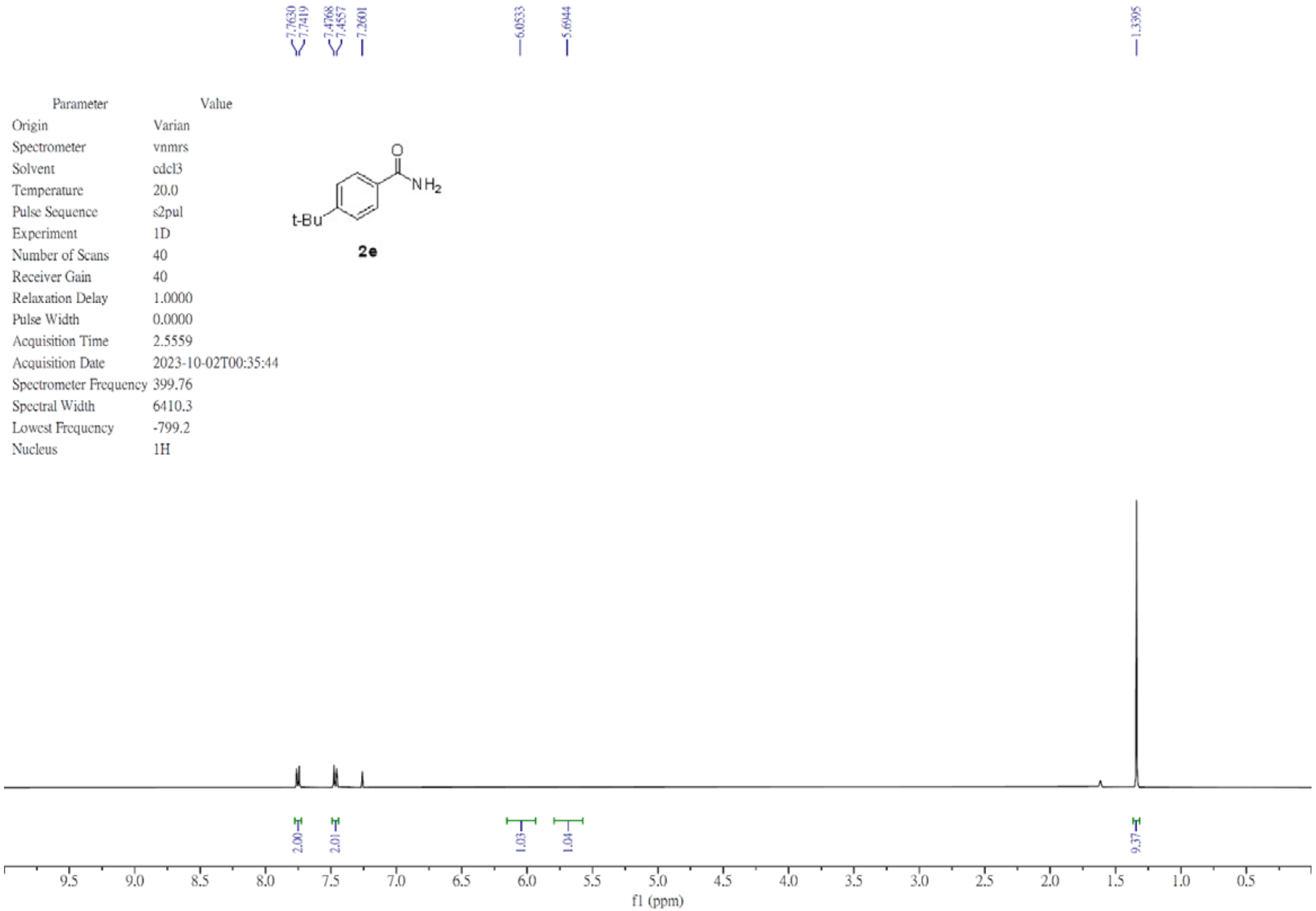

2e ^{1}H NMR spectrum (400 MHz in $CDCl_3$)

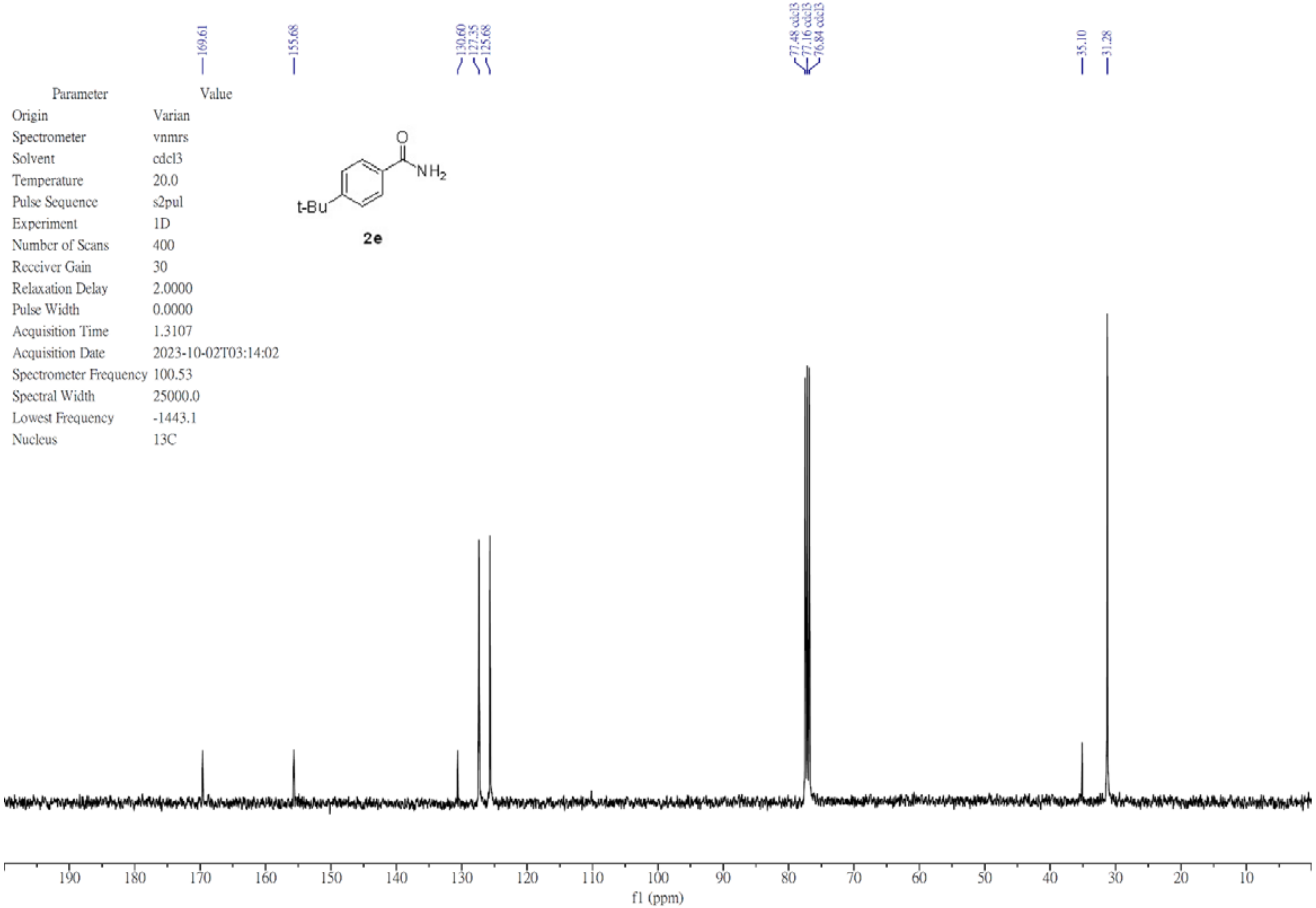

2e $^{13}C\{^{1}H\}$ NMR spectrum (100 MHz in $CDCl_3$)

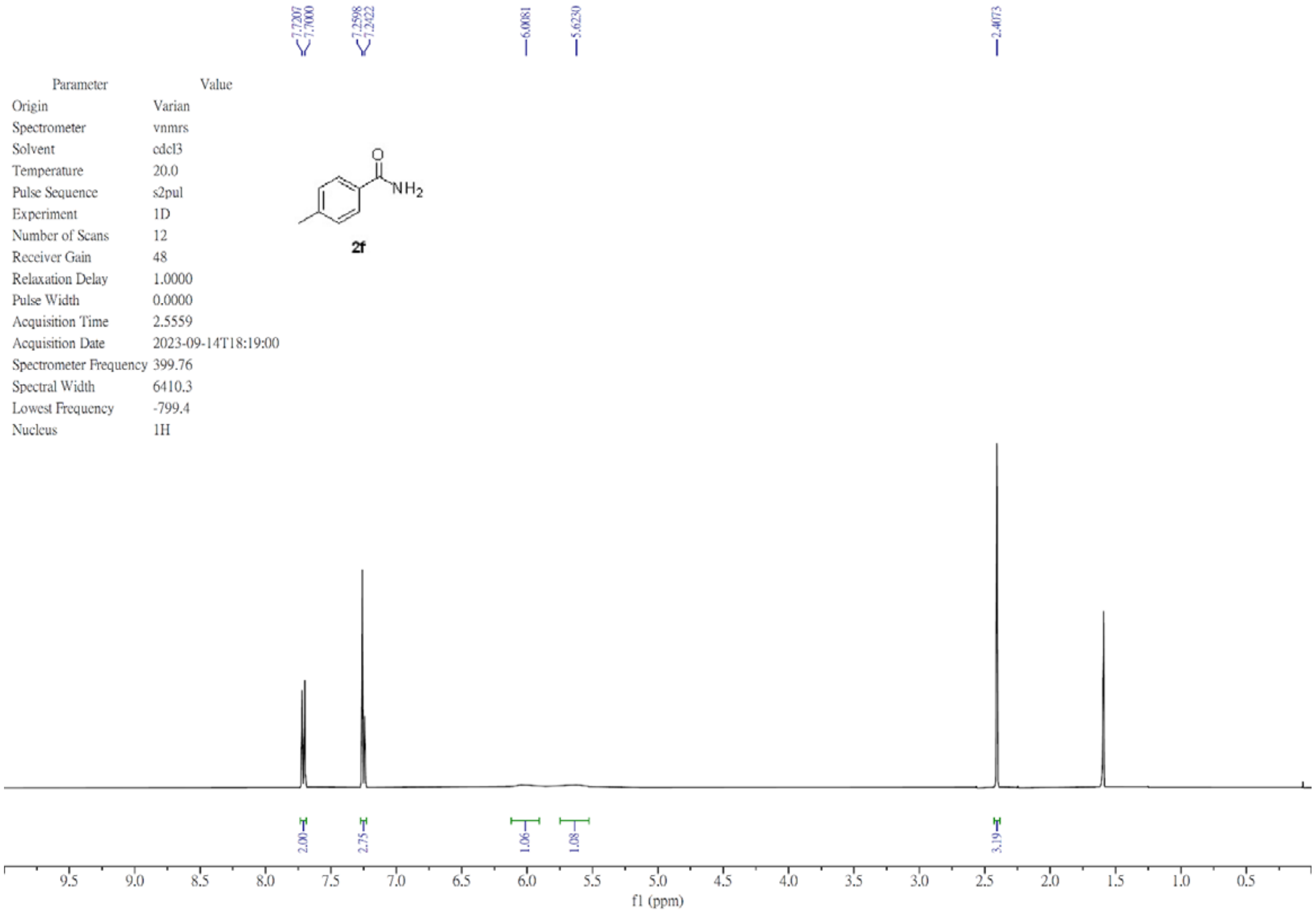

2f ^{1}H NMR spectrum (400 MHz in $CDCl_3$)

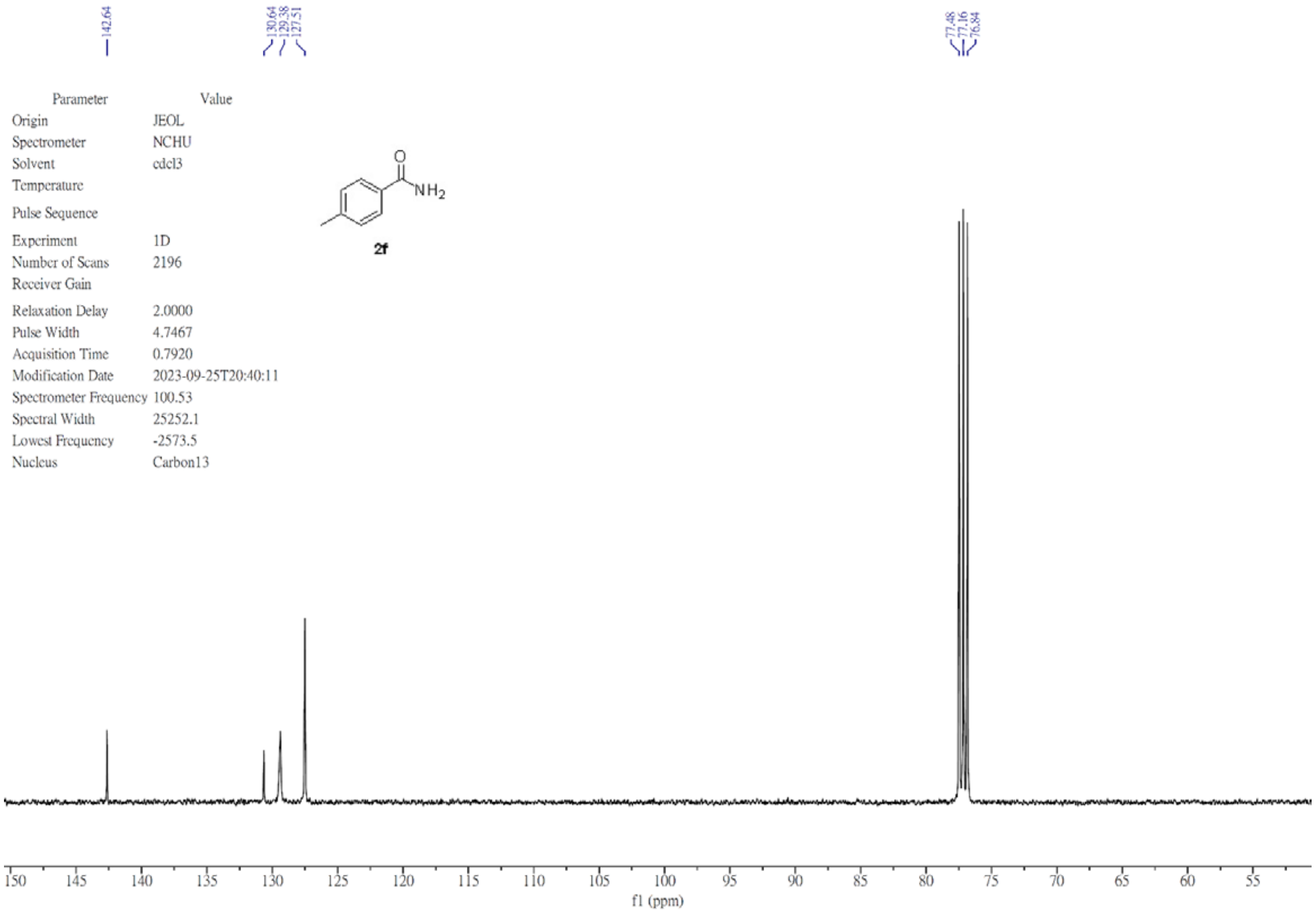

2f $^{13}C\{^{1}H\}$ NMR spectrum (100 MHz in $CDCl_3$)

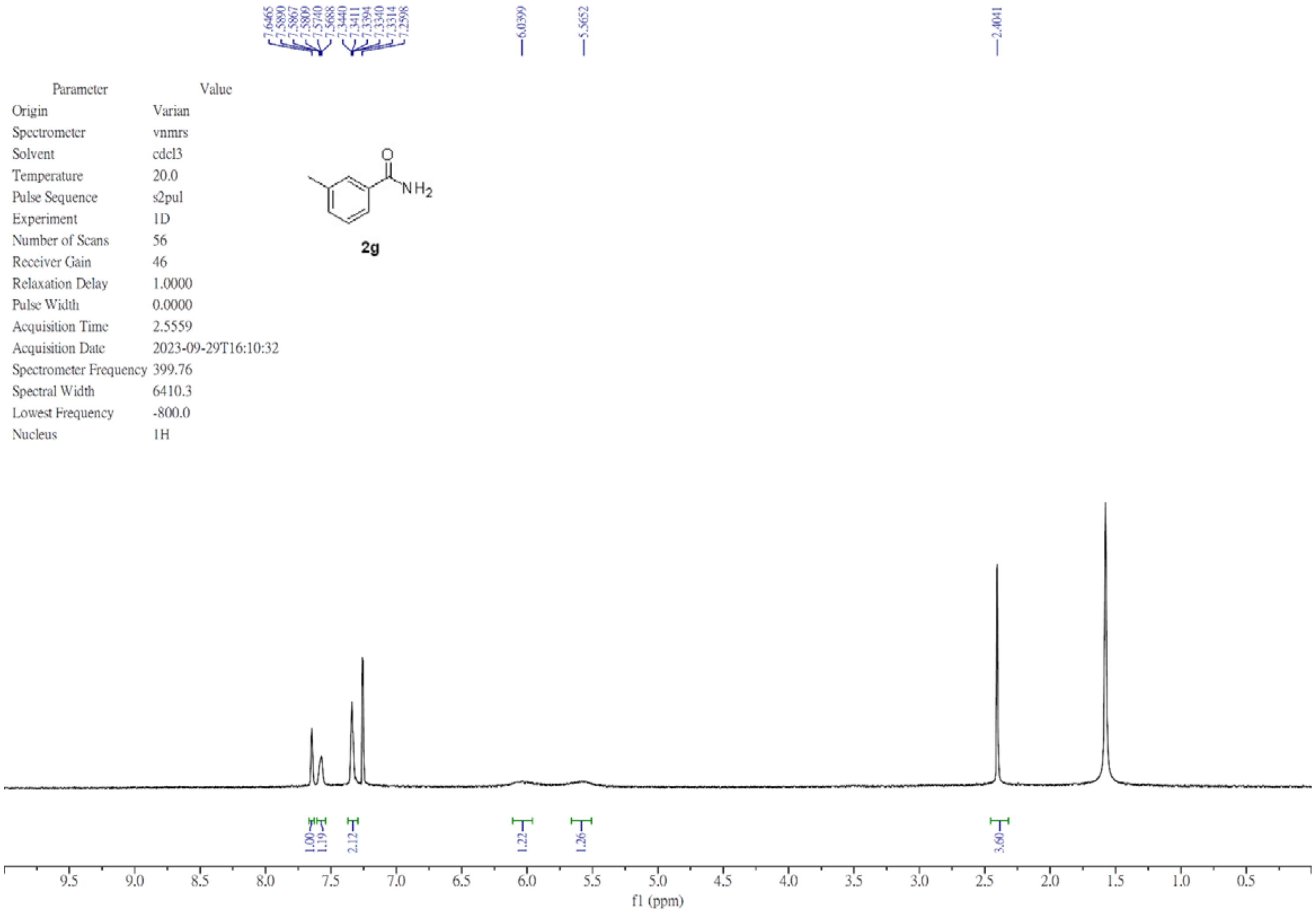

2g ^{1}H NMR spectrum (400 MHz in $CDCl_3$)

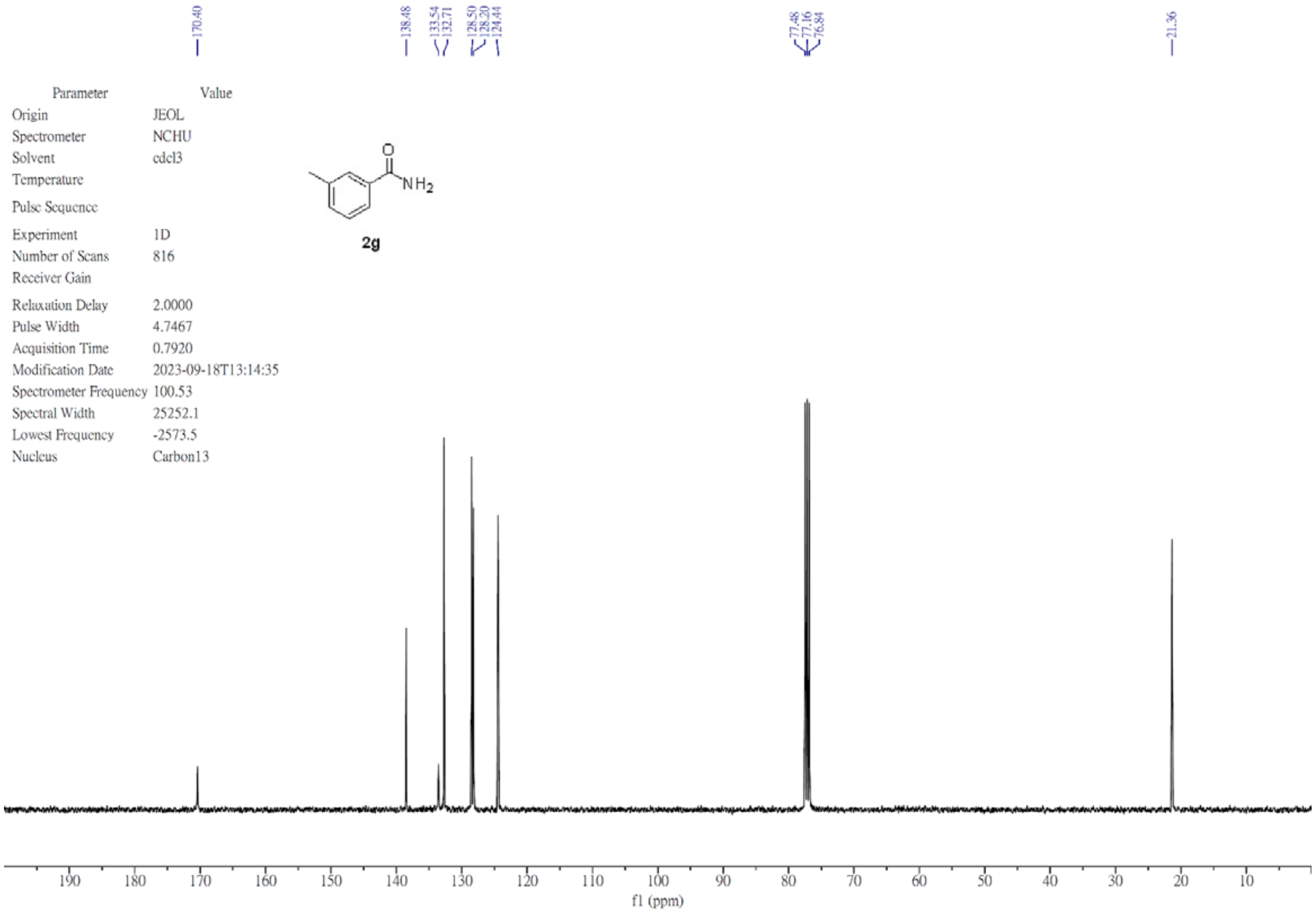

2g $^{13}C\{^{1}H\}$ NMR spectrum (100 MHz in $CDCl_3$)

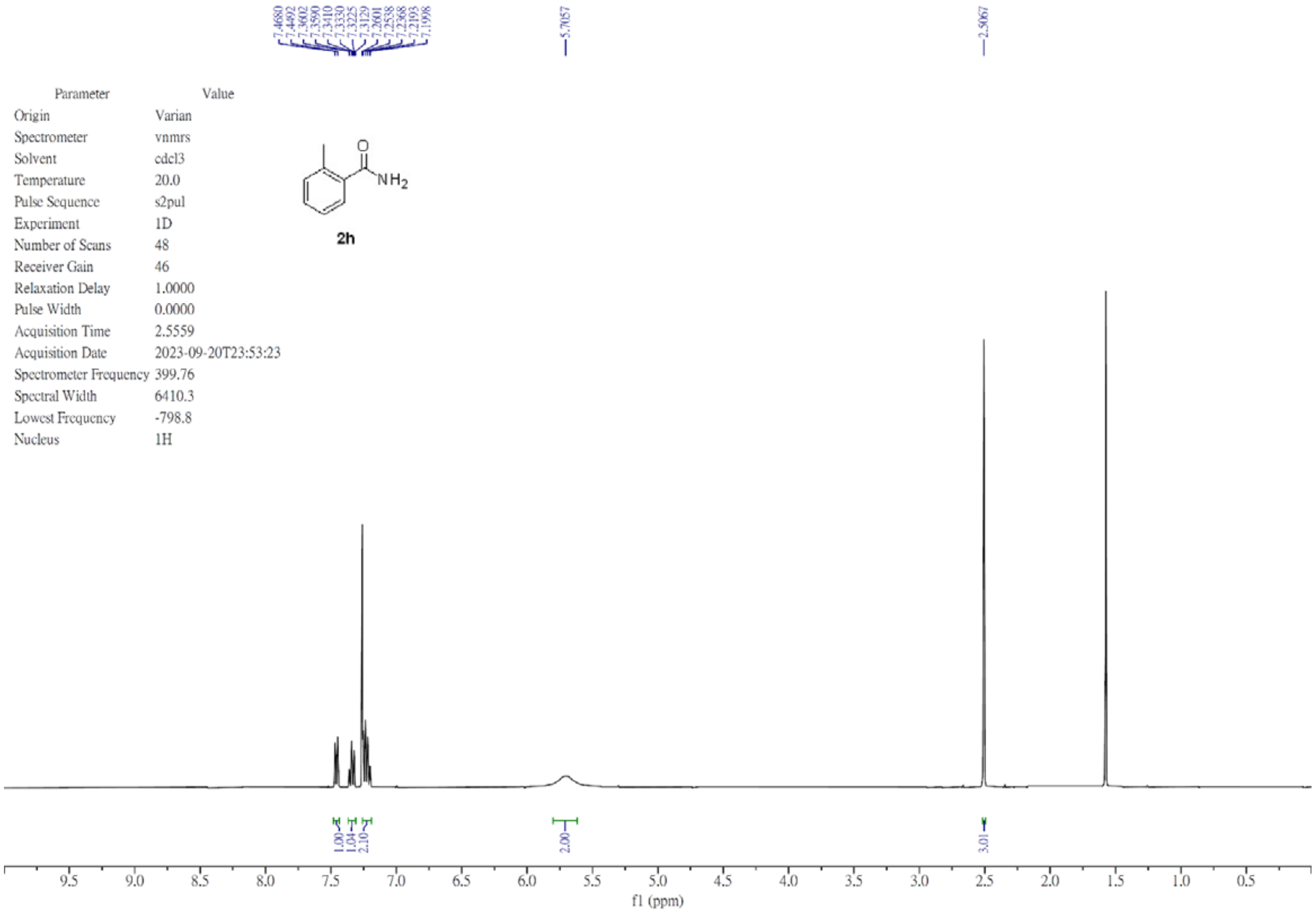

2h ^{1}H NMR spectrum (400 MHz in $CDCl_3$)

Parameter	Value
Origin	JEOL
Spectrometer	NCHU
Solvent	cdcl3
Temperature	
Pulse Sequence	
Experiment	1D
Number of Scans	251
Receiver Gain	
Relaxation Delay	2.0000
Pulse Width	4.7467
Acquisition Time	0.7920
Modification Date	2023-09-30T20:08:18
Spectrometer Frequency	100.53
Spectral Width	25252.1
Lowest Frequency	-2573.5
Nucleus	Carbon13

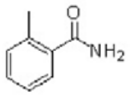

2h

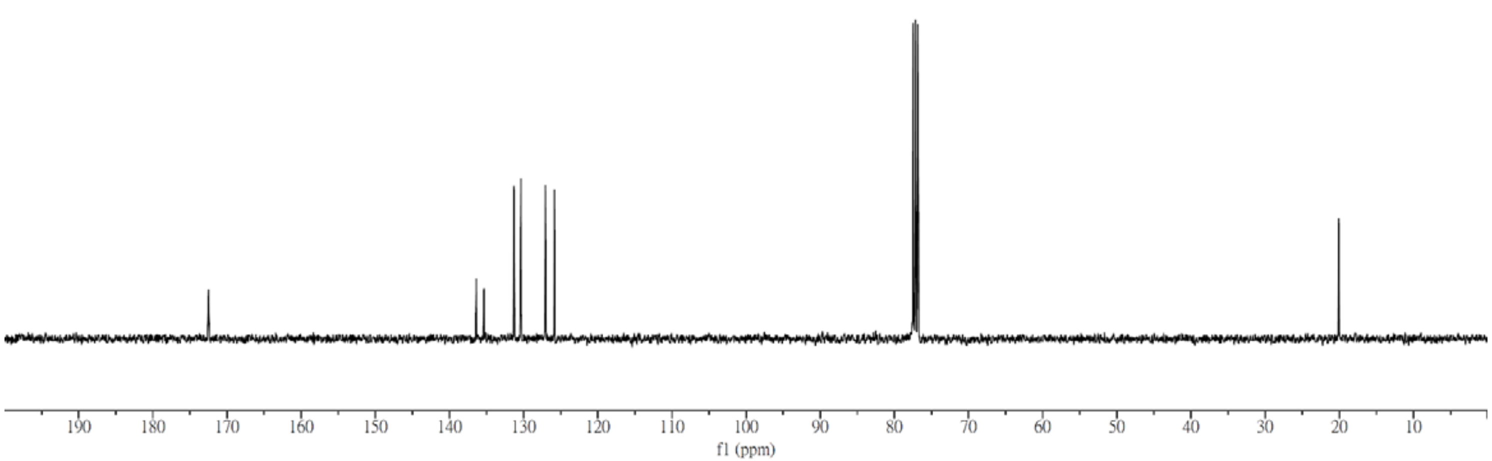

2h $^{13}C\{^{1}H\}$ NMR spectrum (100 MHz in $CDCl_3$)

Parameter	Value
Origin	Varian
Spectrometer	vnmrs
Solvent	cdcl3
Temperature	20.0
Pulse Sequence	s2pul
Experiment	1D
Number of Scans	44
Receiver Gain	42
Relaxation Delay	1.0000
Pulse Width	0.0000
Acquisition Time	2.5559
Acquisition Date	2023-09-29T15:54:16
Spectrometer Frequency	399.76
Spectral Width	6410.3
Lowest Frequency	-799.4
Nucleus	1H

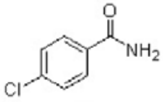

2i

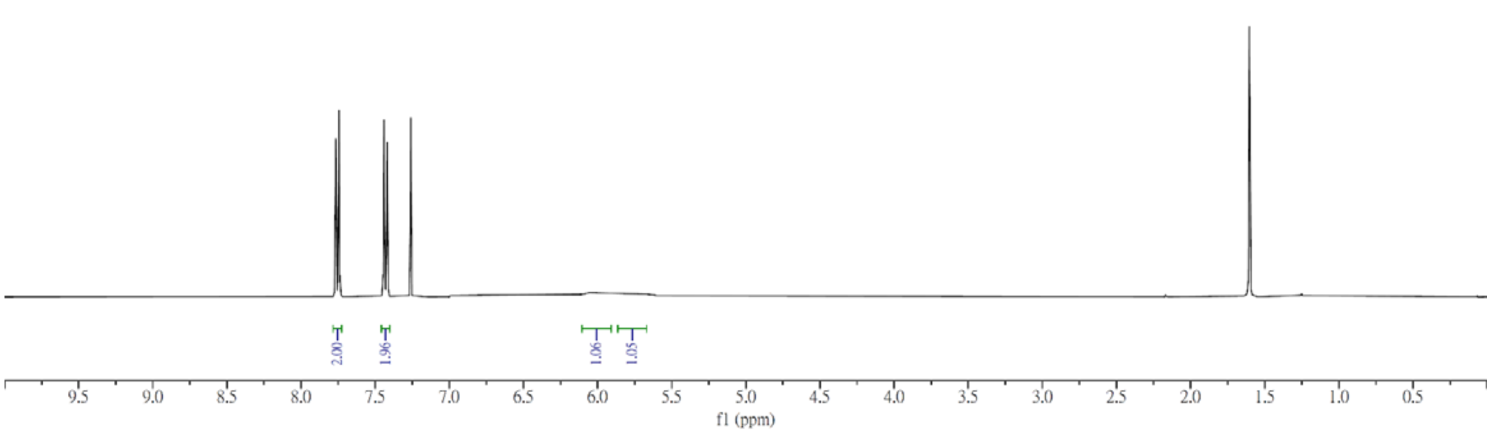

2i ^{1}H NMR spectrum (400 MHz in $CDCl_3$)

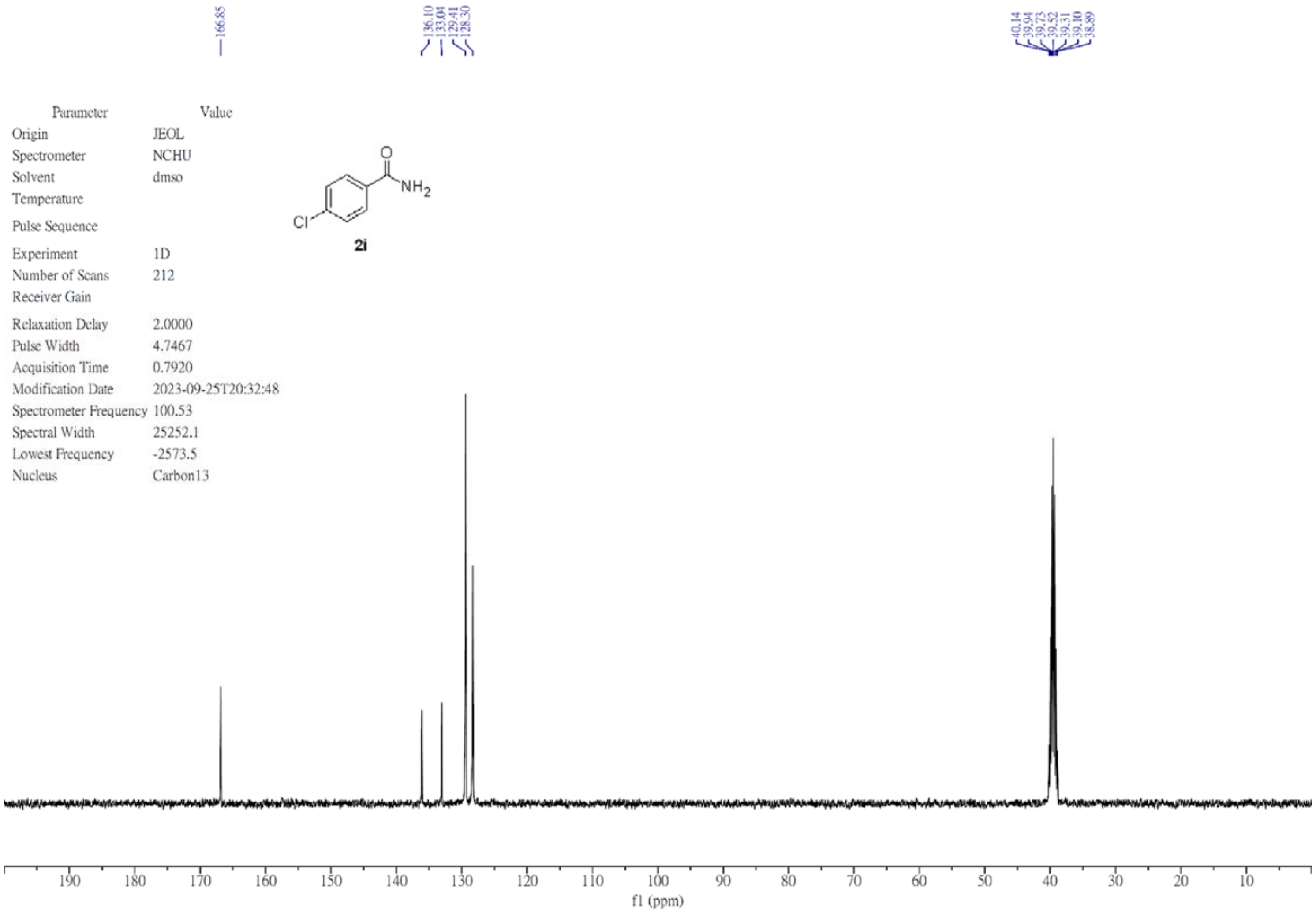

2i $^{13}C\{^{1}H\}$ NMR spectrum (100 MHz in $(CD_3)_2SO$)

Parameter	Value
Origin	Varian
Spectrometer	vnmrs
Solvent	cdcl3
Temperature	20.0
Pulse Sequence	s2pul
Experiment	1D
Number of Scans	40
Receiver Gain	48
Relaxation Delay	1.0000
Pulse Width	0.0000
Acquisition Time	2.5559
Acquisition Date	2023-10-01T05:54:56
Spectrometer Frequency	399.76
Spectral Width	6410.3
Lowest Frequency	-799.2
Nucleus	1H

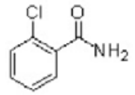

2j

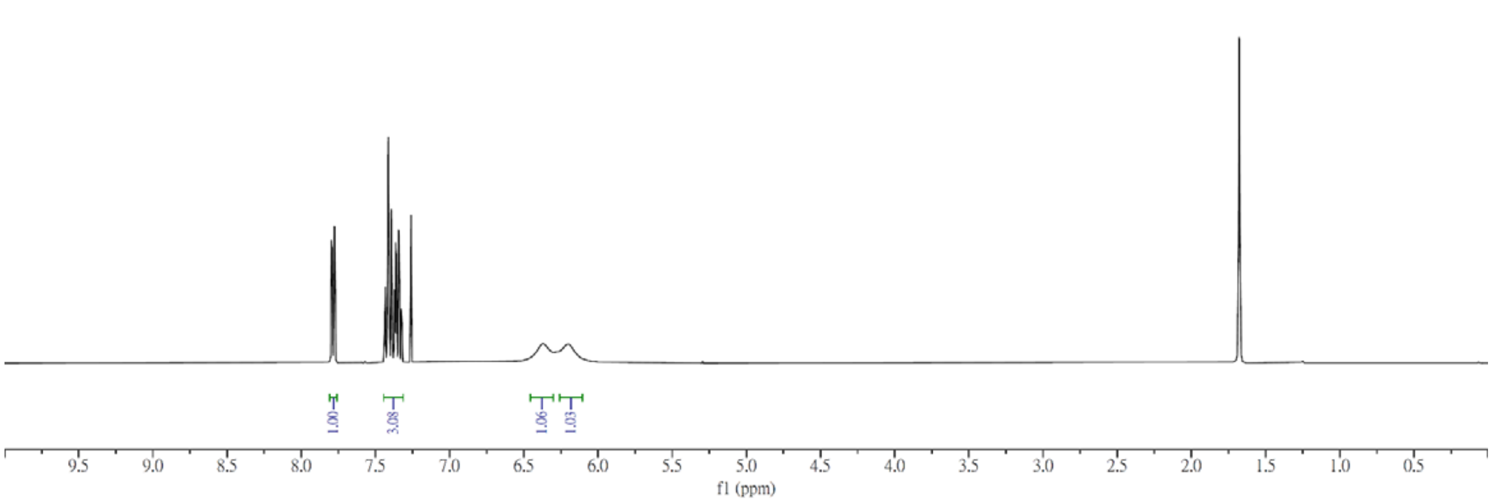

2j ^{1}H NMR spectrum (400 MHz in $CDCl_3$)

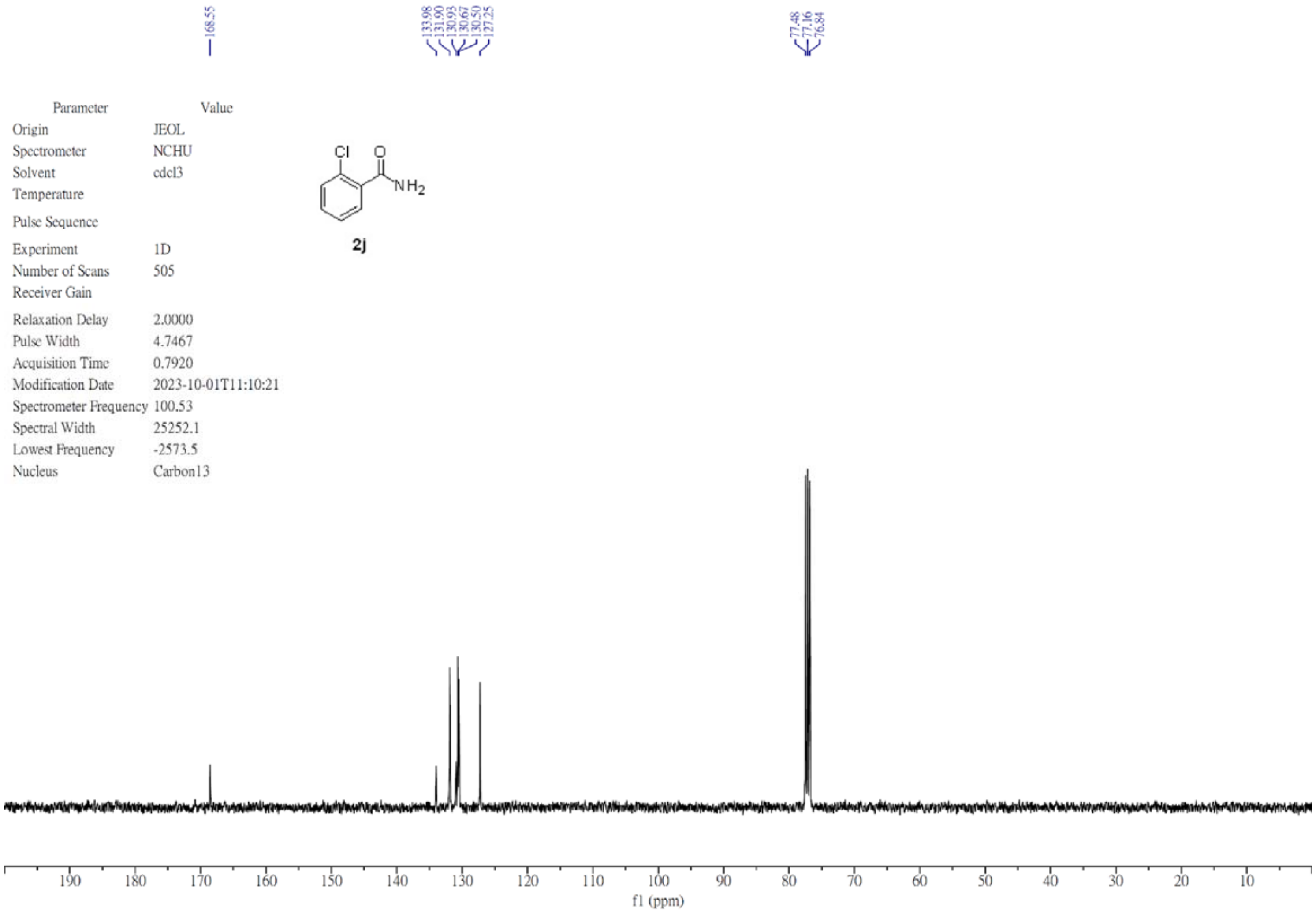

2j $^{13}C\{^{1}H\}$ NMR spectrum (100 MHz in $CDCl_3$)

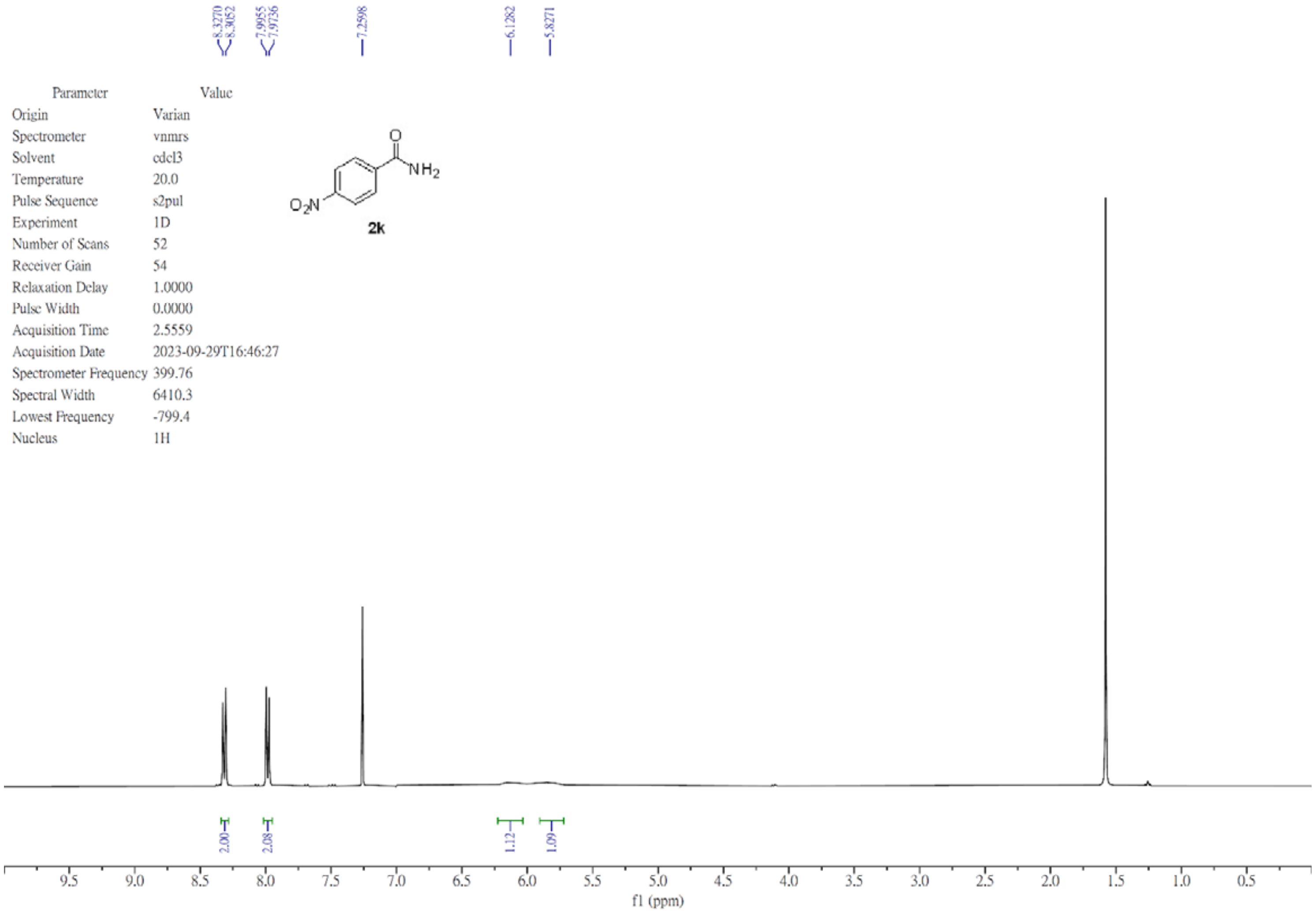

2k ^{1}H NMR spectrum (400 MHz in $CDCl_3$)

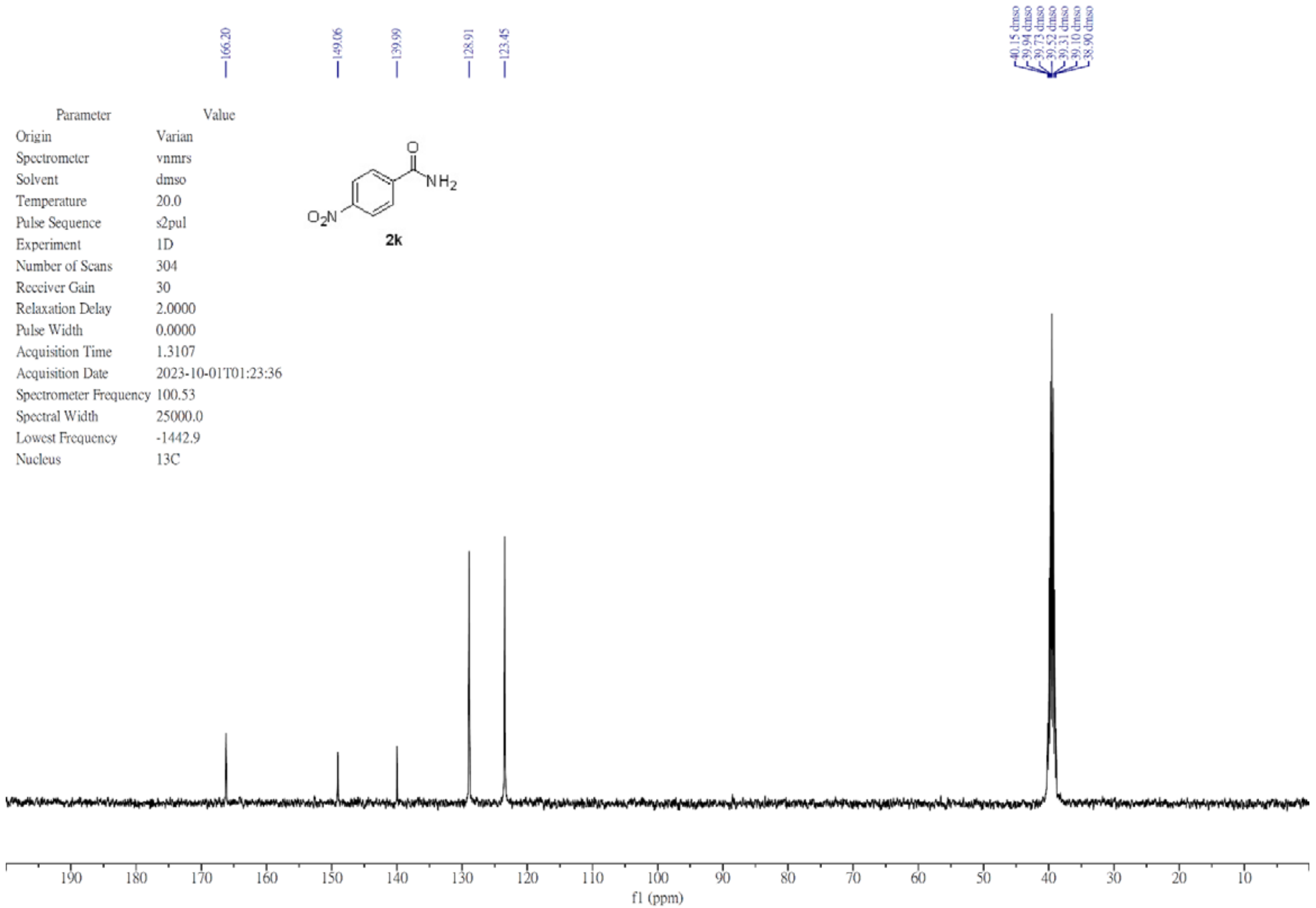

2k $^{13}C\{^{1}H\}$ NMR spectrum (100 MHz in $(CD_3)_2SO$)

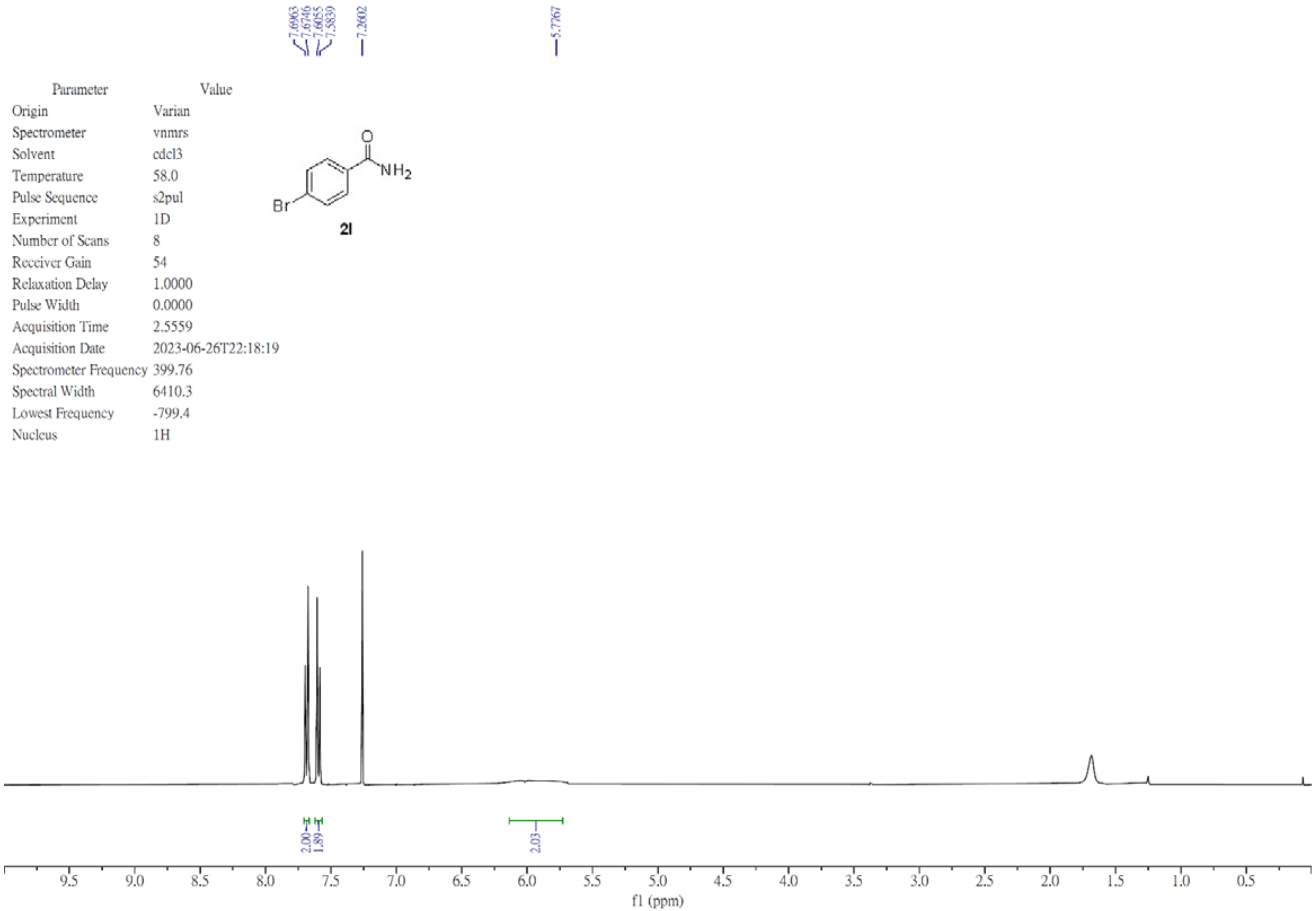

2l ^{1}H NMR spectrum (400 MHz in $CDCl_3$)

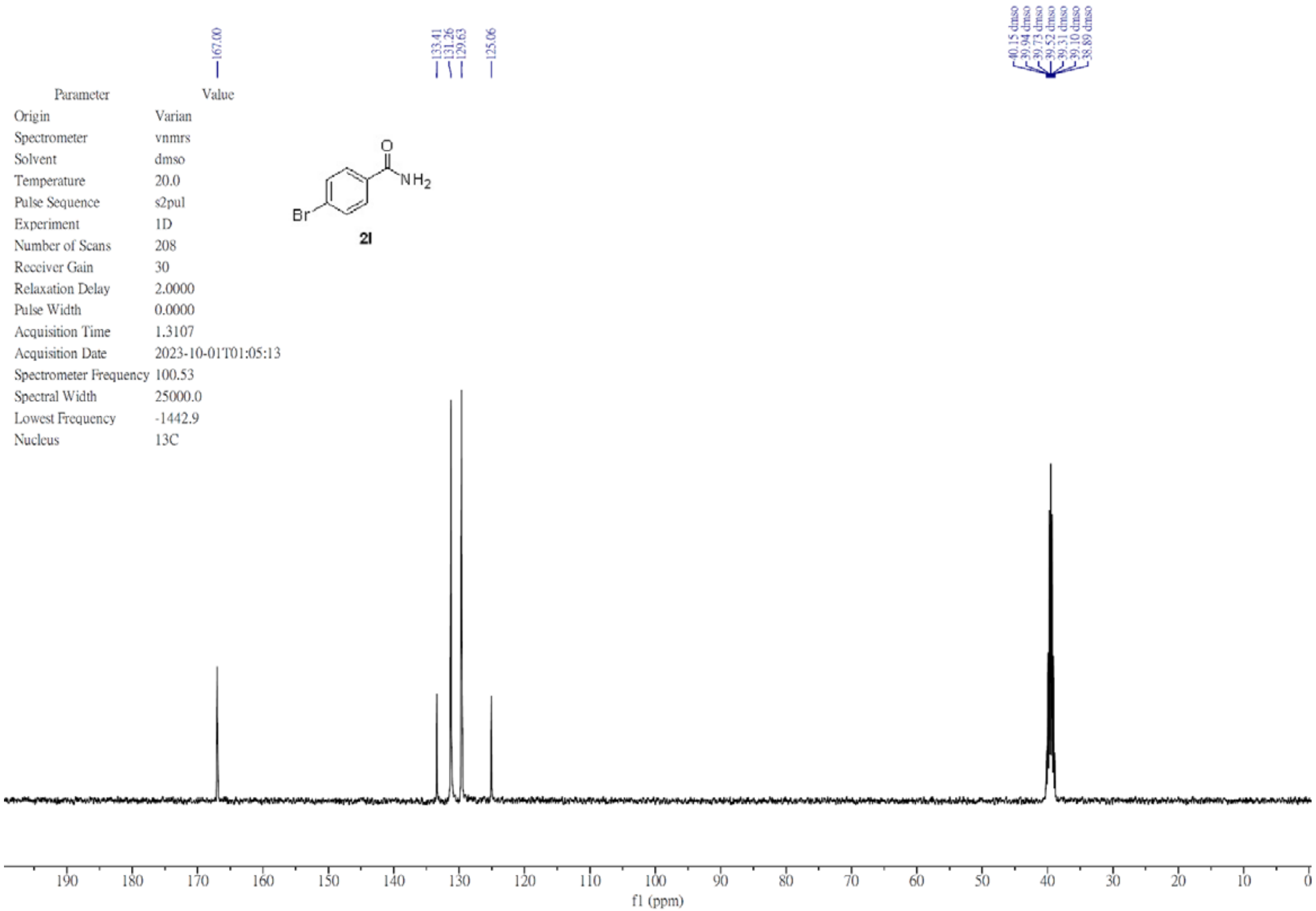

2l $^{13}C\{^{1}H\}$ NMR spectrum (100 MHz in $(CD_3)_2SO$)

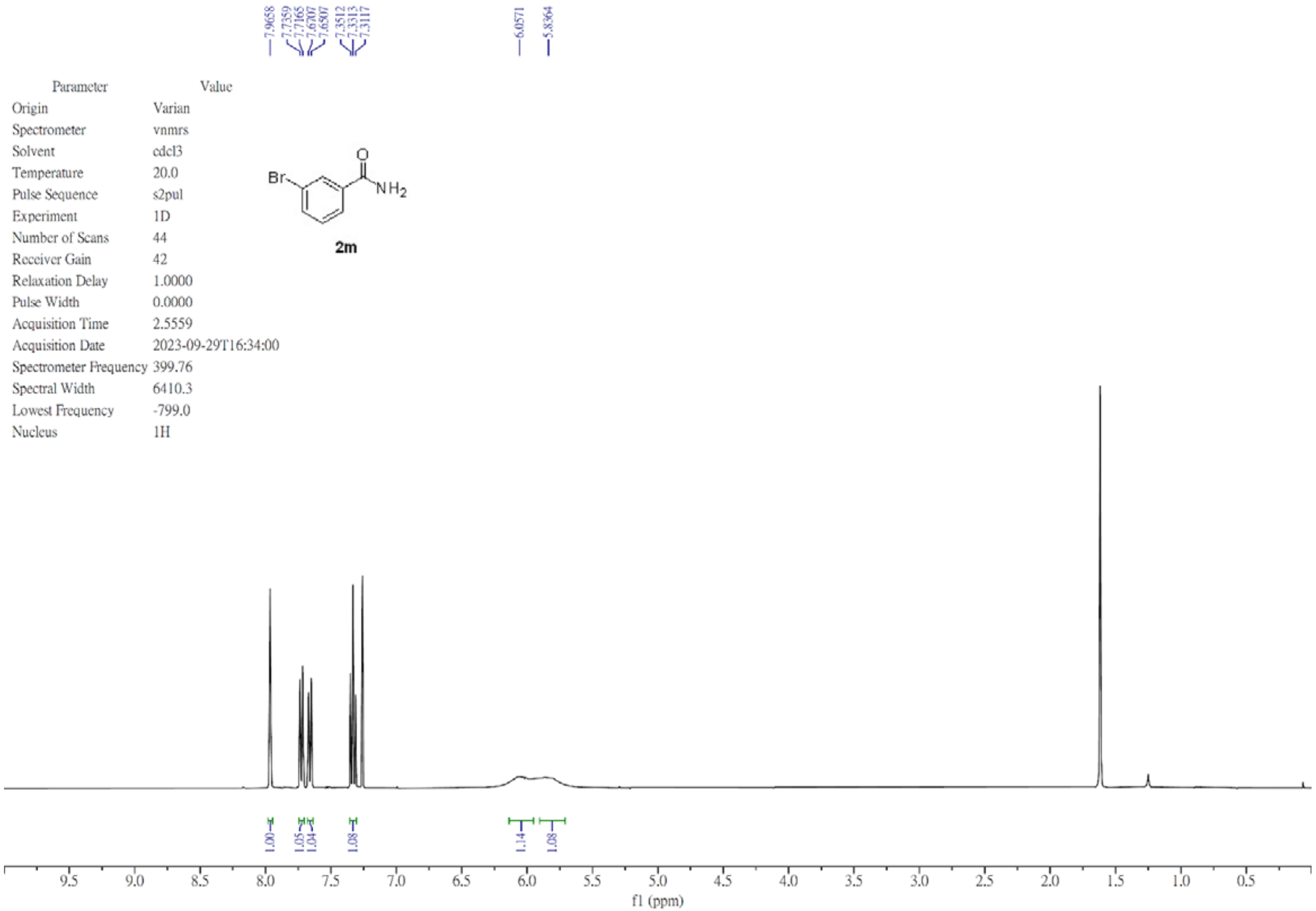

2m ^{1}H NMR spectrum (400 MHz in $CDCl_3$)

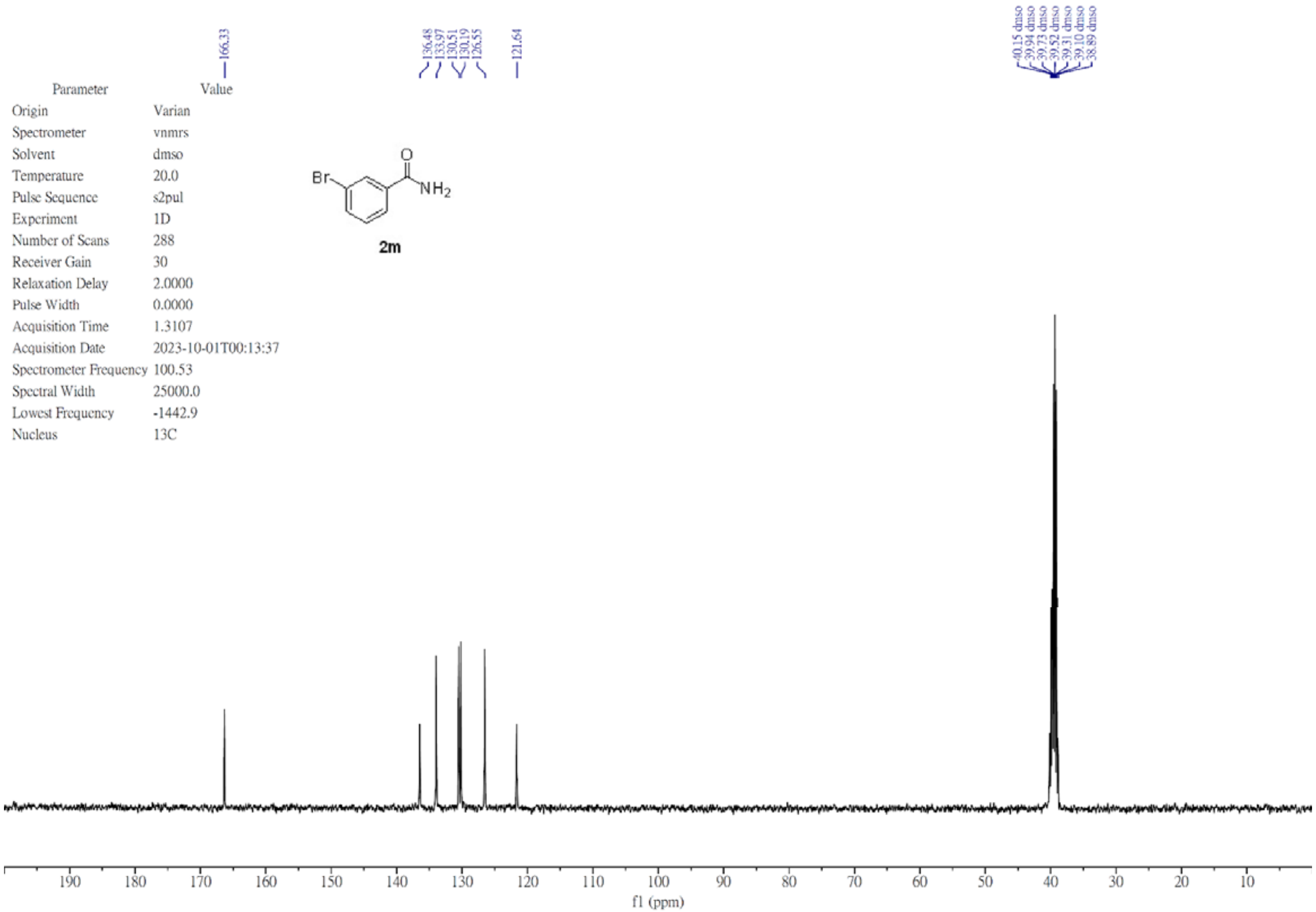

2m $^{13}C\{^{1}H\}$ NMR spectrum (100 MHz in $(CD_3)_2SO$)

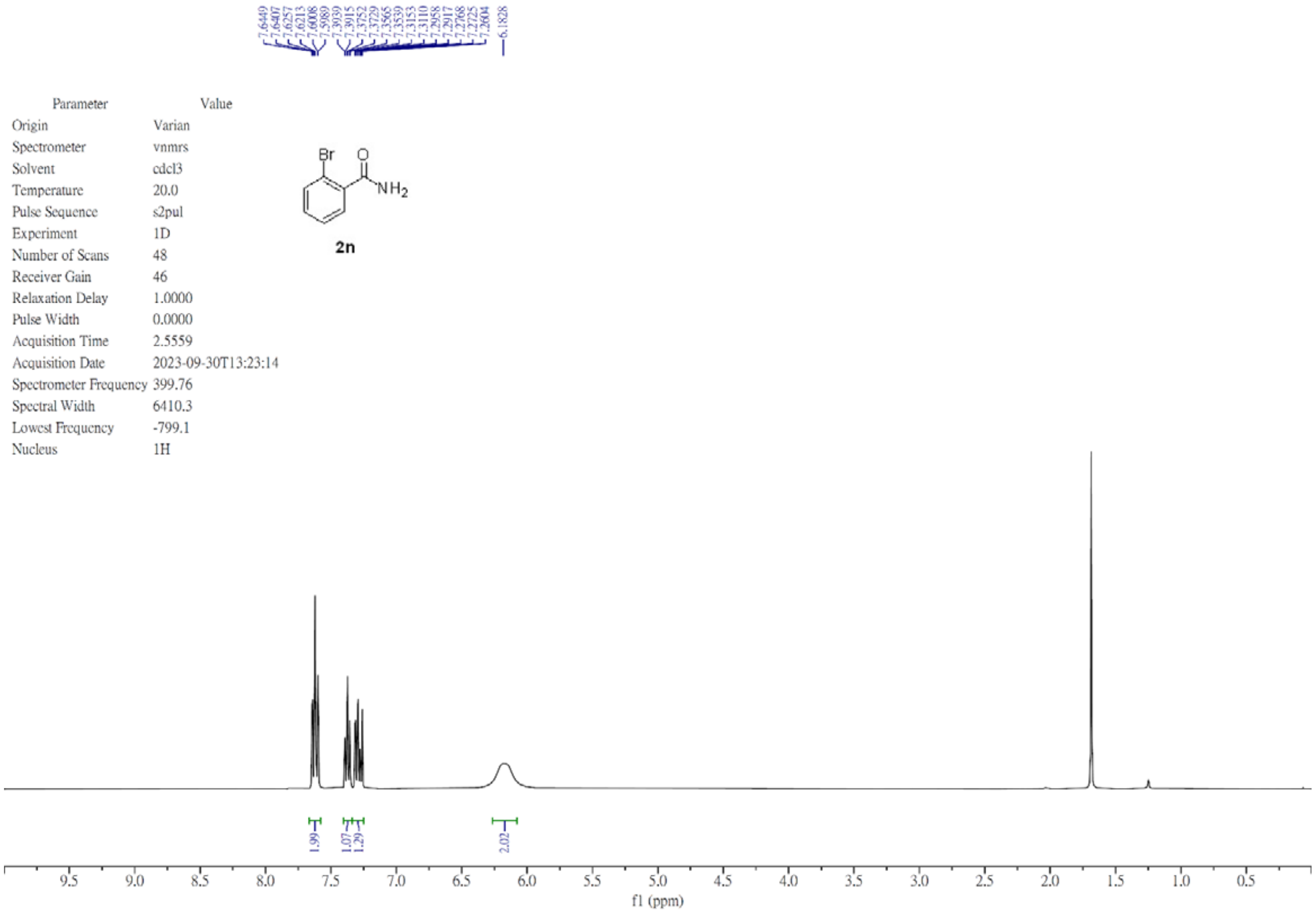

2n ^{1}H NMR spectrum (400 MHz in $CDCl_3$)

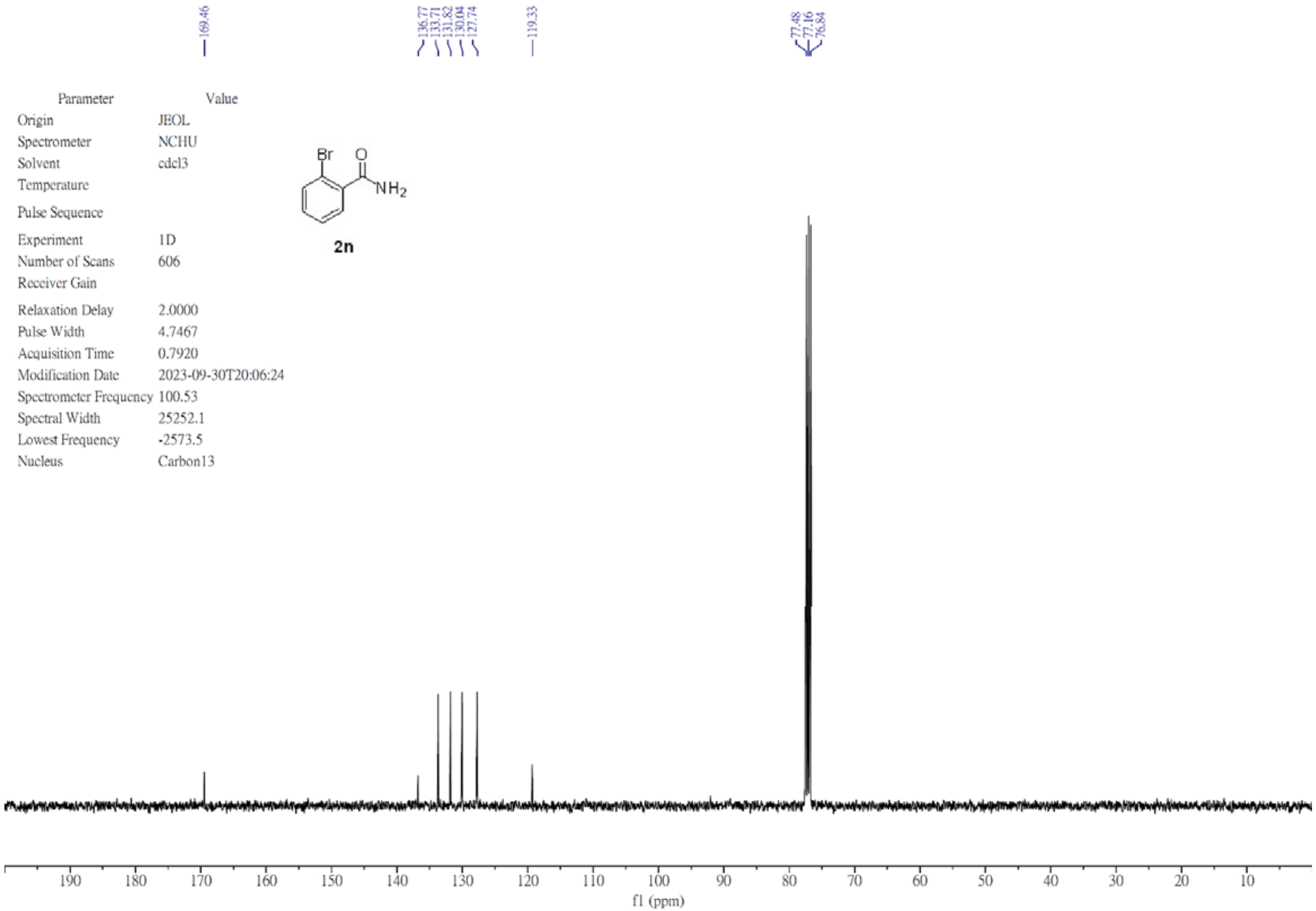

2n $^{13}C\{^{1}H\}$ NMR spectrum (100 MHz in $CDCl_3$)

Parameter	Value
Origin	Varian
Spectrometer	vnmrs
Solvent	cdcl3
Temperature	20.0
Pulse Sequence	s2pul
Experiment	1D
Number of Scans	40
Receiver Gain	52
Relaxation Delay	1.0000
Pulse Width	0.0000
Acquisition Time	2.5559
Acquisition Date	2023-10-01T05:49:31
Spectrometer Frequency	399.76
Spectral Width	6410.3
Lowest Frequency	-799.2
Nucleus	1H

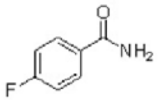

2o

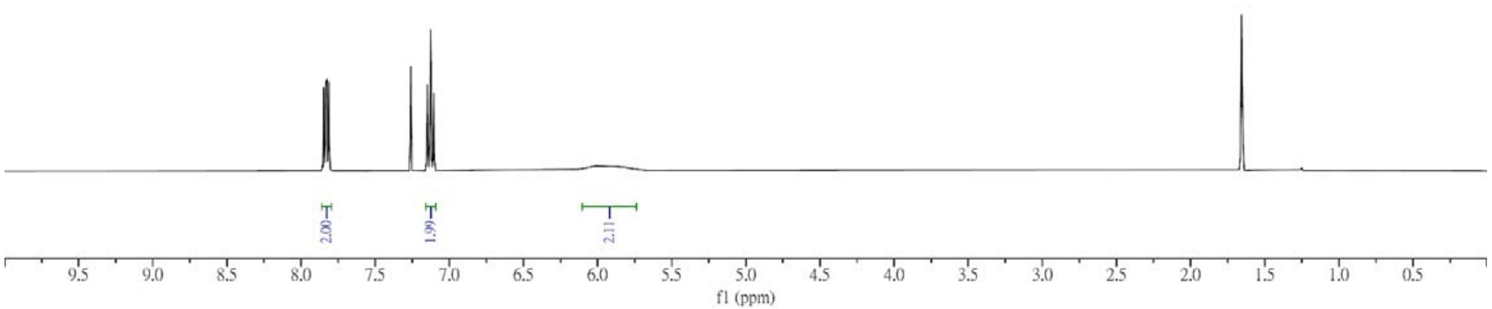

2o ^{1}H NMR spectrum (400 MHz in $CDCl_3$)

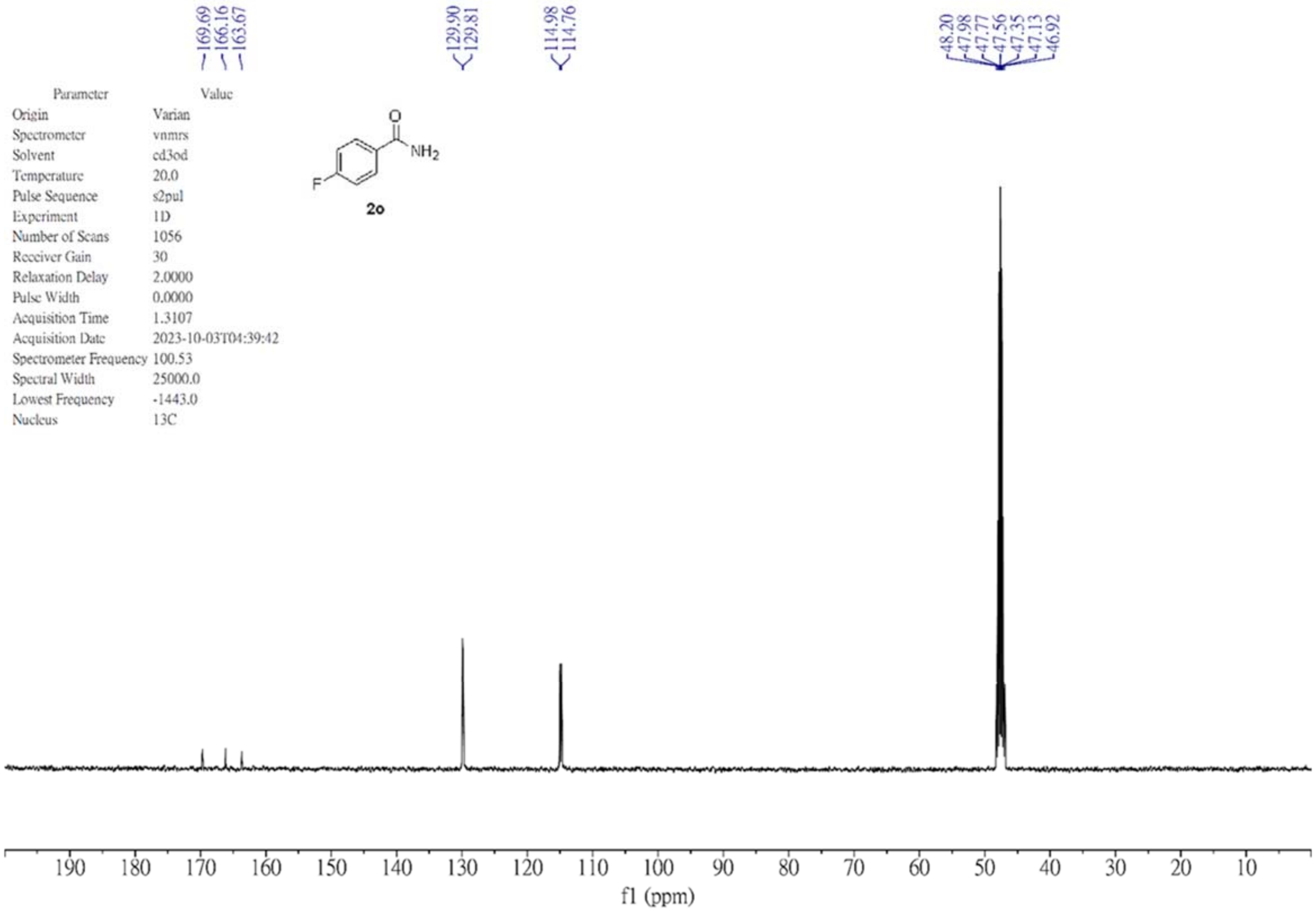

2o $^{13}C\{^{1}H\}$ NMR spectrum (100 MHz in CD_3OD)

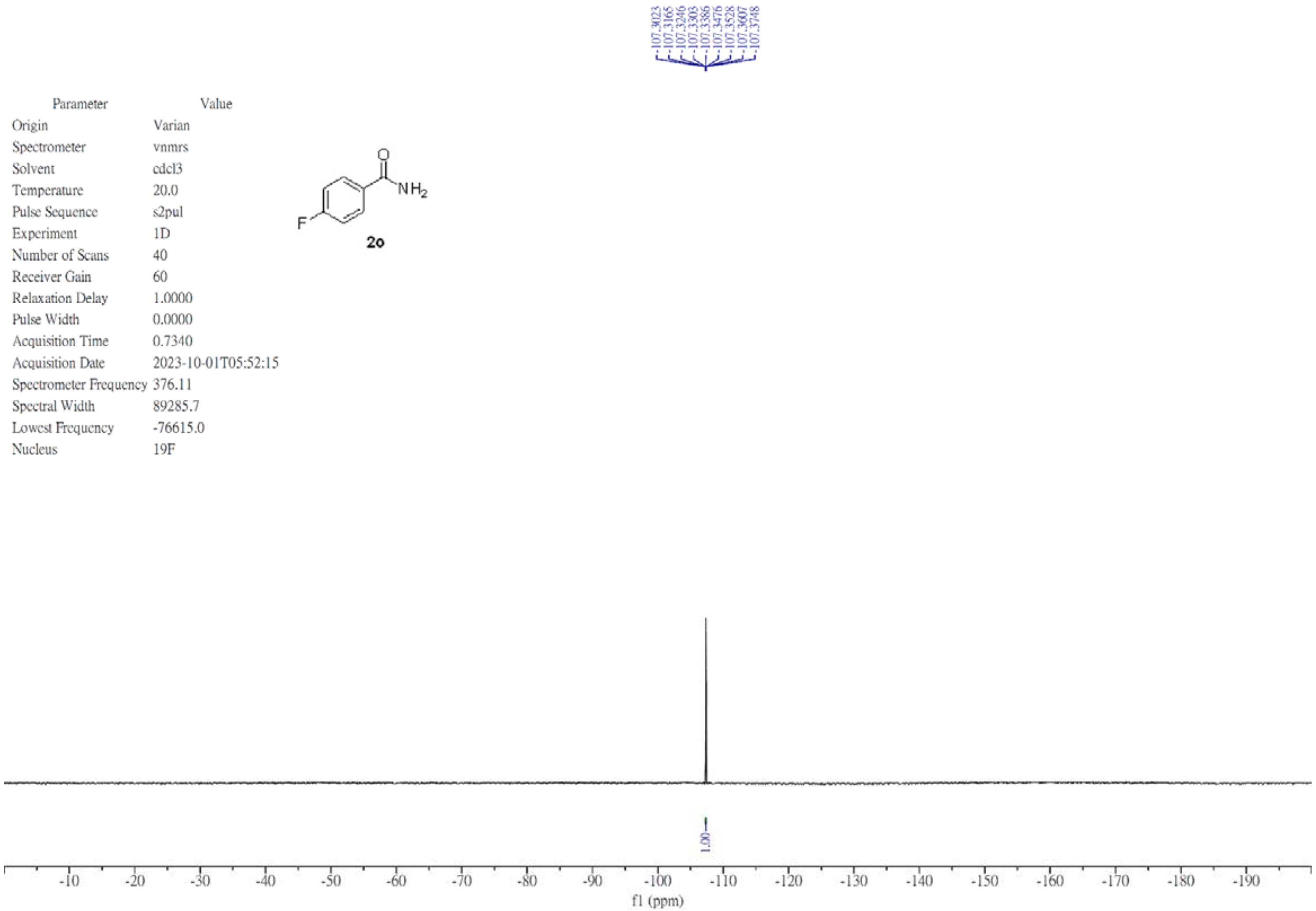

Parameter	Value
Origin	Varian
Spectrometer	vnmrs
Solvent	cdcl3
Temperature	20.0
Pulse Sequence	s2pul
Experiment	1D
Number of Scans	40
Receiver Gain	60
Relaxation Delay	1.0000
Pulse Width	0.0000
Acquisition Time	0.7340
Acquisition Date	2023-10-01T05:52:15
Spectrometer Frequency	376.11
Spectral Width	89285.7
Lowest Frequency	-76615.0
Nucleus	19F

2o ^{19}F NMR spectrum (376 MHz, $CDCl_3$)

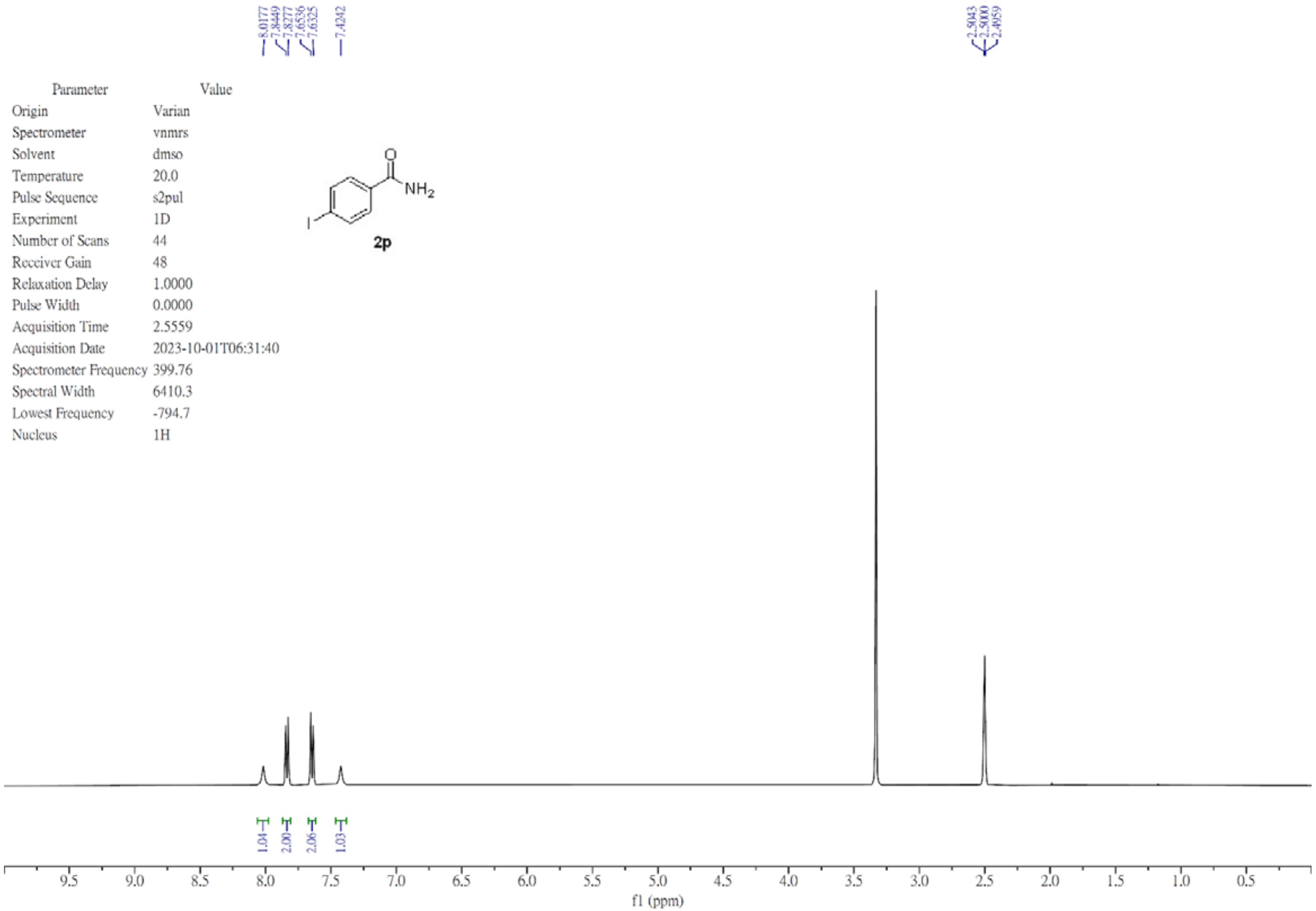

2p ^{1}H NMR spectrum (400 MHz in $CDCl_3$)

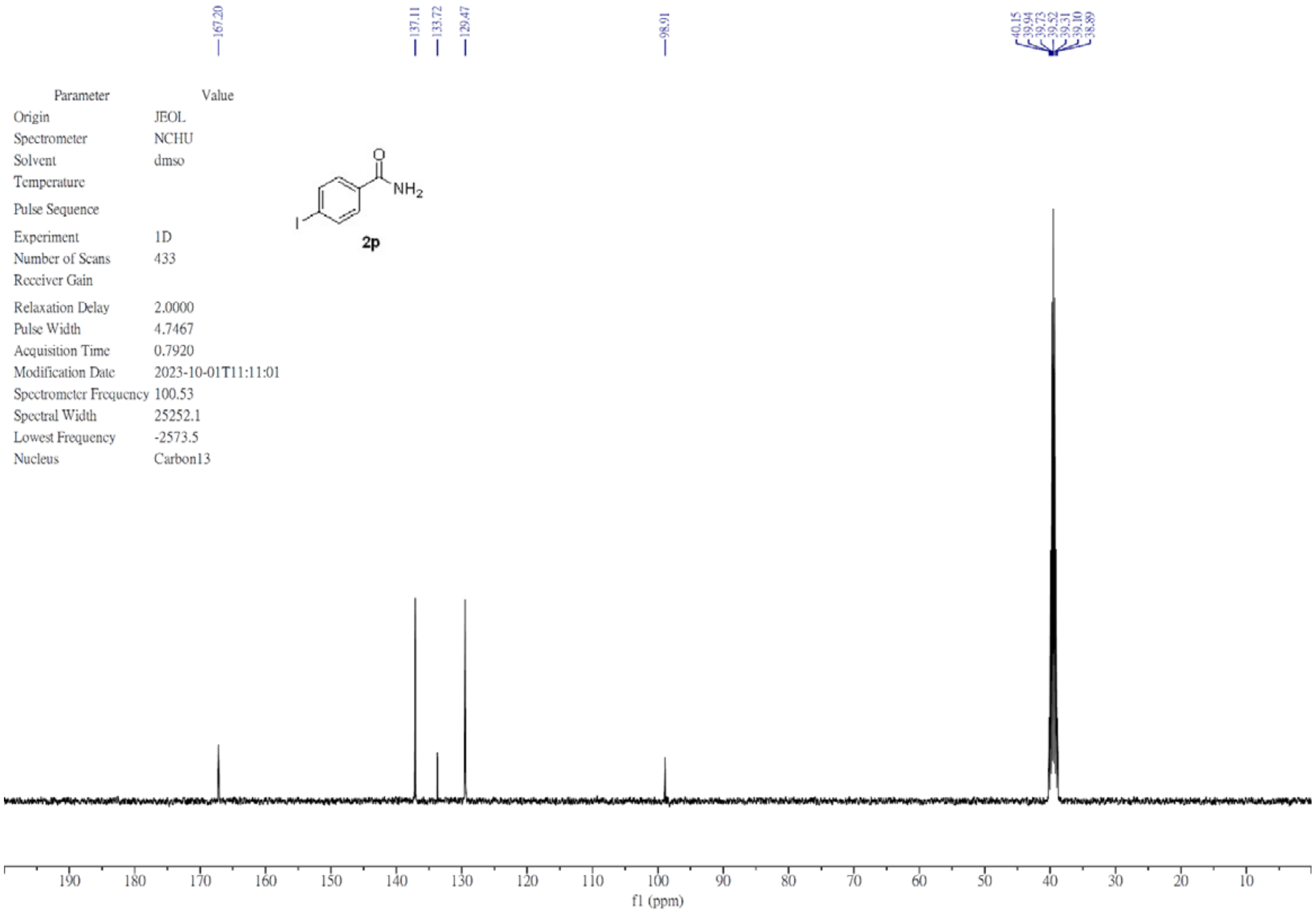

2p $^{13}C\{^{1}H\}$ NMR spectrum (100 MHz in $(CD_3)_2SO$)

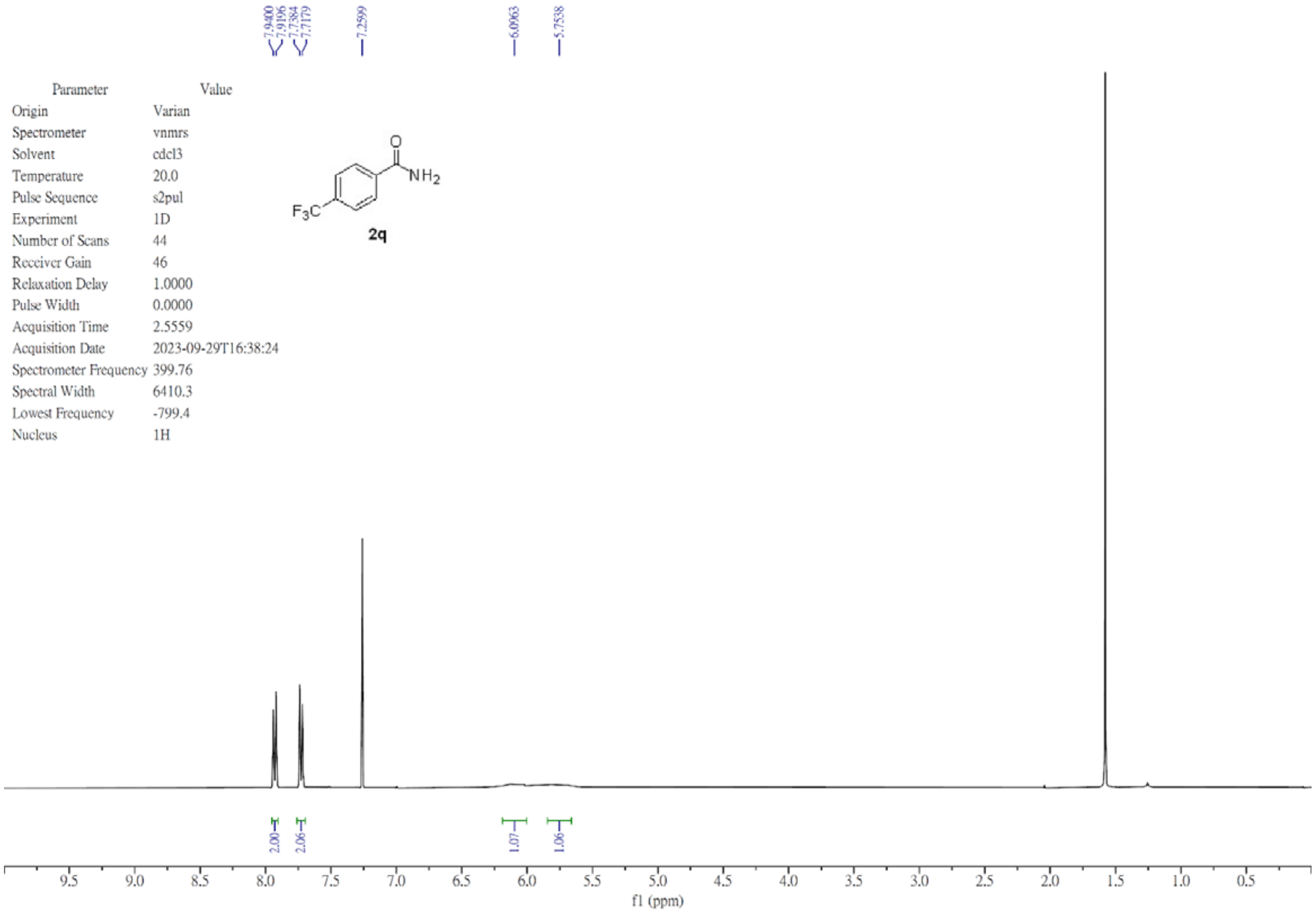

2q ^{1}H NMR spectrum (400 MHz in $CDCl_3$)

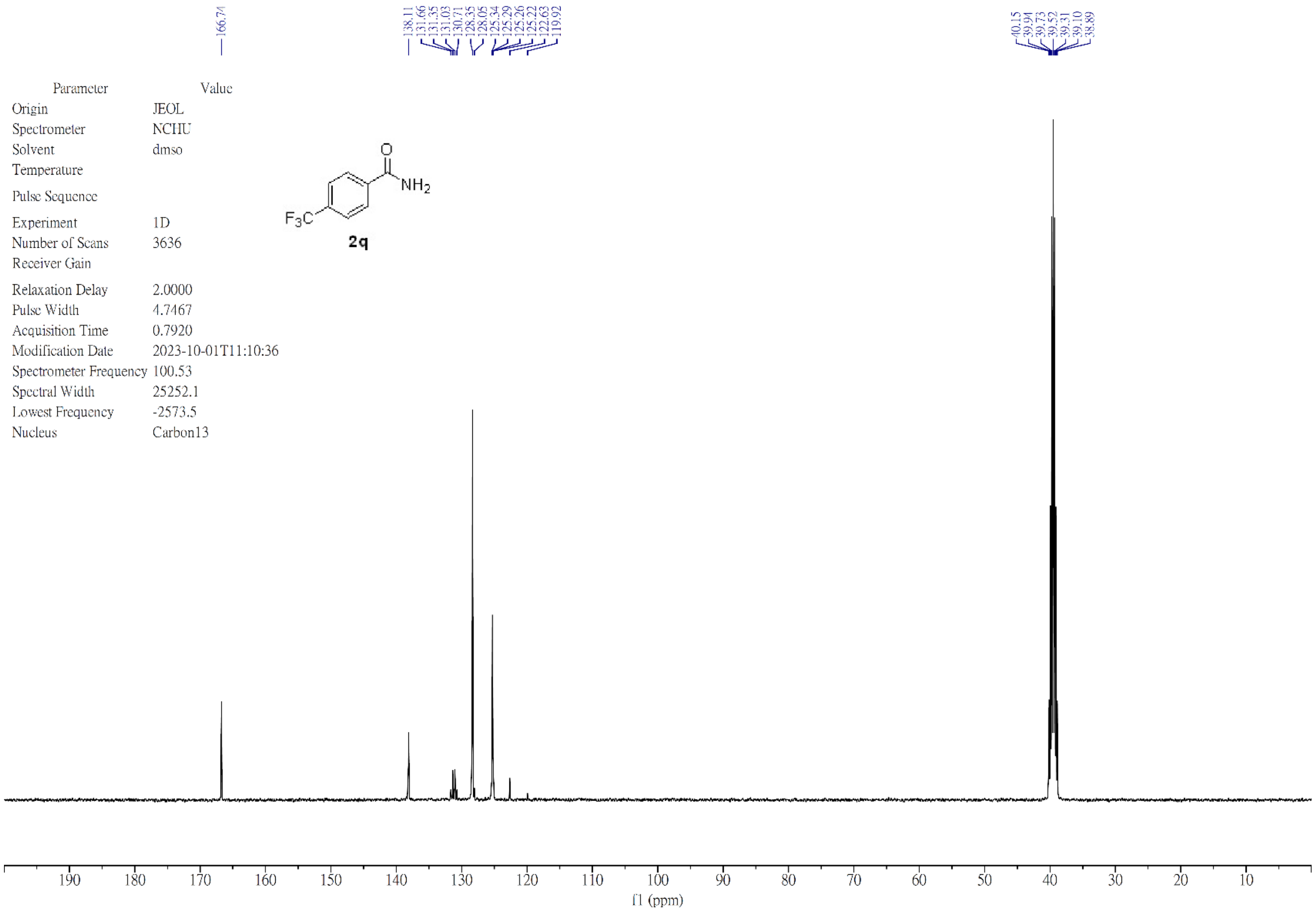

2q $^{13}C\{^{1}H\}$ NMR spectrum (100 MHz in $(CD_3)_2SO$)

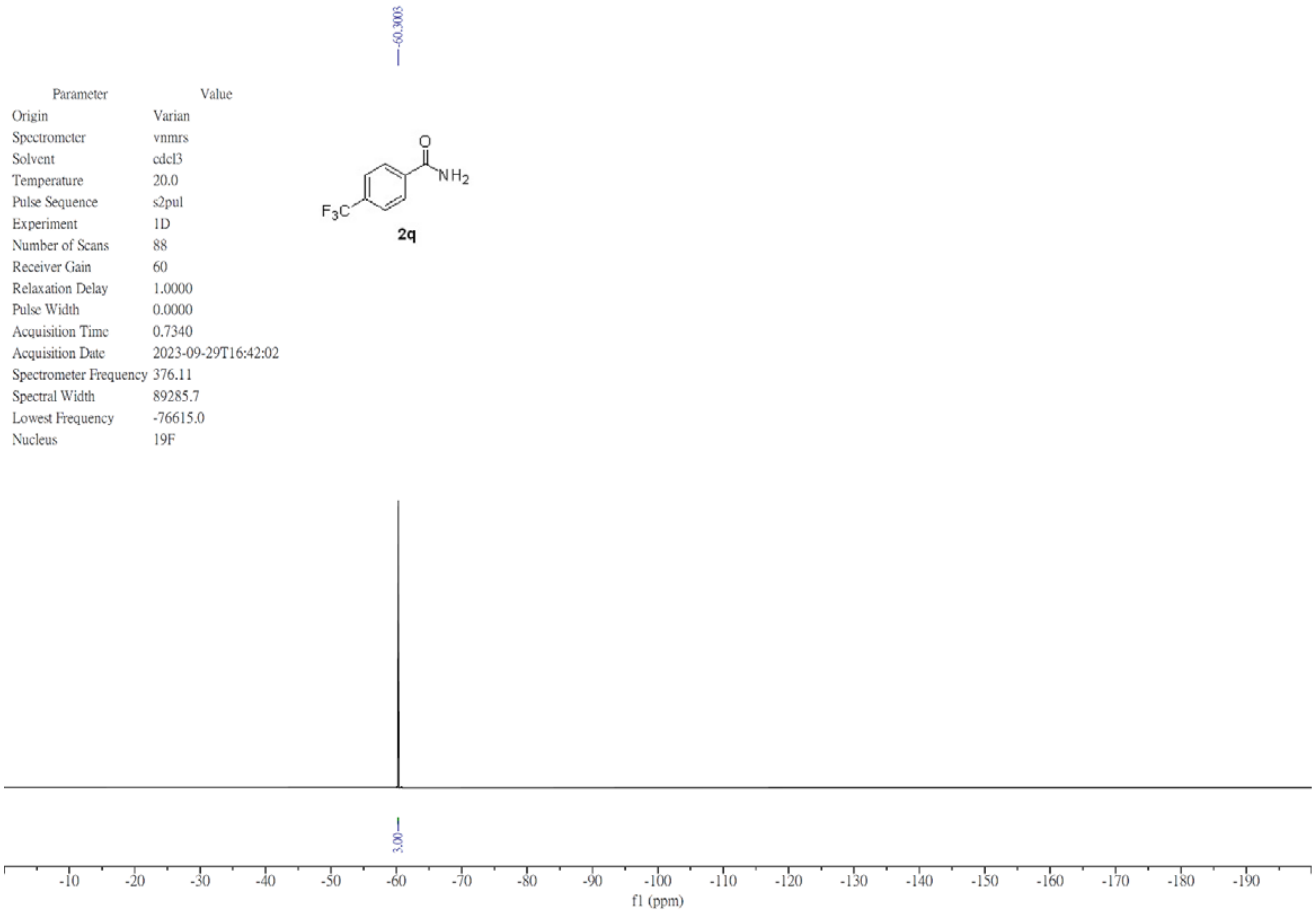

2q ^{19}F NMR spectrum (376 MHz, $CDCl_3$)

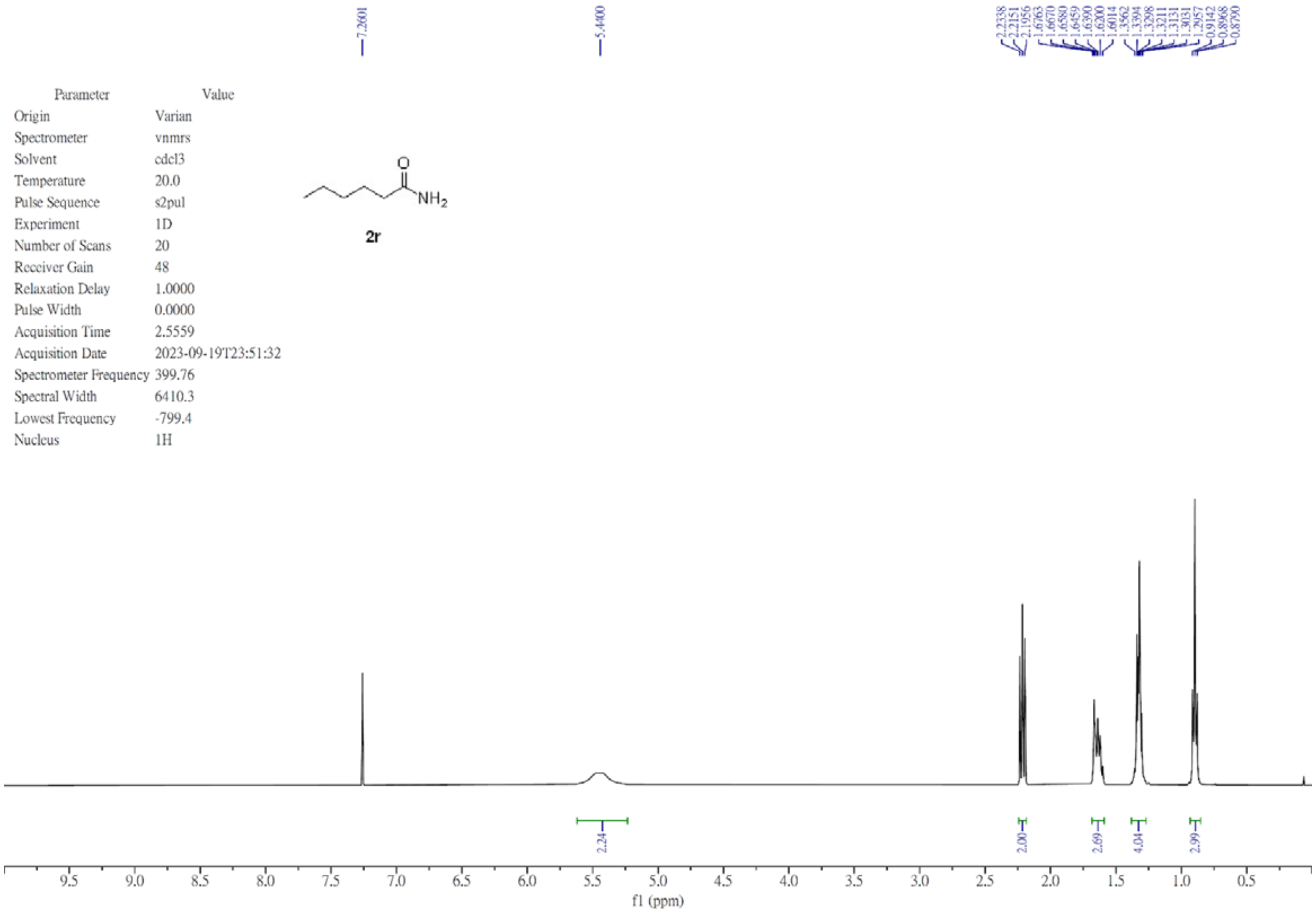

2r ^{1}H NMR spectrum (400 MHz in $CDCl_3$)

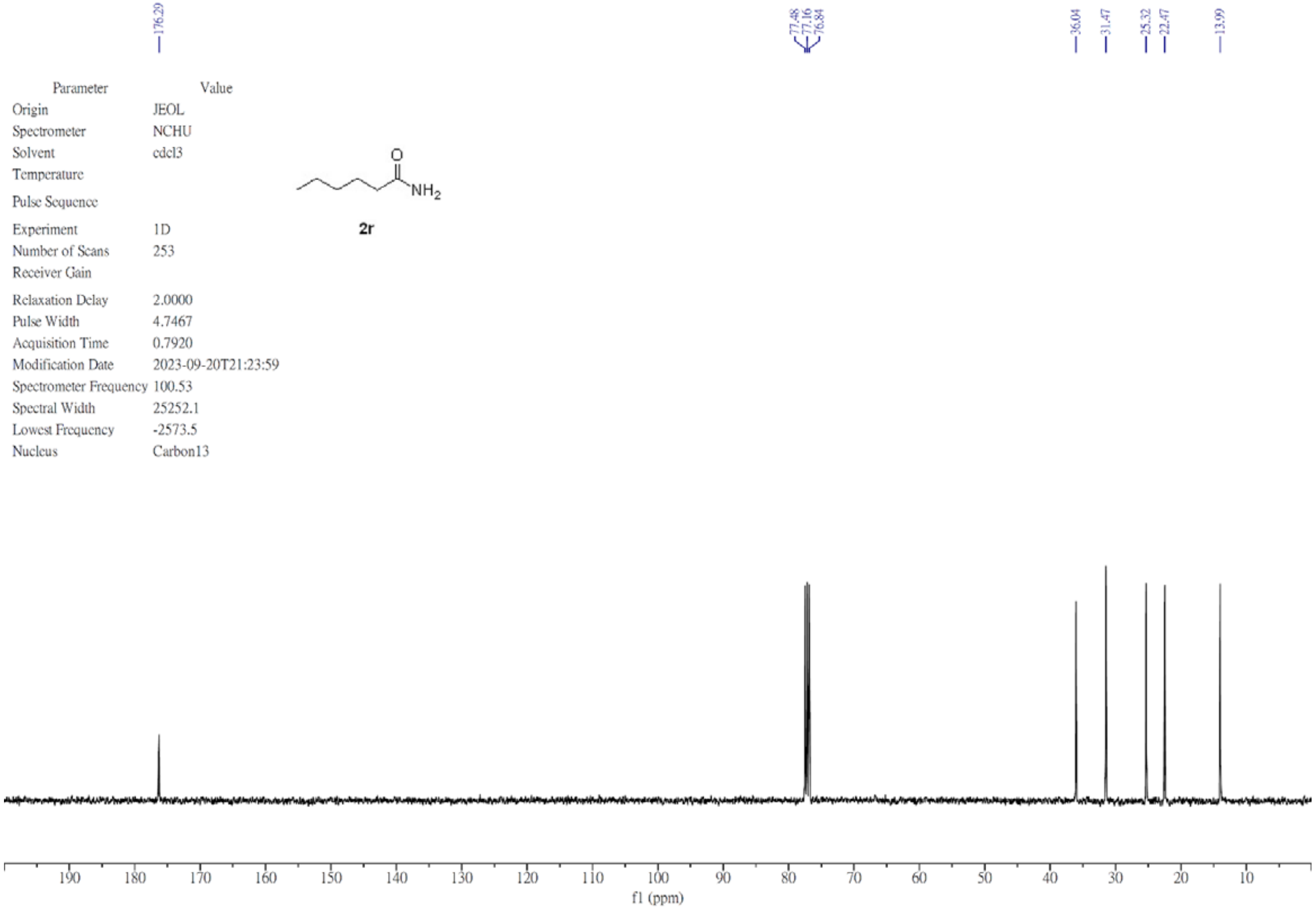

2r $^{13}C\{^{1}H\}$ NMR spectrum (100 MHz in $CDCl_3$)

Parameter	Value
Origin	Varian
Spectrometer	vnmrs
Solvent	cdcl3
Temperature	20.0
Pulse Sequence	s2pul
Experiment	1D
Number of Scans	52
Receiver Gain	42
Relaxation Delay	1.0000
Pulse Width	0.0000
Acquisition Time	2.5559
Acquisition Date	2023-09-22T00:48:11
Spectrometer Frequency	399.76
Spectral Width	6410.3
Lowest Frequency	-799.2
Nucleus	1H

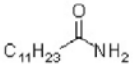

2s

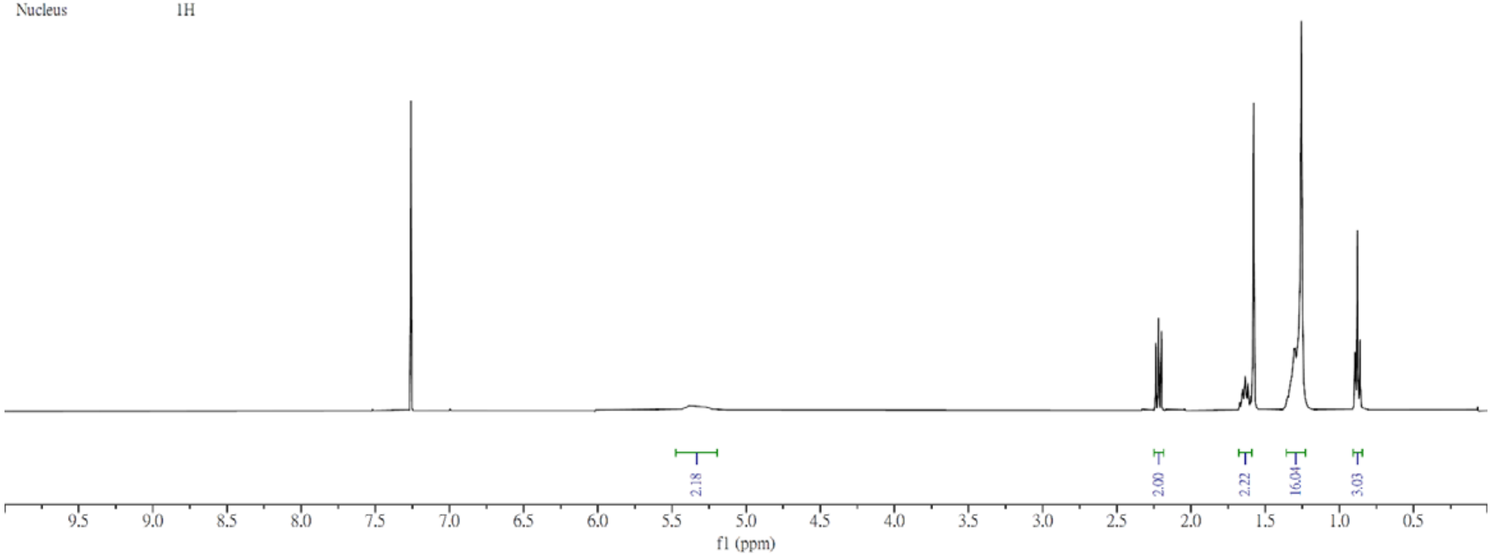

2s ^{1}H NMR spectrum (400 MHz in $CDCl_3$)

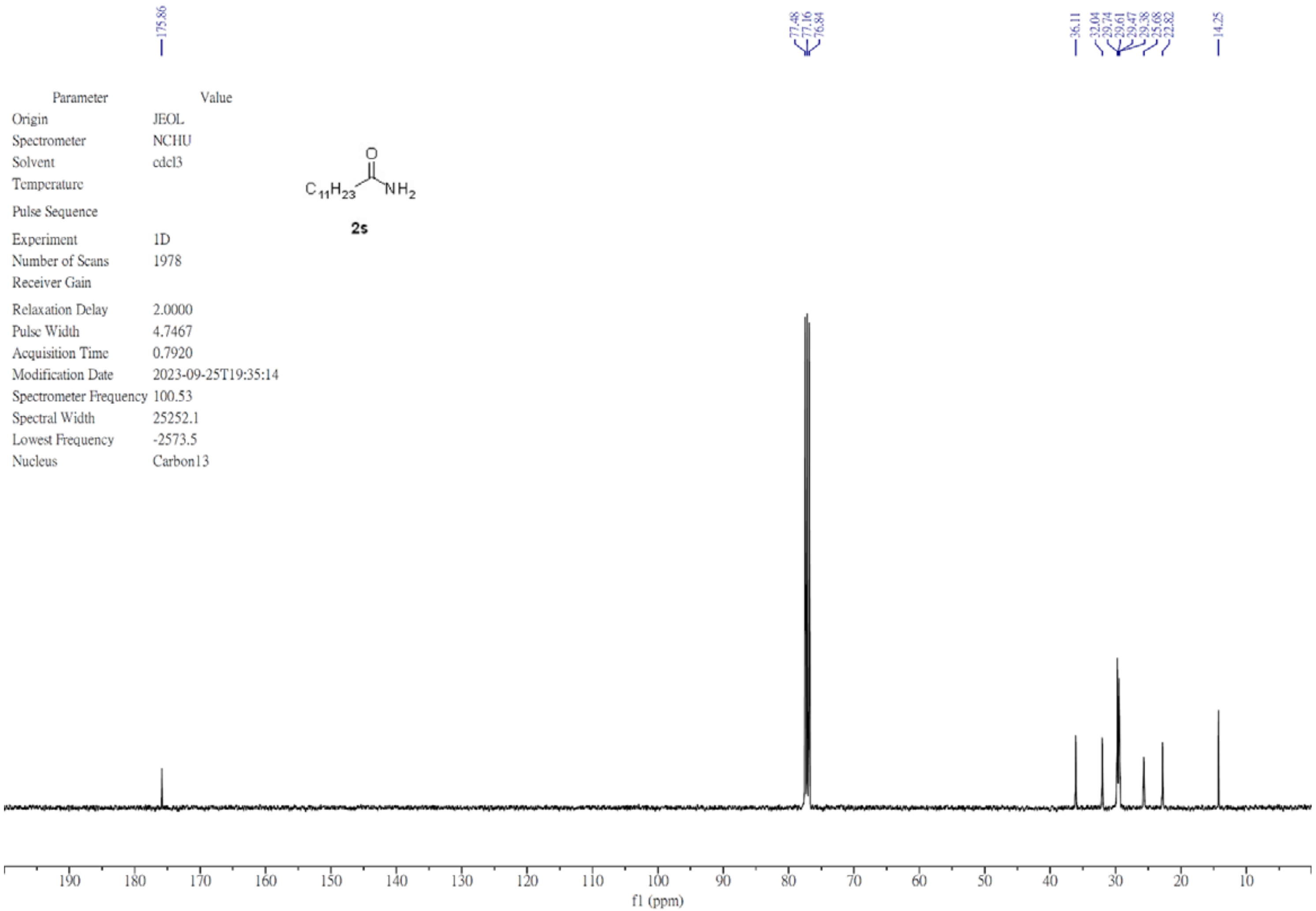

2s $^{13}C\{^{1}H\}$ NMR spectrum (100 MHz in $CDCl_3$)

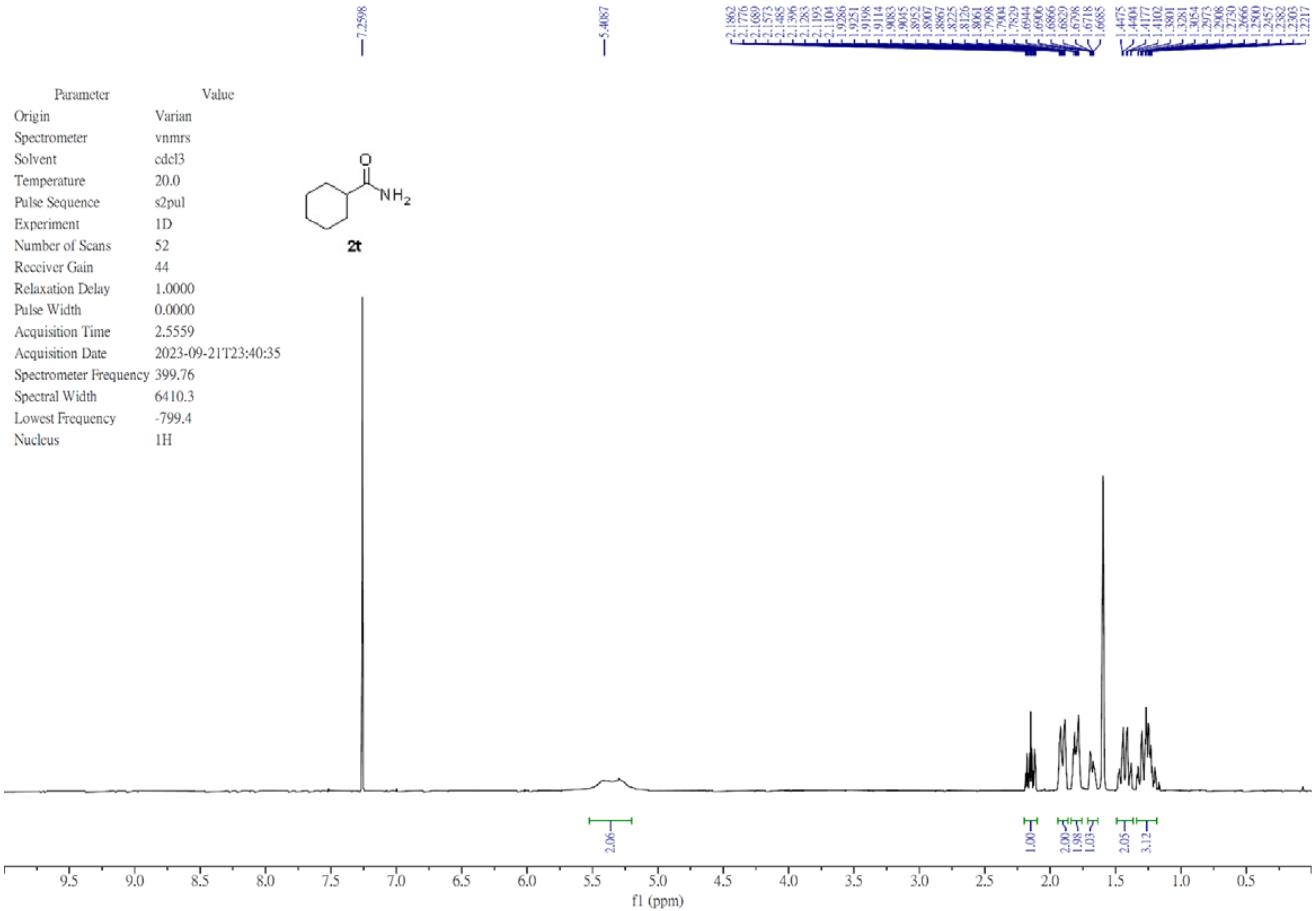

2t ^{1}H NMR spectrum (400 MHz in $CDCl_3$)

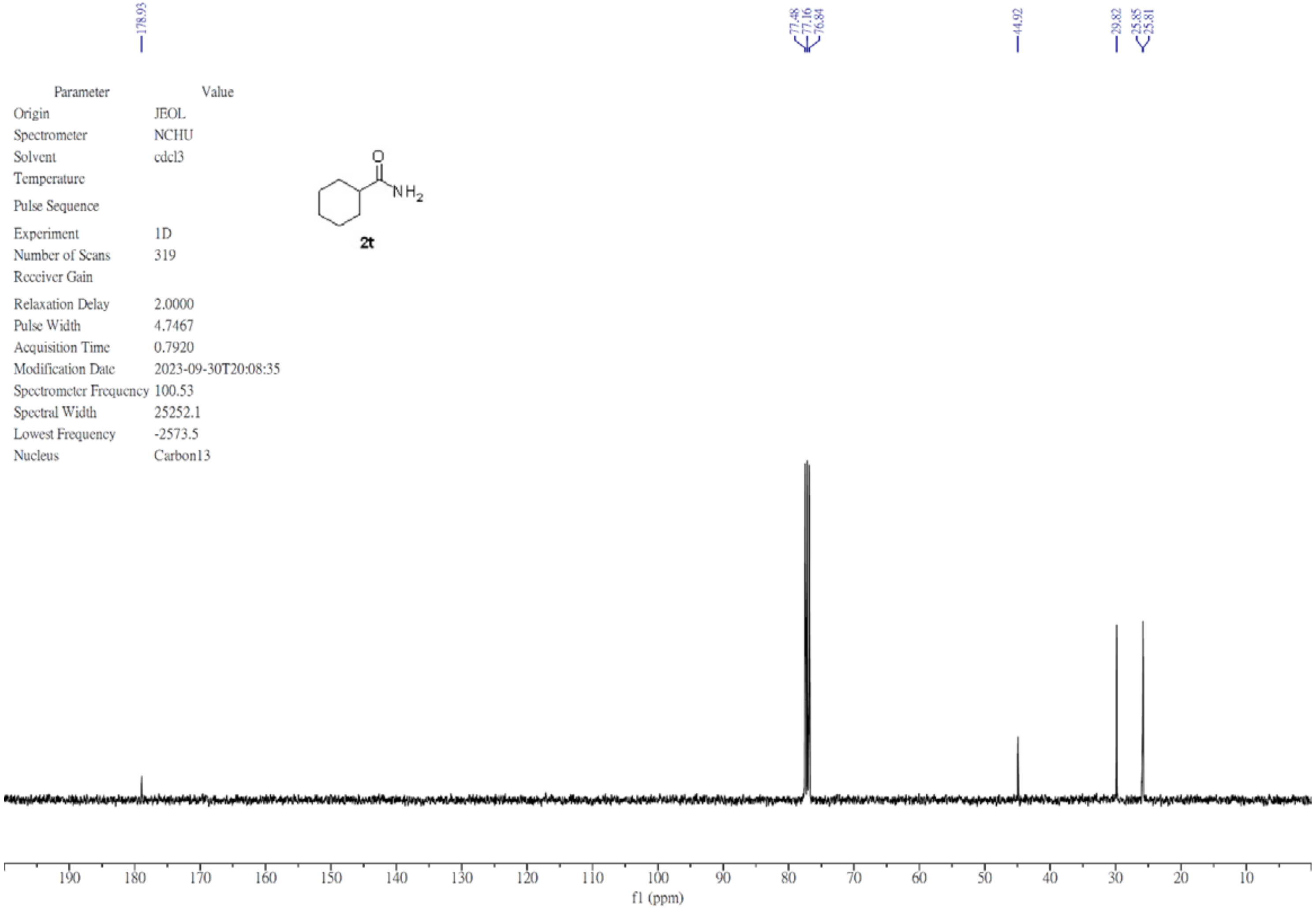

2t $^{13}C\{^{1}H\}$ NMR spectrum (100 MHz in $CDCl_3$)

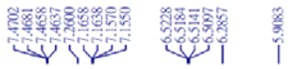

Parameter	Value
Origin	Varian
Spectrometer	vnmrs
Solvent	cdcl3
Temperature	20.0
Pulse Sequence	s2pul
Experiment	1D
Number of Scans	48
Receiver Gain	50
Relaxation Delay	1.0000
Pulse Width	0.0000
Acquisition Time	2.5559
Acquisition Date	2023-10-02T00:43:47
Spectrometer Frequency	399.76
Spectral Width	6410.3
Lowest Frequency	-799.2
Nucleus	1H

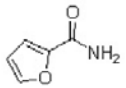

2u

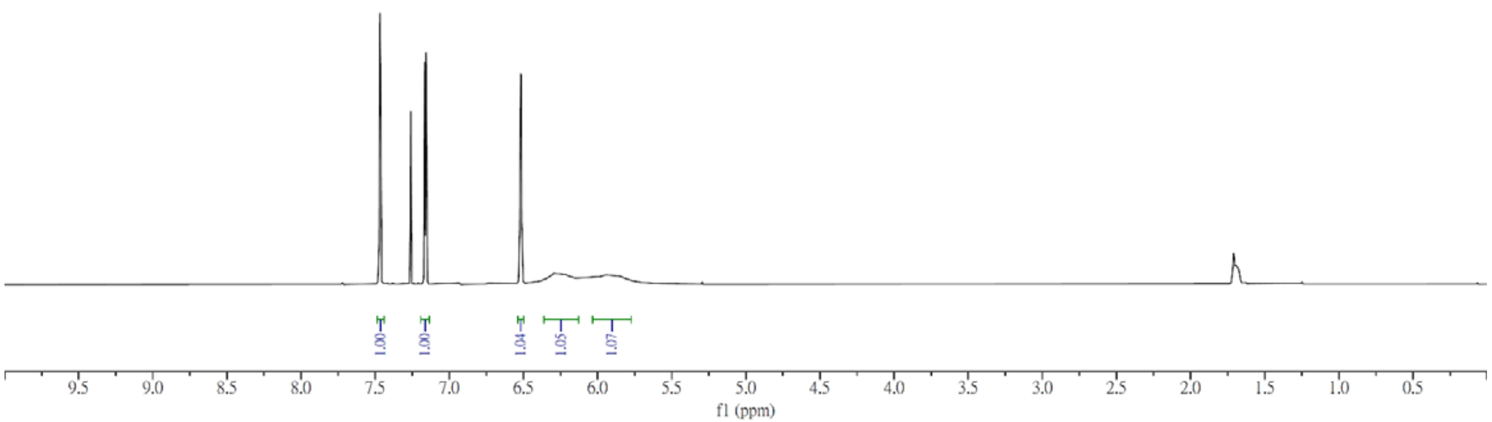

2u ^{1}H NMR spectrum (400 MHz in $CDCl_3$)

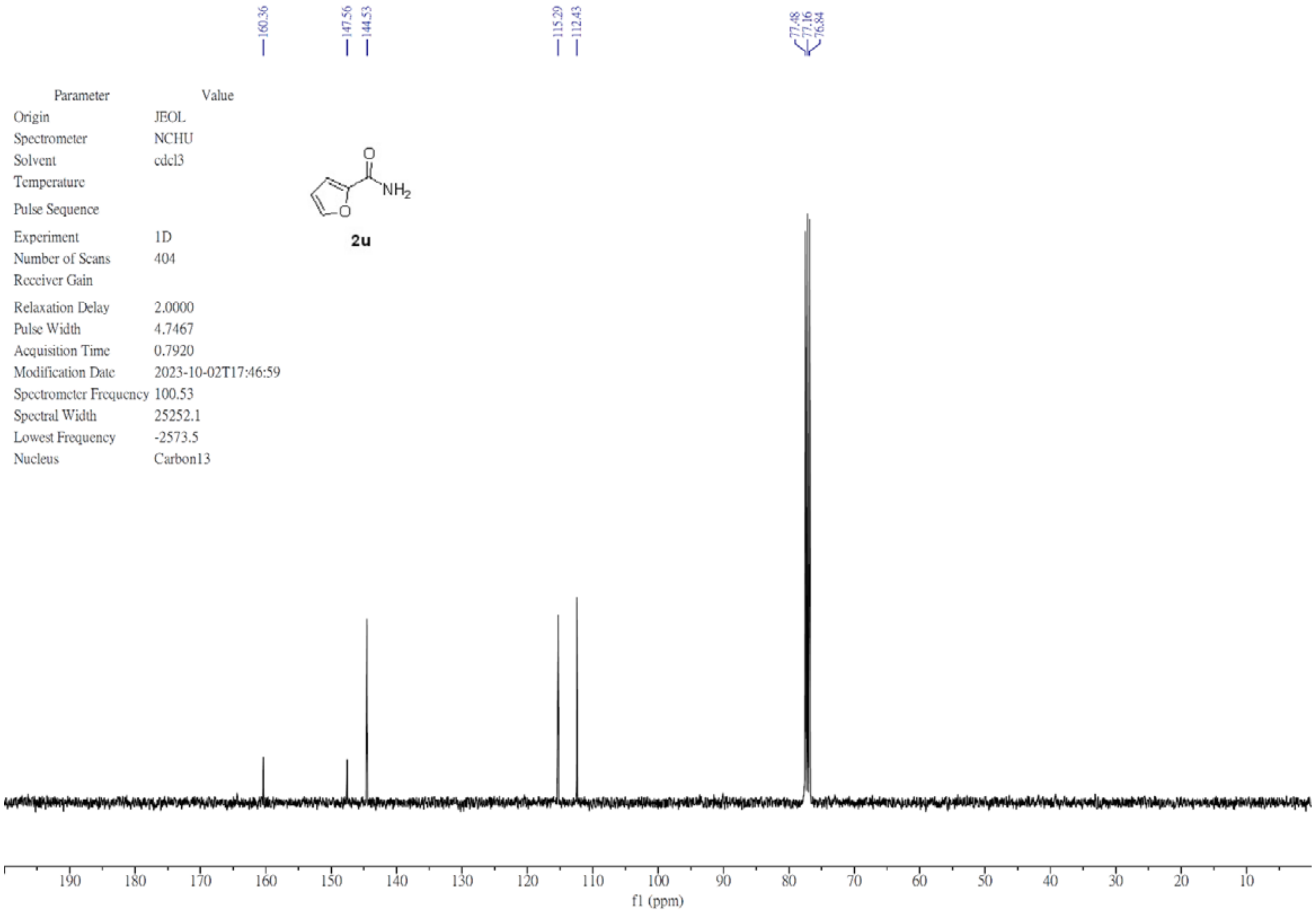

2u $^{13}C\{^{1}H\}$ NMR spectrum (100 MHz in $CDCl_3$)

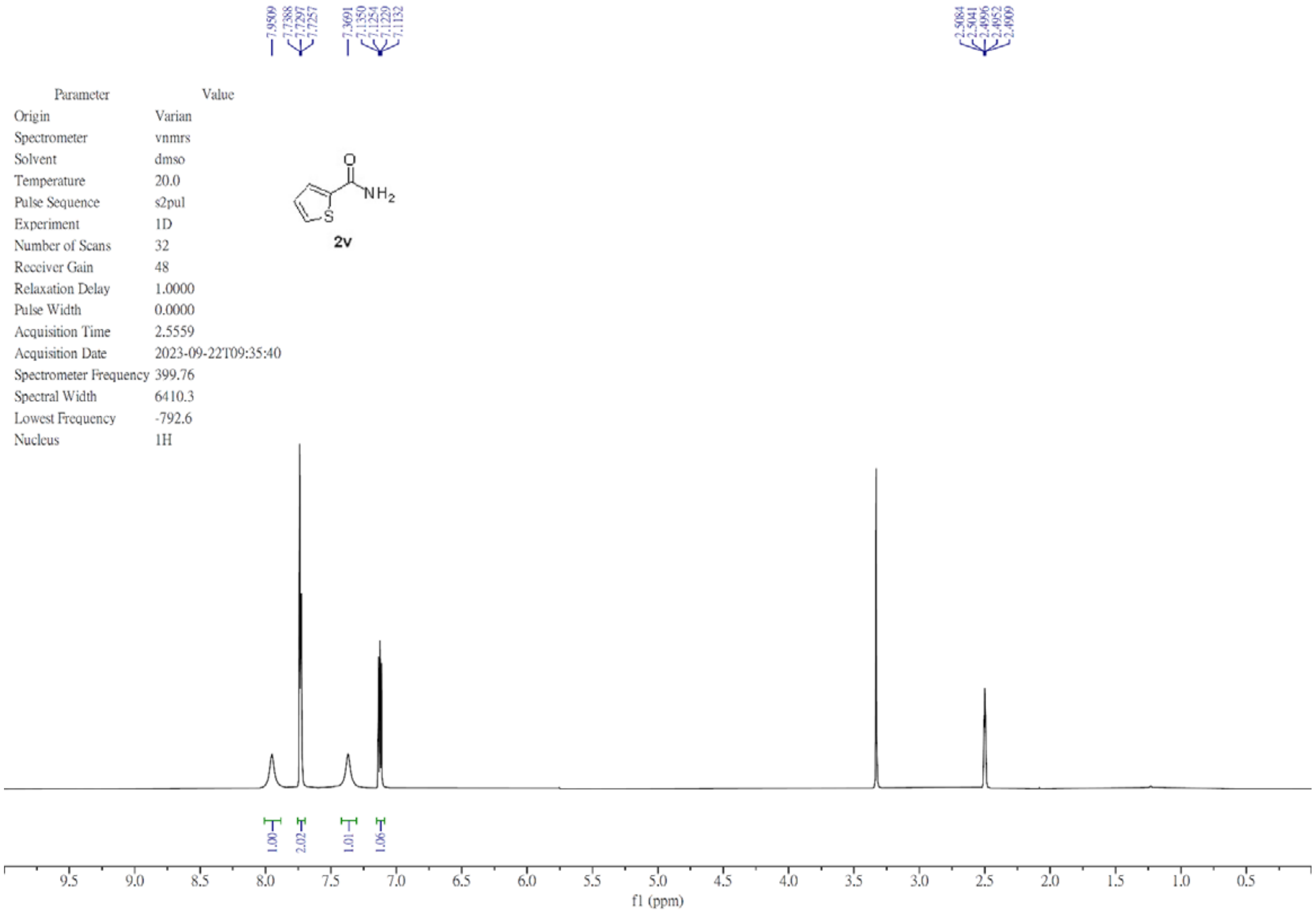

2v ^{1}H NMR spectrum (400 MHz in $(CD_3)_2SO$)

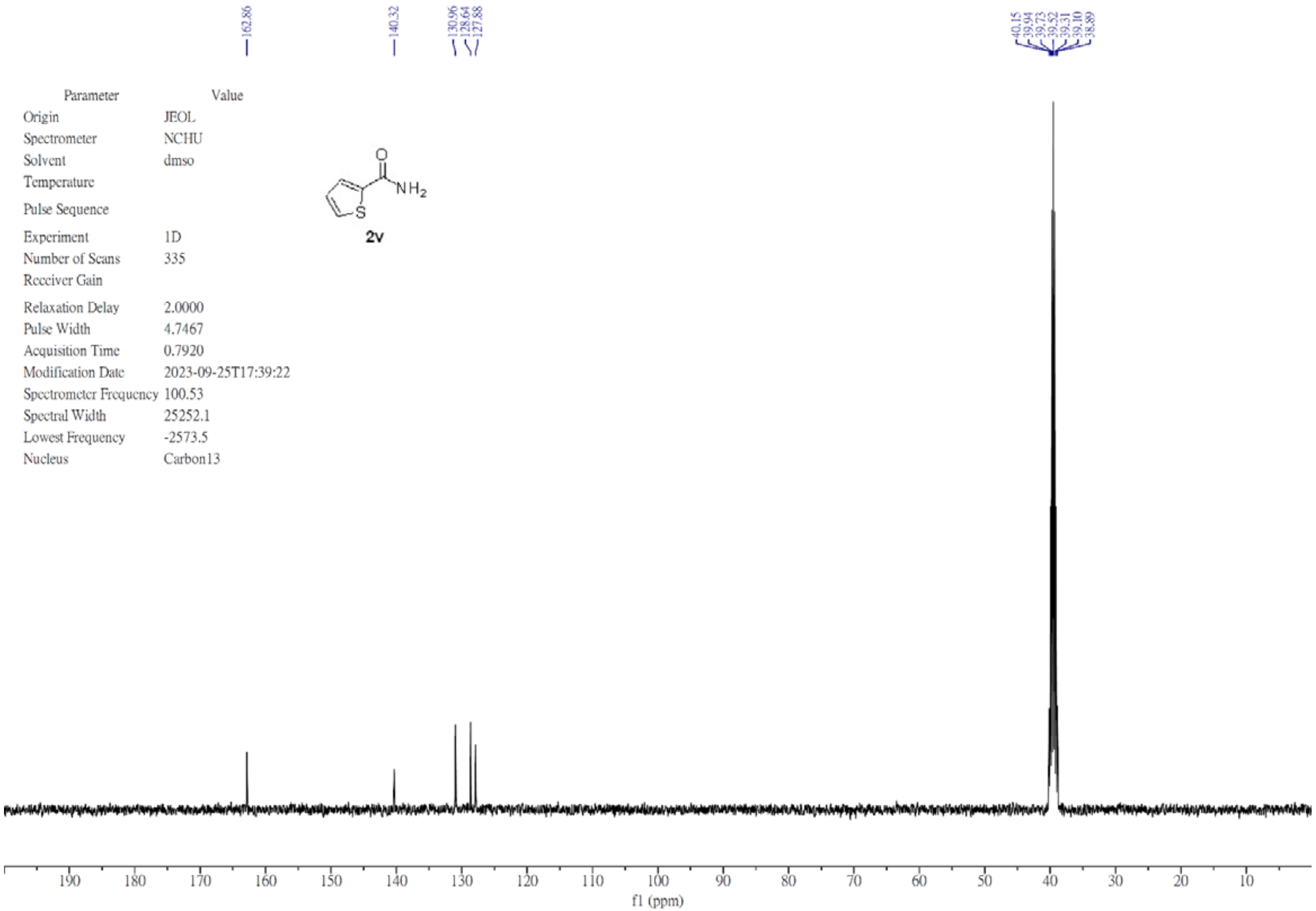

2v $^{13}C\{^{1}H\}$ NMR spectrum (100 MHz in $(CD_3)_2SO$)

Parameter	Value
Origin	Varian
Spectrometer	vnmrs
Solvent	cdcl3
Temperature	20.0
Pulse Sequence	s2pul
Experiment	1D
Number of Scans	60
Receiver Gain	44
Relaxation Delay	1.0000
Pulse Width	0.0000
Acquisition Time	2.5559
Acquisition Date	2023-09-29T16:28:52
Spectrometer Frequency	399.76
Spectral Width	6410.3
Lowest Frequency	-799.2
Nucleus	1H

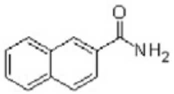

2w

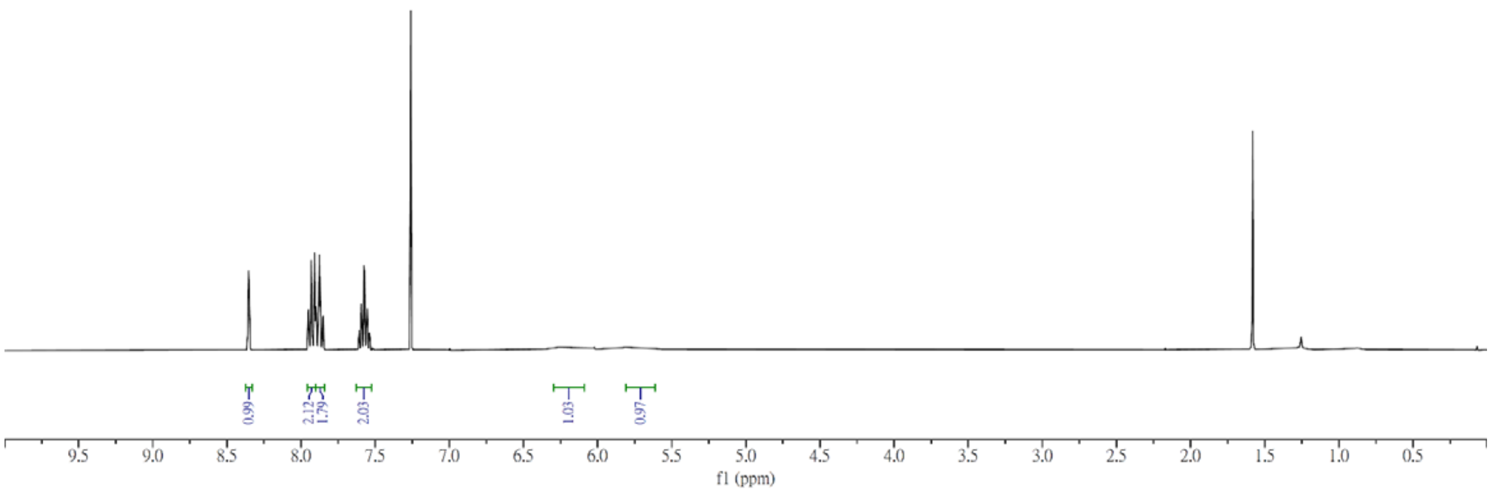

2w ^{1}H NMR spectrum (400 MHz in $CDCl_3$)

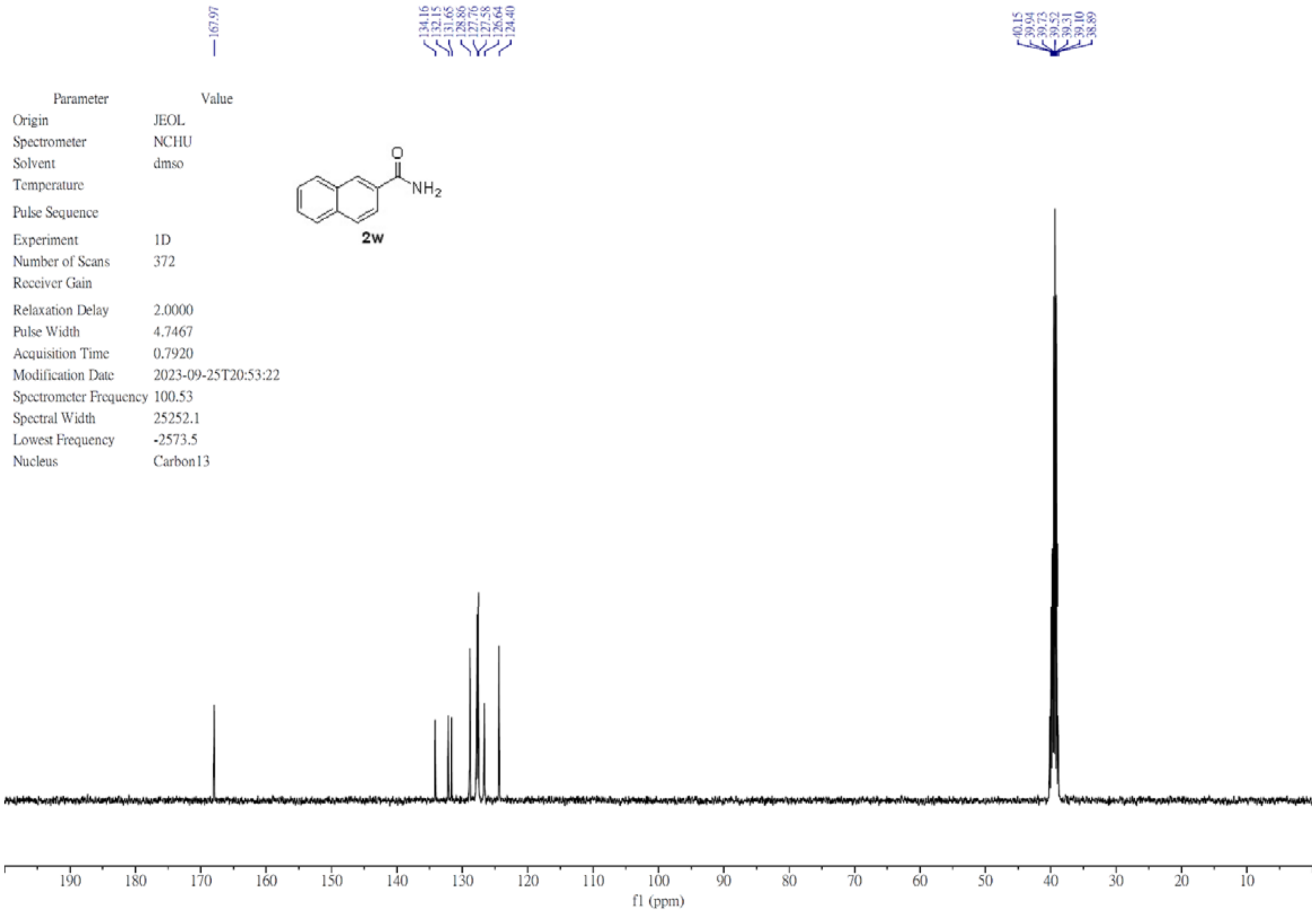

2w $^{13}C\{^{1}H\}$ NMR spectrum (100 MHz in $(CD_3)_2SO$)

Parameter	Value
Origin	Varian
Spectrometer	vnmrs
Solvent	cdcl3
Temperature	20.0
Pulse Sequence	s2pul
Experiment	1D
Number of Scans	16
Receiver Gain	42
Relaxation Delay	1.0000
Pulse Width	0.0000
Acquisition Time	2.5559
Acquisition Date	2023-09-05T18:38:32
Spectrometer Frequency	399.76
Spectral Width	6410.3
Lowest Frequency	-799.2
Nucleus	1H

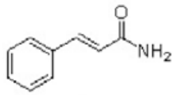

2x

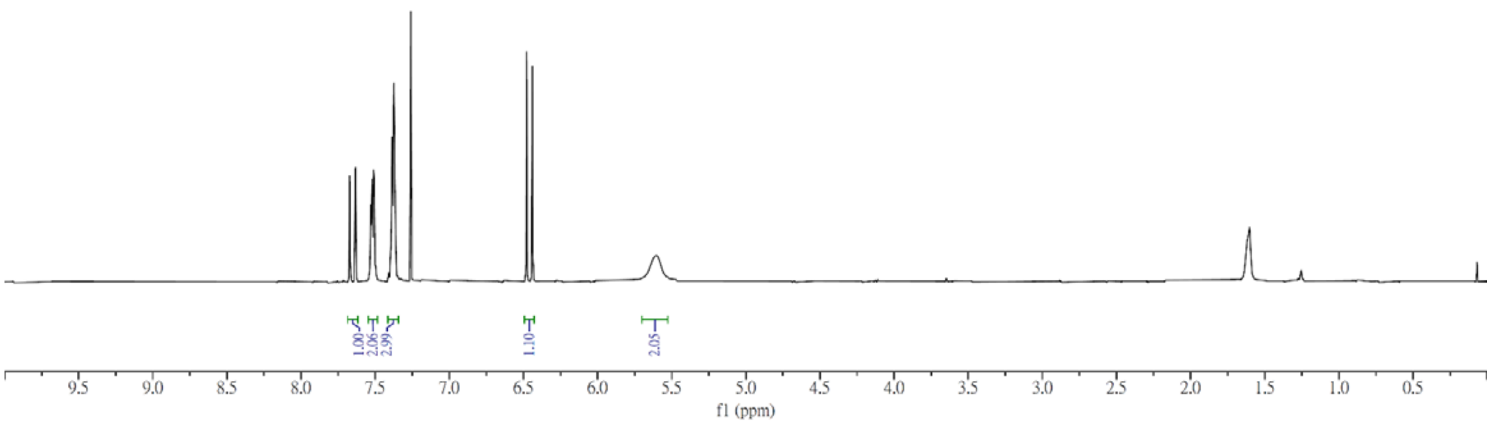

2x ^{1}H NMR spectrum (400 MHz in $CDCl_3$)

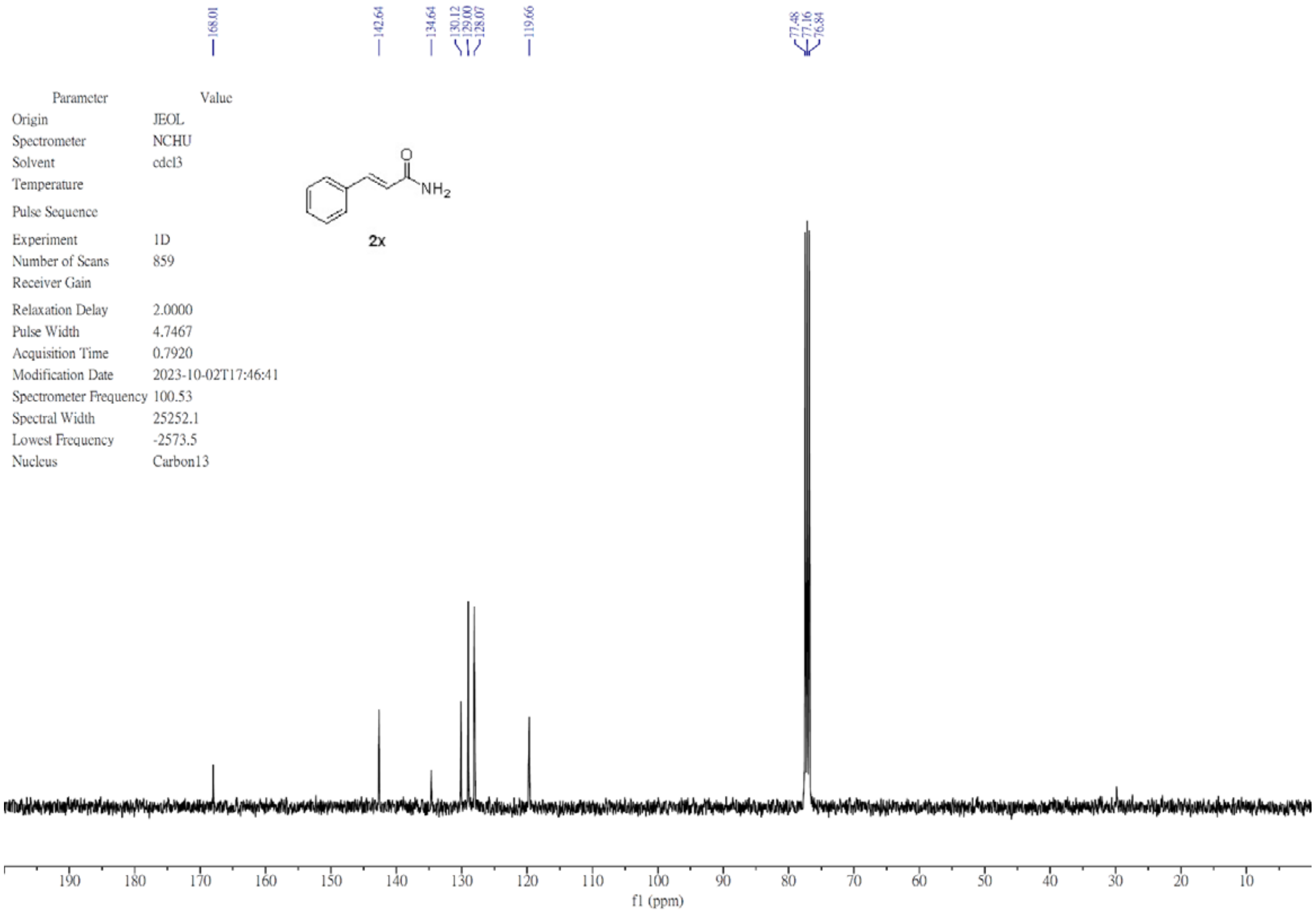

2x $^{13}C\{^{1}H\}$ NMR spectrum (100 MHz in $CDCl_3$)

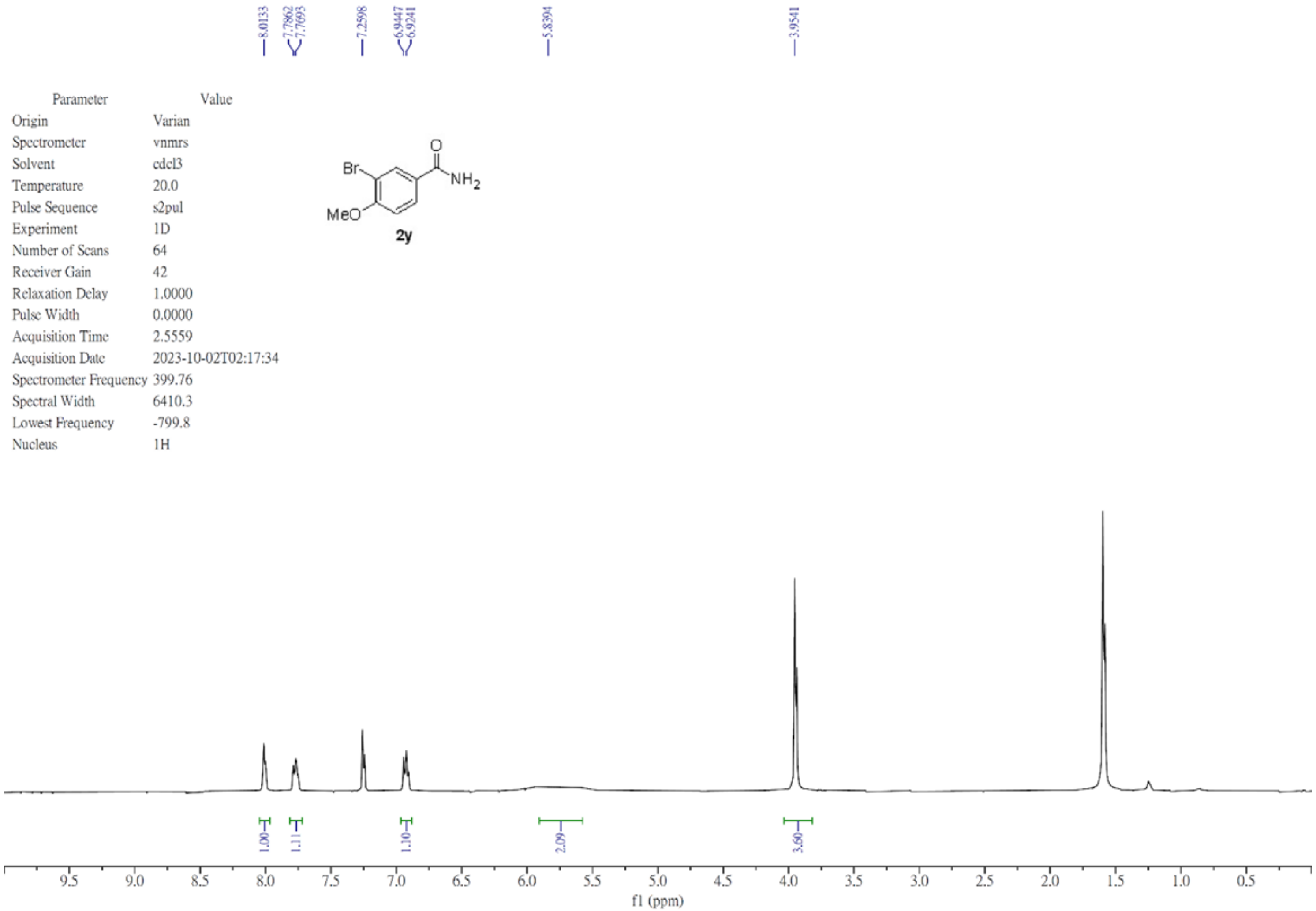

2y ^{1}H NMR spectrum (400 MHz in $CDCl_3$)

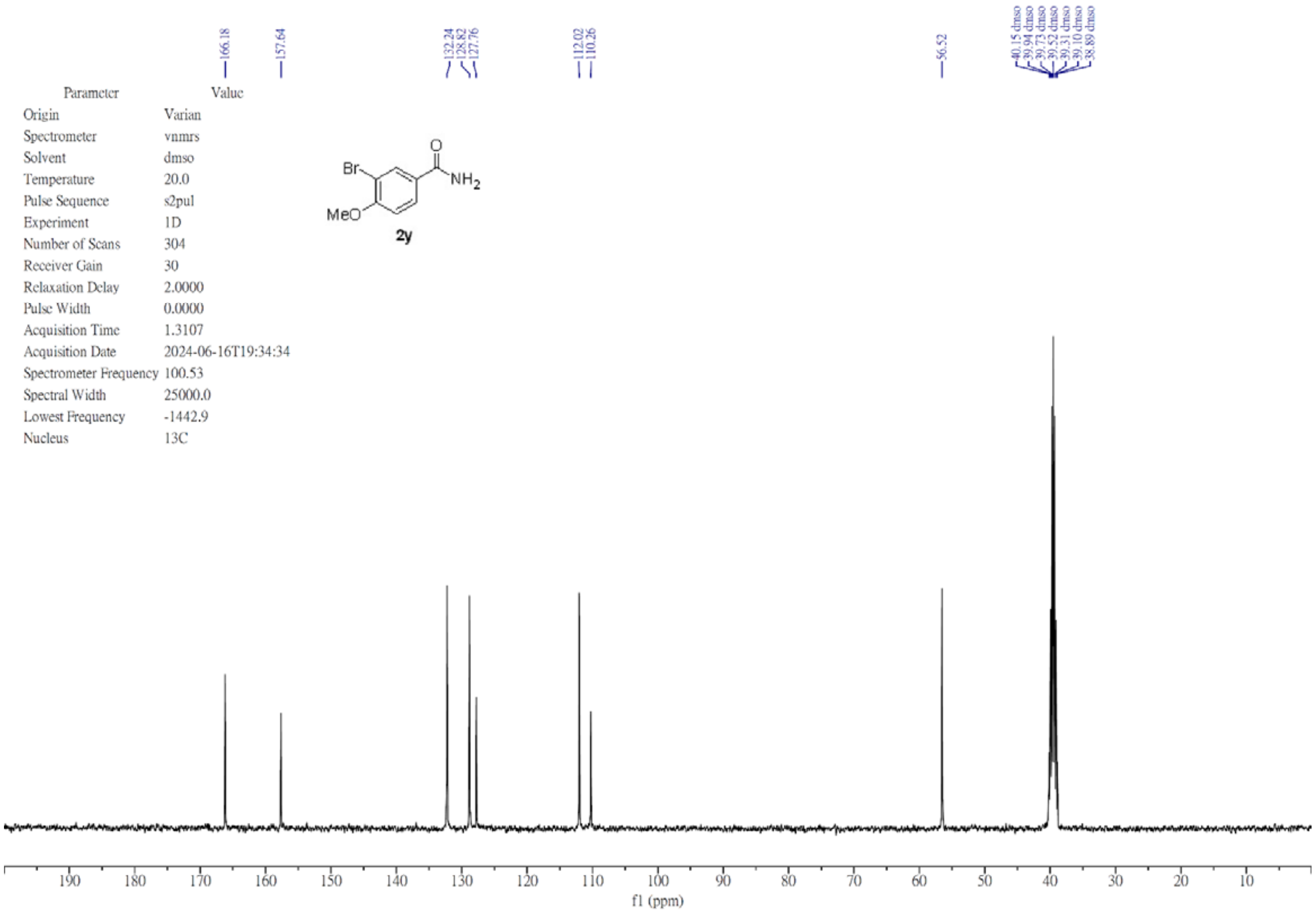

Parameter	Value
Origin	Varian
Spectrometer	vnmrs
Solvent	dmso
Temperature	20.0
Pulse Sequence	s2pul
Experiment	1D
Number of Scans	304
Receiver Gain	30
Relaxation Delay	2.0000
Pulse Width	0.0000
Acquisition Time	1.3107
Acquisition Date	2024-06-16T19:34:34
Spectrometer Frequency	100.53
Spectral Width	25000.0
Lowest Frequency	-1442.9
Nucleus	13C

2y $^{13}C\{^{1}H\}$ NMR spectrum (100 MHz in $(CD_3)_2SO$)

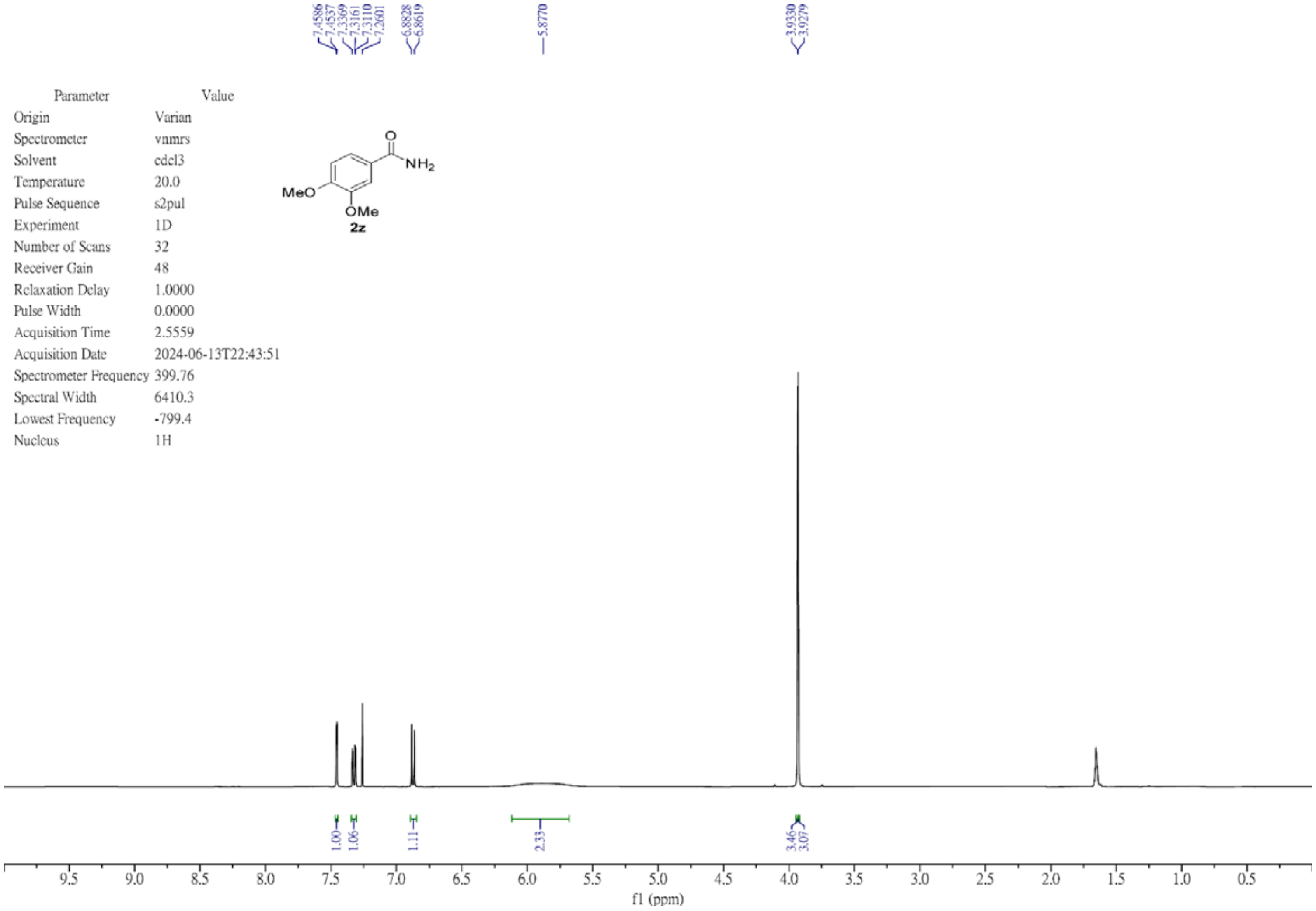

2z ^{1}H NMR spectrum (400 MHz in $CDCl_3$)

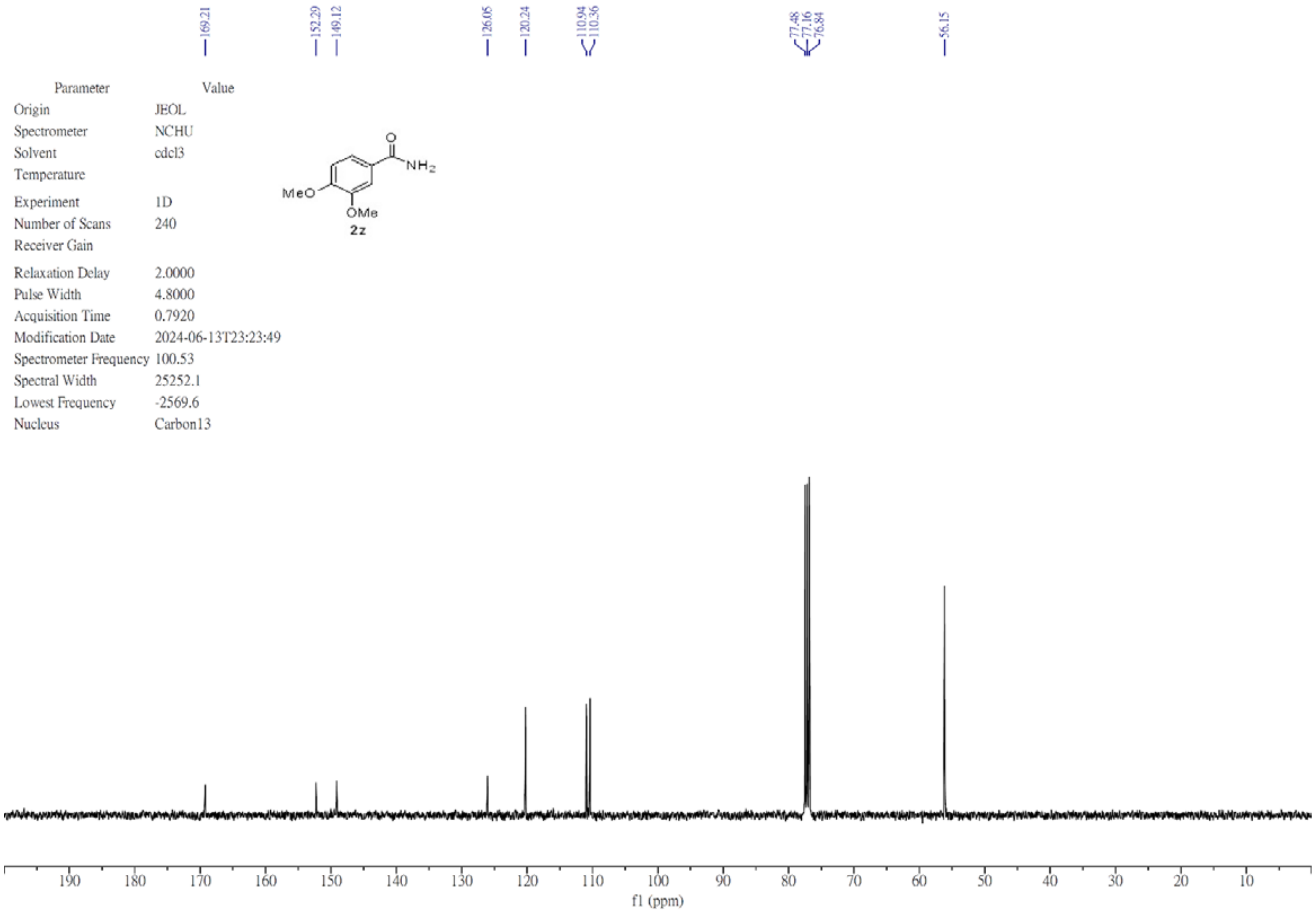

2z $^{13}C\{^{1}H\}$ NMR spectrum (100 MHz in $CDCl_3$)

Parameter	Value
Origin	Varian
Spectrometer	vnmrs
Solvent	CDCl3
Temperature	30.0
Pulse Sequence	s2pul
Experiment	1D
Number of Scans	32
Receiver Gain	50
Relaxation Delay	1.0000
Pulse Width	0.0000
Acquisition Time	2.5559
Modification Date	2022-12-26T18:15:00
Spectrometer Frequency	399.76
Spectral Width	6410.3
Lowest Frequency	-799.4
Nucleus	1H

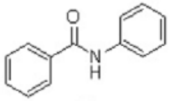

4aa

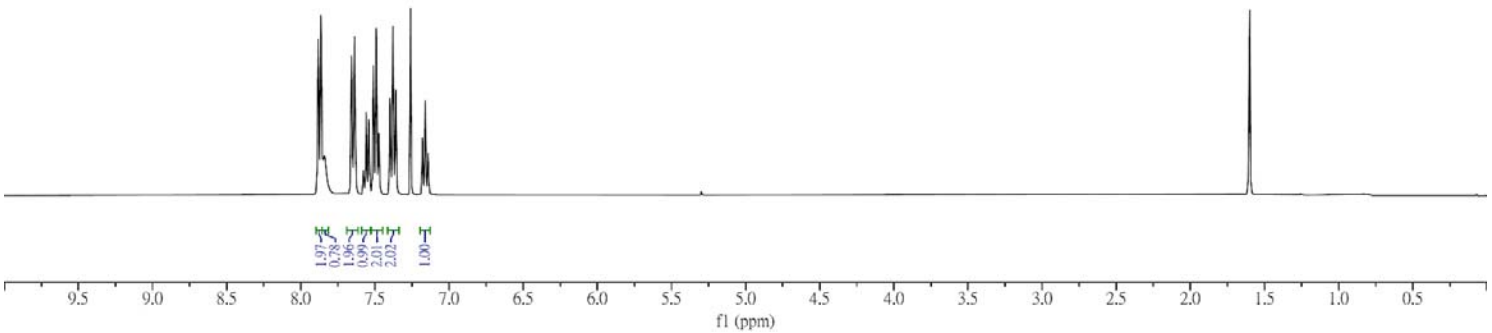

4aa ^{1}H NMR spectrum (400 MHz in $CDCl_3$

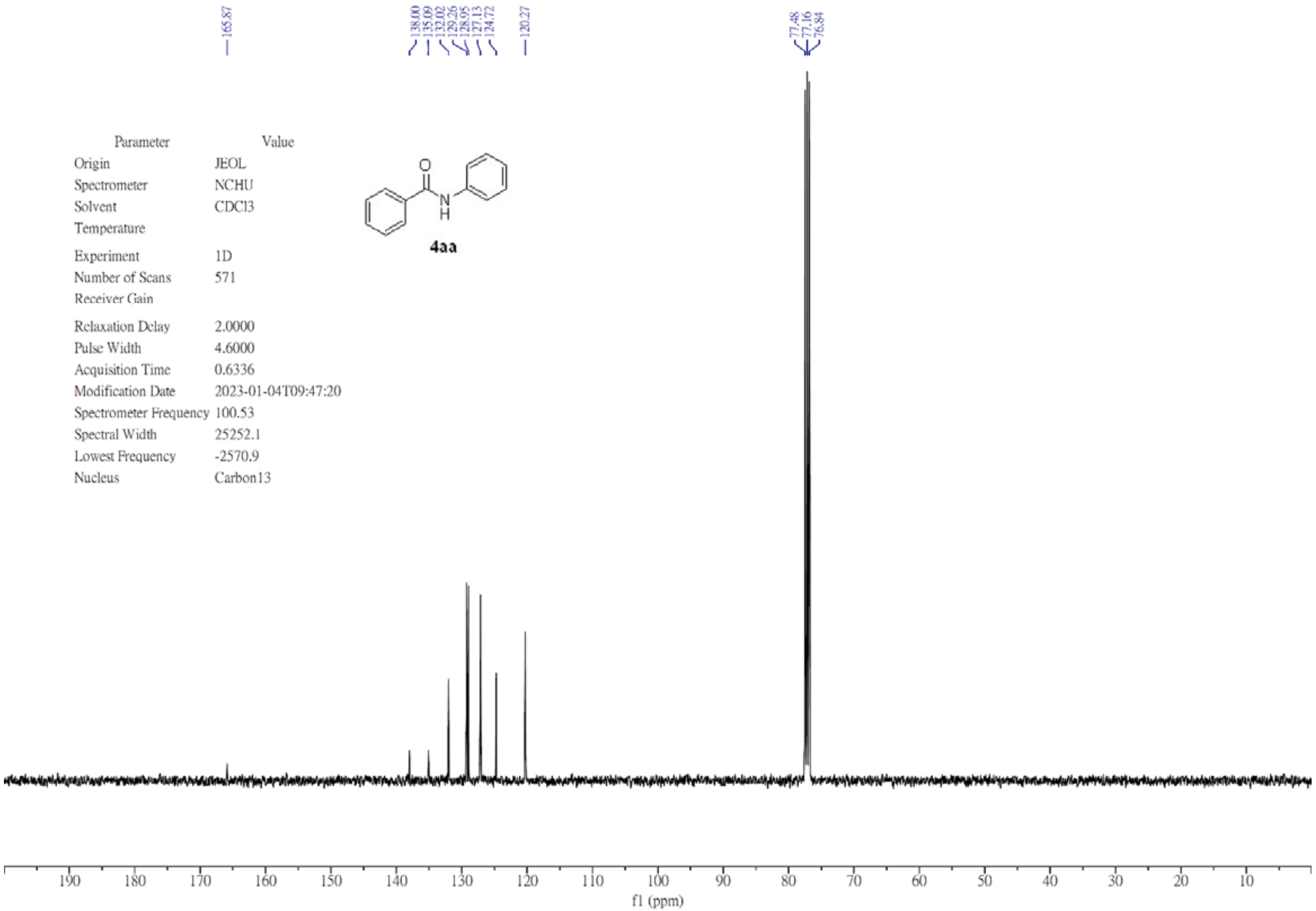

4aa $^{13}C\{^{1}H\}$ NMR spectrum (100 MHz in $CDCl_3$)

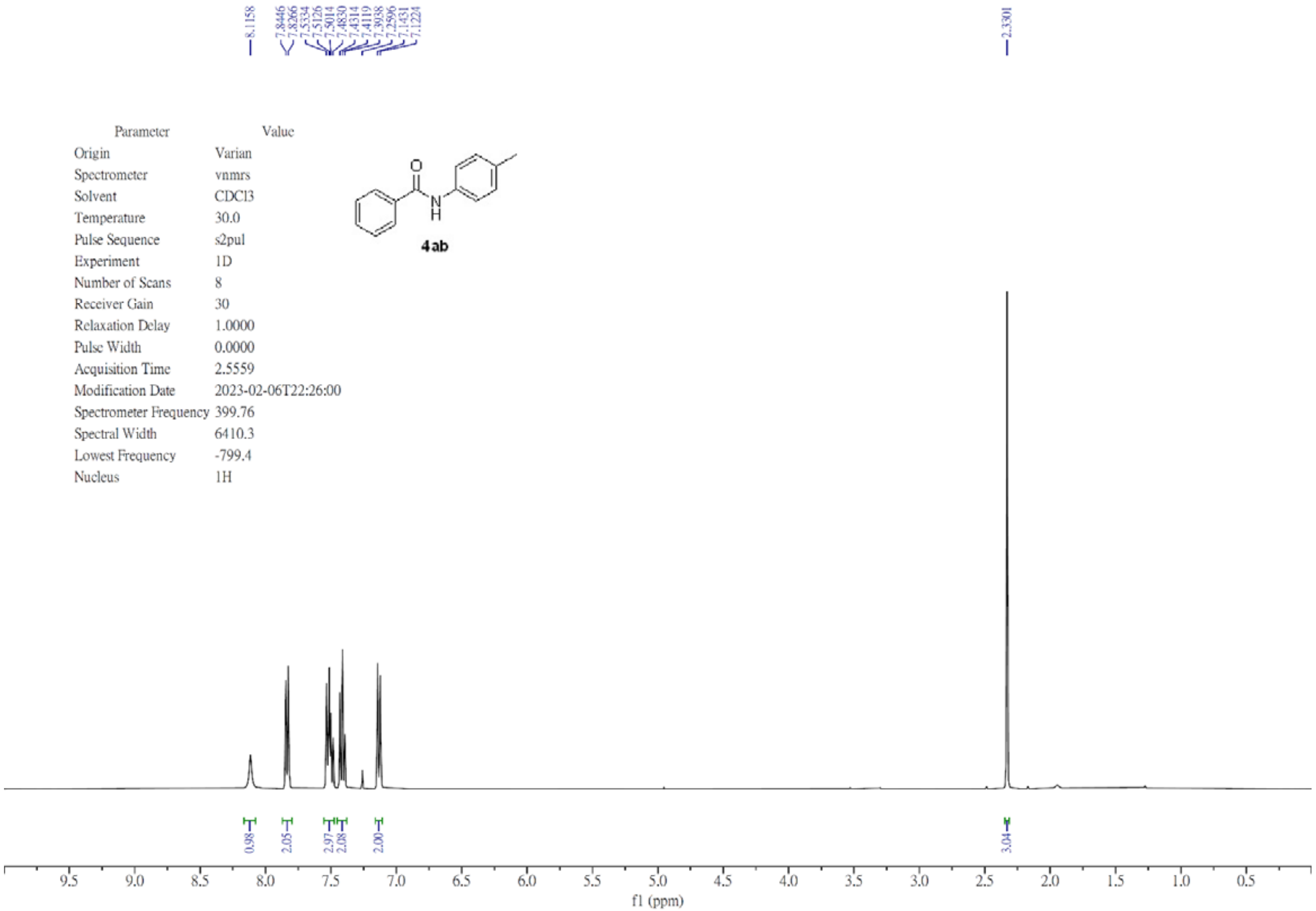

4ab ^{1}H NMR spectrum (400 MHz in $CDCl_3$)

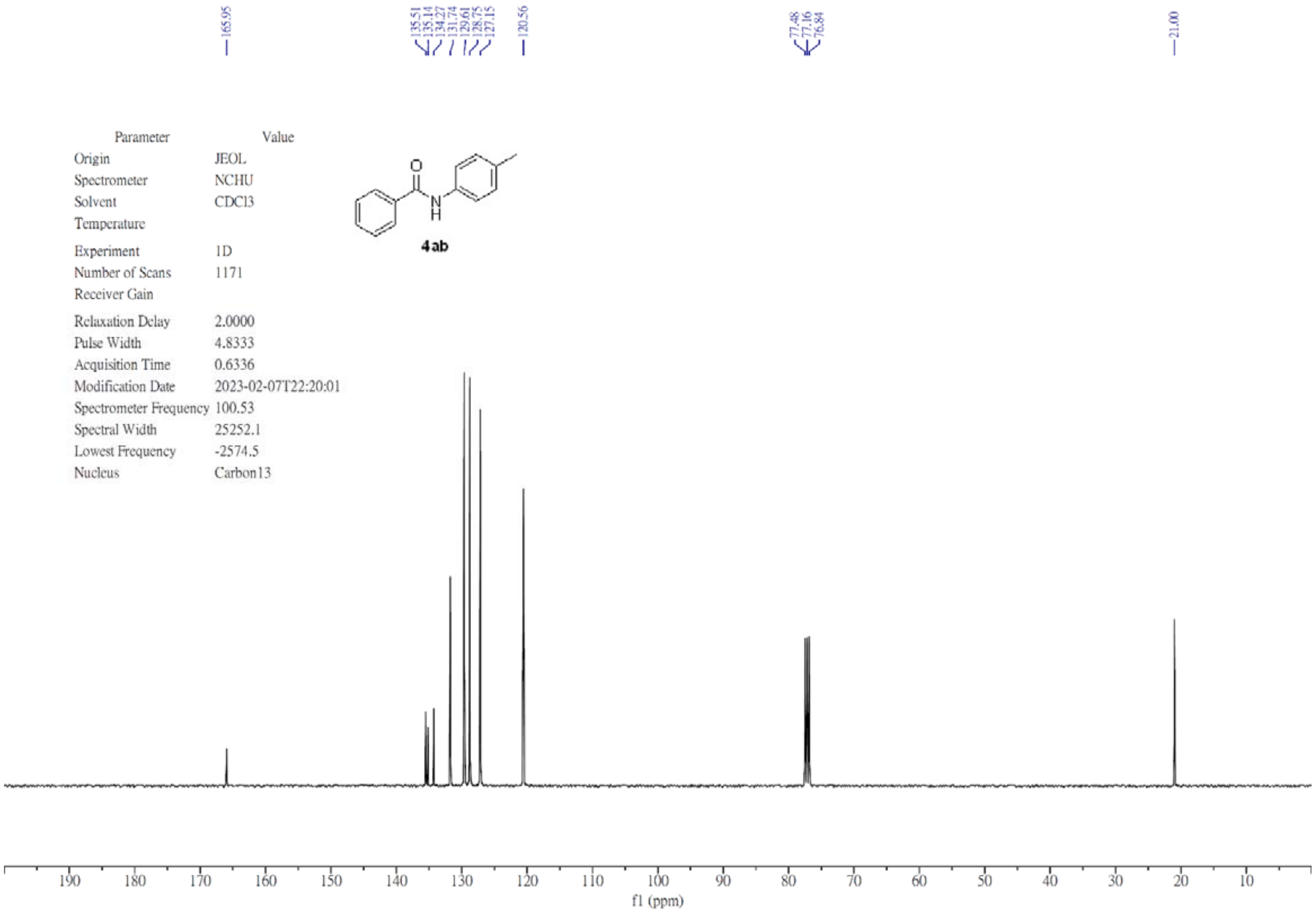

Parameter	Value
Origin	JEOL
Spectrometer	NCHU
Solvent	CDCl3
Temperature	
Experiment	1D
Number of Scans	1171
Receiver Gain	
Relaxation Delay	2.0000
Pulse Width	4.8333
Acquisition Time	0.6336
Modification Date	2023-02-07T22:20:01
Spectrometer Frequency	100.53
Spectral Width	25252.1
Lowest Frequency	-2574.5
Nucleus	Carbon13

4ab $^{13}C\{^{1}H\}$ NMR spectrum (100 MHz in $CDCl_3$)

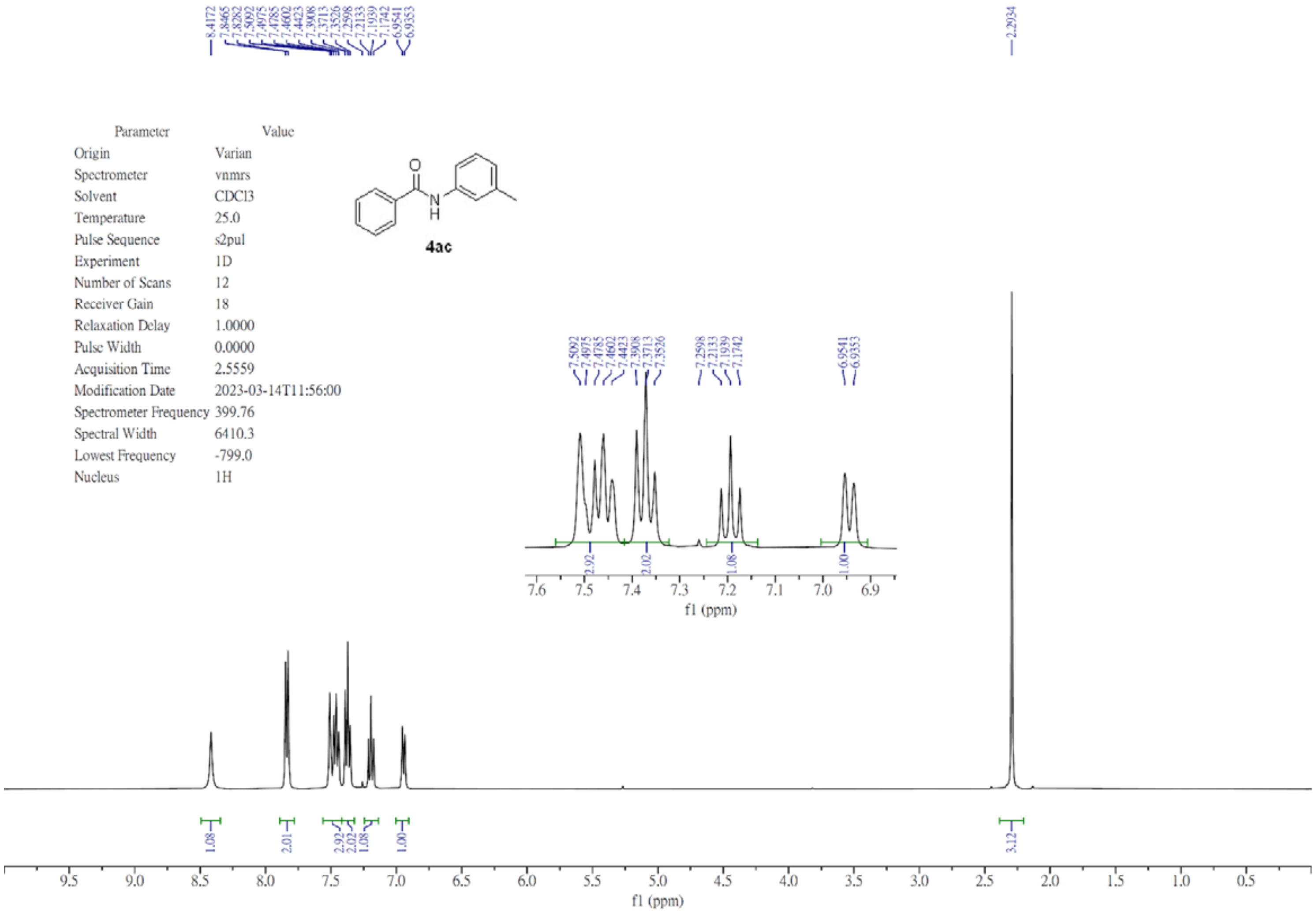

4ac ^{1}H NMR spectrum (400 MHz in $CDCl_3$)

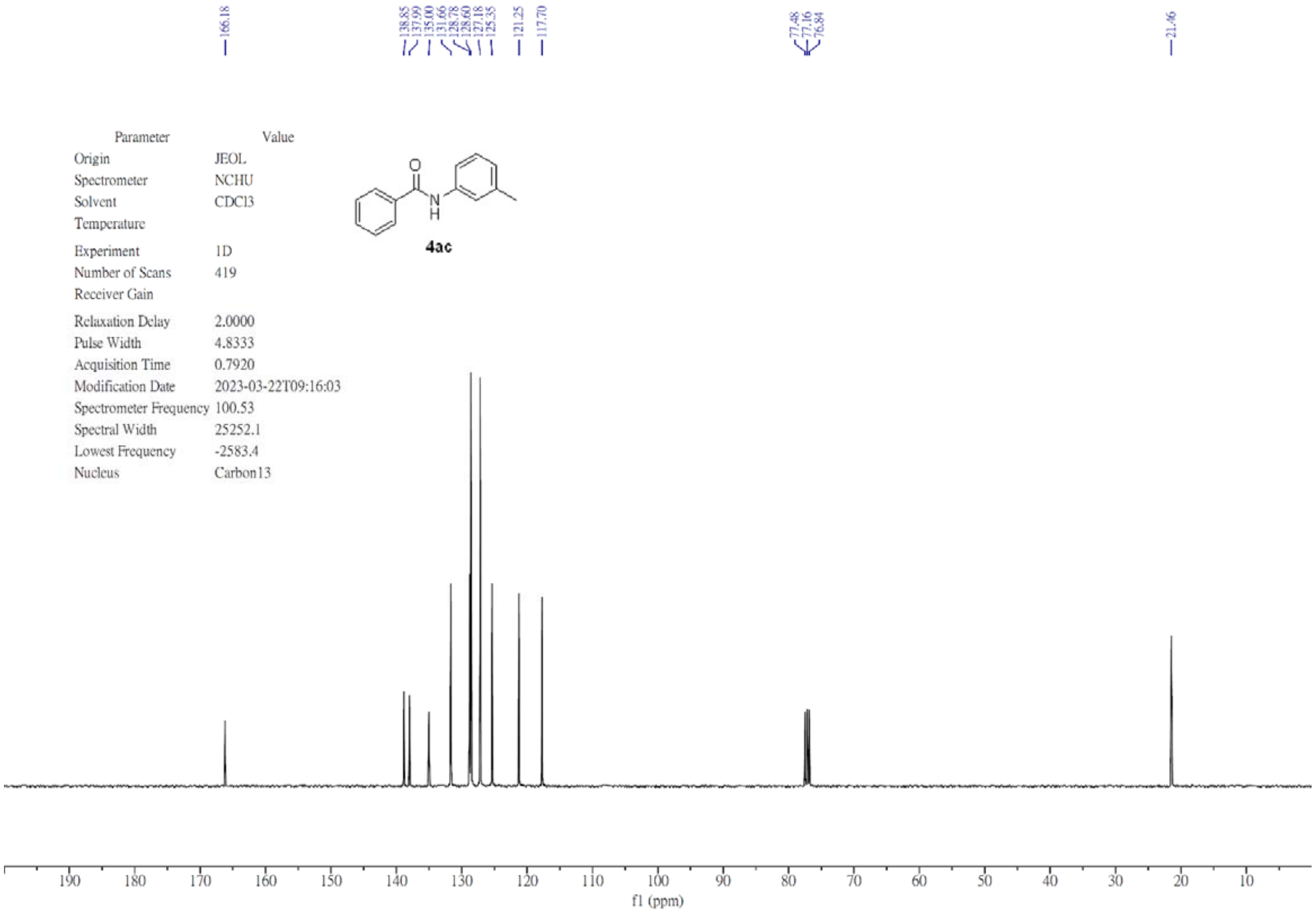

4ac $^{13}C\{^{1}H\}$ NMR spectrum (100 MHz in $CDCl_3$)

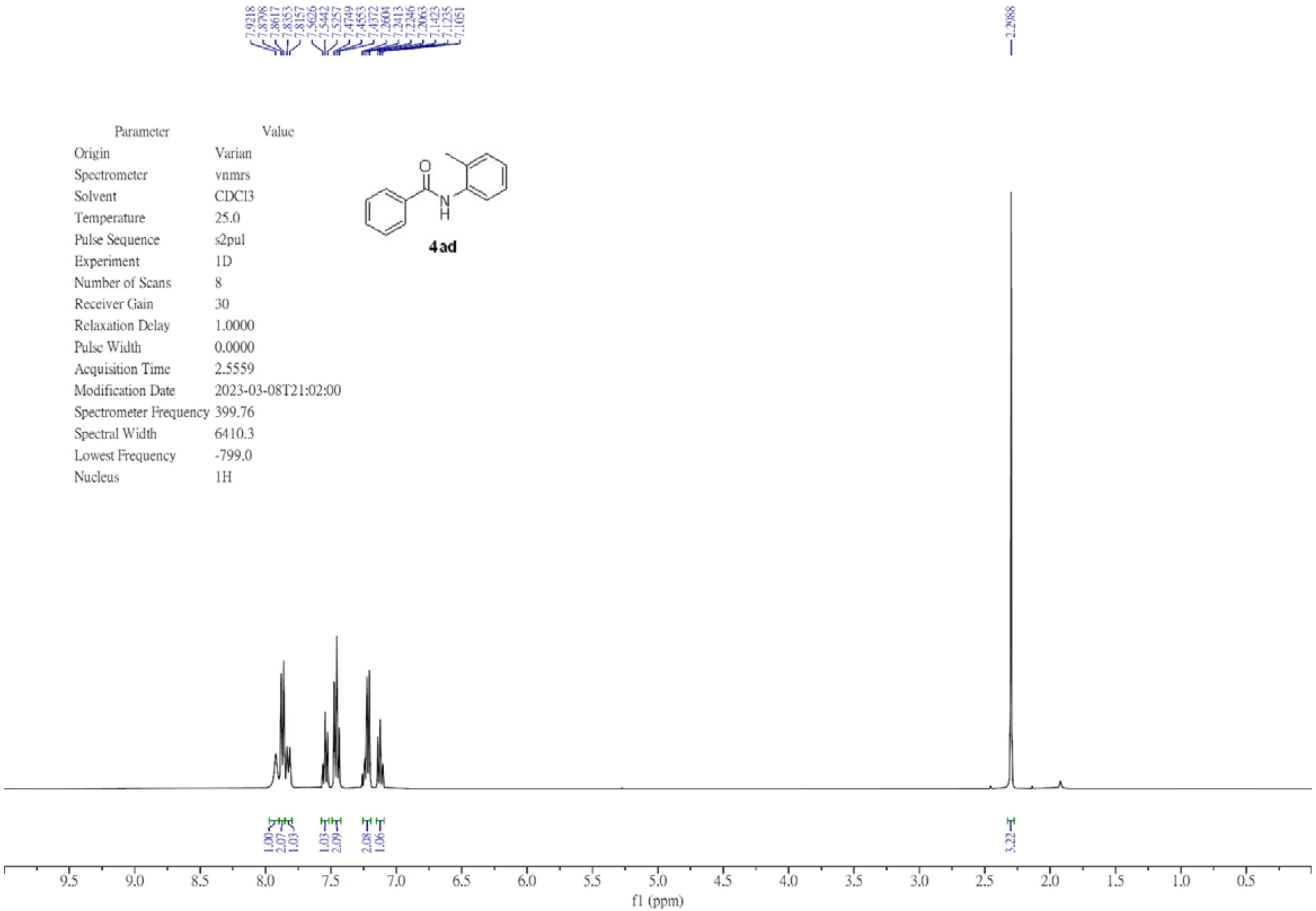

Parameter	Value
Origin	Varian
Spectrometer	vnmrs
Solvent	CDCl3
Temperature	25.0
Pulse Sequence	s2pul
Experiment	1D
Number of Scans	8
Receiver Gain	30
Relaxation Delay	1.0000
Pulse Width	0.0000
Acquisition Time	2.5559
Modification Date	2023-03-08T21:02:00
Spectrometer Frequency	399.76
Spectral Width	6410.3
Lowest Frequency	-799.0
Nucleus	1H

4ad ^{1}H NMR spectrum (400 MHz in $CDCl_3$)

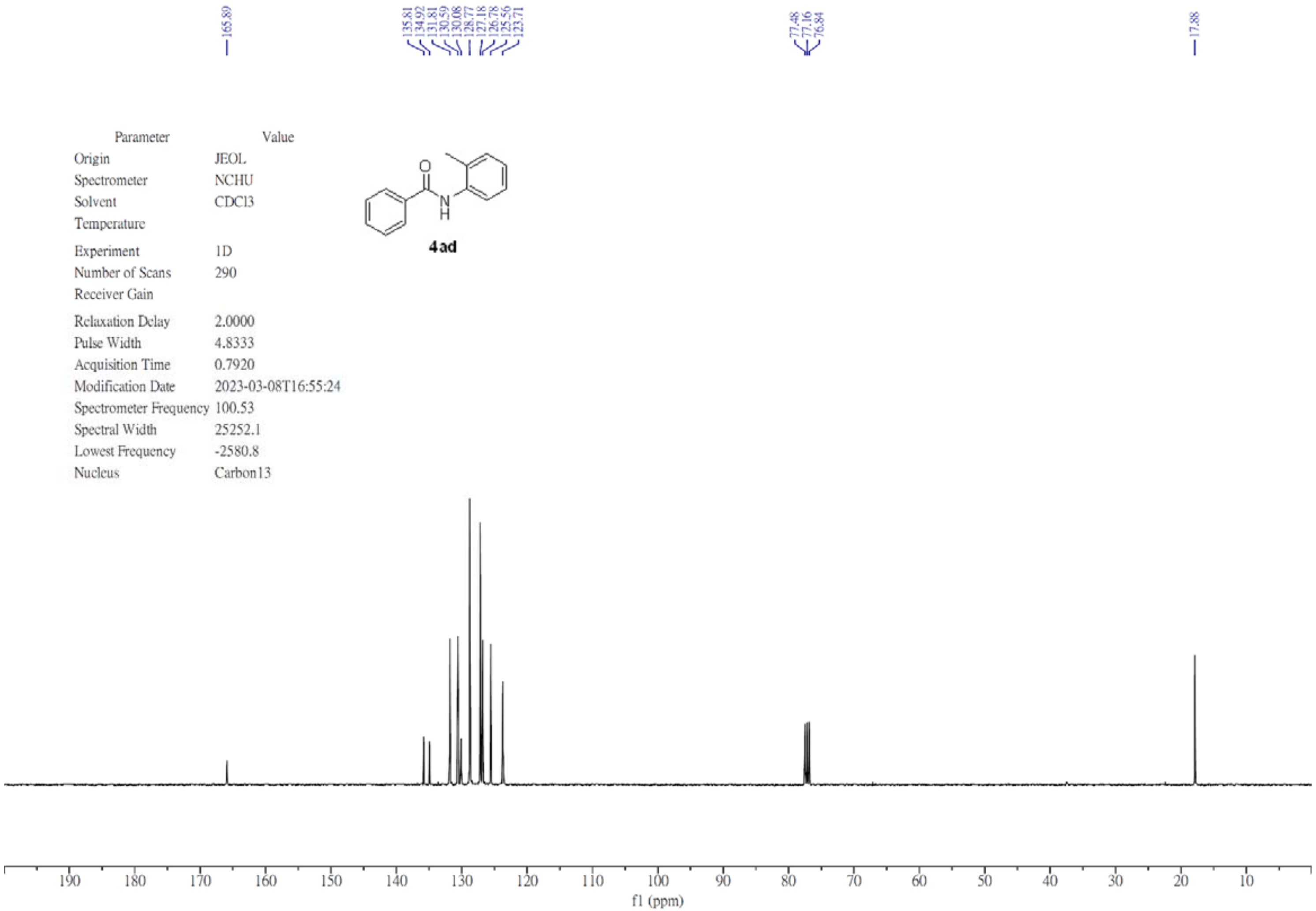

4ad $^{13}C\{^{1}H\}$ NMR spectrum (100 MHz in $CDCl_3$)

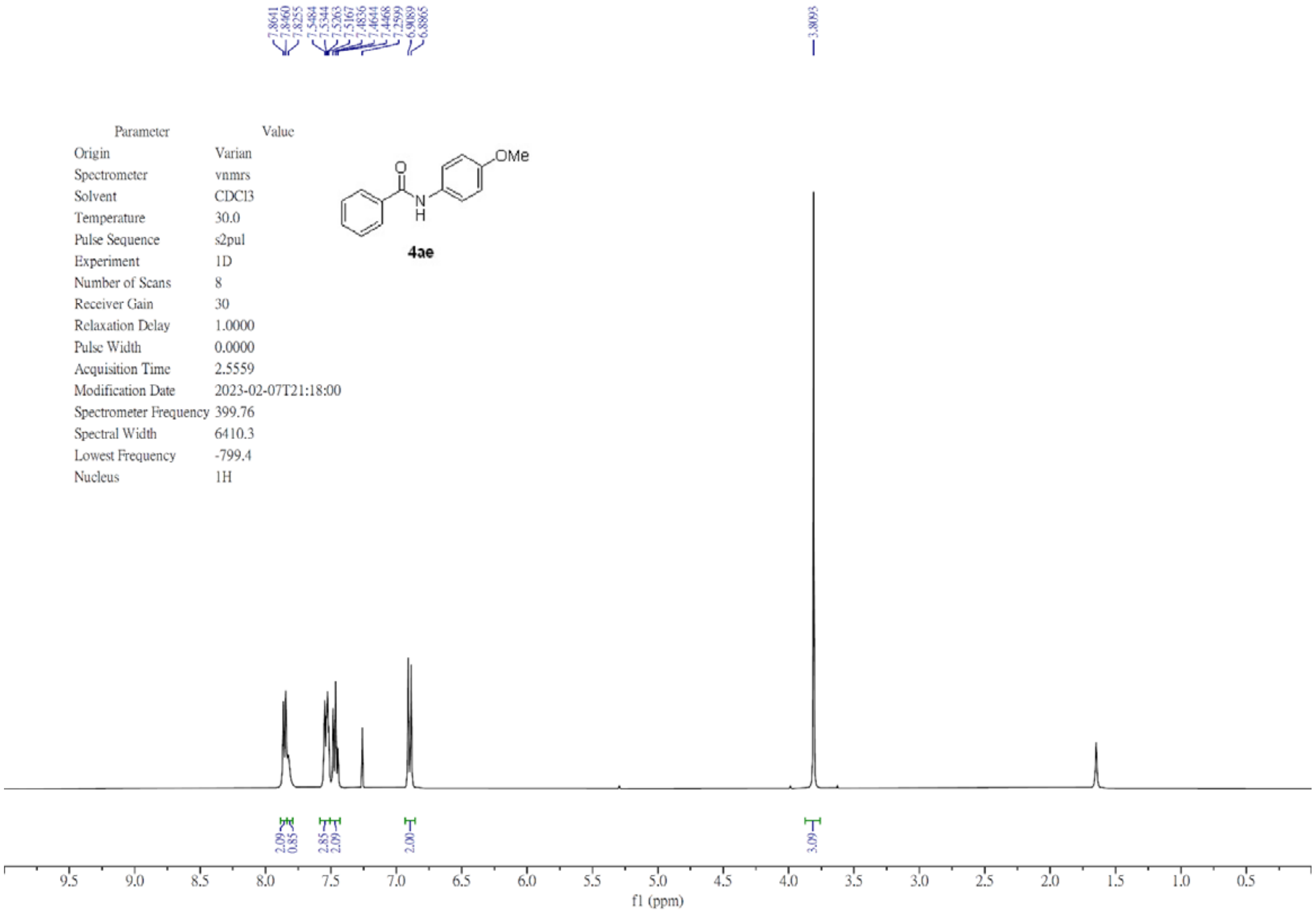

4ae ^{1}H NMR spectrum (400 MHz in $CDCl_3$)

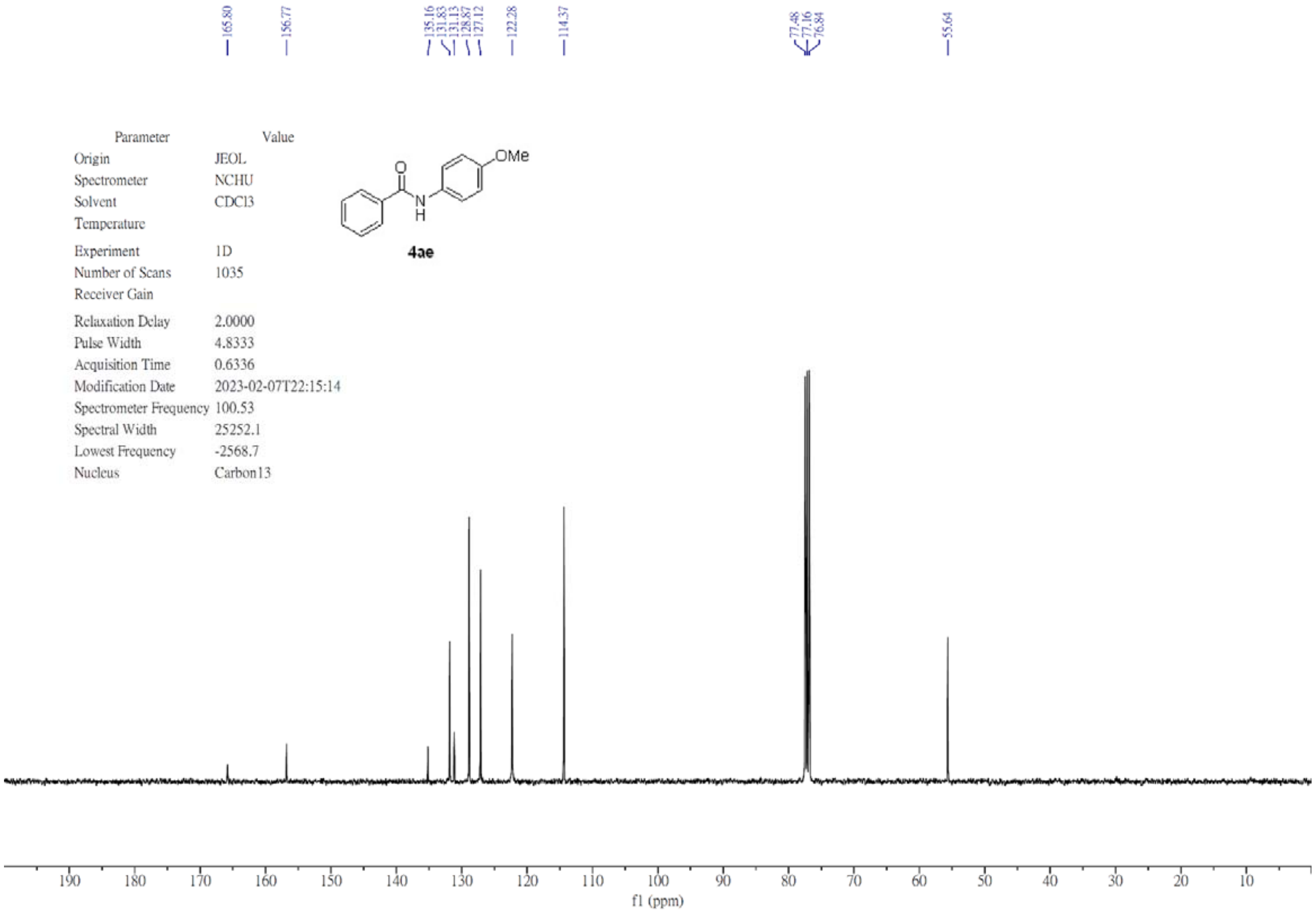

4ae $^{13}C\{^{1}H\}$ NMR spectrum (100 MHz in $CDCl_3$)

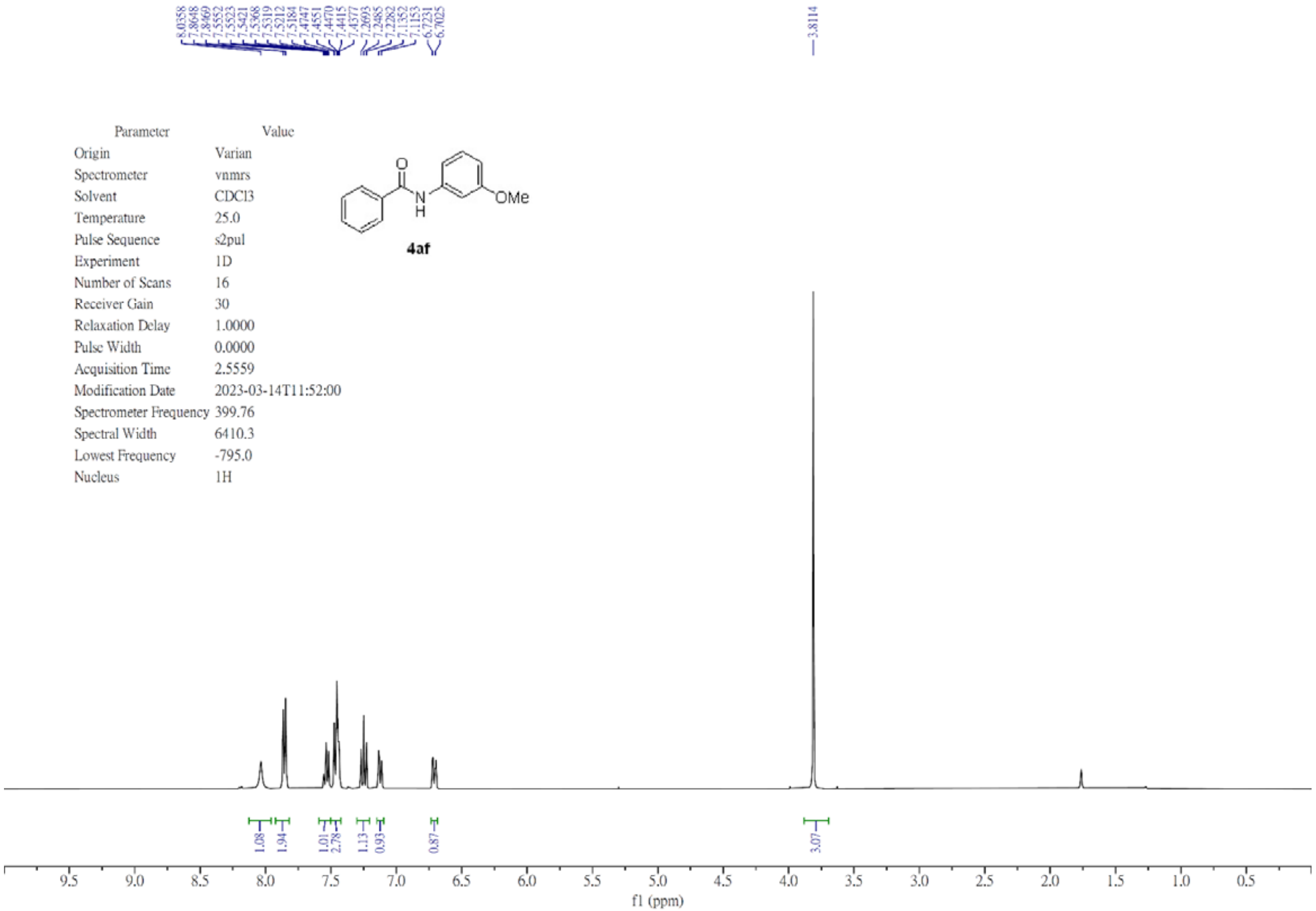

4af ^{1}H NMR spectrum (400 MHz in $CDCl_3$)

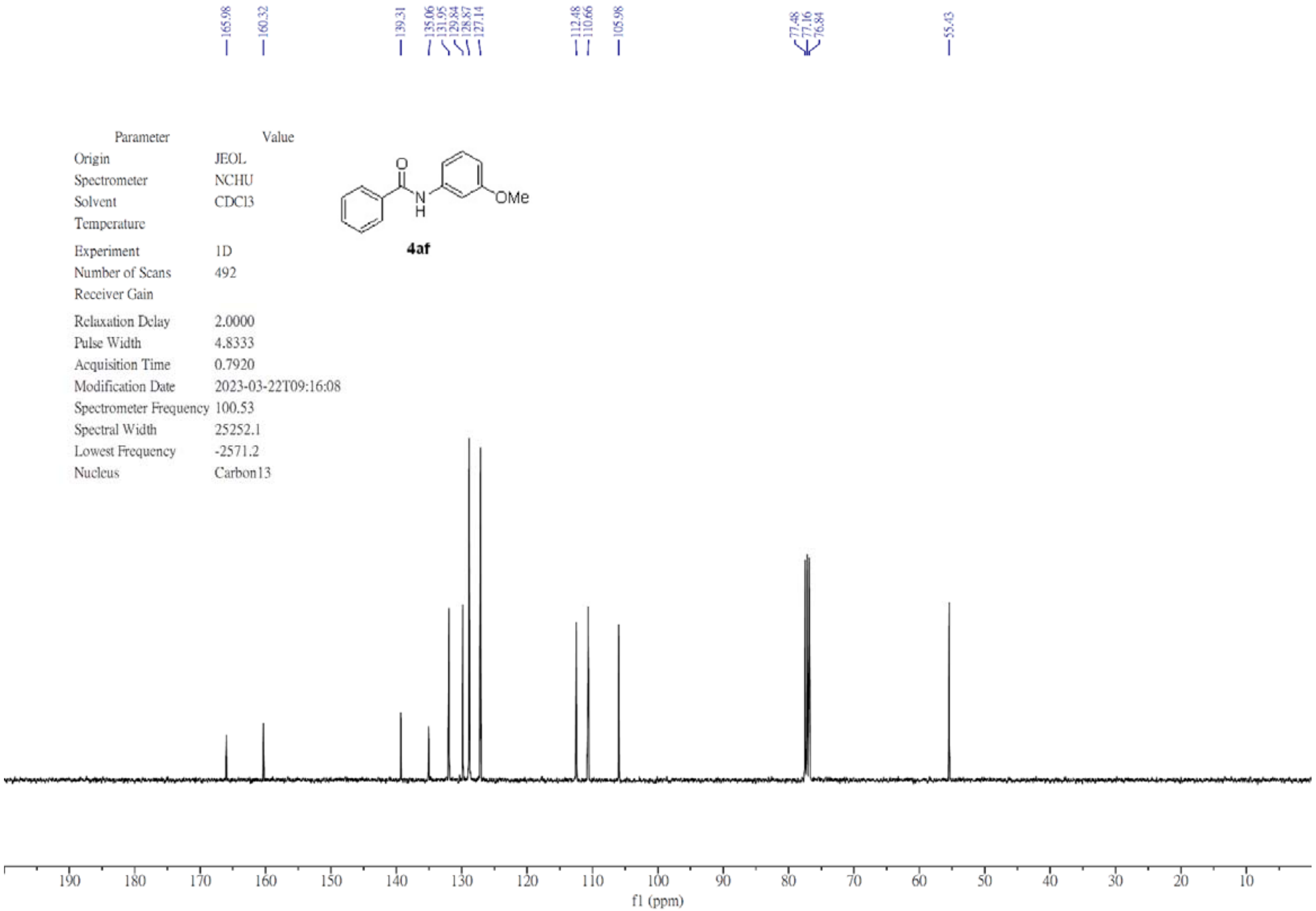

4af $^{13}C\{^1H\}$ NMR spectrum (100 MHz in $CDCl_3$)

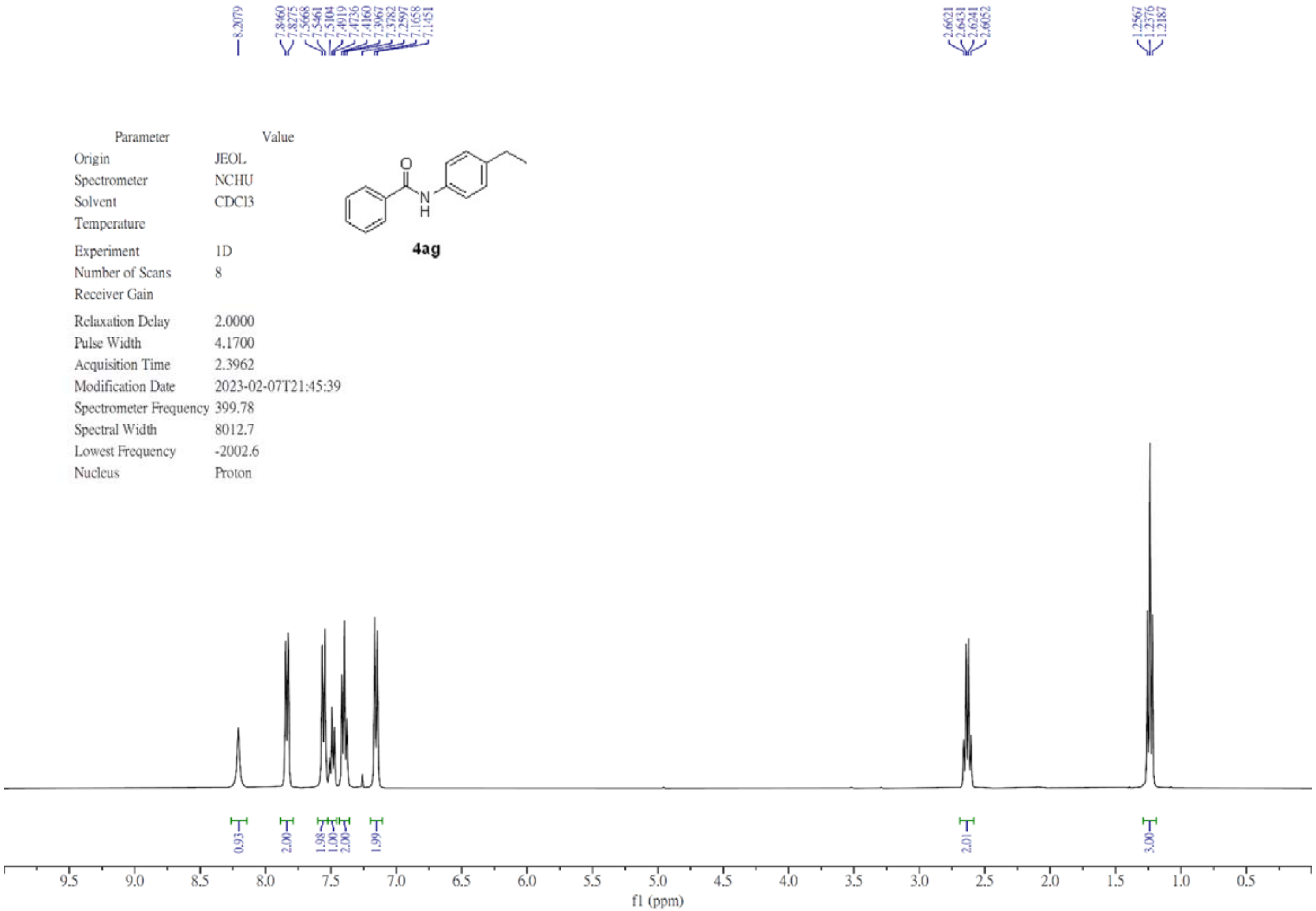

4ag ^{1}H NMR spectrum (400 MHz in $CDCl_3$)

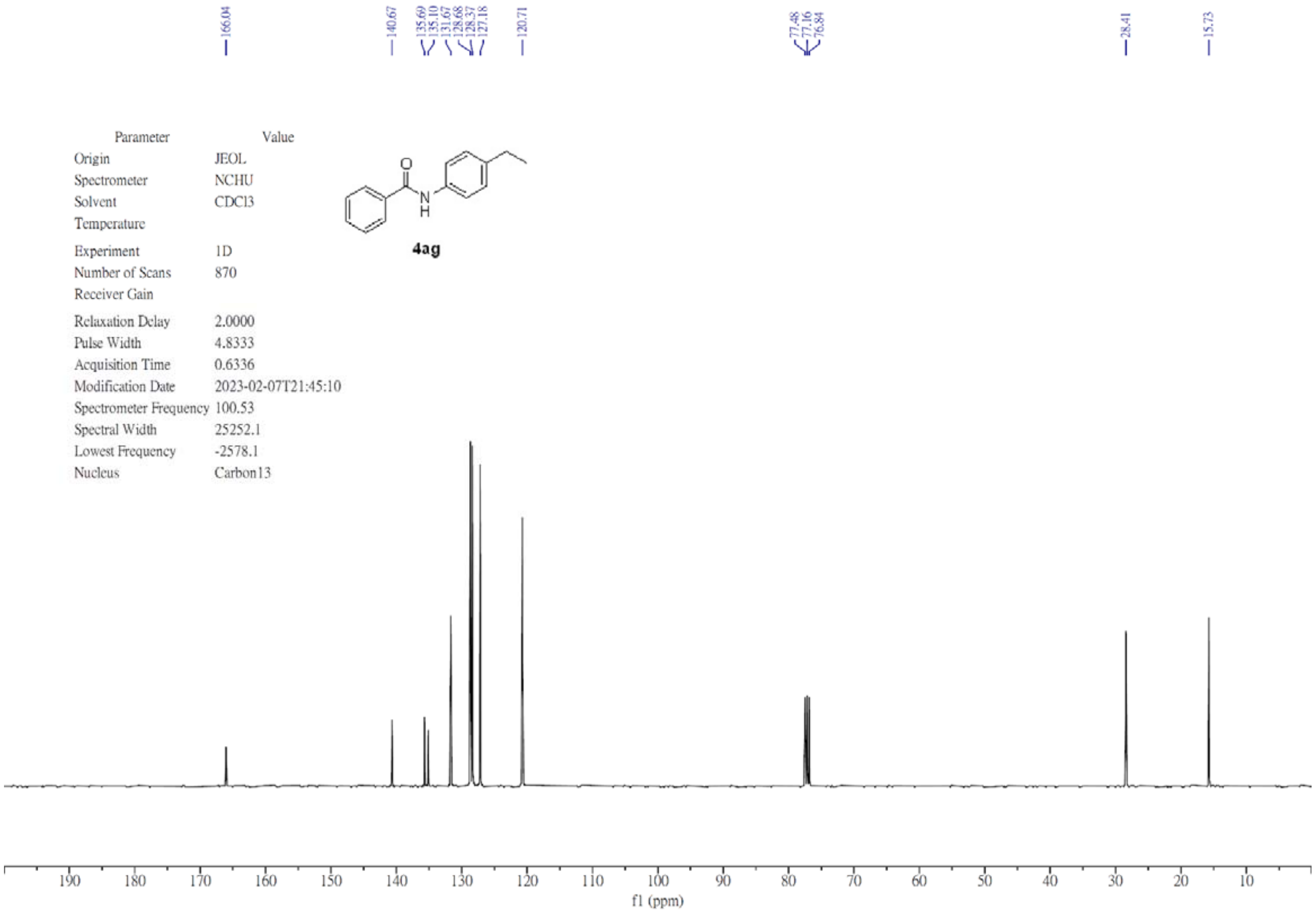

4ag $^{13}C\{^{1}H\}$ NMR spectrum (100 MHz in $CDCl_3$)

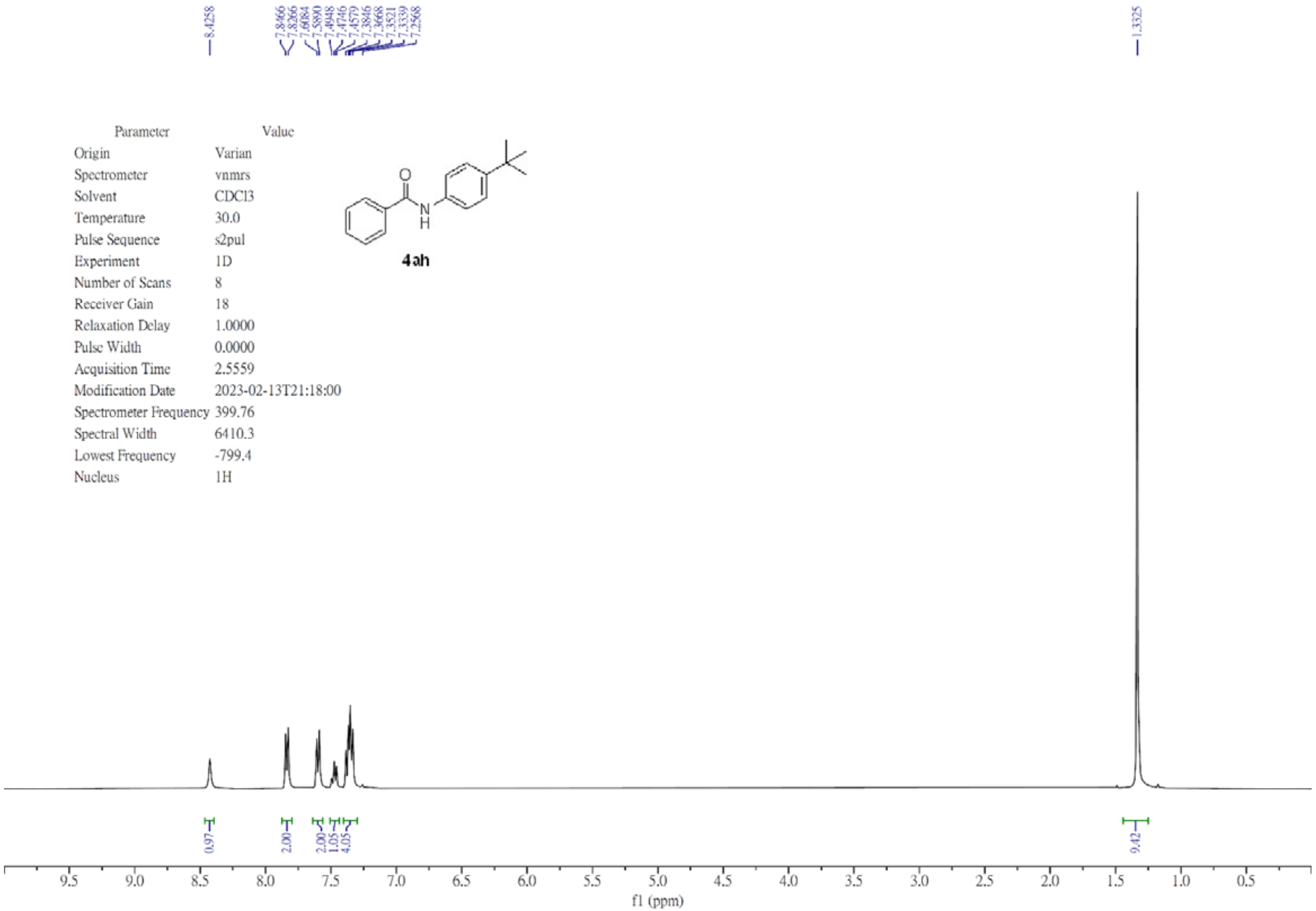

4ah ^{1}H NMR spectrum (400 MHz in $(CD_3)_2SO$)

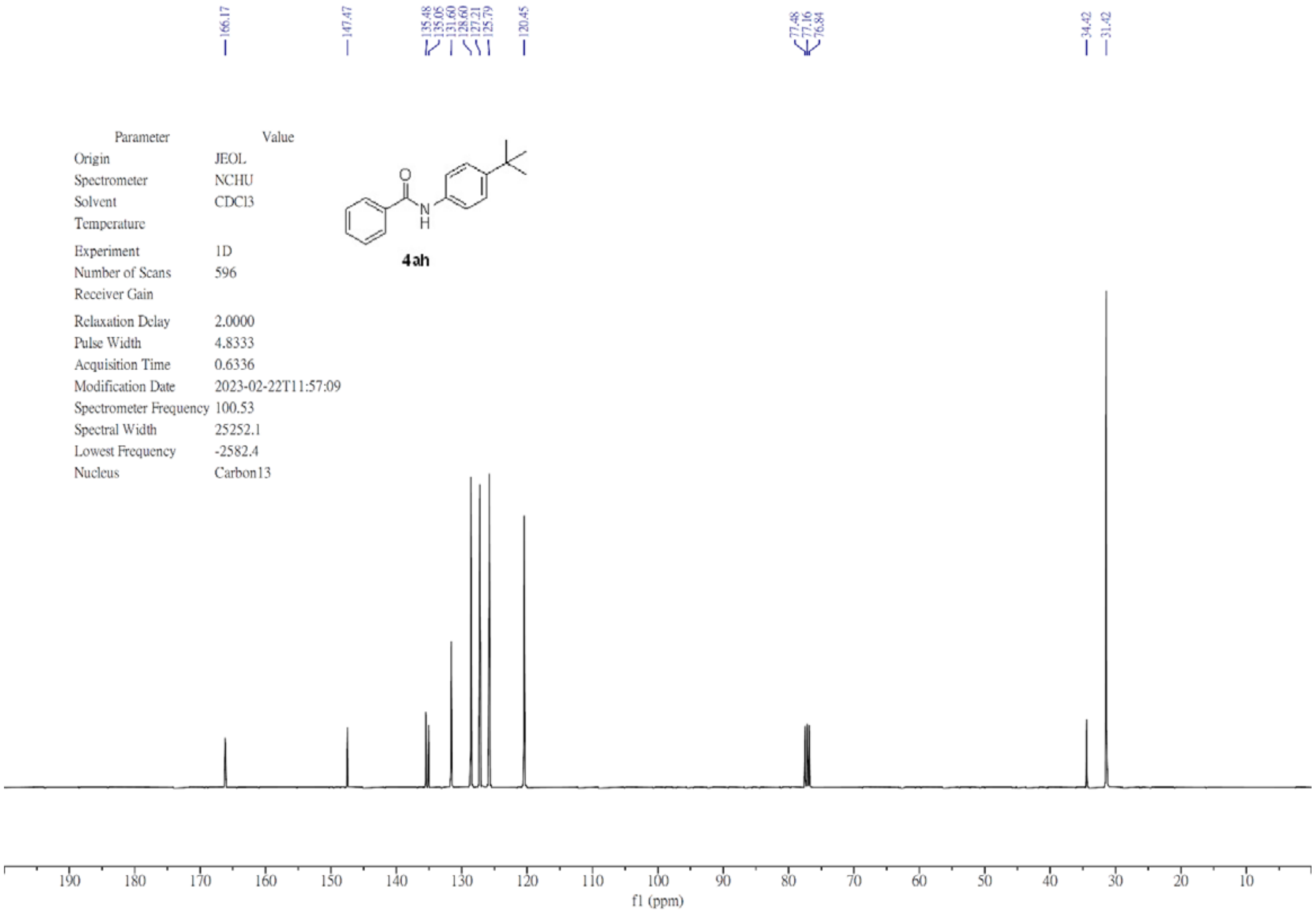

4ah $^{13}C\{^{1}H\}$ NMR spectrum (100 MHz in $CDCl_3$)

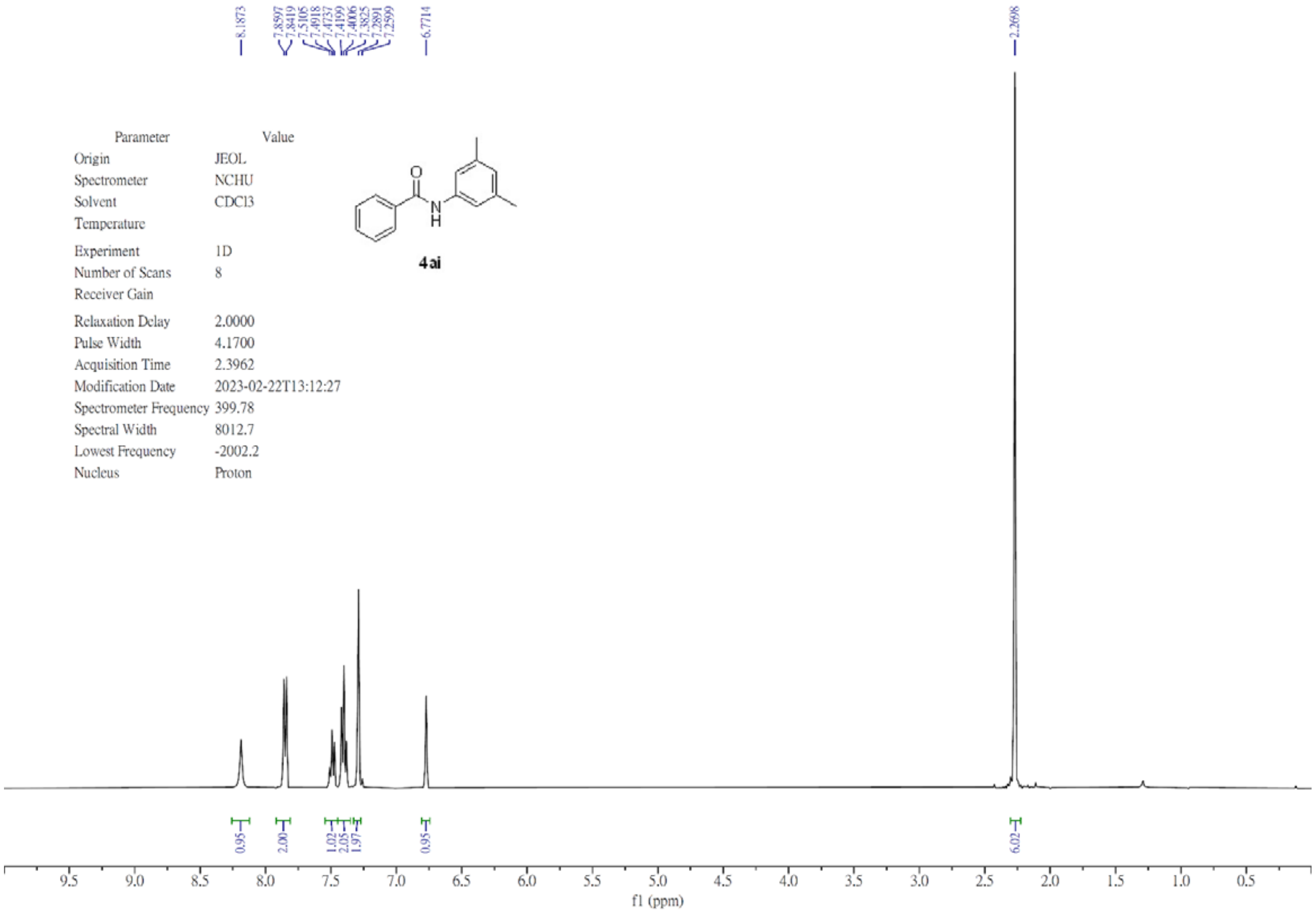

4ai ^{1}H NMR spectrum (400 MHz in $CDCl_3$)

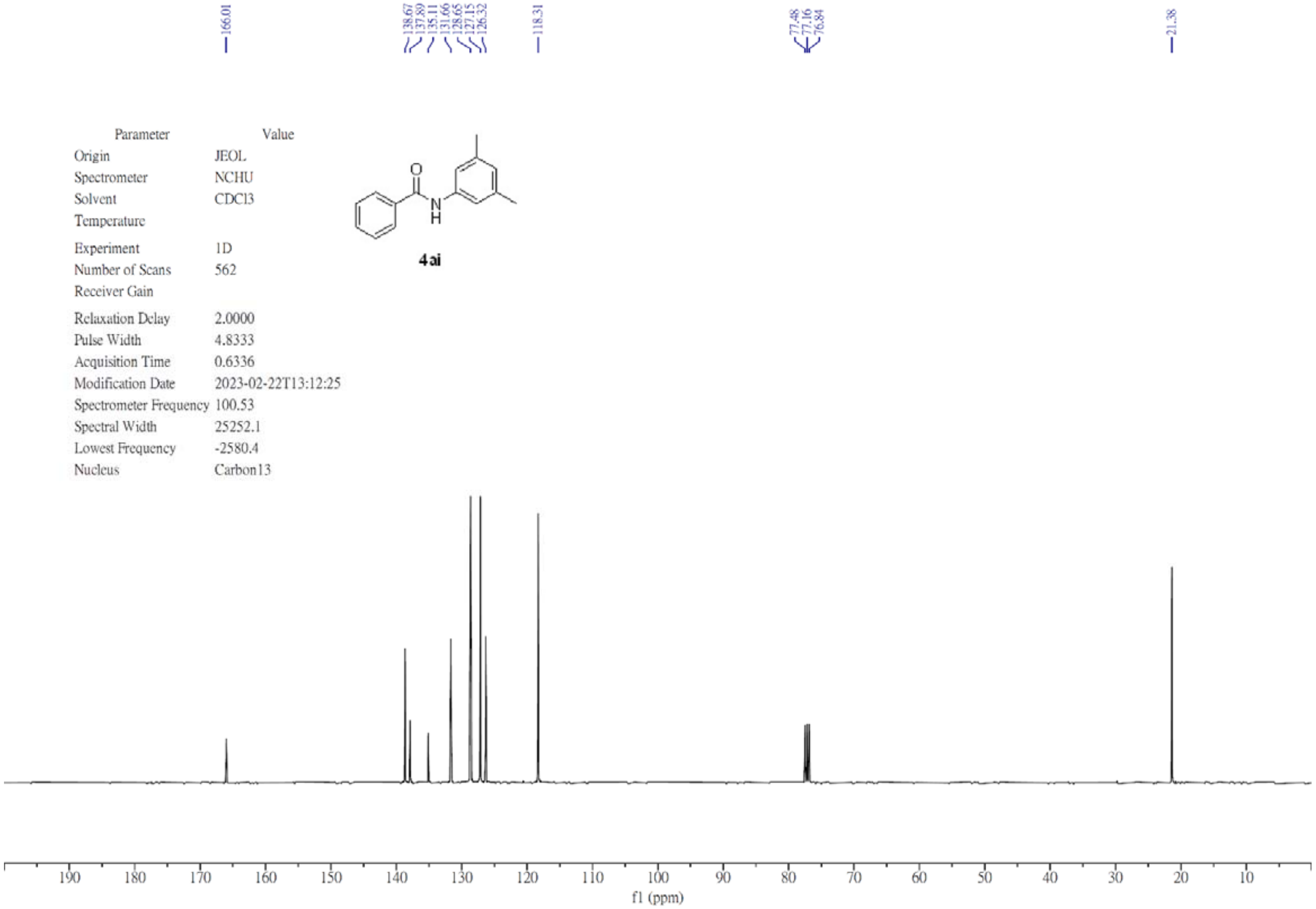

4ai $^{13}C\{^{1}H\}$ NMR spectrum (100 MHz in $CDCl_3$)

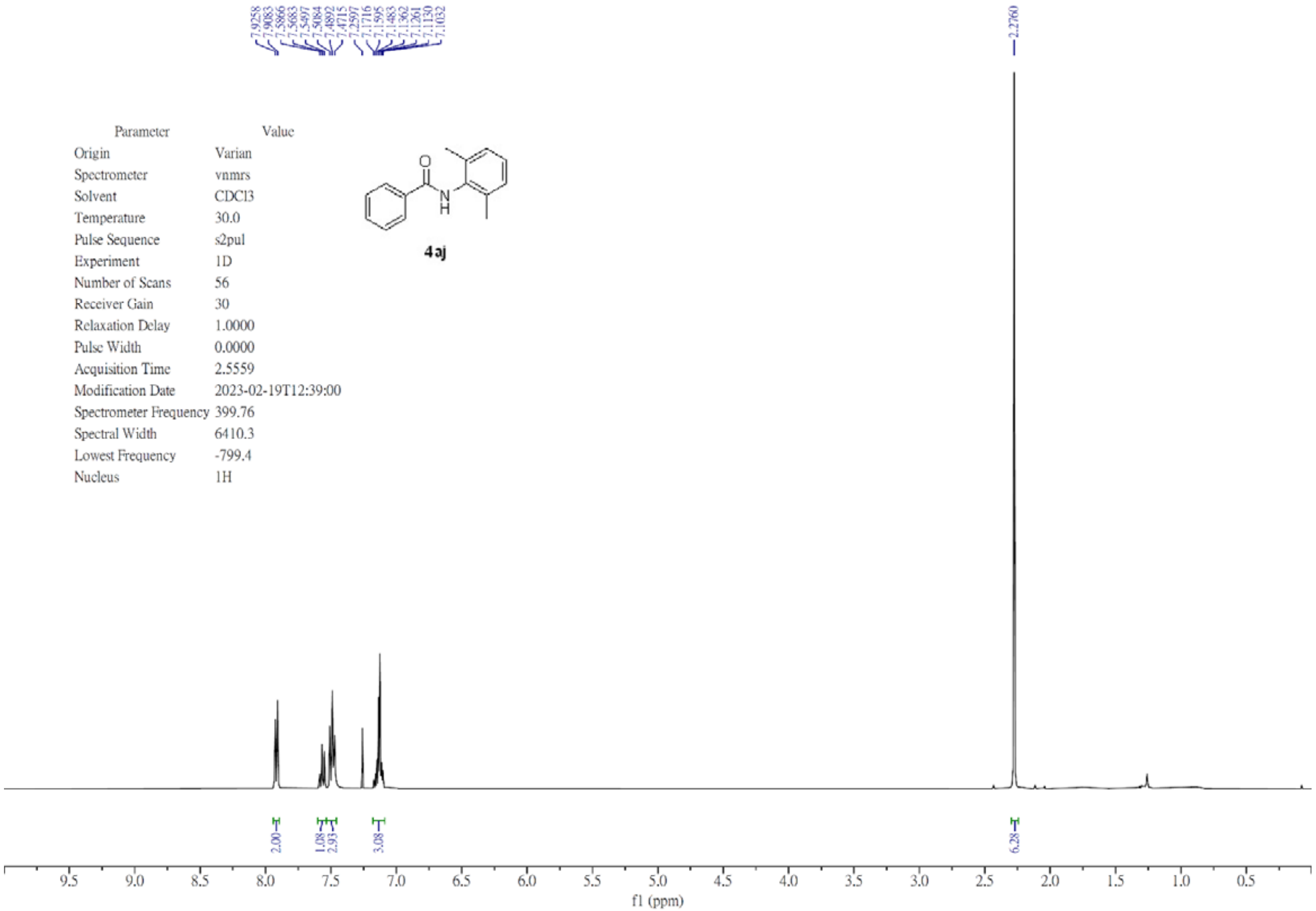

4aj ^{1}H NMR spectrum (400 MHz in $CDCl_3$)

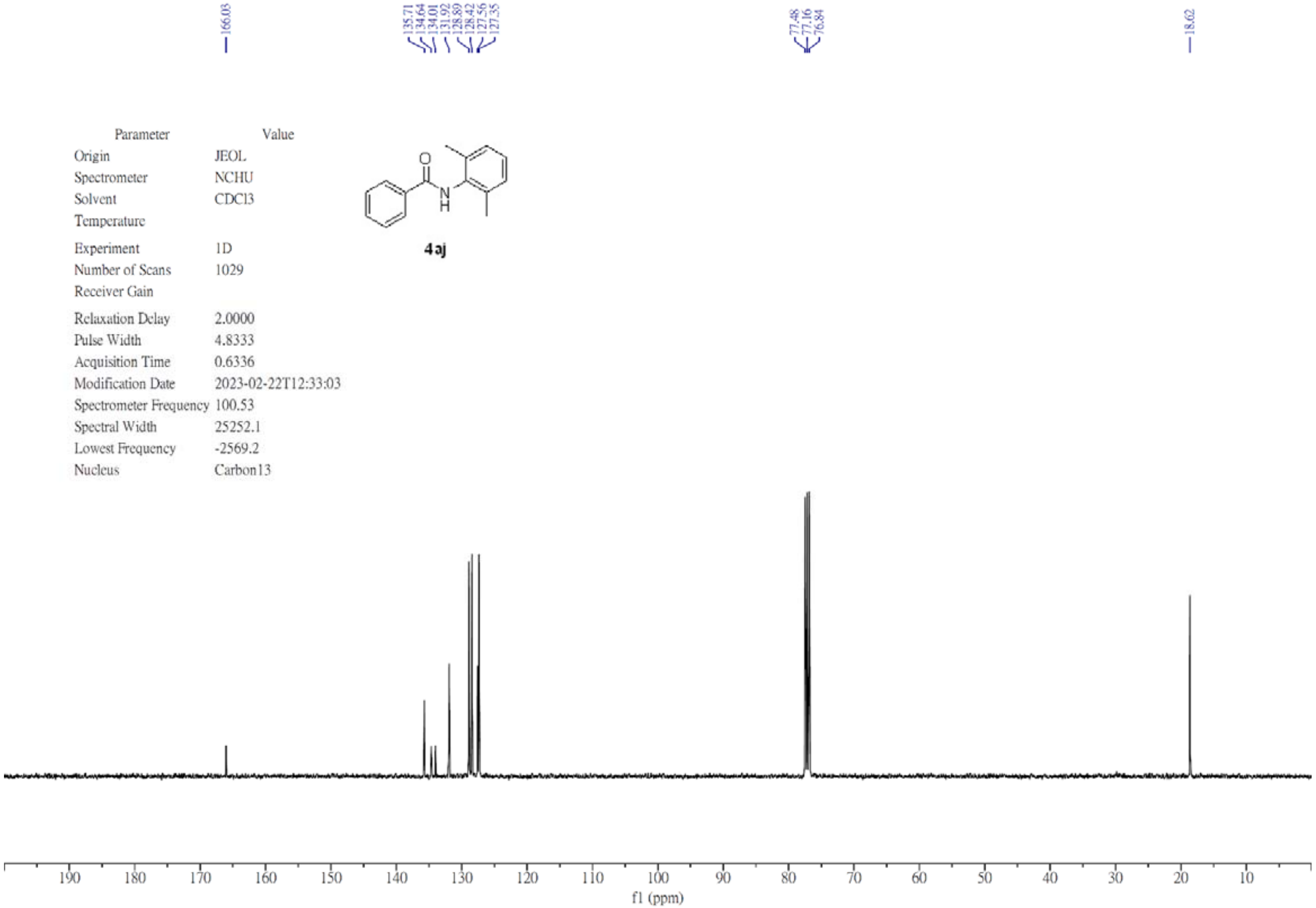

4aj $^{13}C\{^{1}H\}$ NMR spectrum (100 MHz in $CDCl_3$)

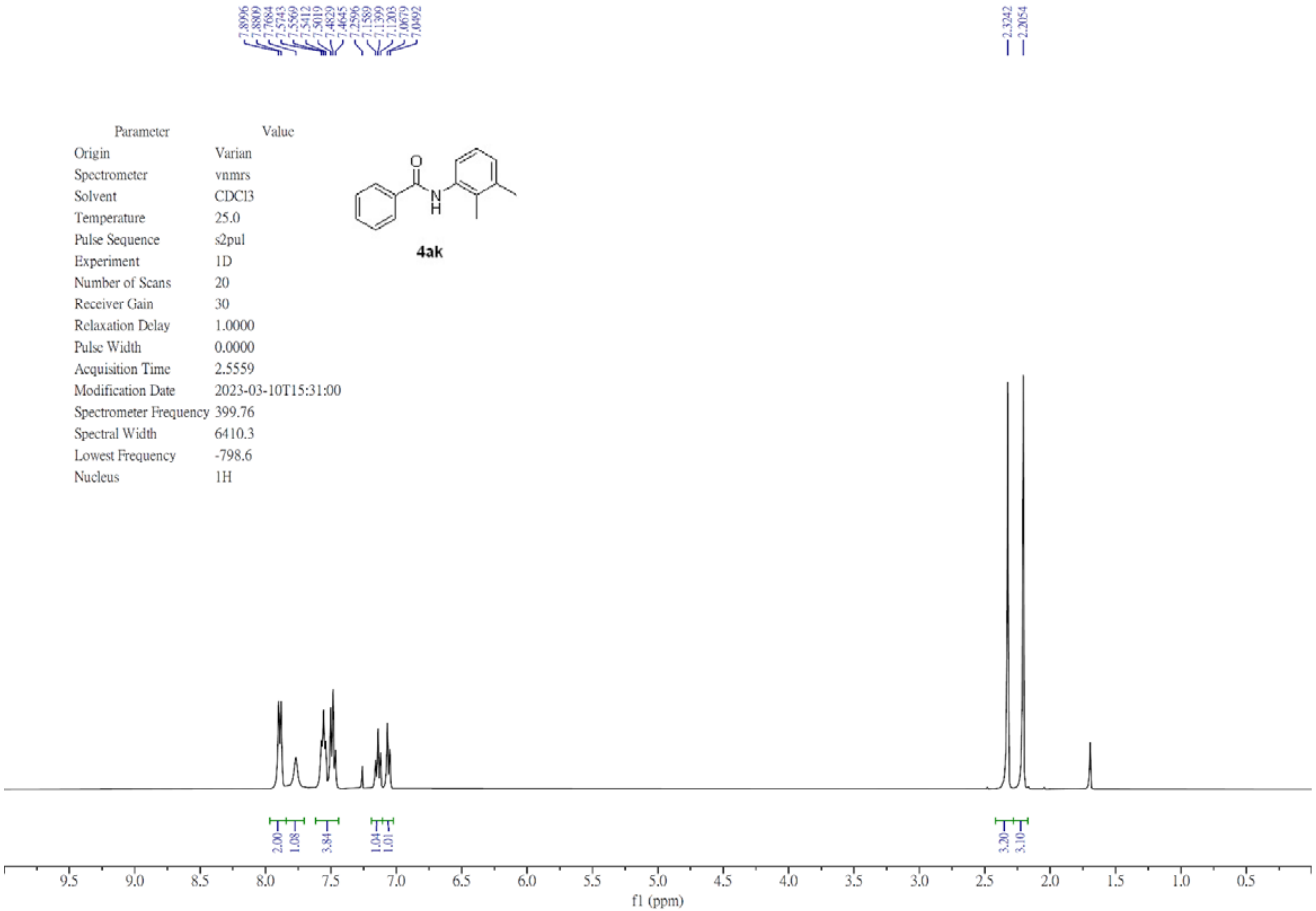

4ak ^{1}H NMR spectrum (400 MHz in $(CD_3)_2SO$)

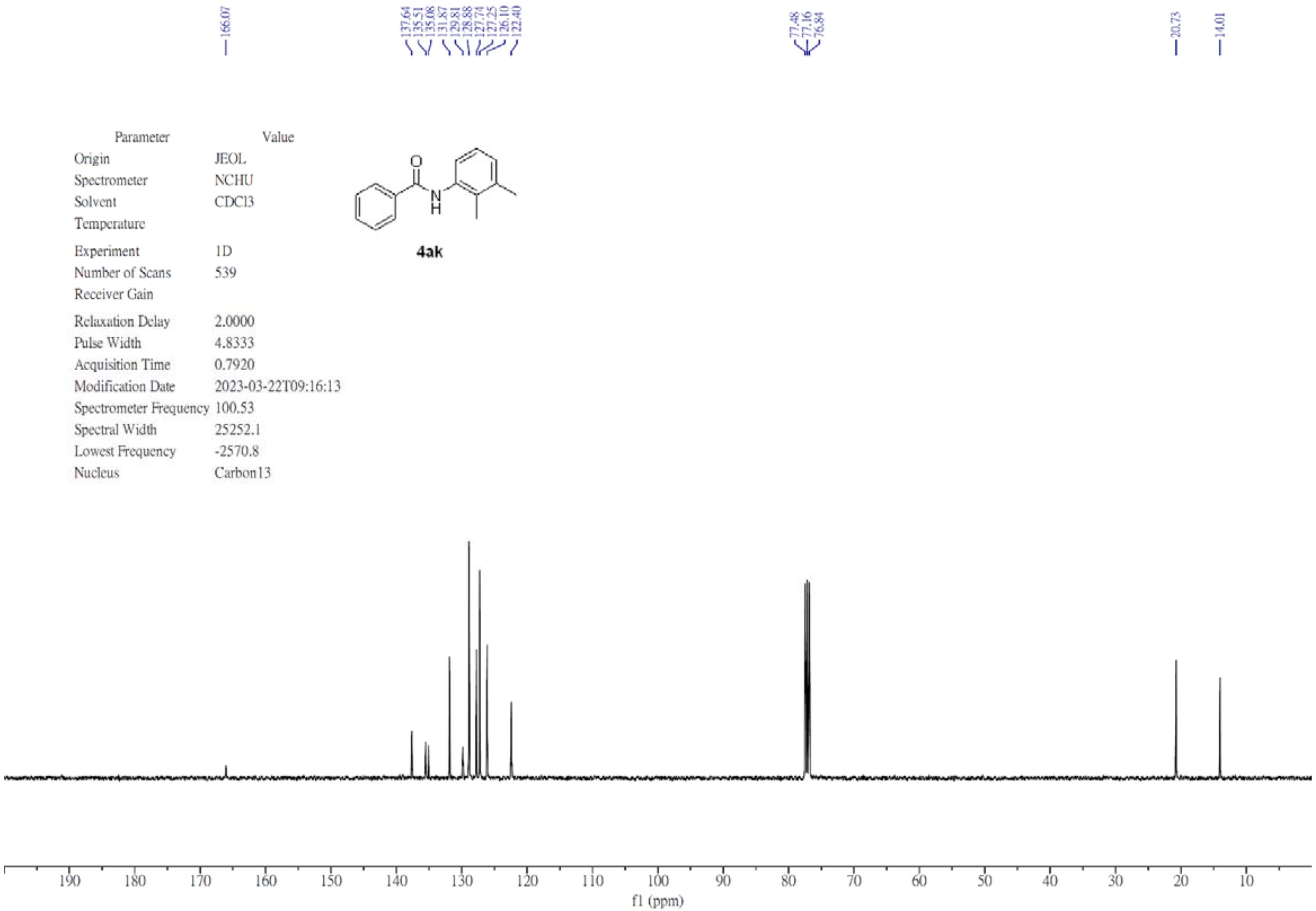

4ak $^{13}C\{^{1}H\}$ NMR spectrum (100 MHz in $(CD_3)_2SO$)

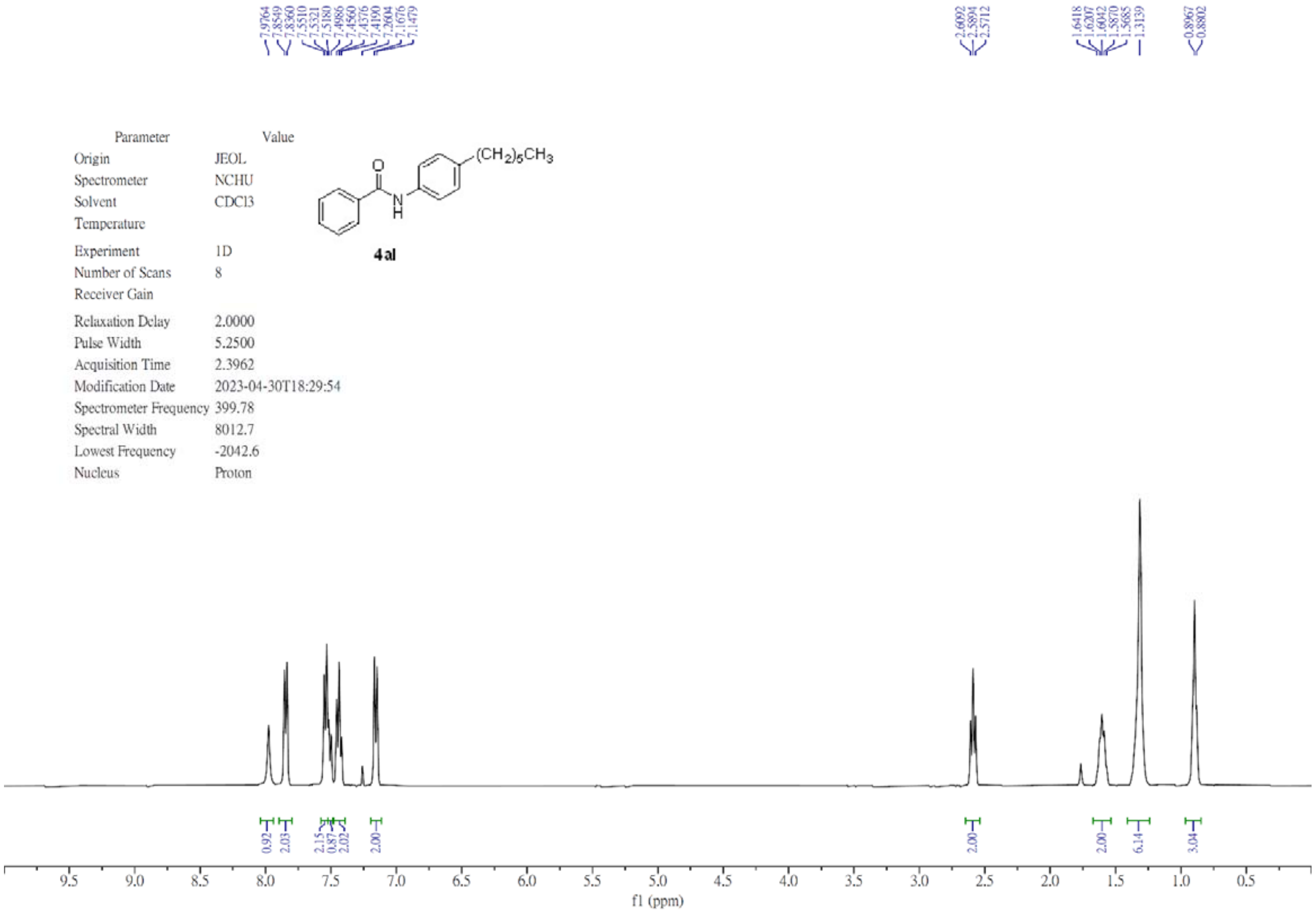

4al ^{1}H NMR spectrum (400 MHz in $CDCl_3$)

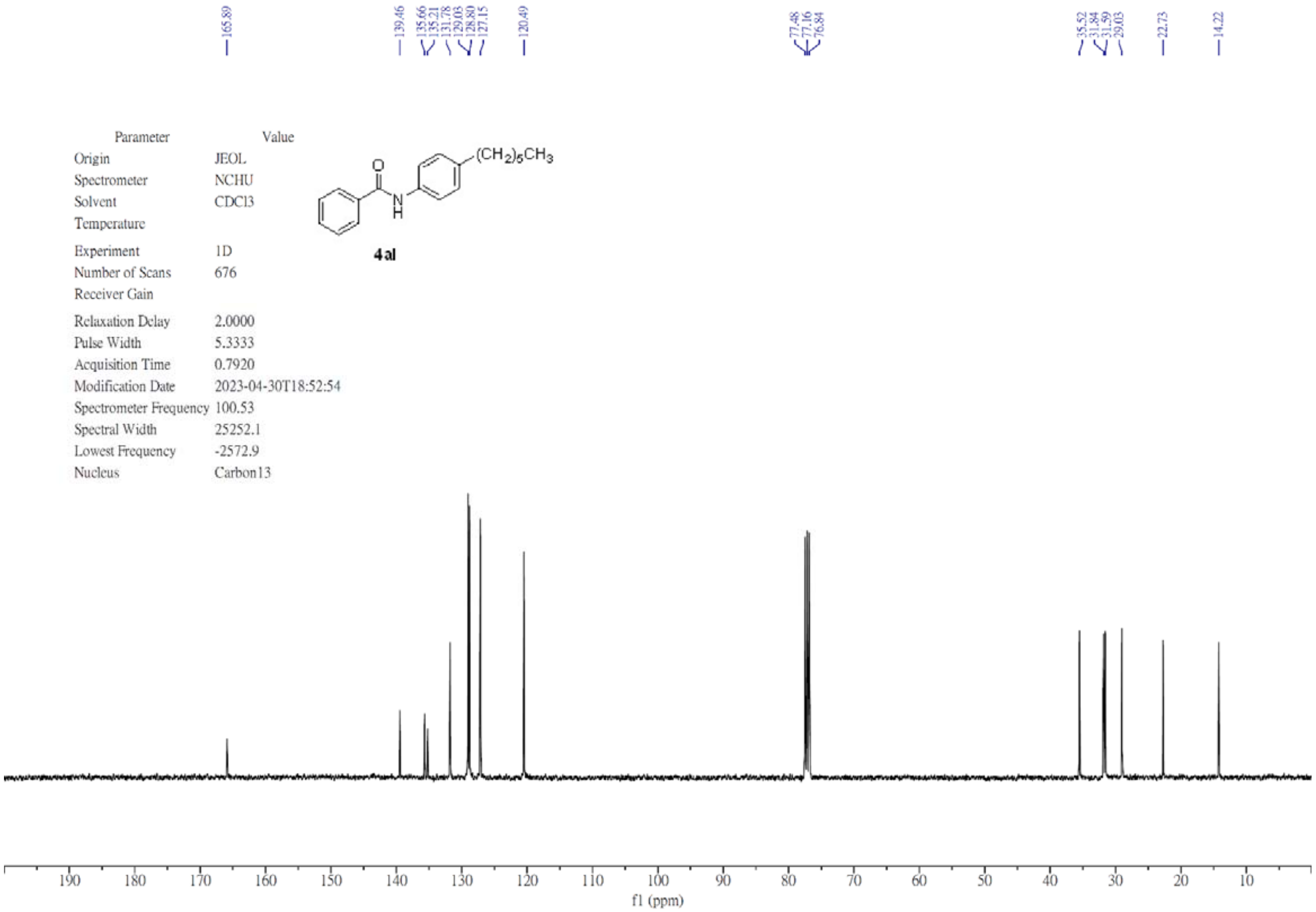

4al $^{13}C\{^{1}H\}$ NMR spectrum (100 MHz in $CDCl_3$)

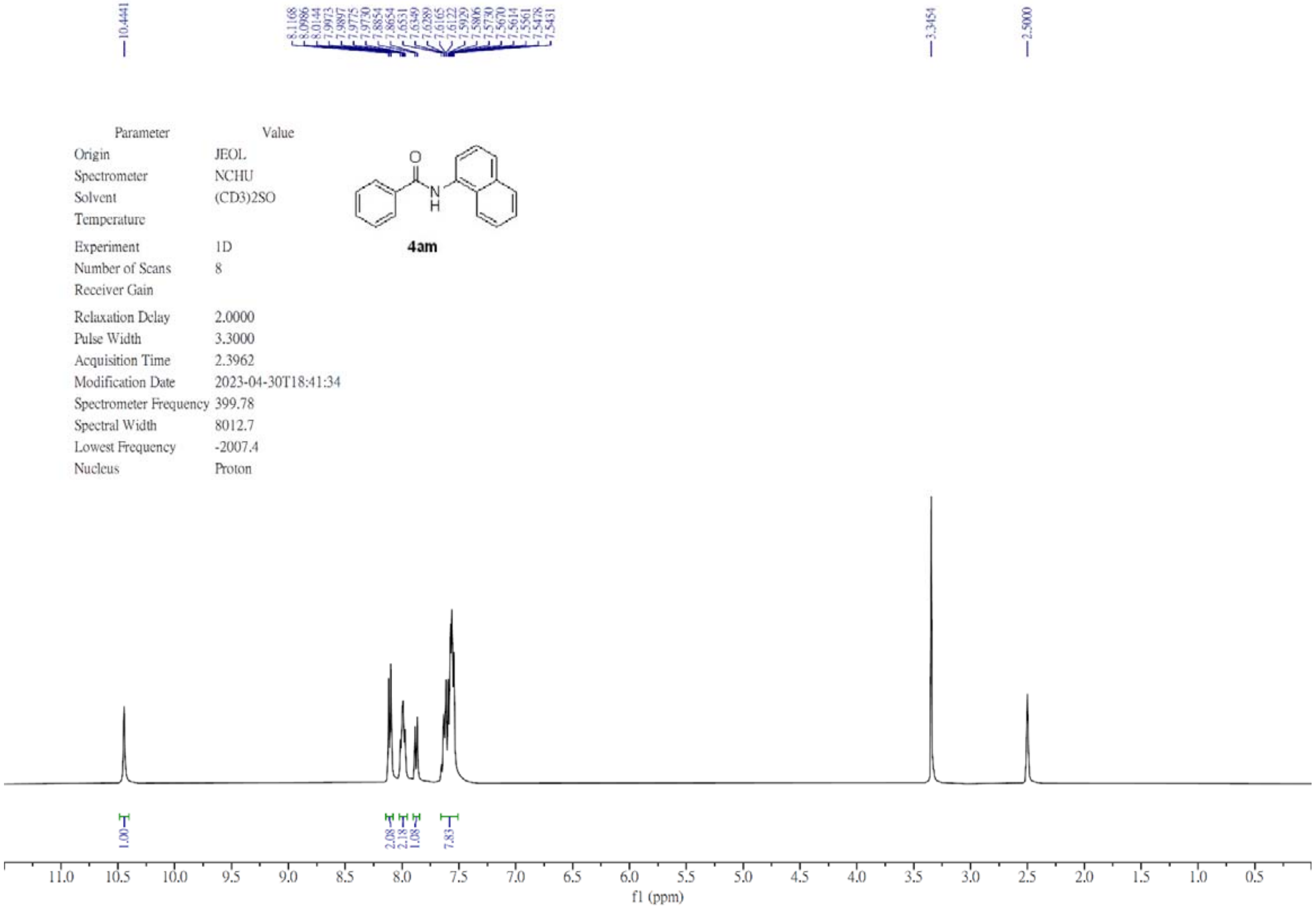

4am ^{1}H NMR spectrum (400 MHz in $(CD_3)_2SO$)

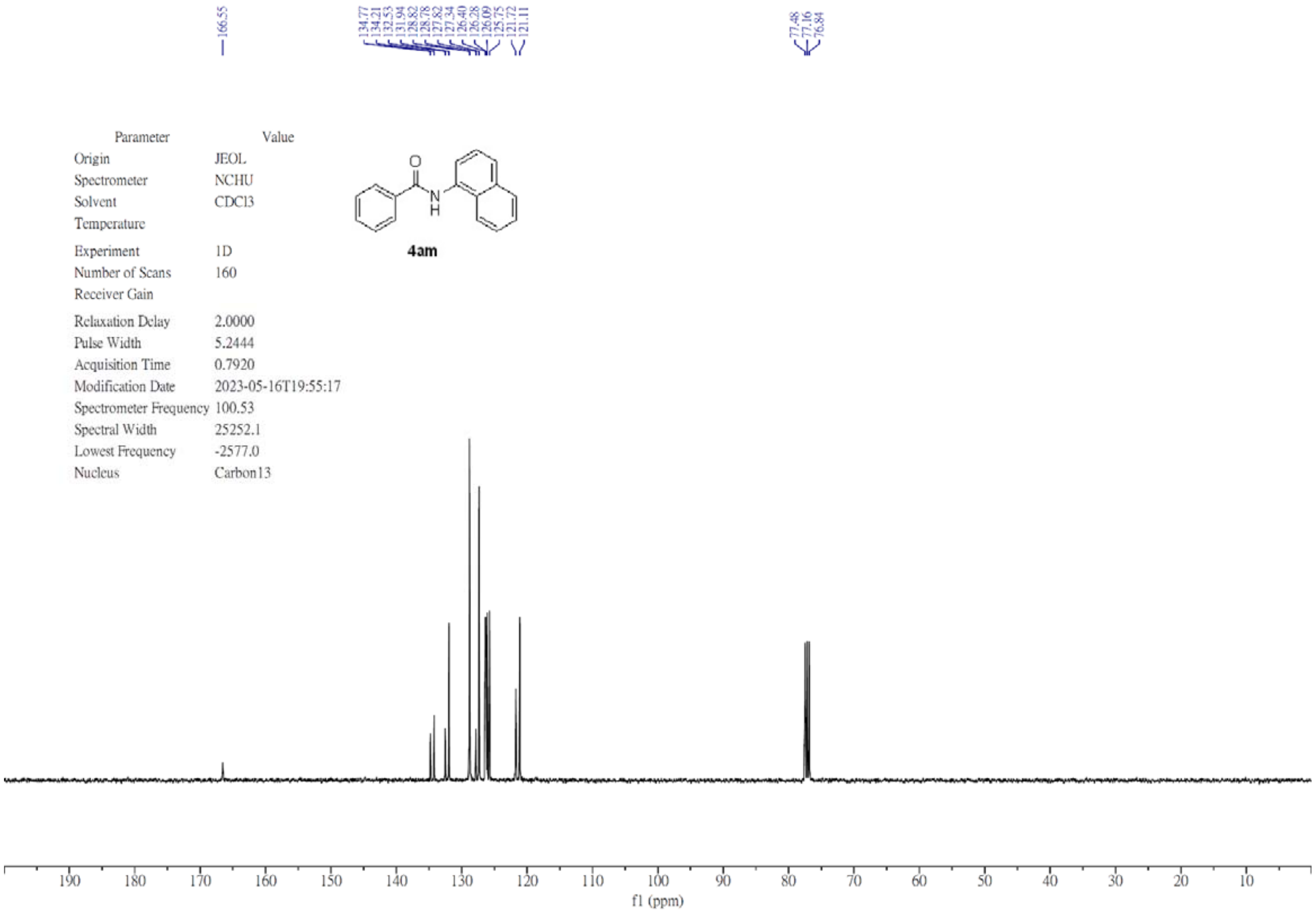

4am $^{13}C\{^{1}H\}$ NMR spectrum (100 MHz in $(CD_3)_2SO$)

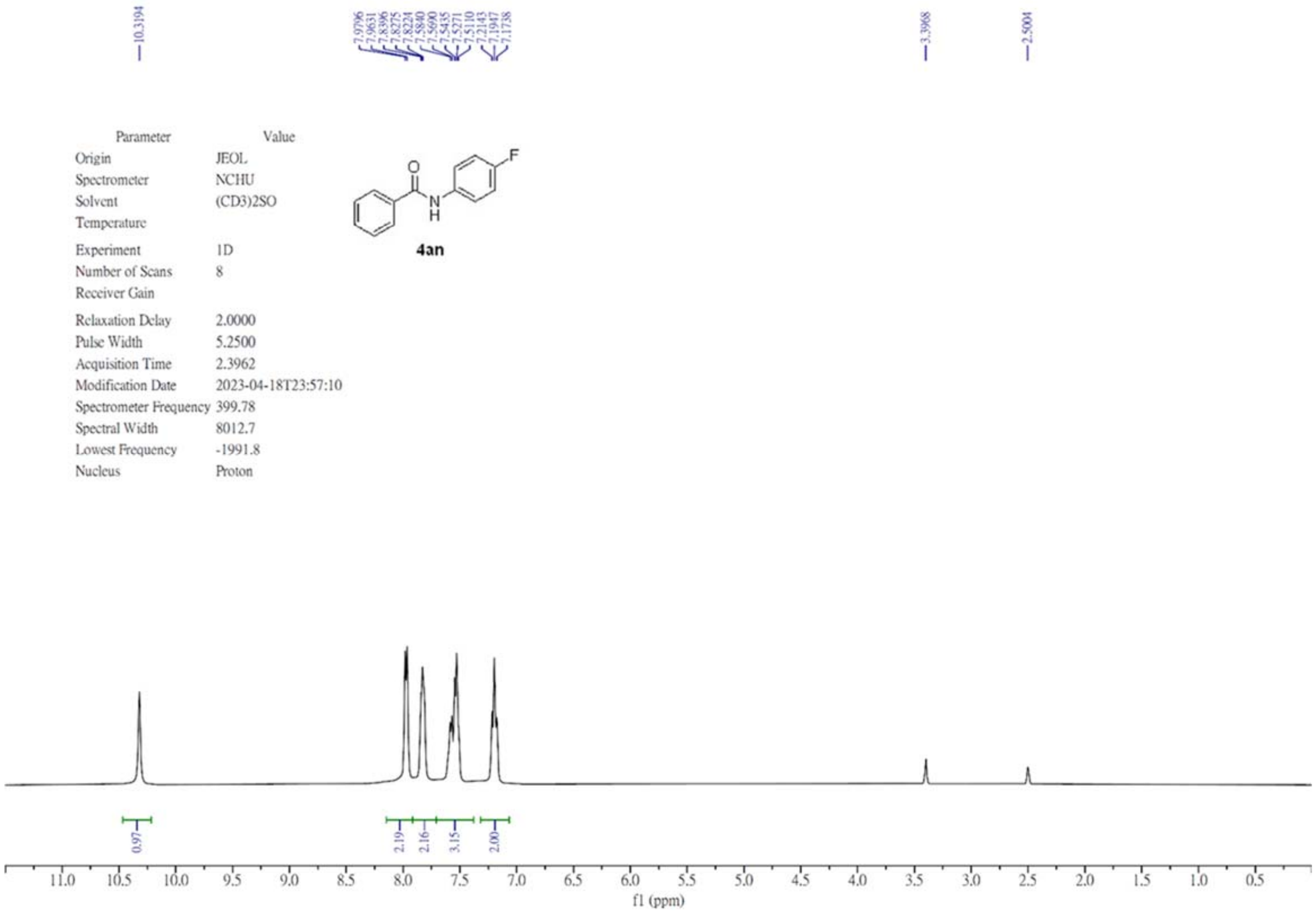

4an ^{1}H NMR spectrum (400 MHz in $CDCl_3$)

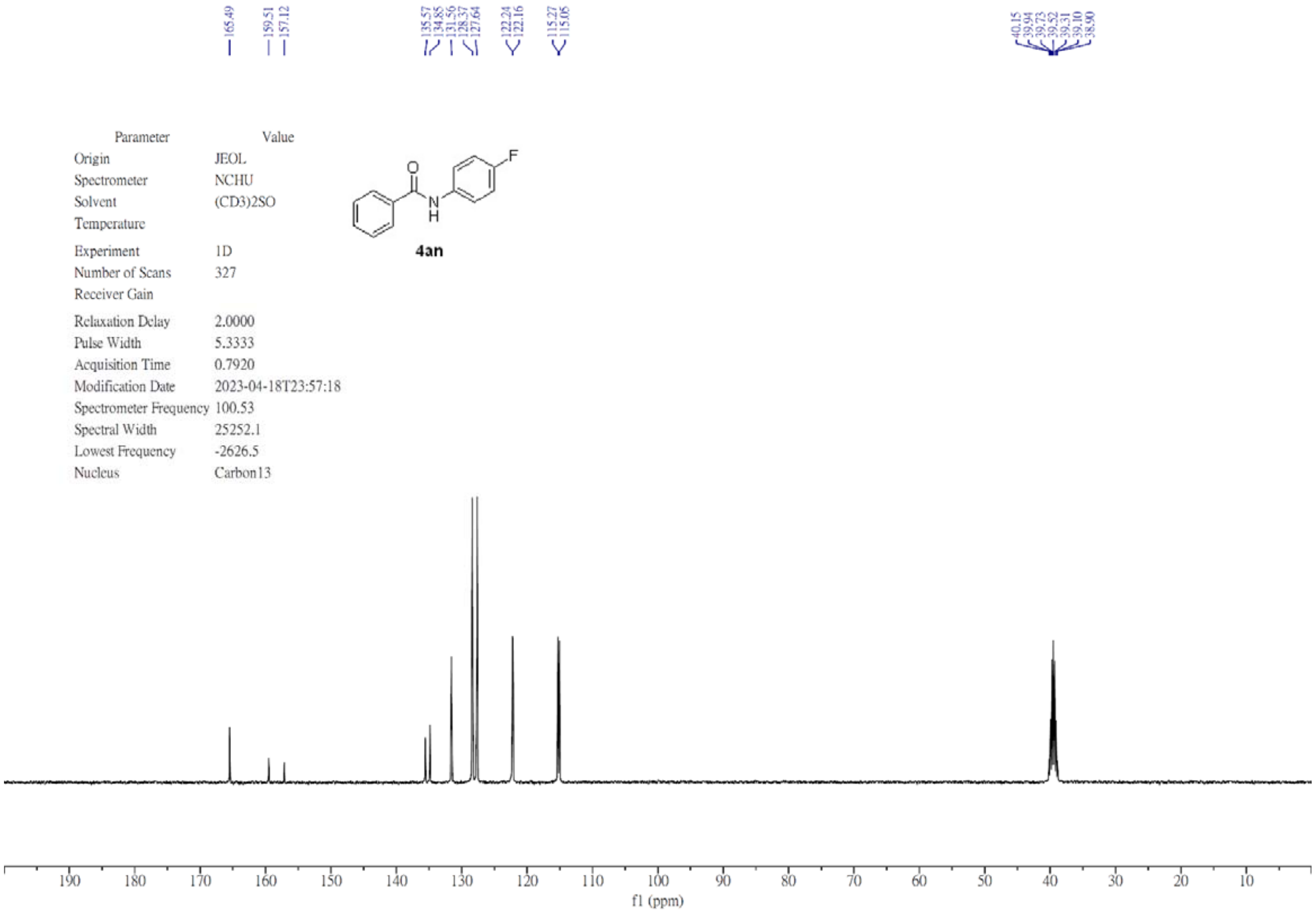

4an $^{13}C\{^1H\}$ NMR spectrum (100 MHz in $CDCl_3$)

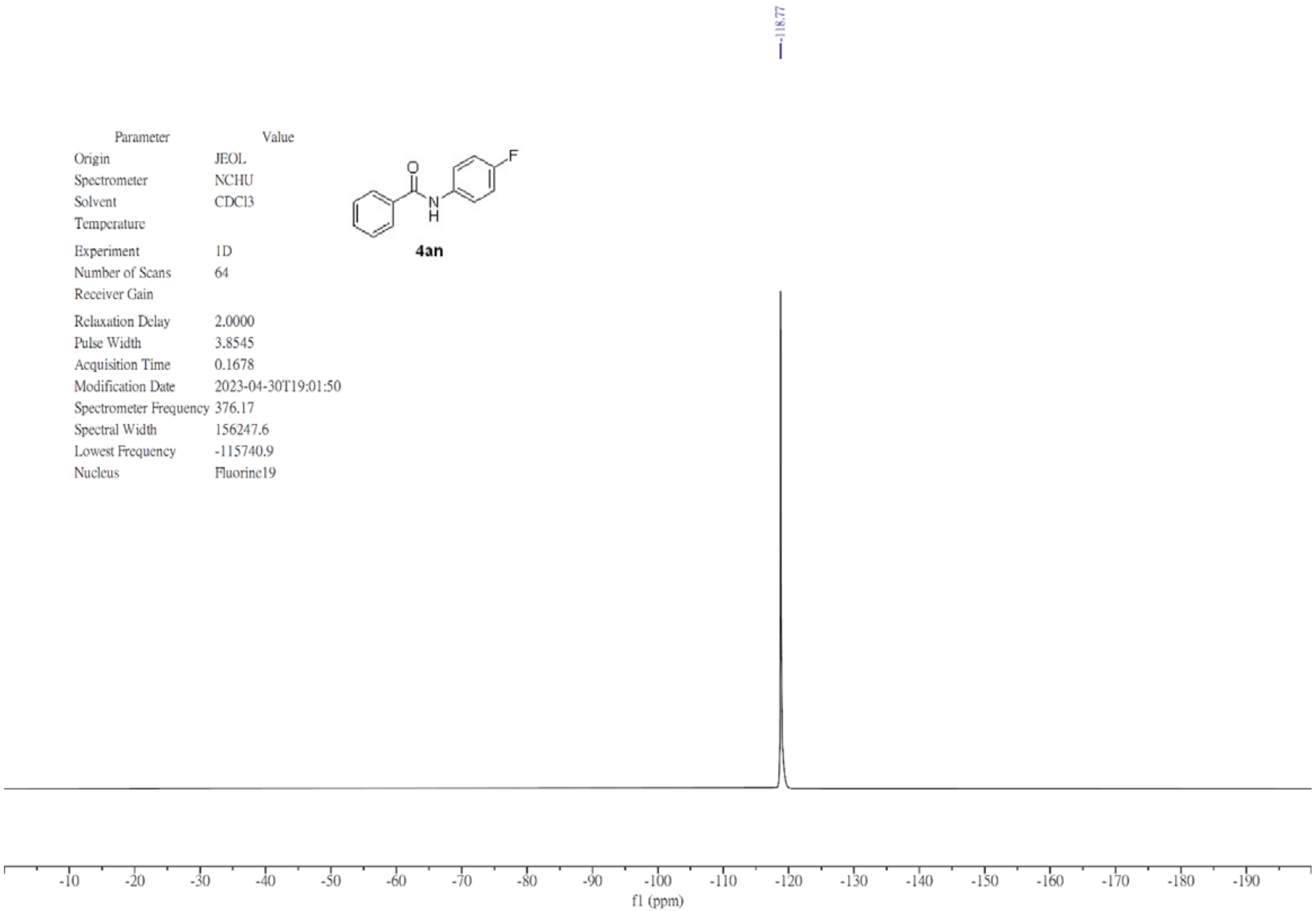

Parameter	Value
Origin	JEOL
Spectrometer	NCHU
Solvent	CDCl3
Temperature	
Experiment	1D
Number of Scans	64
Receiver Gain	
Relaxation Delay	2.0000
Pulse Width	3.8545
Acquisition Time	0.1678
Modification Date	2023-04-30T19:01:50
Spectrometer Frequency	376.17
Spectral Width	156247.6
Lowest Frequency	-115740.9
Nucleus	Fluorine19

4an

4an ^{19}F NMR spectrum (376 MHz, $CDCl_3$)

Parameter	Value
Origin	JEOL
Spectrometer	NCHU
Solvent	CDCl3
Temperature	
Experiment	1D
Number of Scans	8
Receiver Gain	
Relaxation Delay	2.0000
Pulse Width	4.1700
Acquisition Time	2.3962
Modification Date	2023-01-17T16:50:24
Spectrometer Frequency	399.78
Spectral Width	8012.7
Lowest Frequency	-2003.8
Nucleus	Proton

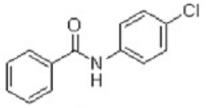

4ao

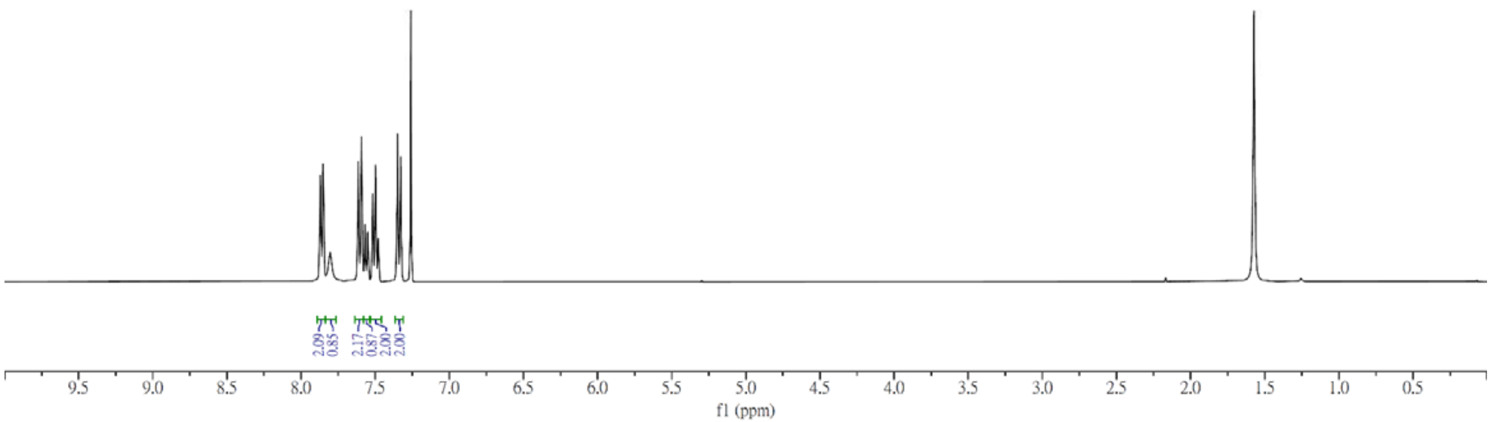

4ao ^{1}H NMR spectrum (400 MHz in $CDCl_3$)

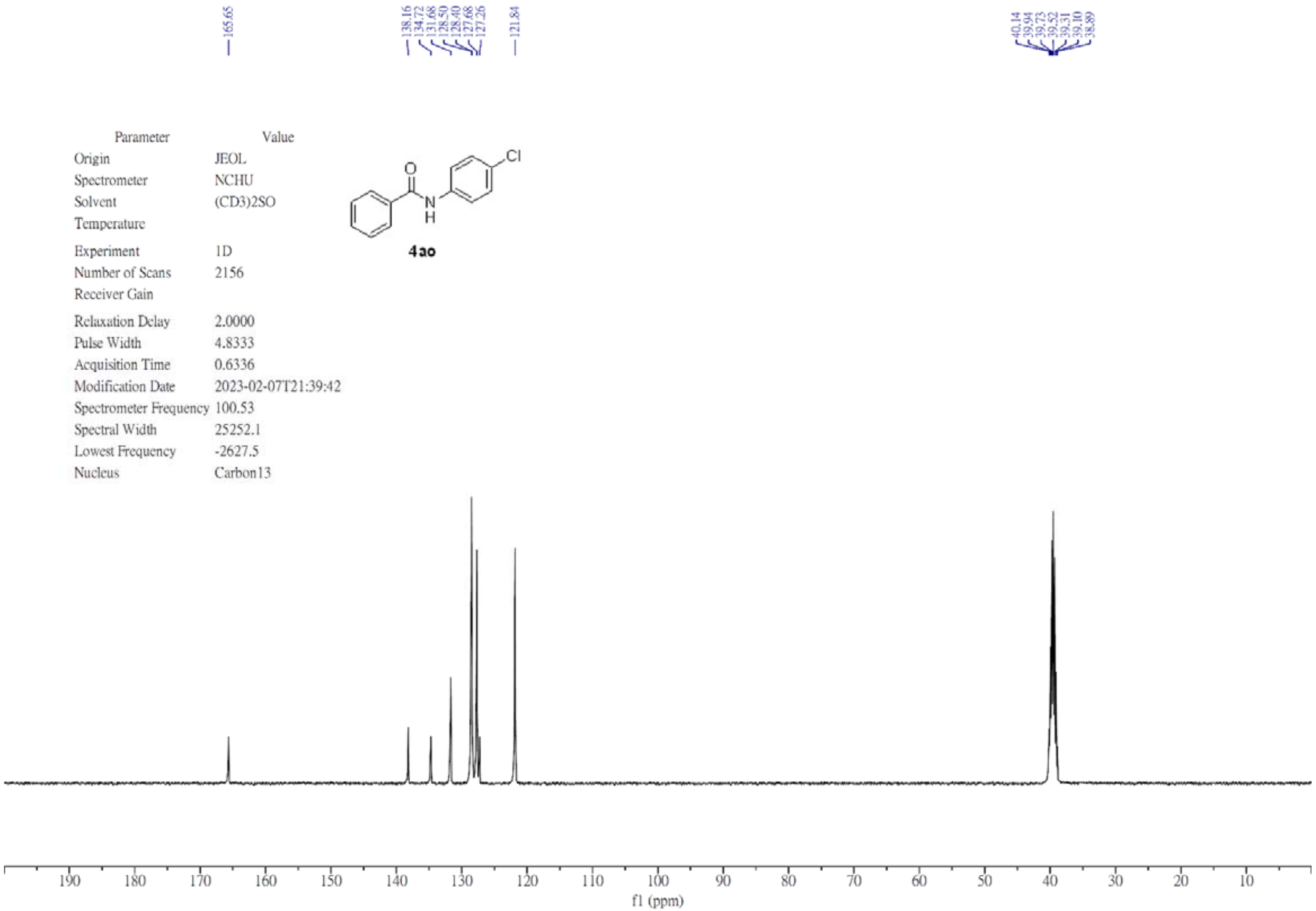

4ao $^{13}C\{^{1}H\}$ NMR spectrum (100 MHz in $CDCl_3$)

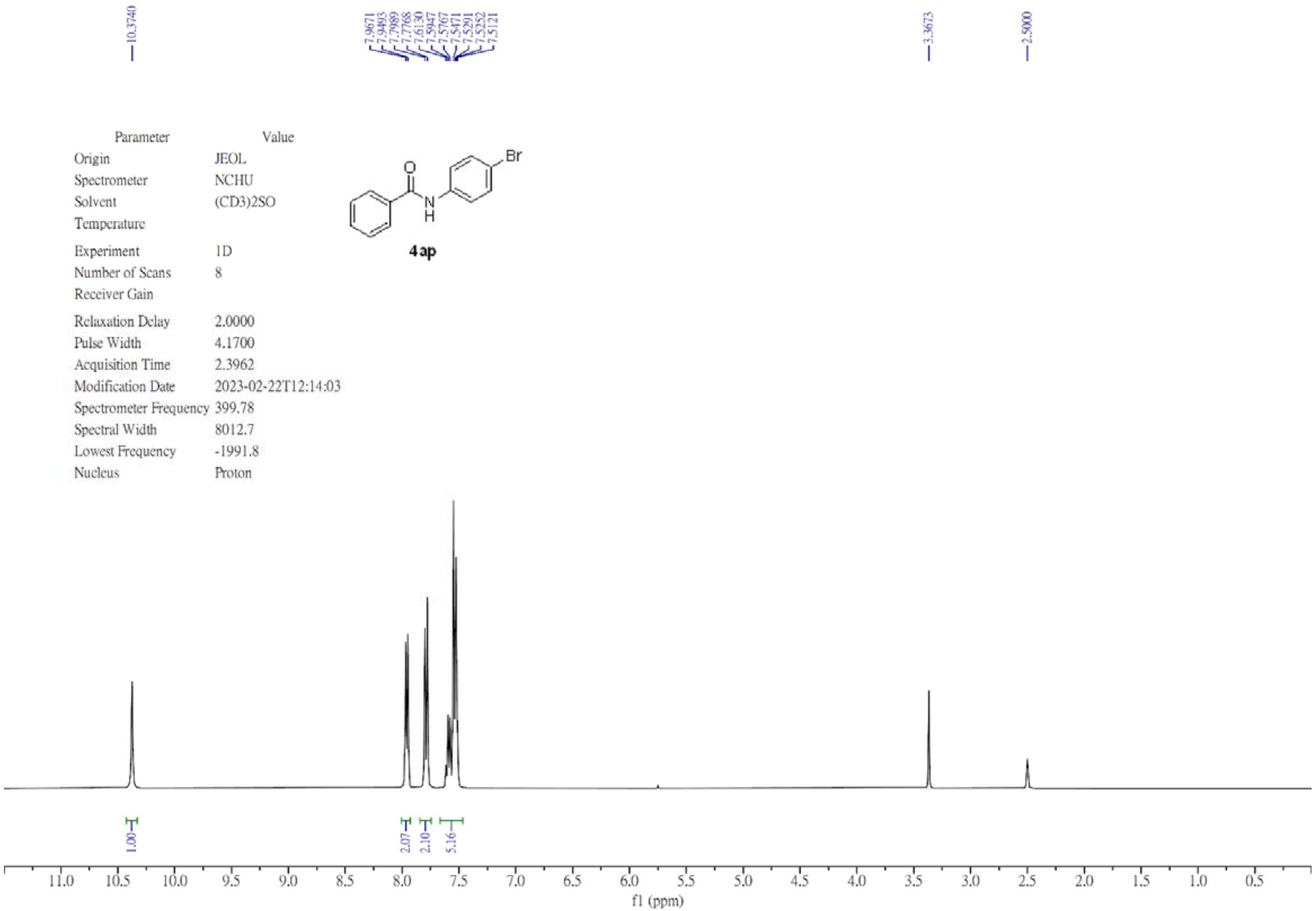

4ap ^{1}H NMR spectrum (400 MHz in $(CD_3)_2SO$)

Parameter	Value
Origin	JEOL
Spectrometer	NCHU
Solvent	(CD3)2SO
Temperature	
Experiment	1D
Number of Scans	1027
Receiver Gain	
Relaxation Delay	2.0000
Pulse Width	4.8333
Acquisition Time	0.6336
Modification Date	2023-02-22T12:14:08
Spectrometer Frequency	100.53
Spectral Width	25252.1
Lowest Frequency	-2628.3
Nucleus	Carbon13

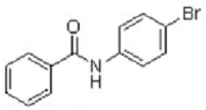

4ap

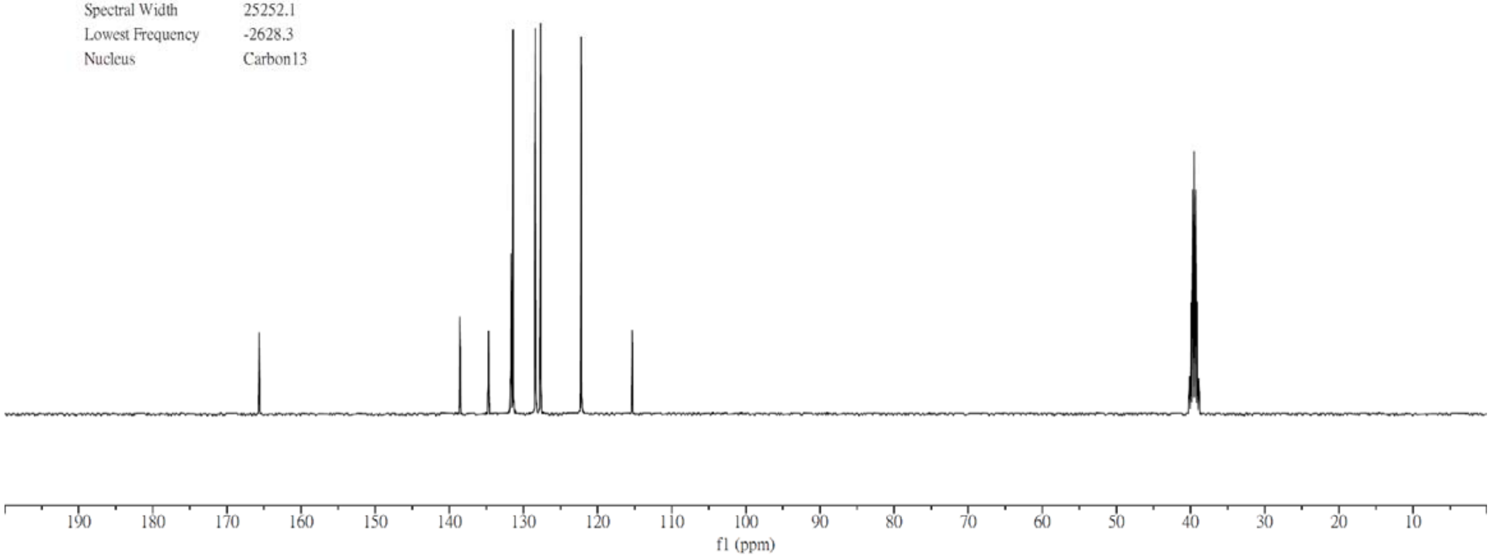

4ap $^{13}C\{^{1}H\}$ NMR spectrum (100 MHz in $(CD_3)_2SO$)

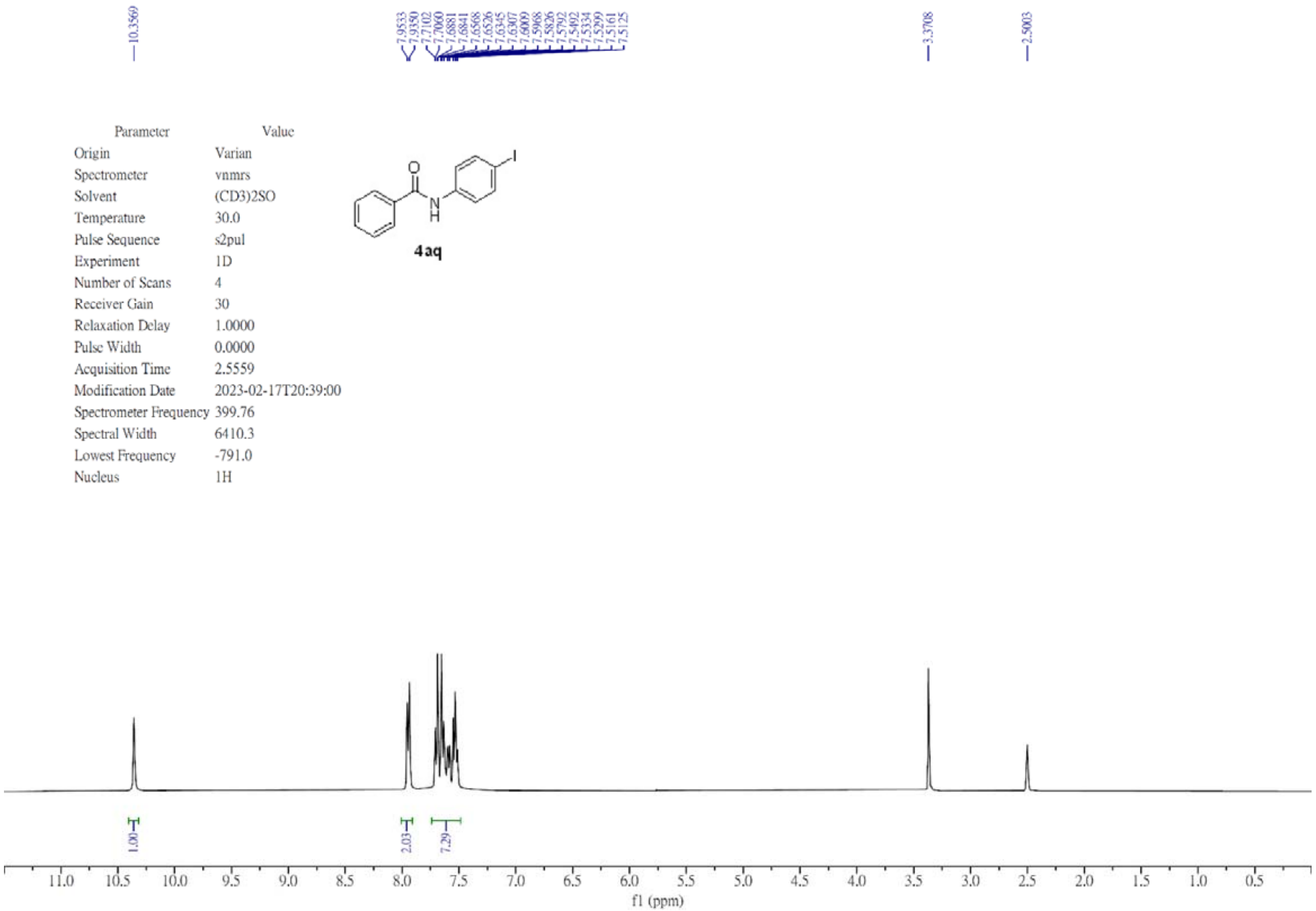

4aq ^{1}H NMR spectrum (400 MHz in $CDCl_3$)

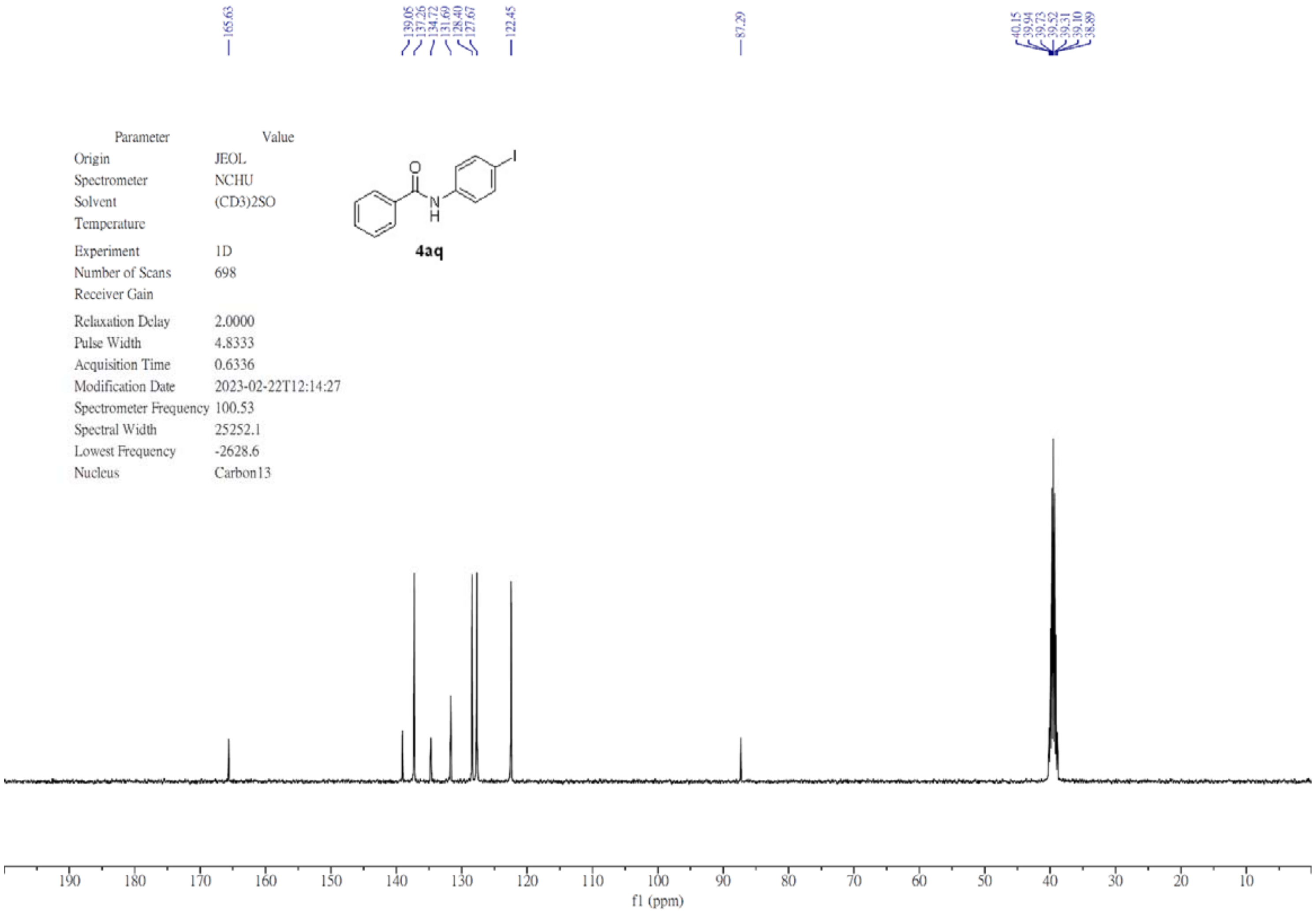

4aq $^{13}C\{^{1}H\}$ NMR spectrum (100 MHz in $CDCl_3$)

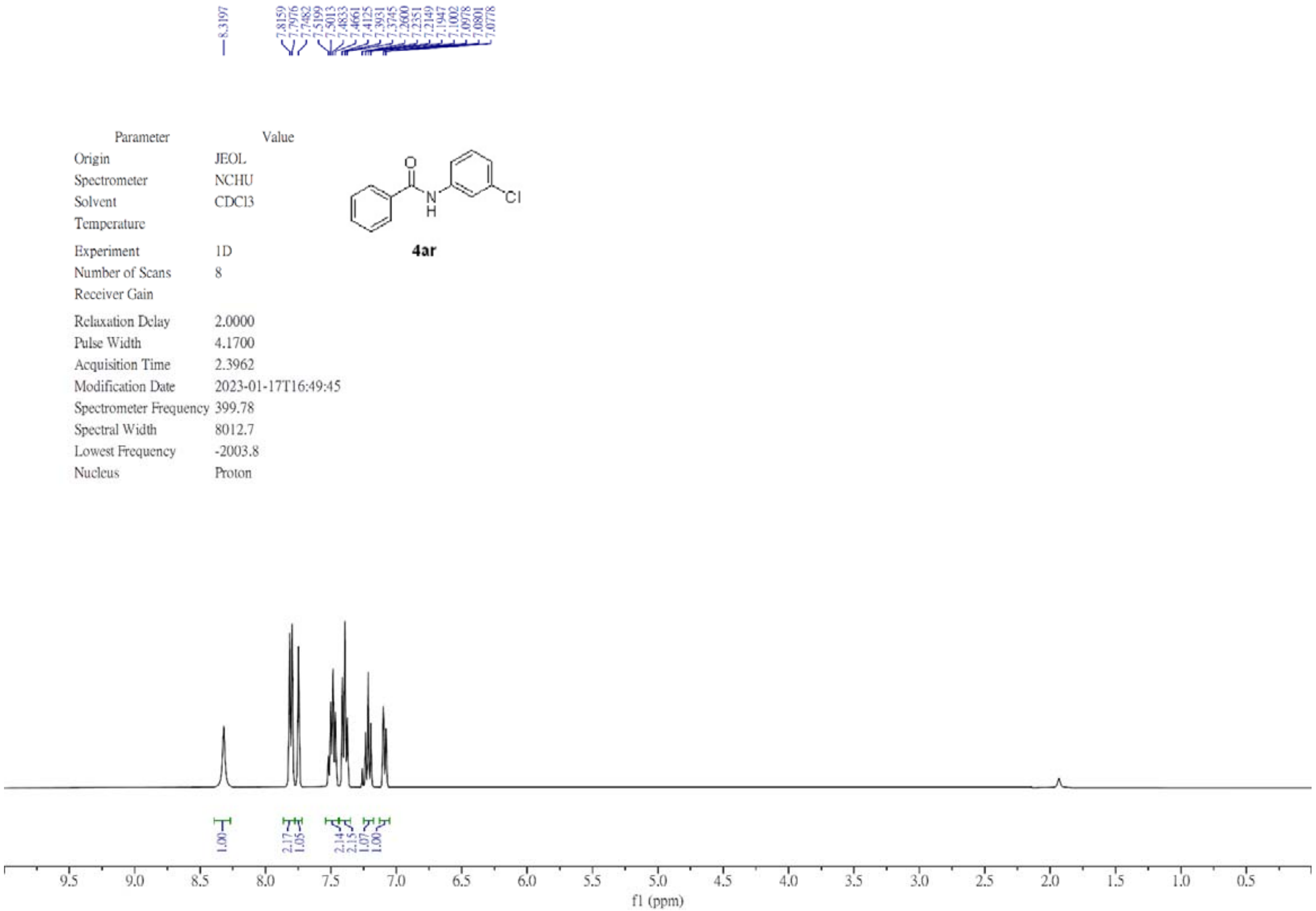

4ar ^{1}H NMR spectrum (400 MHz in $CDCl_3$)

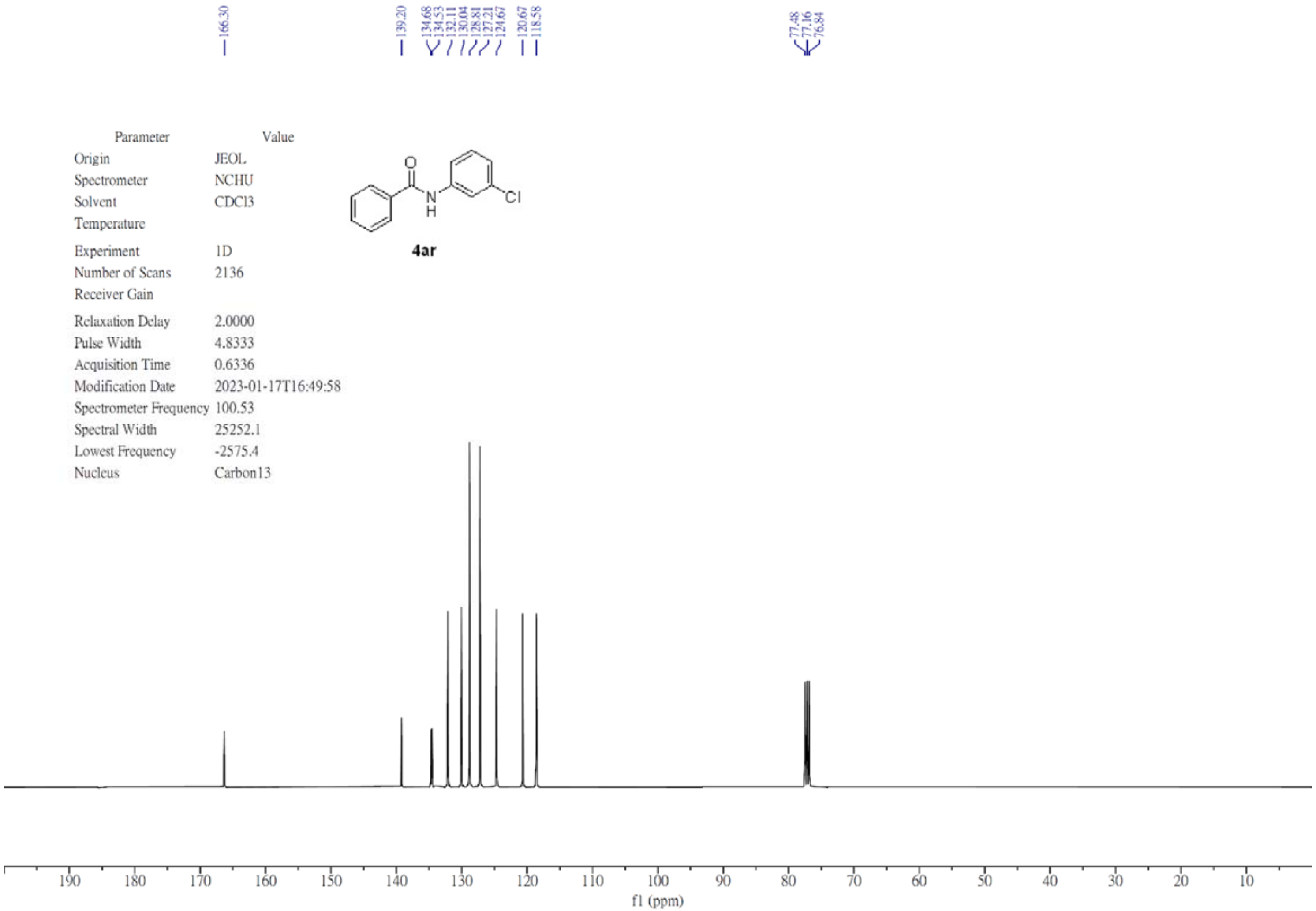

4ar $^{13}C\{^{1}H\}$ NMR spectrum (100 MHz in $CDCl_3$)

Parameter	Value
Origin	Varian
Spectrometer	vnmrs
Solvent	CDCl3
Temperature	25.0
Pulse Sequence	s2pul
Experiment	1D
Number of Scans	8
Receiver Gain	30
Relaxation Delay	1.0000
Pulse Width	0.0000
Acquisition Time	2.5559
Modification Date	2023-03-07T15:25:00
Spectrometer Frequency	399.76
Spectral Width	6410.3
Lowest Frequency	-799.0
Nucleus	1H

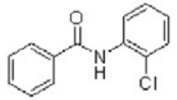

4as

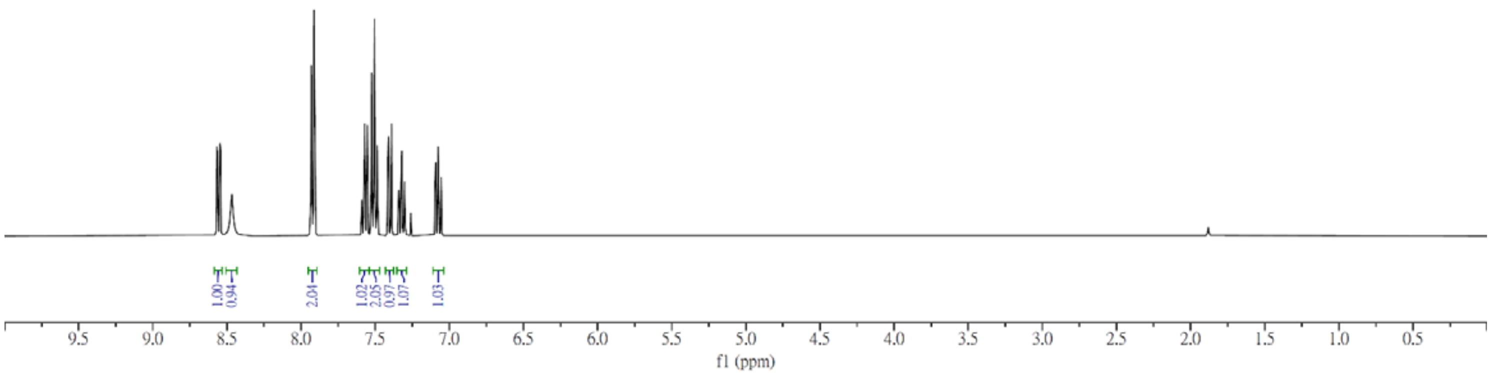

4as ^{1}H NMR spectrum (400 MHz in $CDCl_3$)

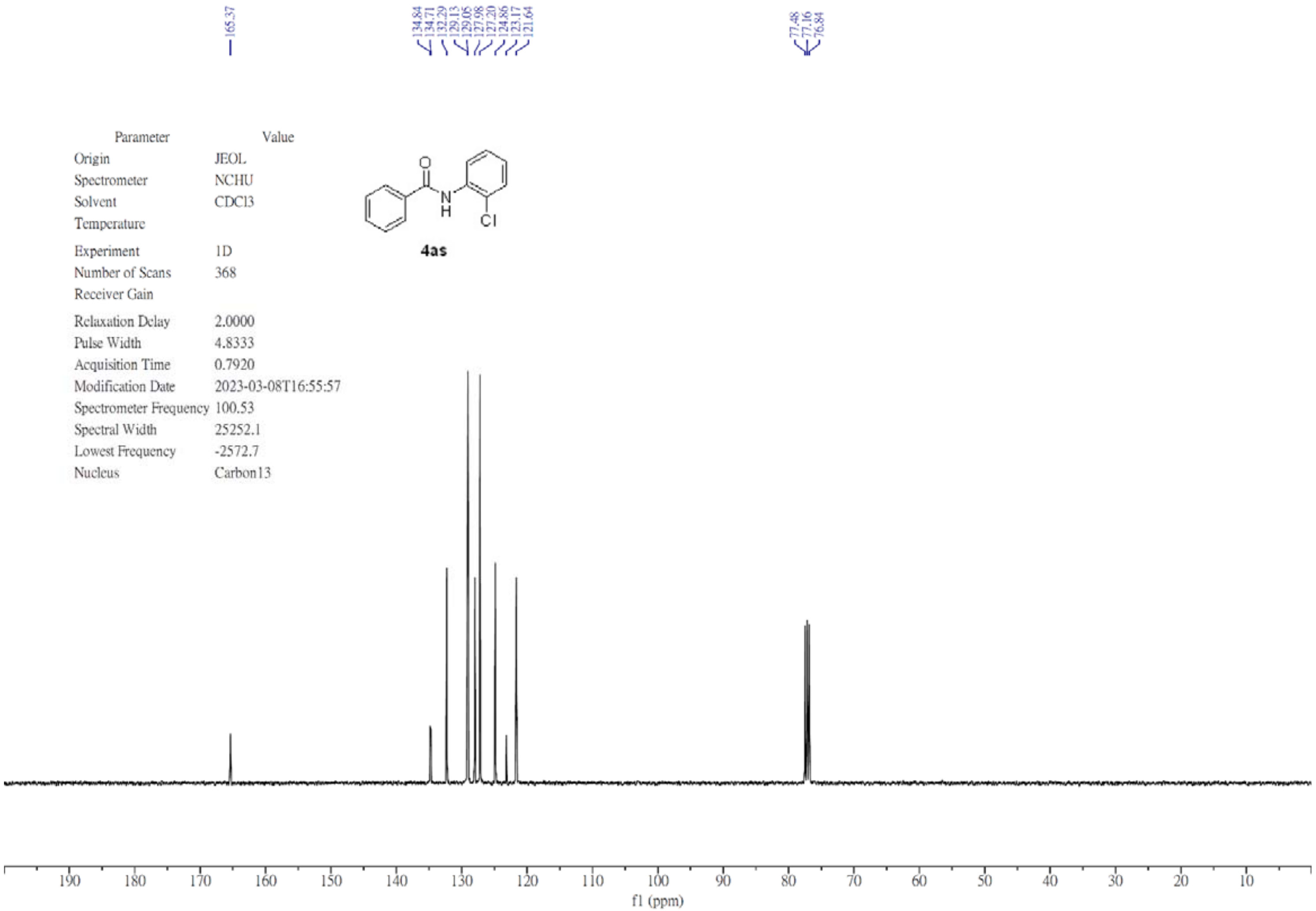

4as $^{13}C\{^{1}H\}$ NMR spectrum (100 MHz in $CDCl_3$)

Parameter	Value
Origin	Varian
Spectrometer	vnmrs
Solvent	CDCl3
Temperature	25.0
Pulse Sequence	s2pul
Experiment	1D
Number of Scans	8
Receiver Gain	30
Relaxation Delay	1.0000
Pulse Width	0.0000
Acquisition Time	2.5559
Modification Date	2023-03-07T15:24:00
Spectrometer Frequency	399.76
Spectral Width	6410.3
Lowest Frequency	-799.0
Nucleus	1H

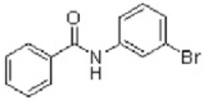

4at

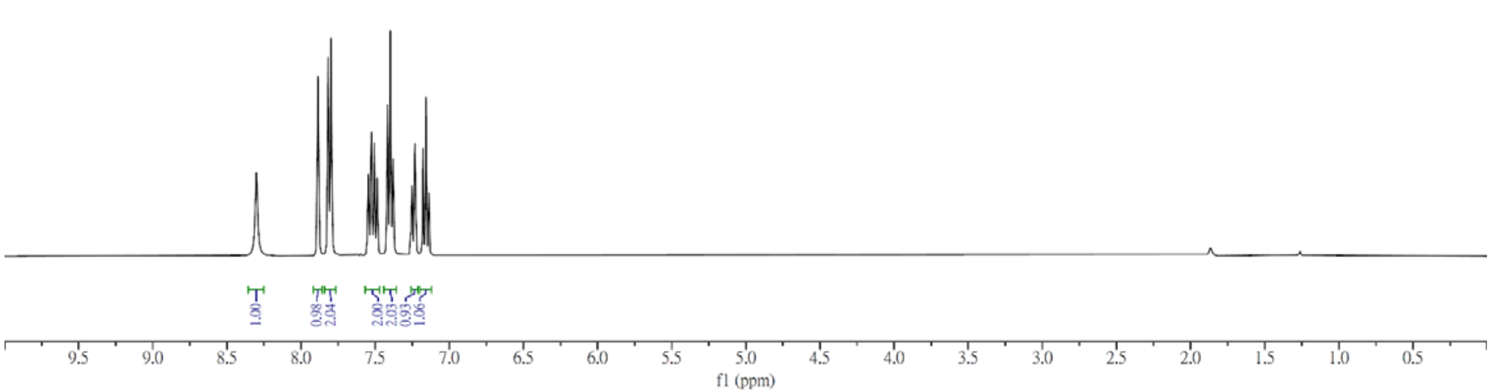

4at ^{1}H NMR spectrum (400 MHz in $CDCl_3$)

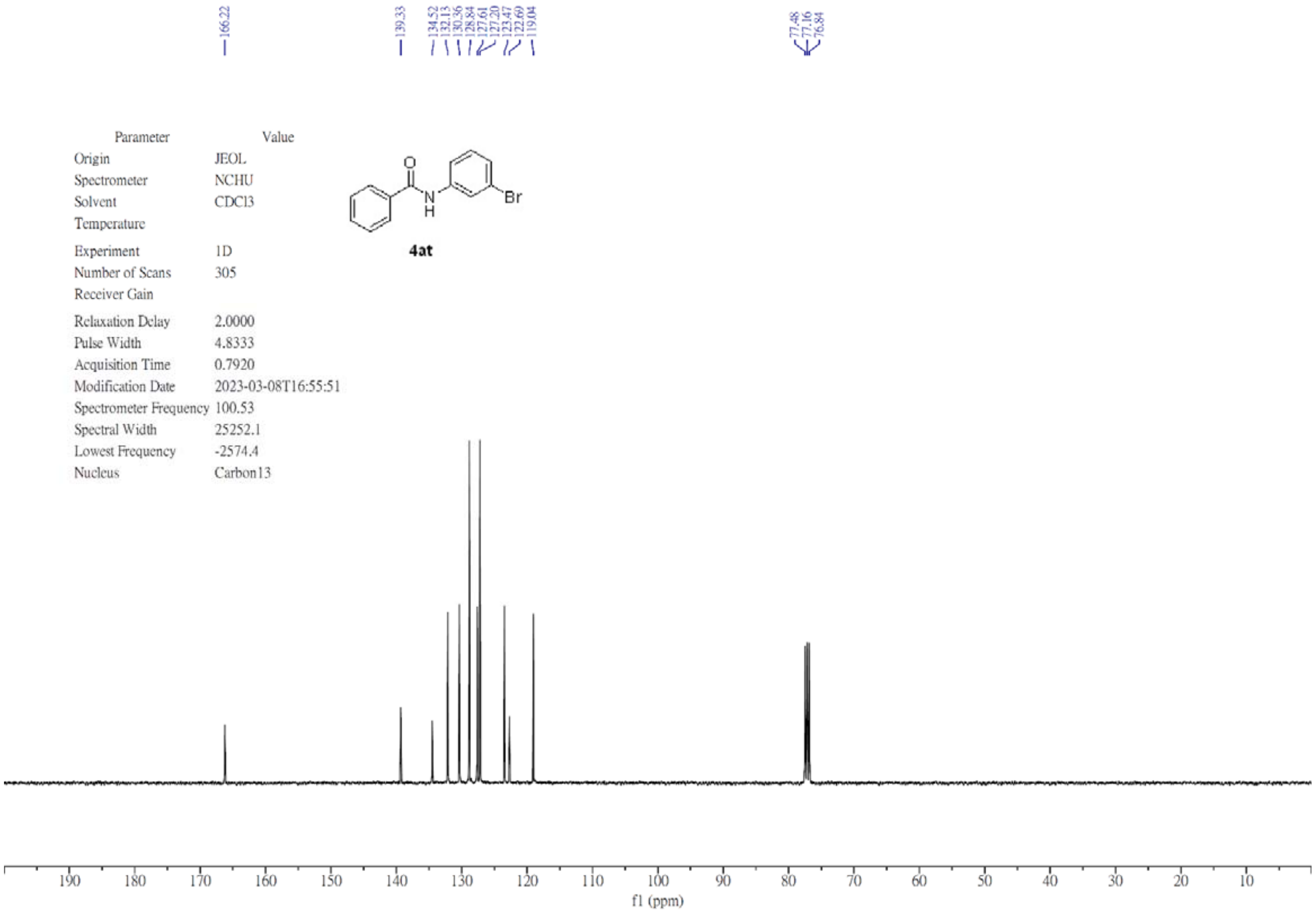

4at $^{13}C\{^{1}H\}$ NMR spectrum (100 MHz in $CDCl_3$)

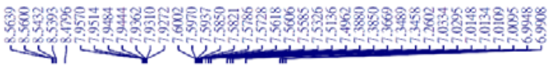

Parameter	Value
Origin	Varian
Spectrometer	vnmrs
Solvent	CDCl3
Temperature	30.0
Pulse Sequence	s2pul
Experiment	1D
Number of Scans	72
Receiver Gain	30
Relaxation Delay	1.0000
Pulse Width	0.0000
Acquisition Time	2.5559
Modification Date	2023-02-19T12:18:00
Spectrometer Frequency	399.76
Spectral Width	6410.3
Lowest Frequency	-799.0
Nucleus	1H

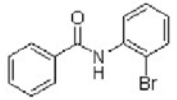

4au

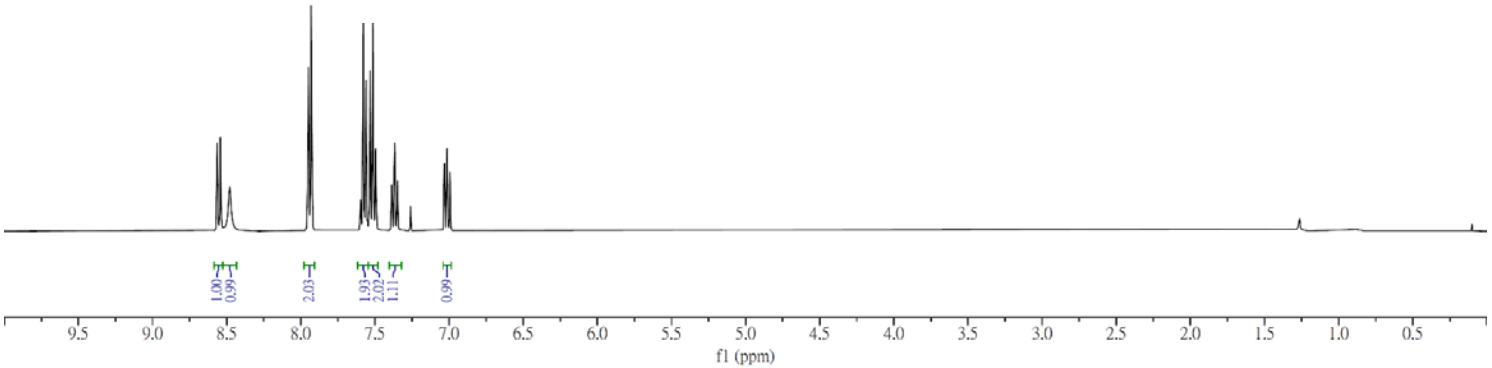

4au ^{1}H NMR spectrum (400 MHz in $CDCl_3$)

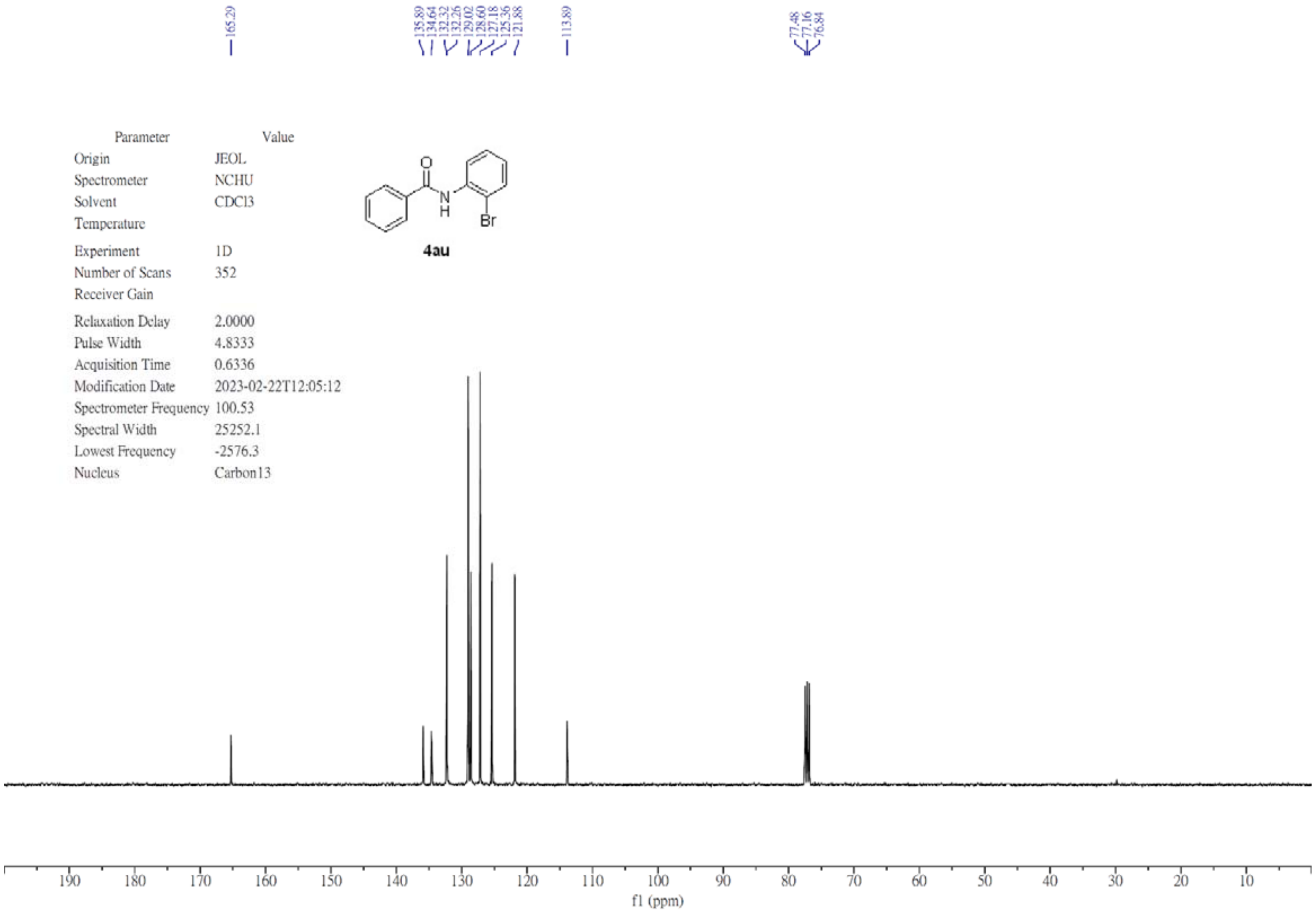

4au $^{13}C\{^{1}H\}$ NMR spectrum (100 MHz in $CDCl_3$)

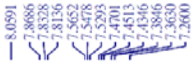

Parameter	Value
Origin	JEOL
Spectrometer	NCHU
Solvent	CDCl3
Temperature	
Experiment	1D
Number of Scans	8
Receiver Gain	
Relaxation Delay	2.0000
Pulse Width	5.2500
Acquisition Time	2.3962
Modification Date	2023-04-17T13:17:52
Spectrometer Frequency	399.78
Spectral Width	8012.7
Lowest Frequency	-2002.6
Nucleus	Proton

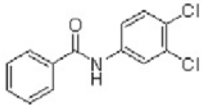

4aw

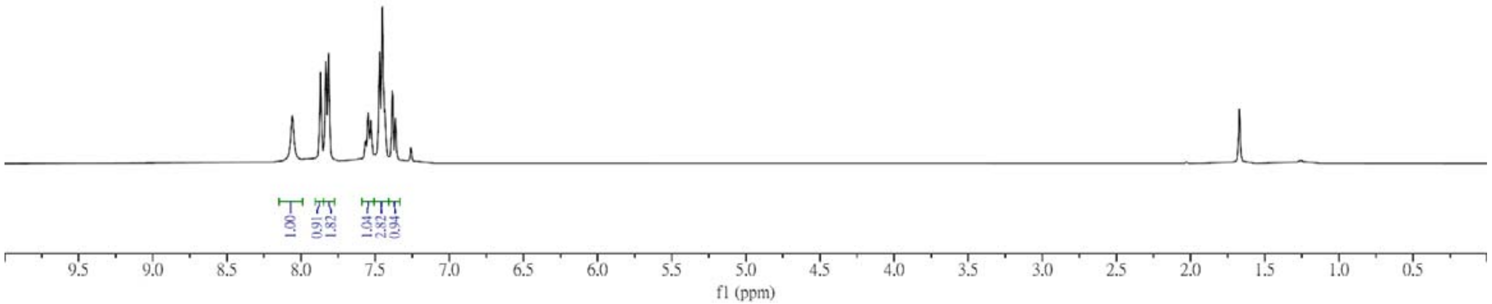

4aw ^{1}H NMR spectrum (400 MHz in $CDCl_3$)

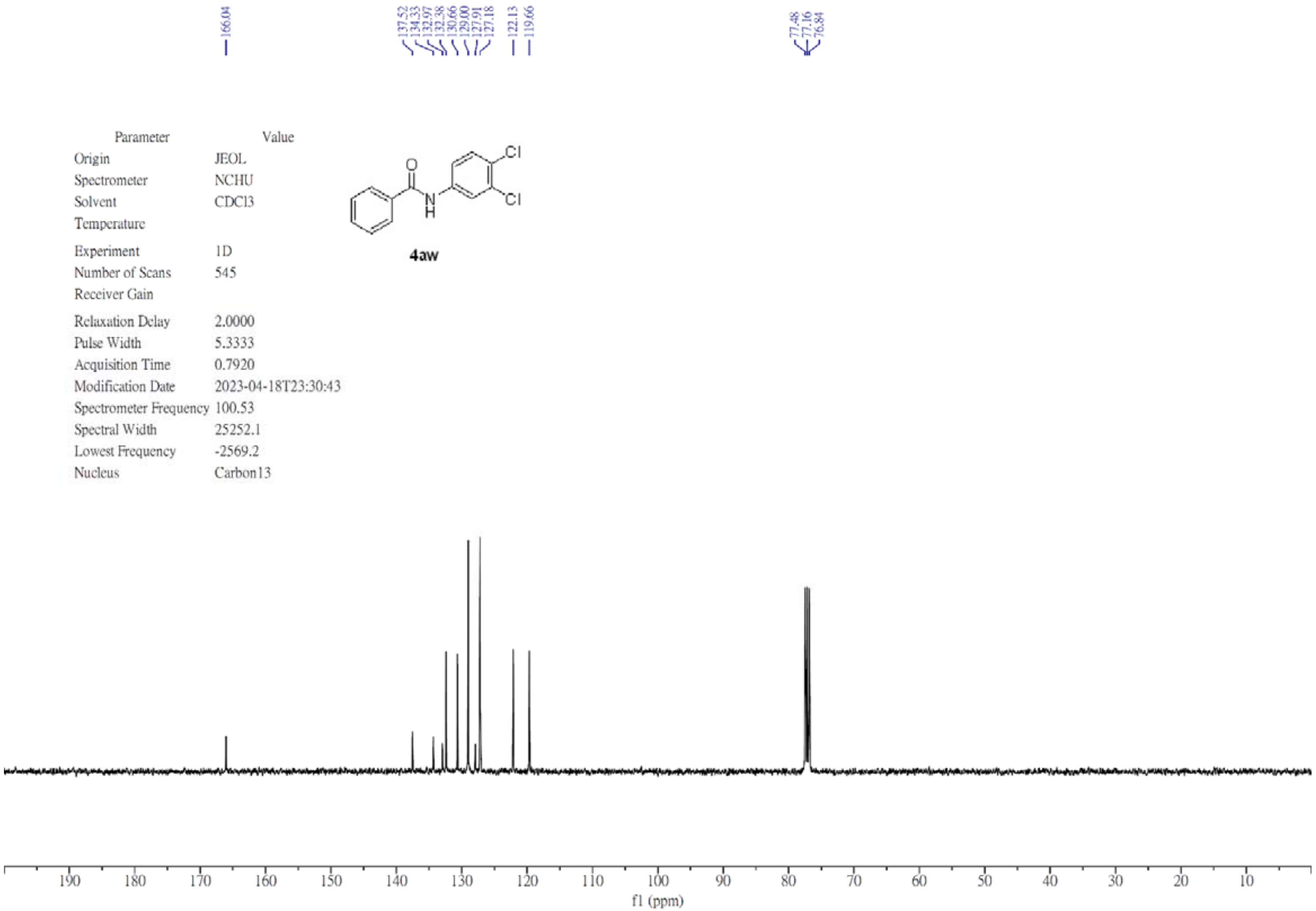

4aw $^{13}C\{^{1}H\}$ NMR spectrum (100 MHz in $CDCl_3$)

Parameter	Value
Origin	Varian
Spectrometer	vnmrs
Solvent	CDCl3
Temperature	25.0
Pulse Sequence	s2pul
Experiment	1D
Number of Scans	4
Receiver Gain	30
Relaxation Delay	1.0000
Pulse Width	0.0000
Acquisition Time	2.5559
Modification Date	2023-04-27T16:31:00
Spectrometer Frequency	399.76
Spectral Width	6410.3
Lowest Frequency	-798.8
Nucleus	1H

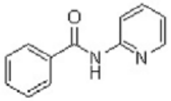

4ax

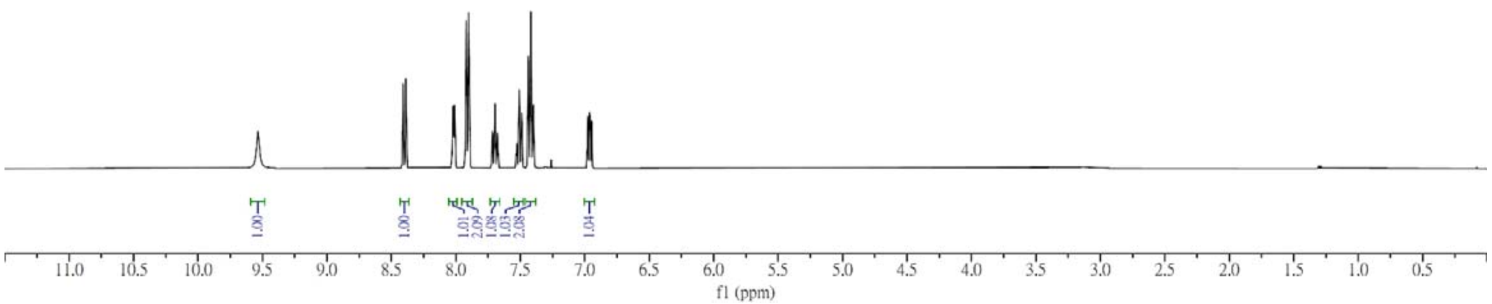

4ax ^{1}H NMR spectrum (400 MHz in $CDCl_3$)

Parameter	Value
Origin	JEOL
Spectrometer	NCHU
Solvent	CDCl3
Temperature	
Experiment	1D
Number of Scans	449
Receiver Gain	
Relaxation Delay	2.0000
Pulse Width	5.3333
Acquisition Time	0.7920
Modification Date	2023-04-18T23:22:06
Spectrometer Frequency	100.53
Spectral Width	25252.1
Lowest Frequency	-2590.5
Nucleus	Carbon13

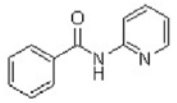

4ax

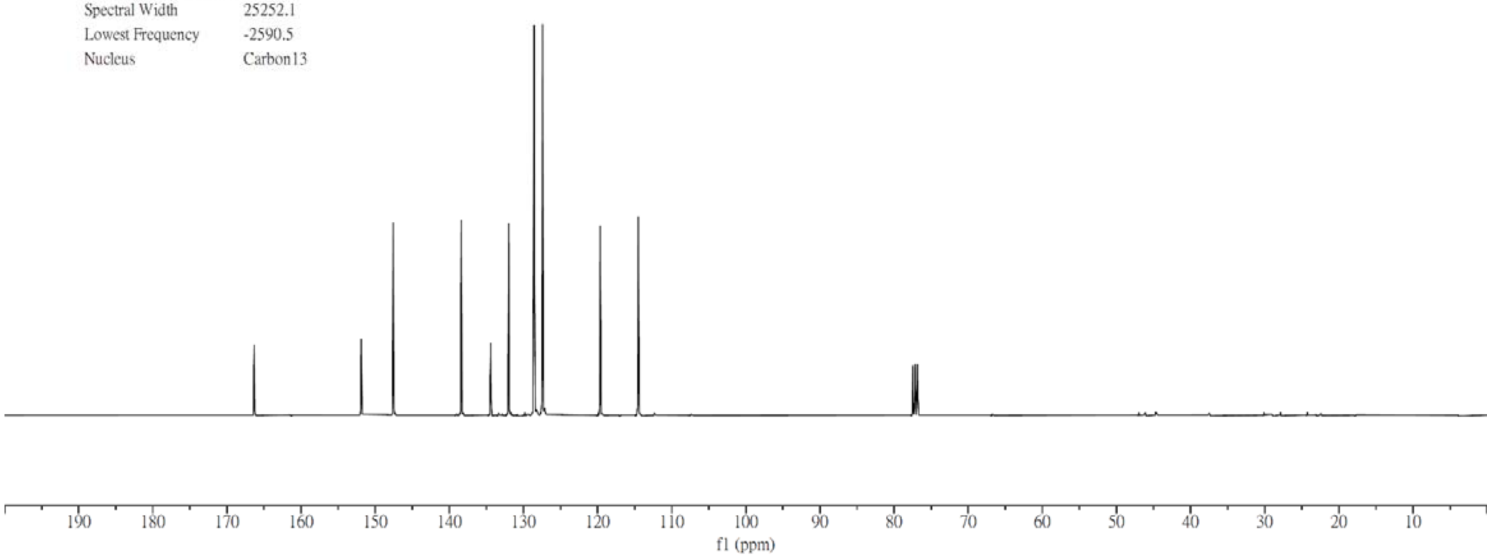

4ax $^{13}C\{^{1}H\}$ NMR spectrum (100 MHz in $CDCl_3$)

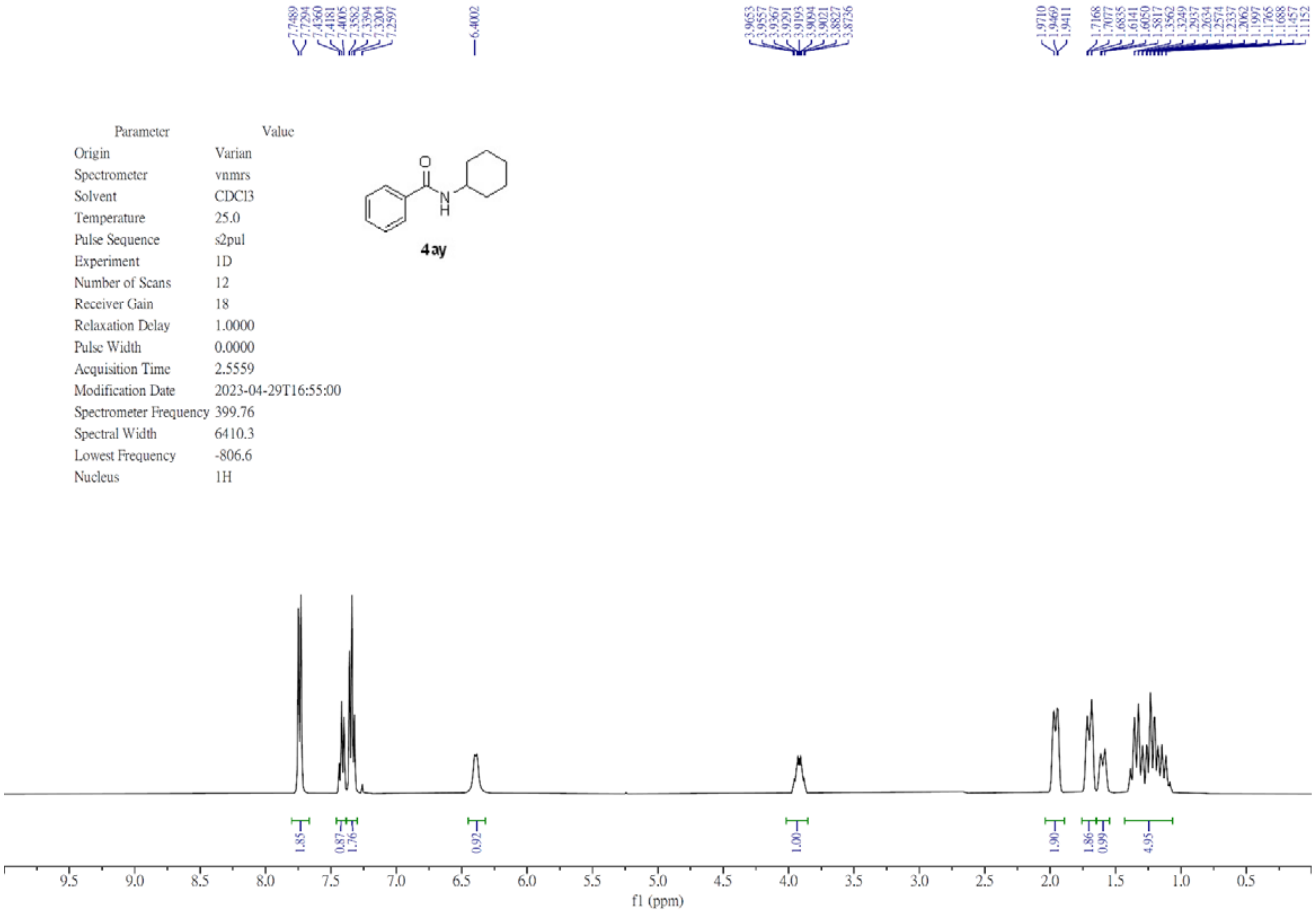

4ay ^{1}H NMR spectrum (400 MHz in $CDCl_3$)

Parameter	Value
Origin	JEOL
Spectrometer	NCHU
Solvent	CDCl3
Temperature	
Experiment	1D
Number of Scans	96
Receiver Gain	
Relaxation Delay	2.0000
Pulse Width	5.3333
Acquisition Time	0.7920
Modification Date	2023-04-18T23:42:49
Spectrometer Frequency	100.53
Spectral Width	25252.1
Lowest Frequency	-2575.7
Nucleus	Carbon13

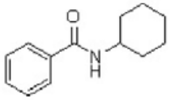

4ay

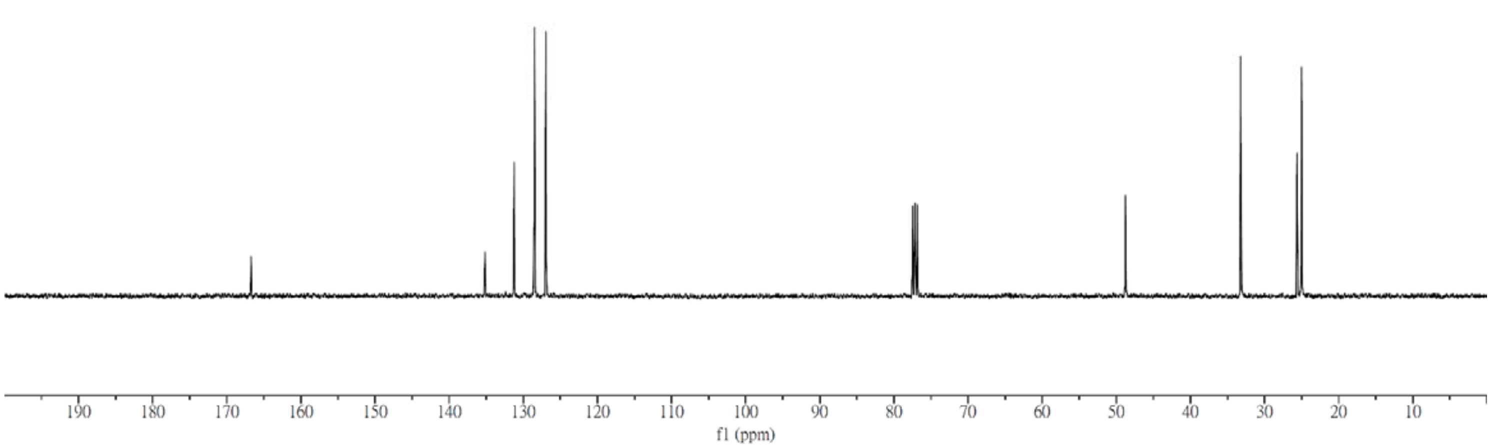

4ay $^{13}C\{^{1}H\}$ NMR spectrum (100 MHz in $CDCl_3$)

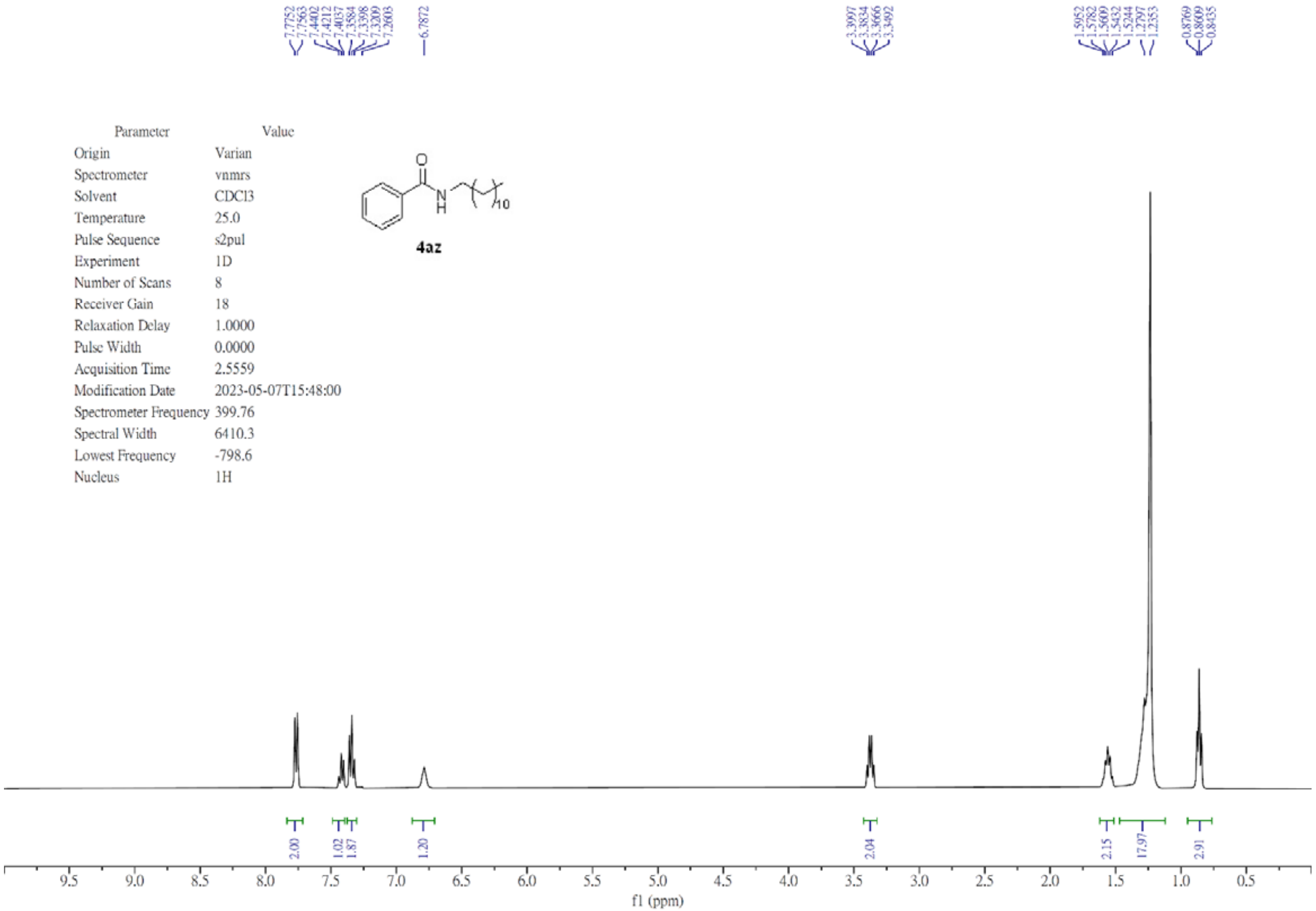

4az ^{1}H NMR spectrum (400 MHz in $CDCl_3$)

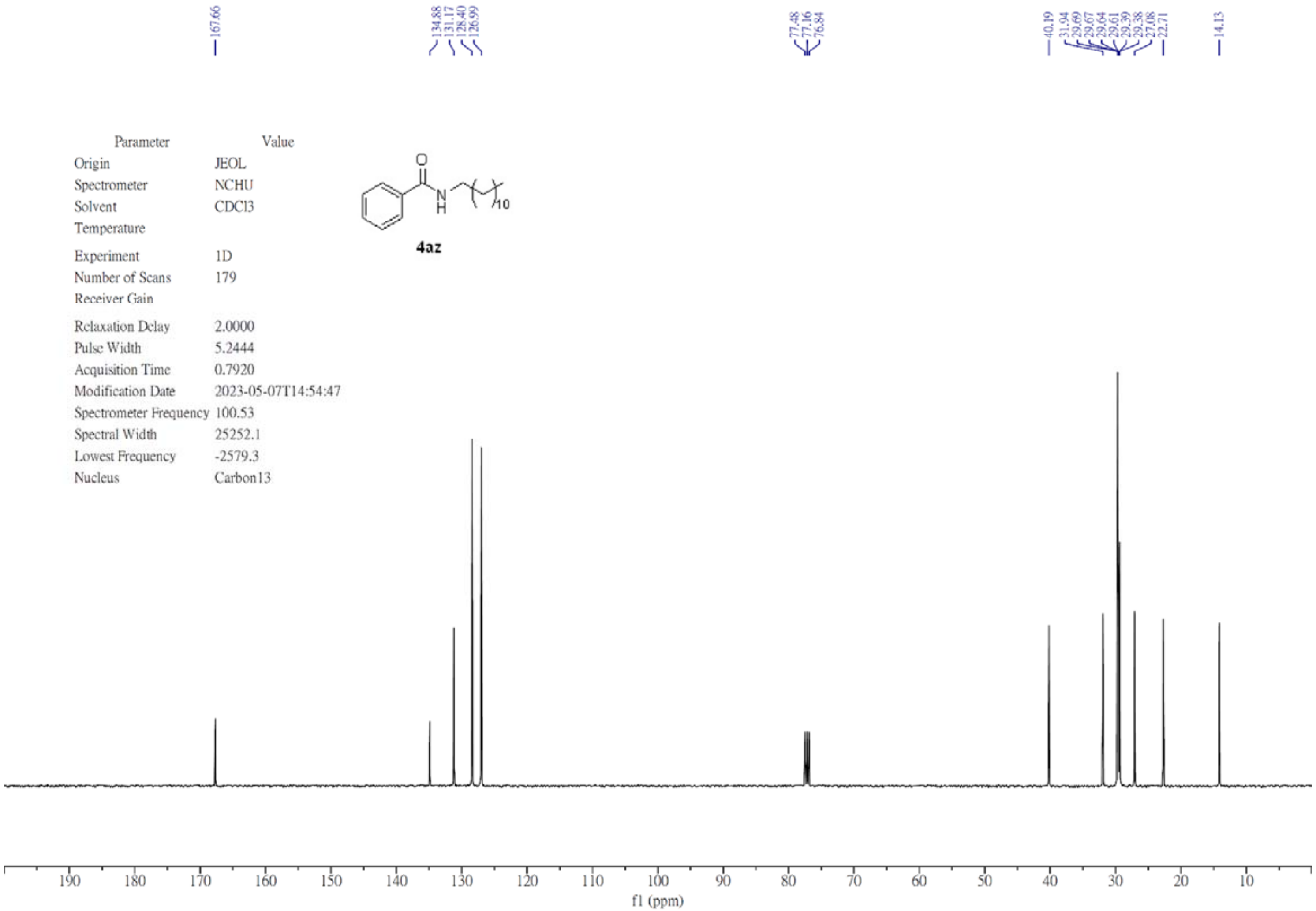

4az $^{13}C\{^{1}H\}$ NMR spectrum (100 MHz in $CDCl_3$)

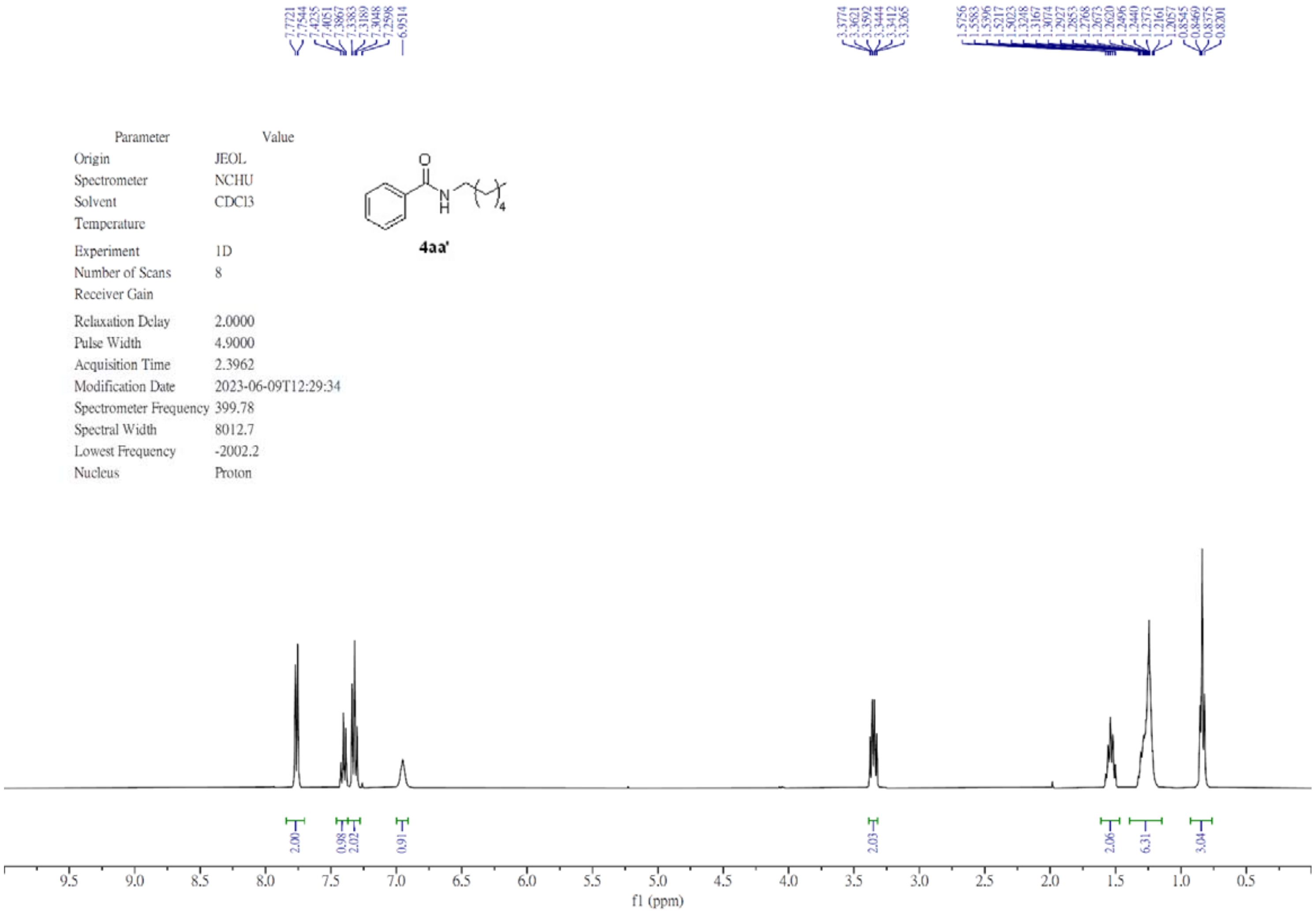

4aa' ^{1}H NMR spectrum (400 MHz in $CDCl_3$)

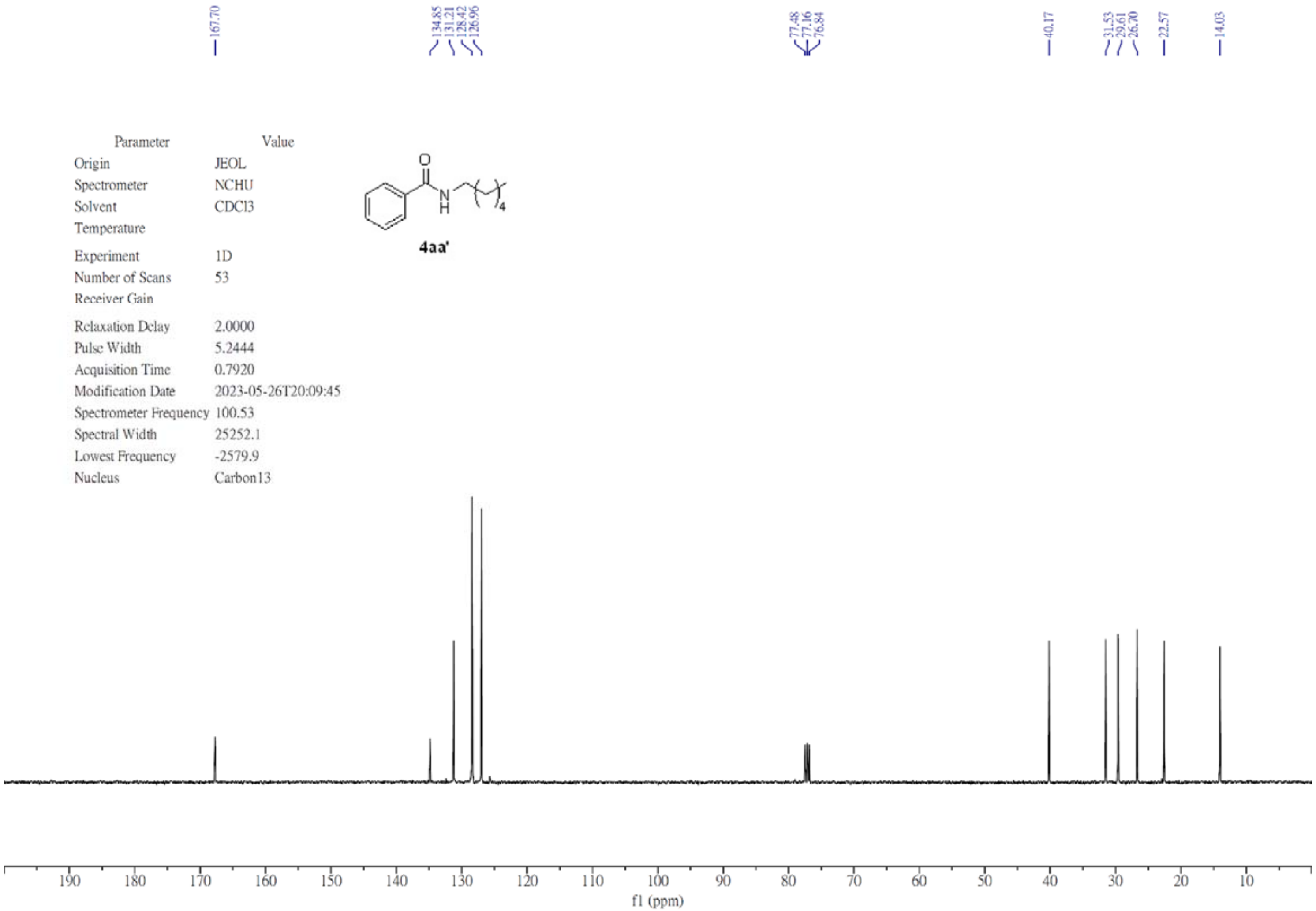

4aa' $^{13}C\{^{1}H\}$ NMR spectrum (100 MHz in $CDCl_3$)

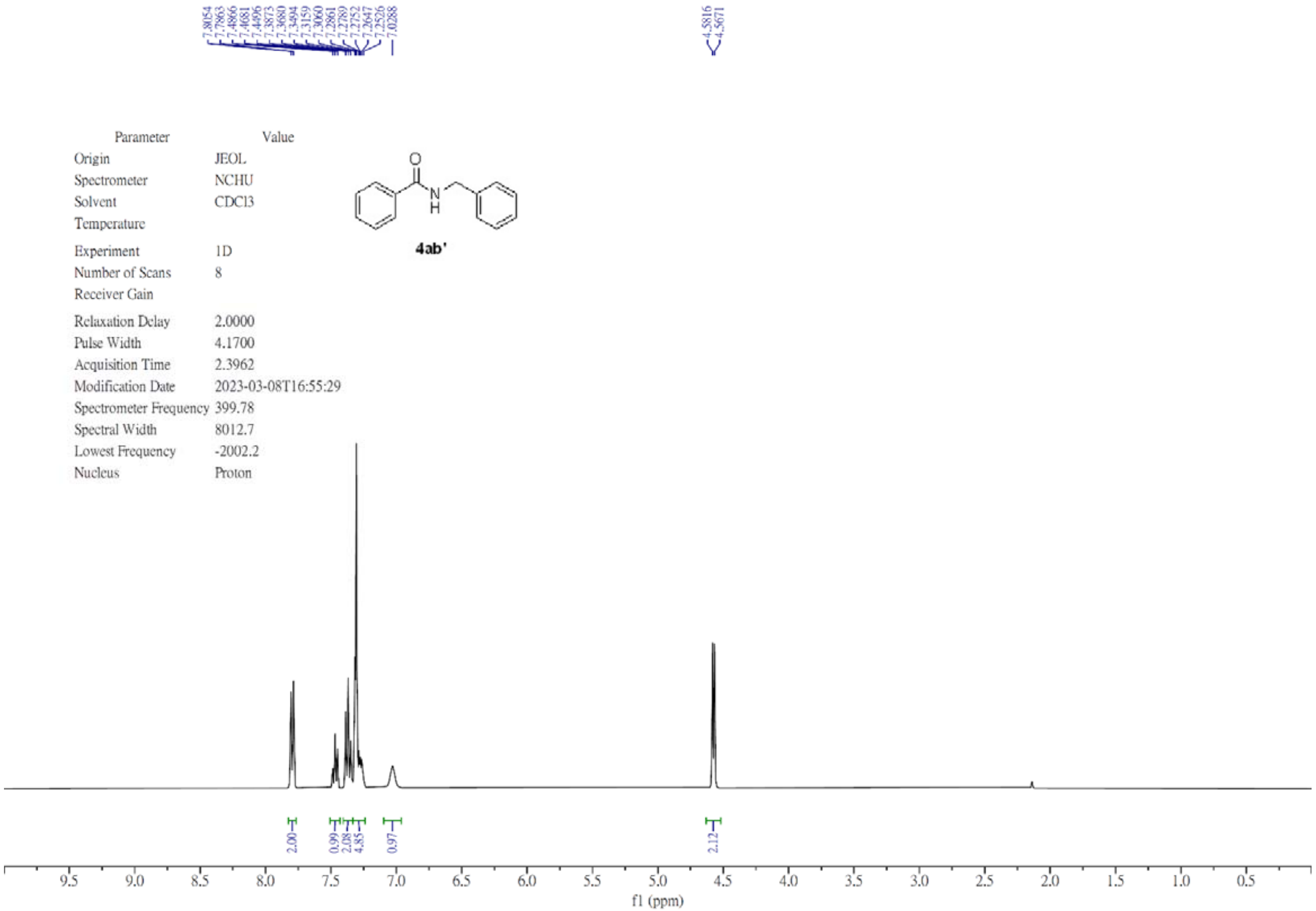

4ab' ^{1}H NMR spectrum (400 MHz in $CDCl_3$)

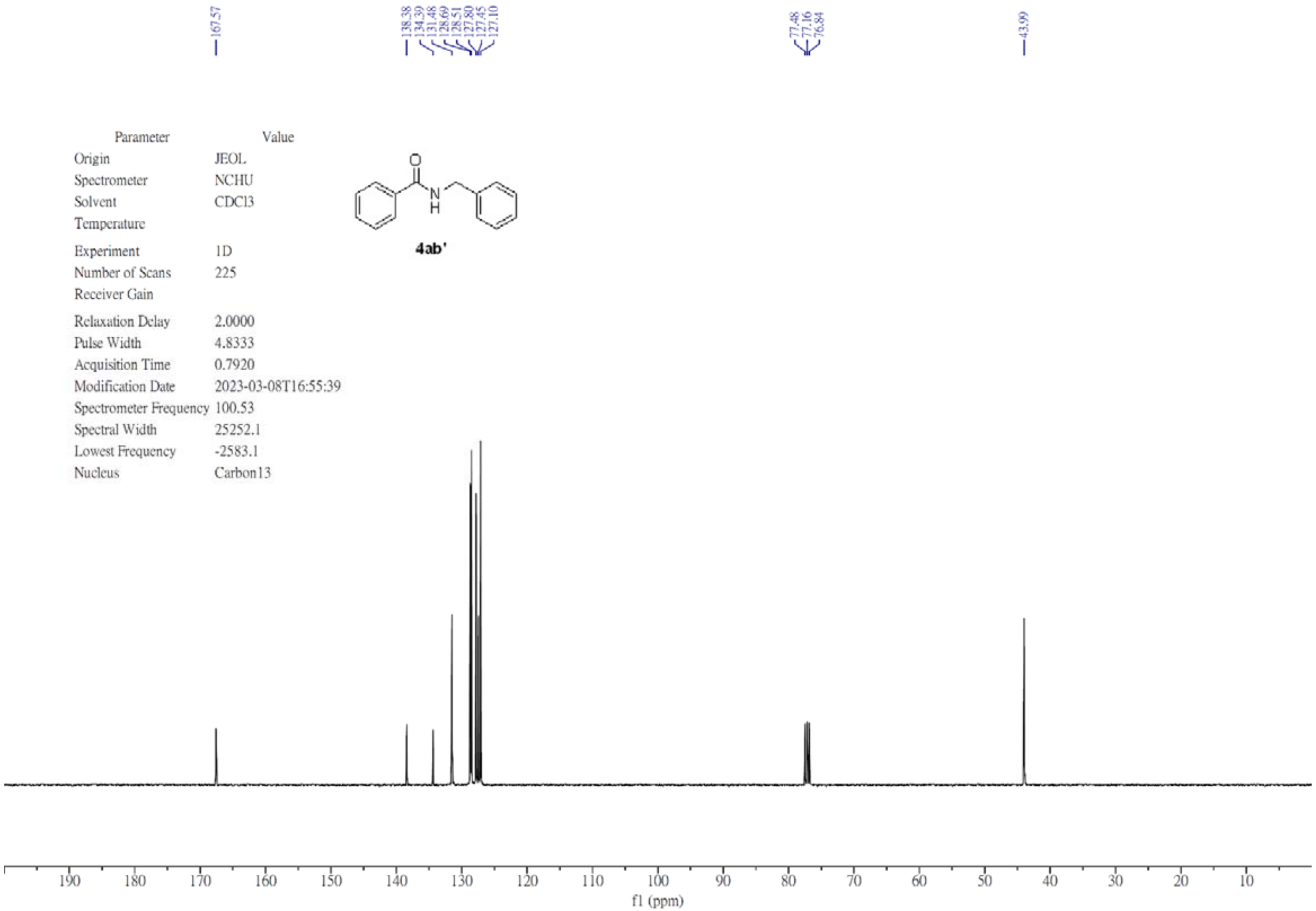

4ab' $^{13}C\{^{1}H\}$ NMR spectrum (100 MHz in $CDCl_3$)

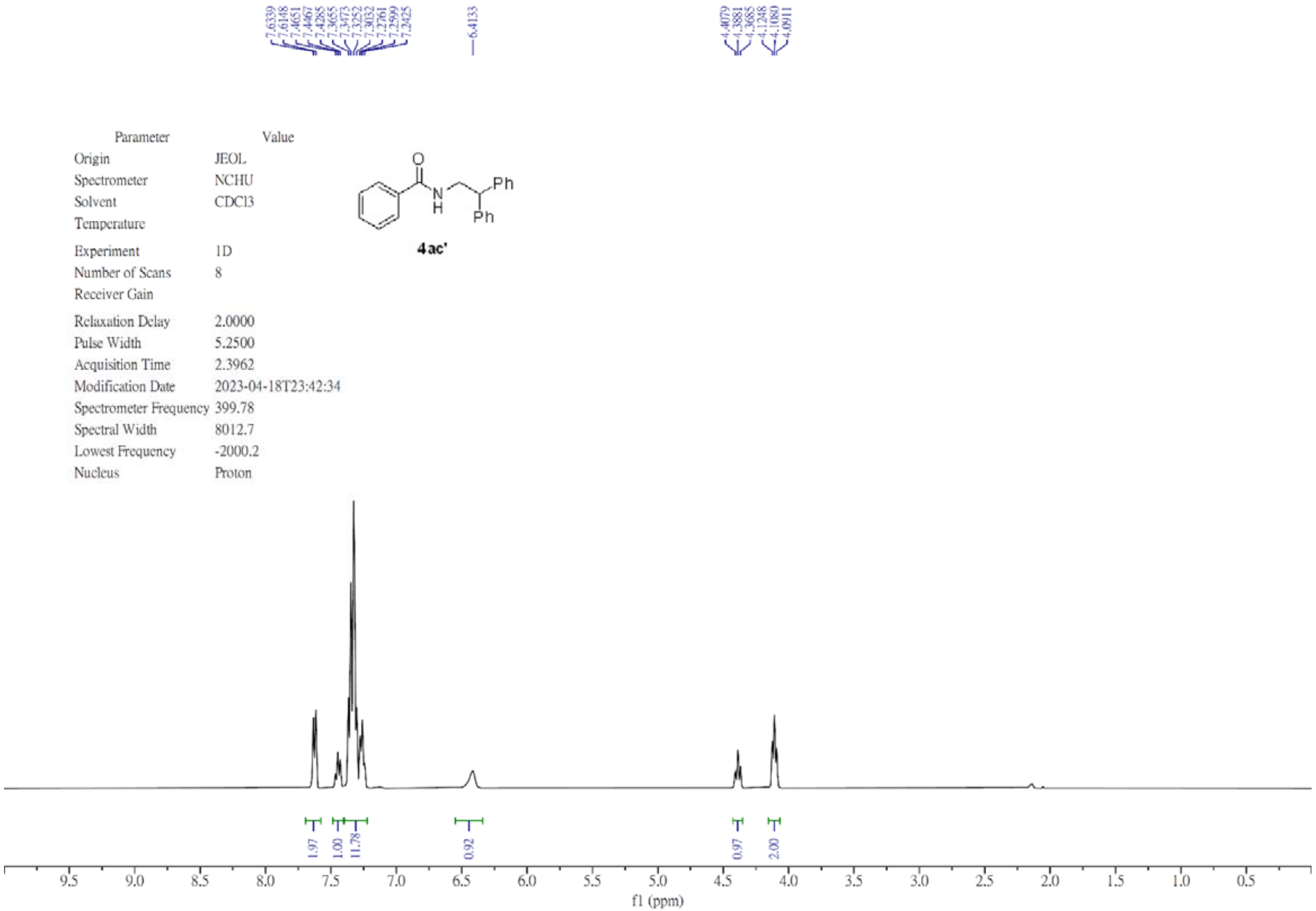

Parameter	Value
Origin	JEOL
Spectrometer	NCHU
Solvent	CDCl3
Temperature	
Experiment	1D
Number of Scans	8
Receiver Gain	
Relaxation Delay	2.0000
Pulse Width	5.2500
Acquisition Time	2.3962
Modification Date	2023-04-18T23:42:34
Spectrometer Frequency	399.78
Spectral Width	8012.7
Lowest Frequency	-2000.2
Nucleus	Proton

4ac' ^{1}H NMR spectrum (400 MHz in $CDCl_3$)

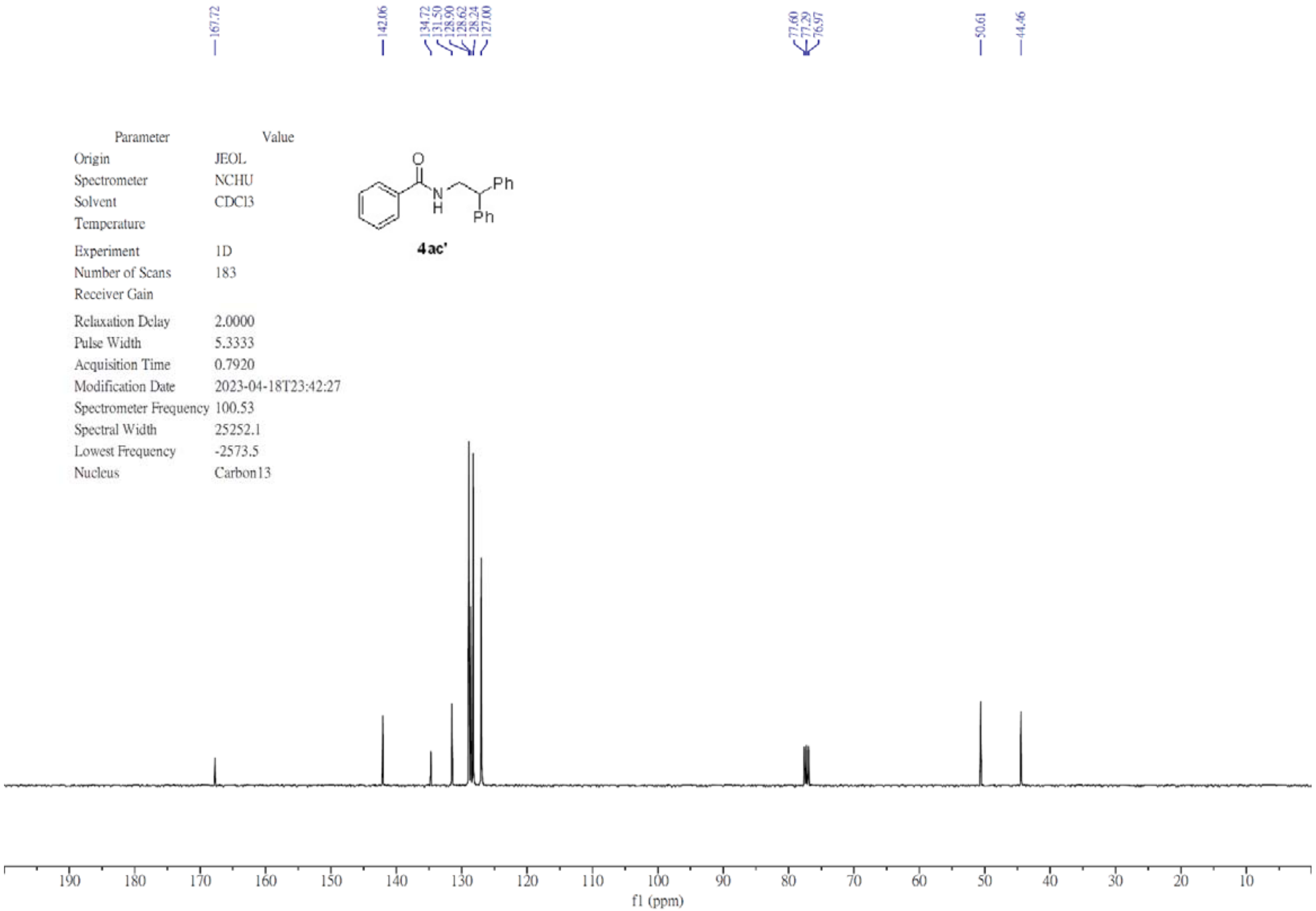

4ac' $^{13}C\{^{1}H\}$ NMR spectrum (100 MHz in $CDCl_3$)

Parameter	Value
Origin	JEOL
Spectrometer	NCHU
Solvent	CDCl3
Temperature	
Experiment	1D
Number of Scans	8
Receiver Gain	
Relaxation Delay	2.0000
Pulse Width	4.9000
Acquisition Time	2.3962
Modification Date	2023-05-19T18:09:32
Spectrometer Frequency	399.78
Spectral Width	8012.7
Lowest Frequency	-2002.2
Nucleus	Proton

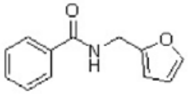

4ad'

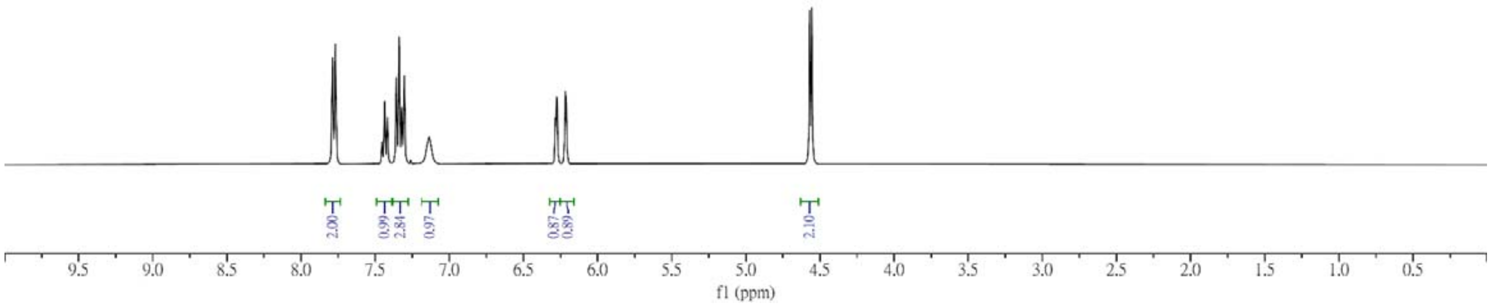

4ad' ^{1}H NMR spectrum (400 MHz in $CDCl_3$)

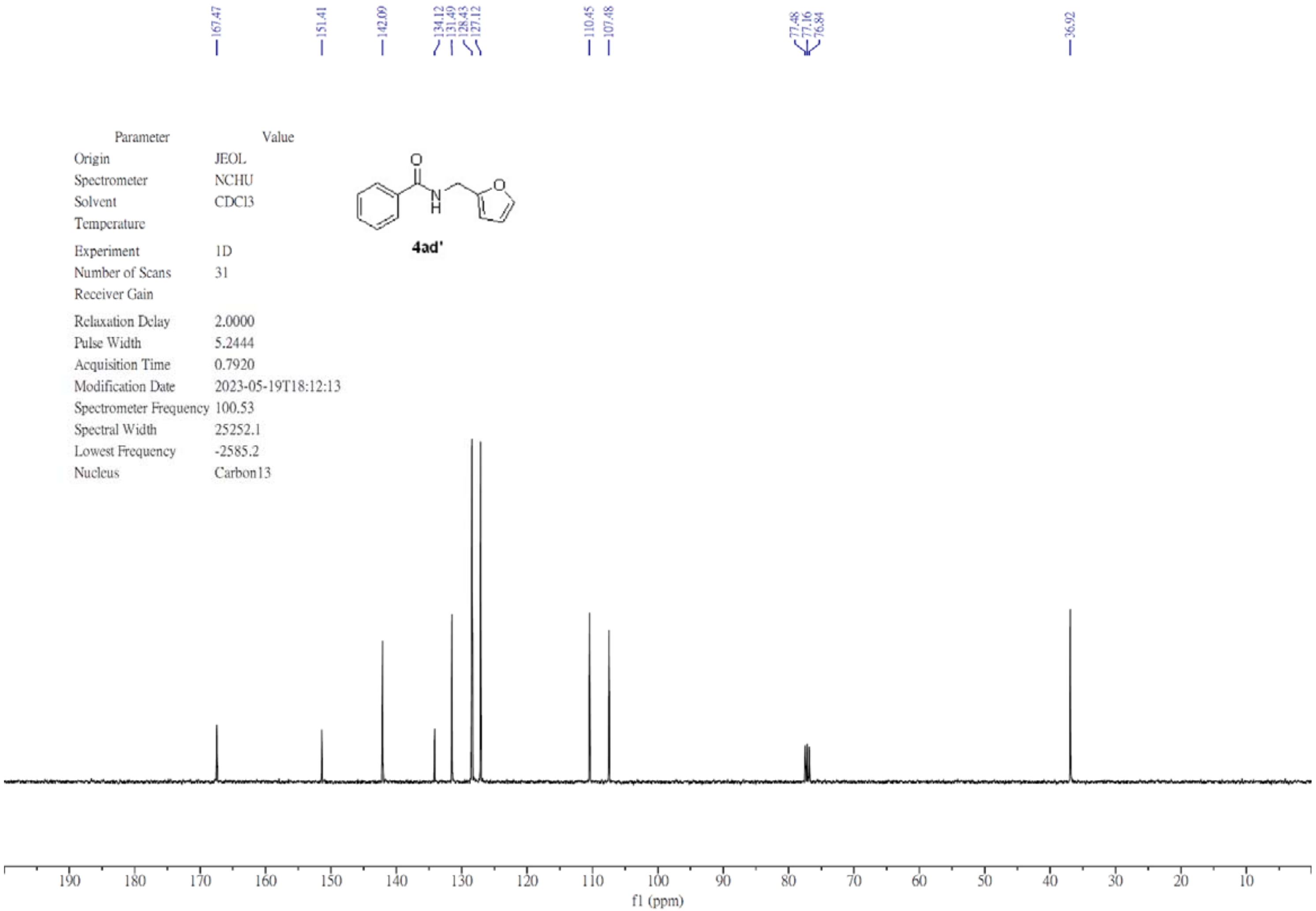

4ad' $^{13}C\{^{1}H\}$ NMR spectrum (100 MHz in $CDCl_3$)

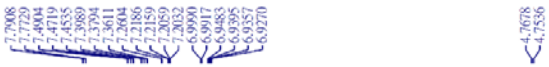

Parameter	Value
Origin	JEOL
Spectrometer	NCHU
Solvent	CDCl3
Temperature	
Experiment	1D
Number of Scans	16
Receiver Gain	
Relaxation Delay	2.0000
Pulse Width	4.9000
Acquisition Time	2.3962
Modification Date	2023-06-09T11:40:55
Spectrometer Frequency	399.78
Spectral Width	8012.7
Lowest Frequency	-2002.2
Nucleus	Proton

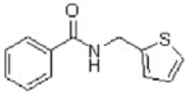

4ae'

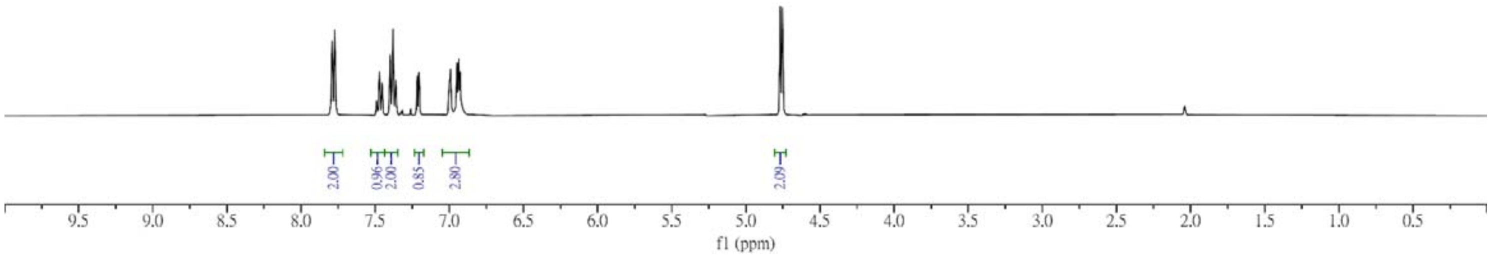

4ae' ^{1}H NMR spectrum (400 MHz in $CDCl_3$)

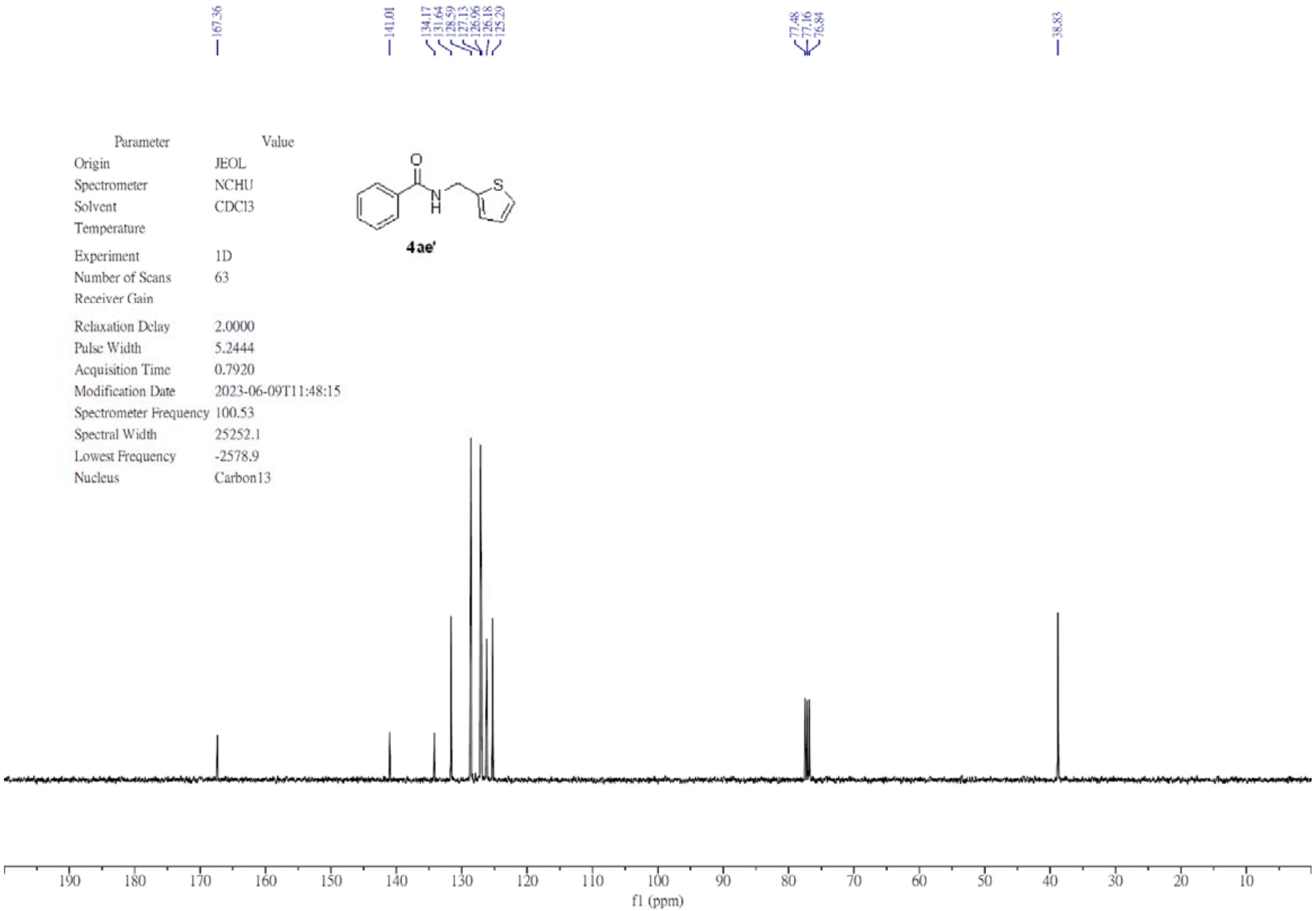

4ae' $^{13}C\{^{1}H\}$ NMR spectrum (100 MHz in $CDCl_3$)

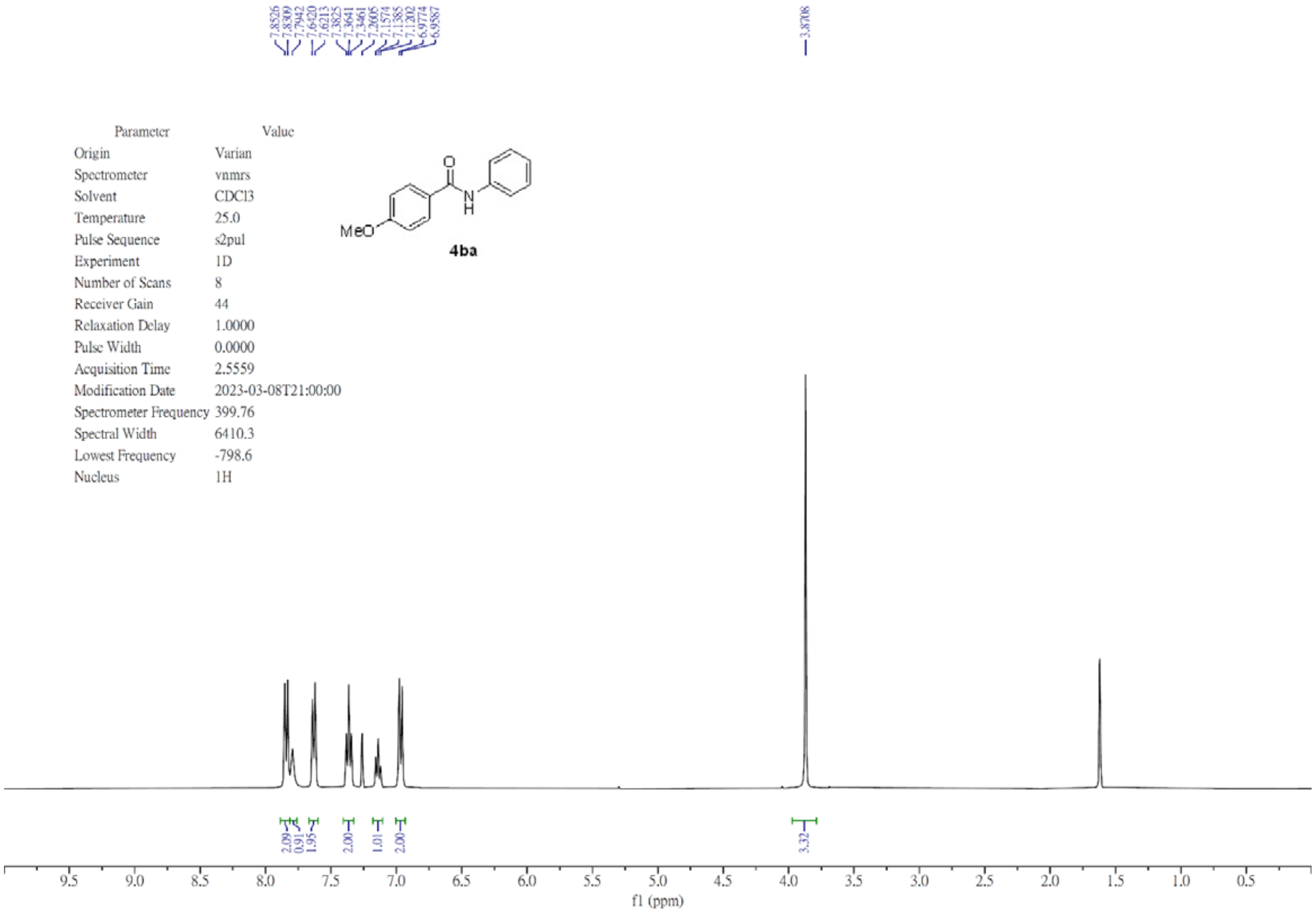

4ba ^{1}H NMR spectrum (400 MHz in $CDCl_3$)

Parameter	Value
Origin	JEOL
Spectrometer	NCHU
Solvent	CDCl3
Temperature	
Experiment	1D
Number of Scans	719
Receiver Gain	
Relaxation Delay	2.0000
Pulse Width	4.8333
Acquisition Time	0.7920
Modification Date	2023-03-08T16:55:45
Spectrometer Frequency	100.53
Spectral Width	25252.1
Lowest Frequency	-2567.8
Nucleus	Carbon13

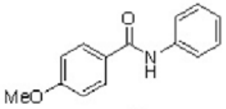

4ba

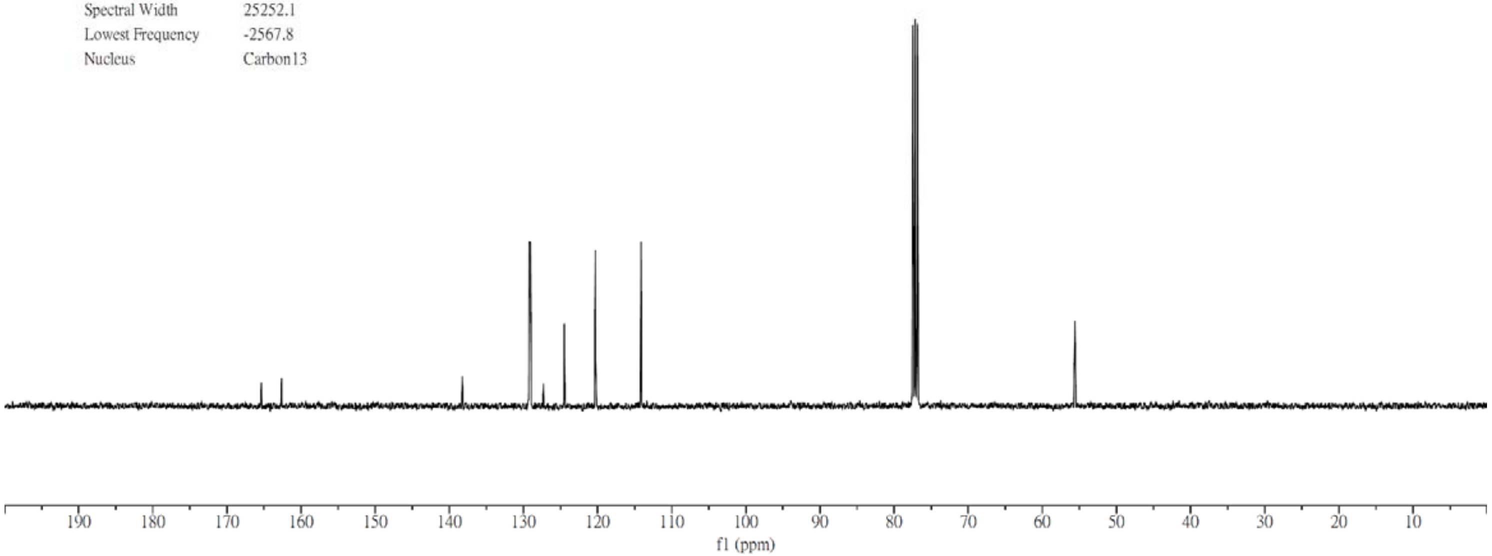

4ba $^{13}C\{^1H\}$ NMR spectrum (100 MHz in $CDCl_3$)

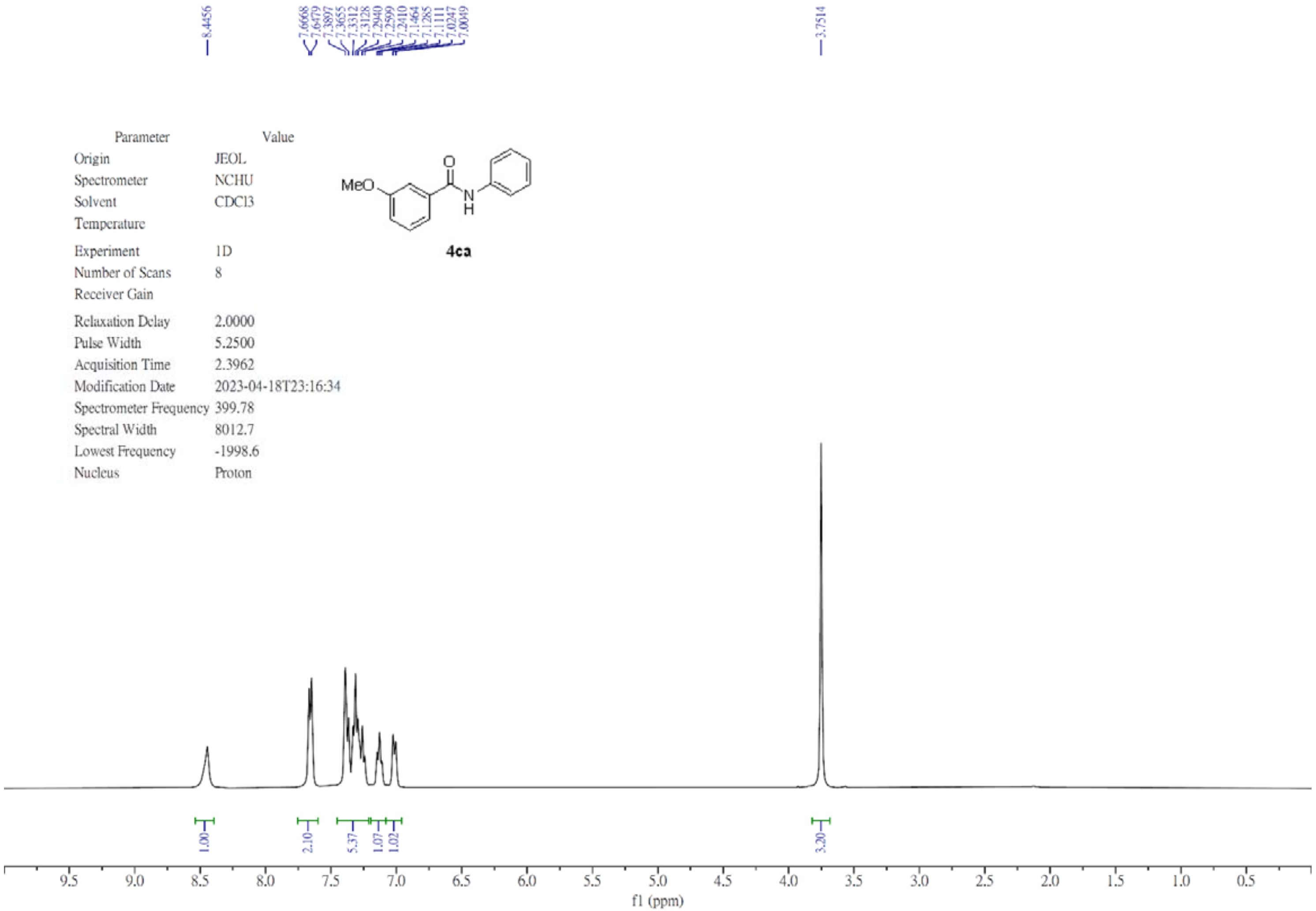

4ca ^{1}H NMR spectrum (400 MHz in $CDCl_3$)

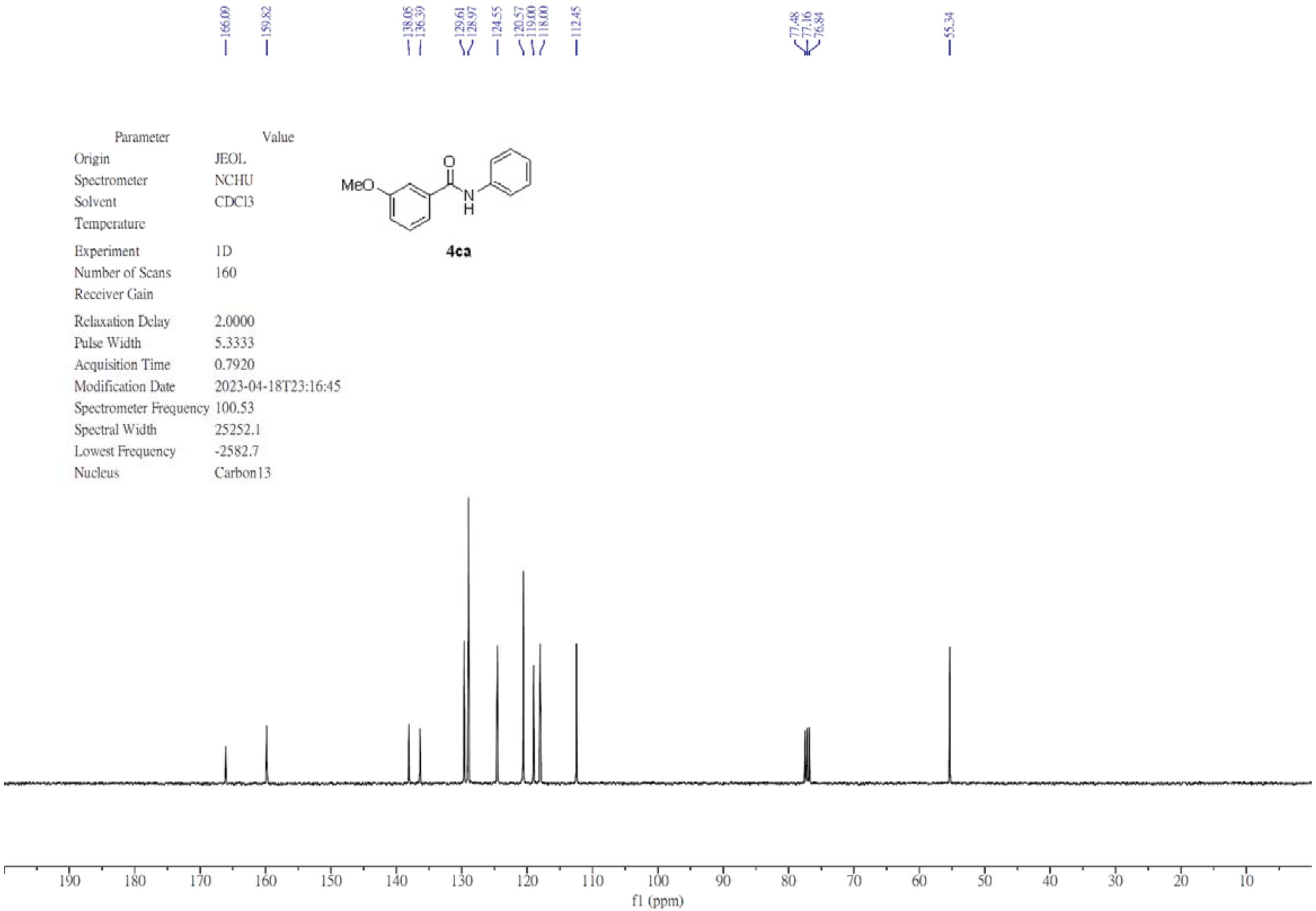

4ca $^{13}C\{^{1}H\}$ NMR spectrum (100 MHz in $CDCl_3$)

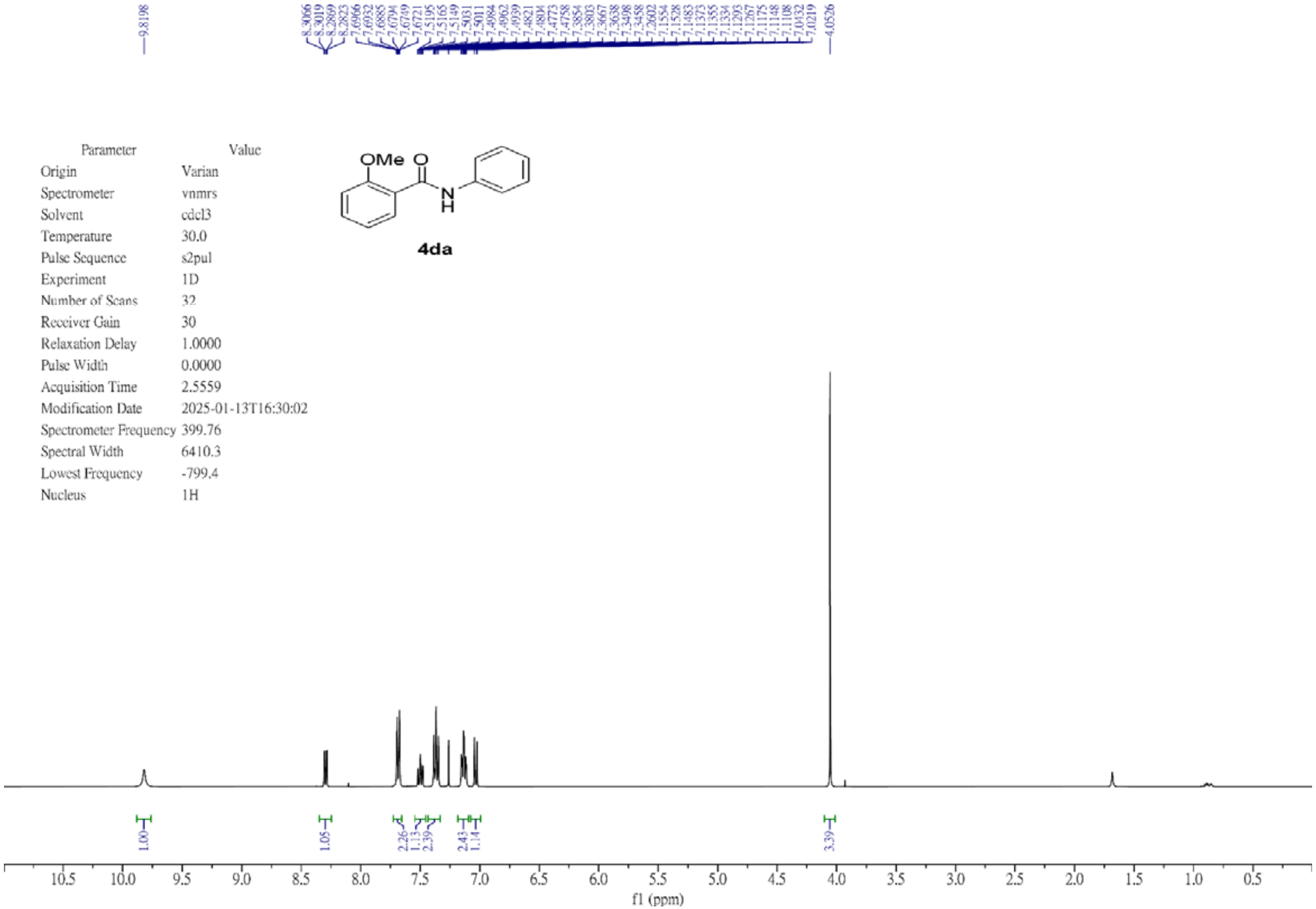

4da ^{1}H NMR spectrum (400 MHz in $CDCl_3$)

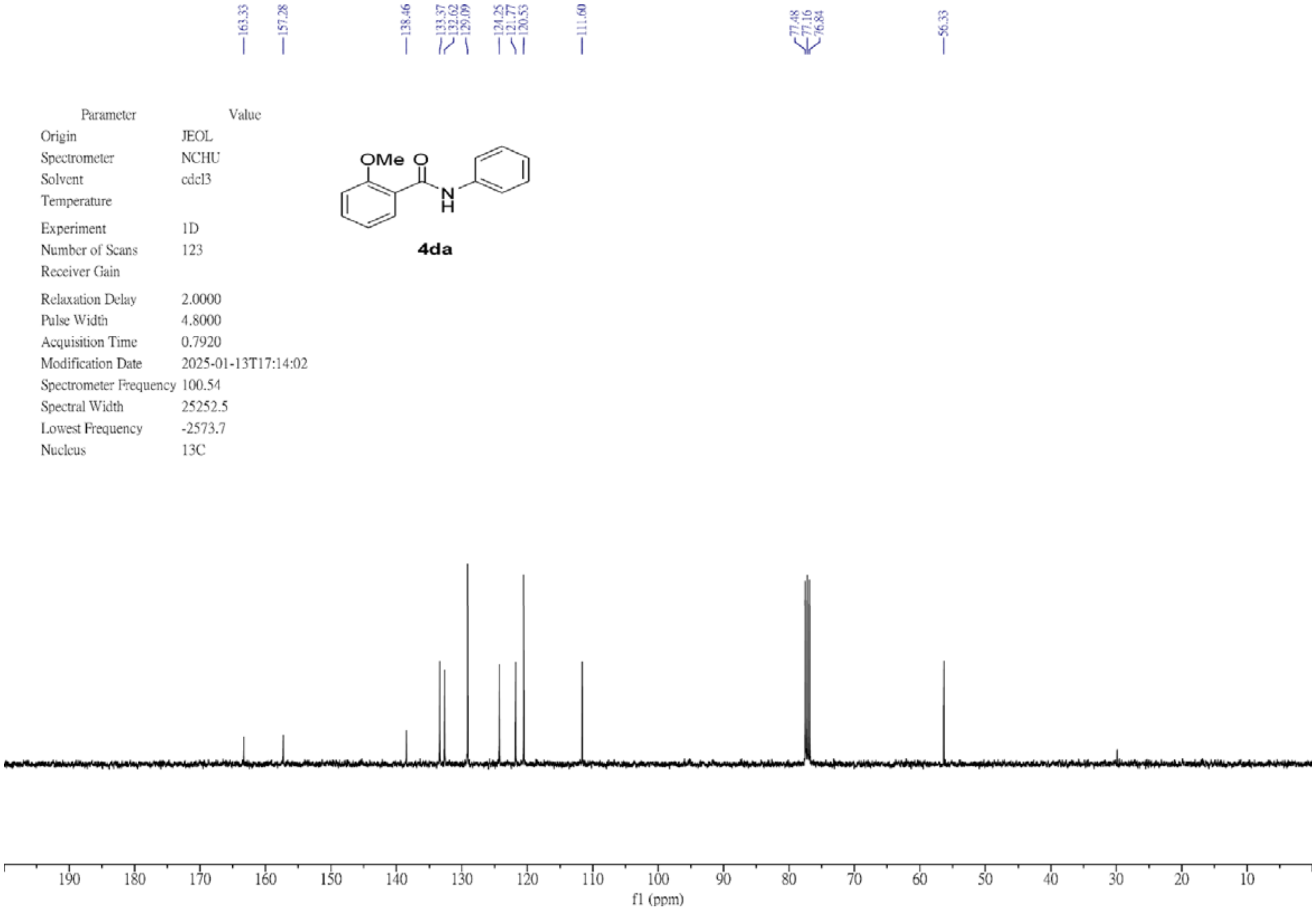

4da $^{13}C\{^{1}H\}$ NMR spectrum (100 MHz in $CDCl_3$)

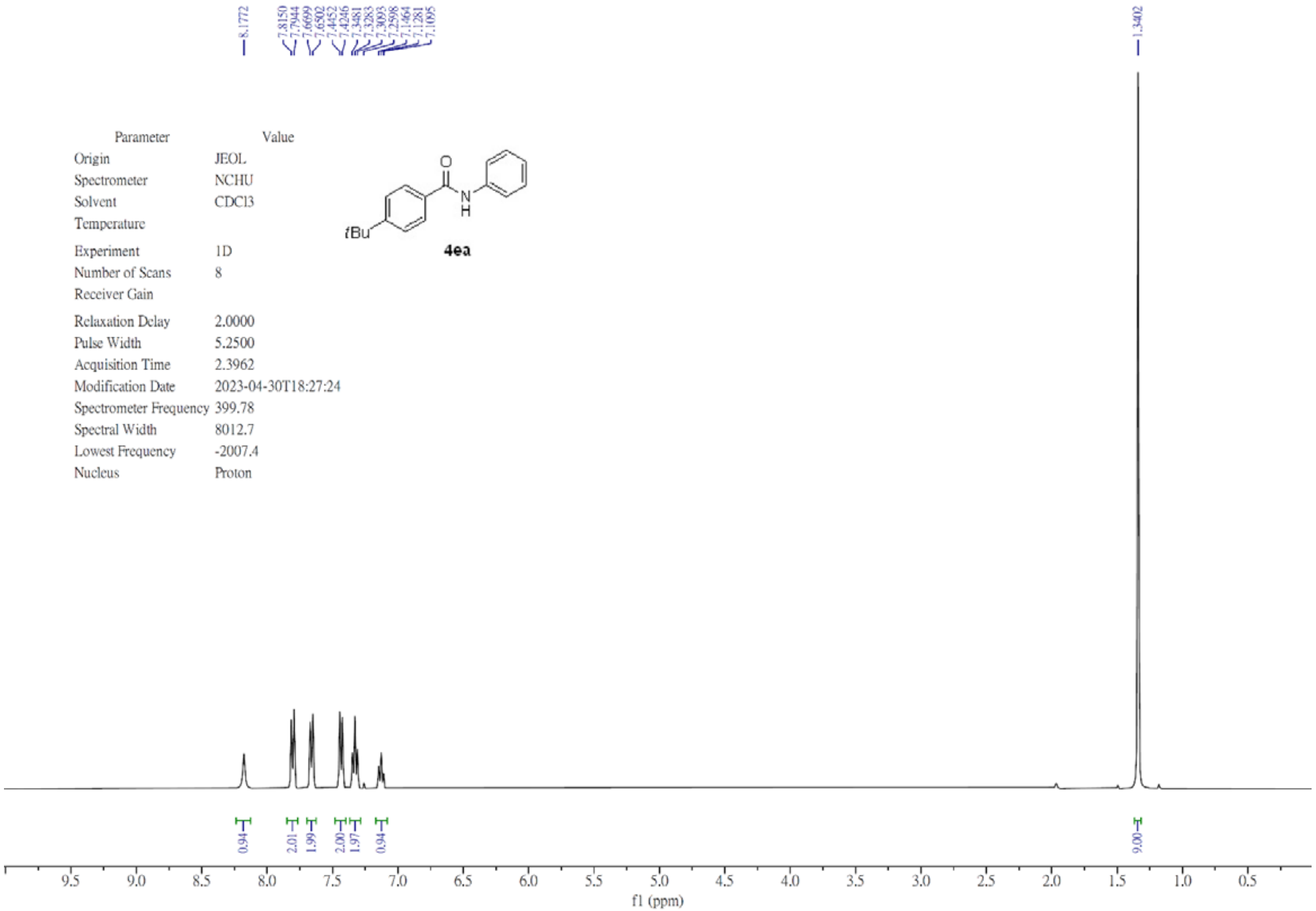

Parameter	Value
Origin	JEOL
Spectrometer	NCHU
Solvent	CDCl3
Temperature	
Experiment	1D
Number of Scans	8
Receiver Gain	
Relaxation Delay	2.0000
Pulse Width	5.2500
Acquisition Time	2.3962
Modification Date	2023-04-30T18:27:24
Spectrometer Frequency	399.78
Spectral Width	8012.7
Lowest Frequency	-2007.4
Nucleus	Proton

4ea

4ea ^{1}H NMR spectrum (400 MHz in $CDCl_3$)

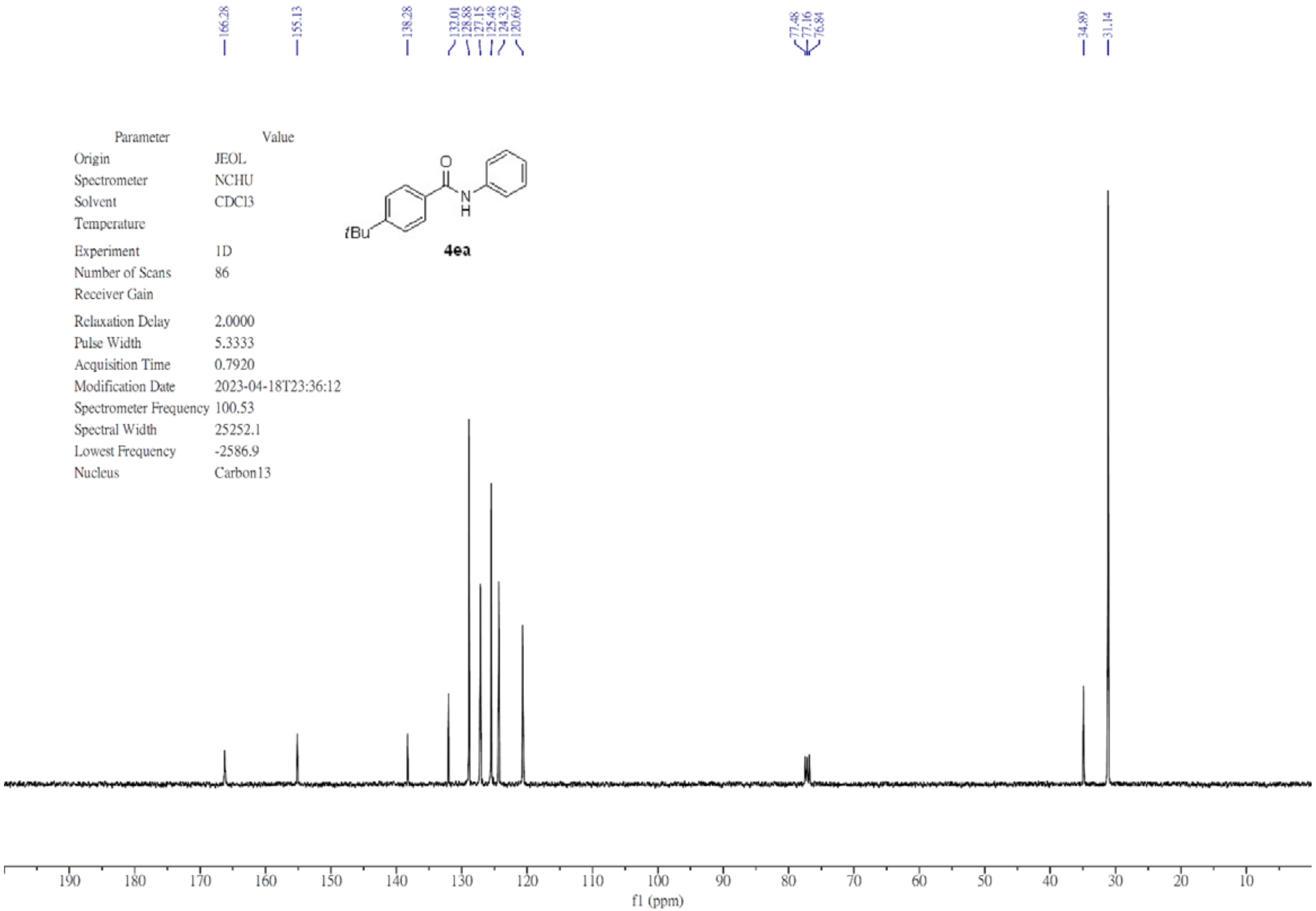

4ea $^{13}C\{^{1}H\}$ NMR spectrum (100 MHz in $CDCl_3$)

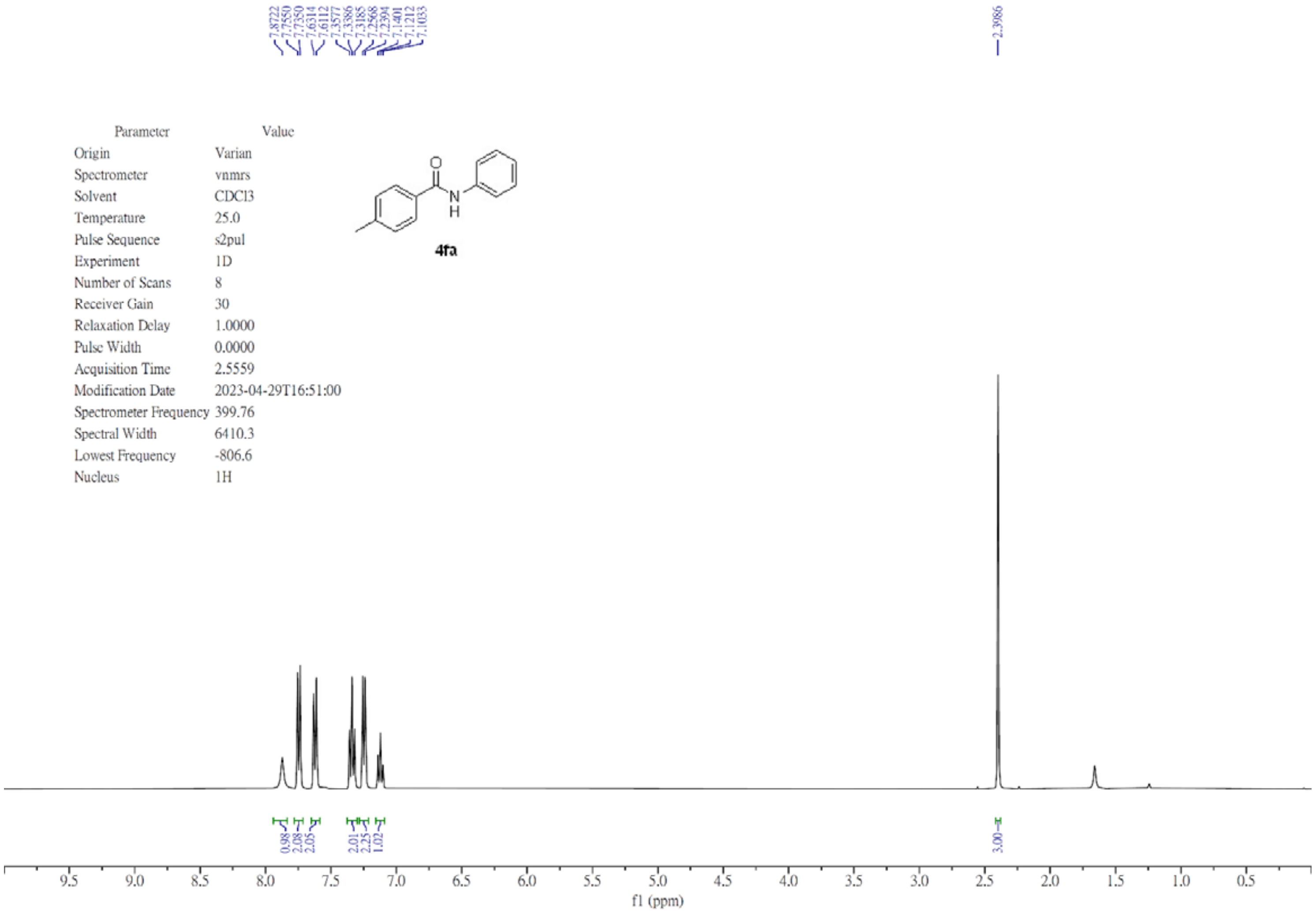

4fa ^{1}H NMR spectrum (400 MHz in $(CD_3)_2SO$)

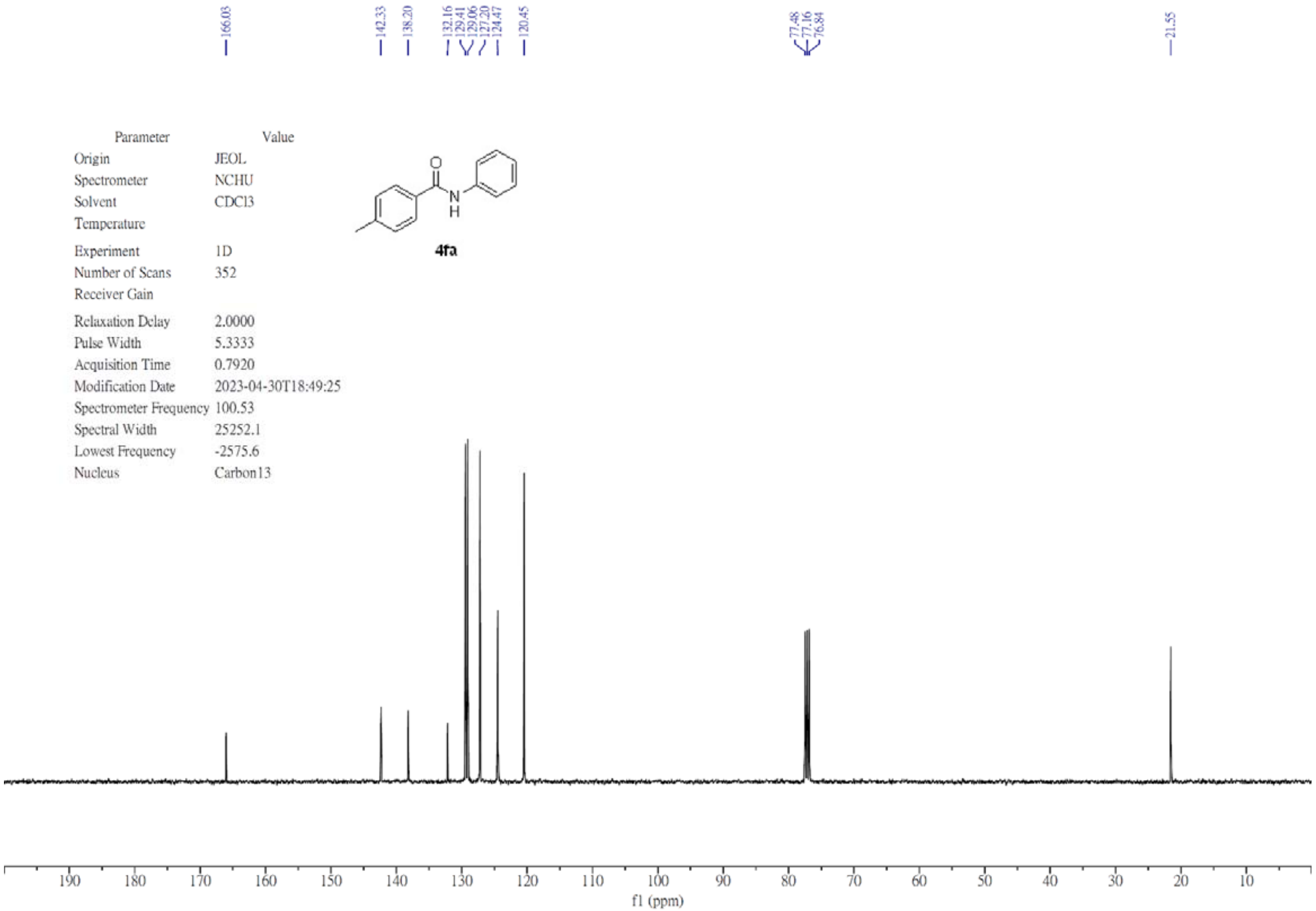

4fa $^{13}C\{^{1}H\}$ NMR spectrum (100 MHz in $(CD_3)_2SO$)

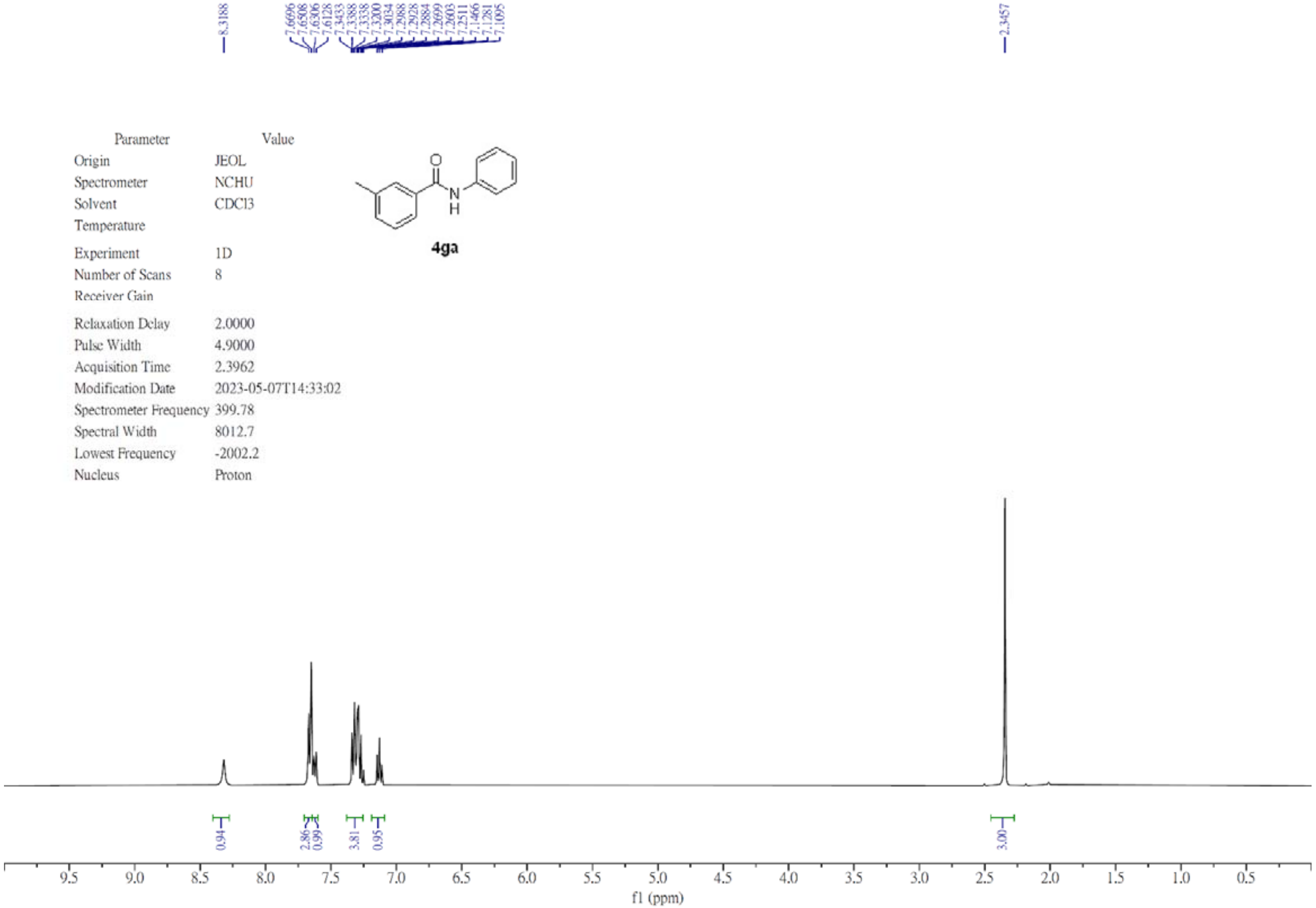

4ga ^{1}H NMR spectrum (400 MHz in $CDCl_3$)

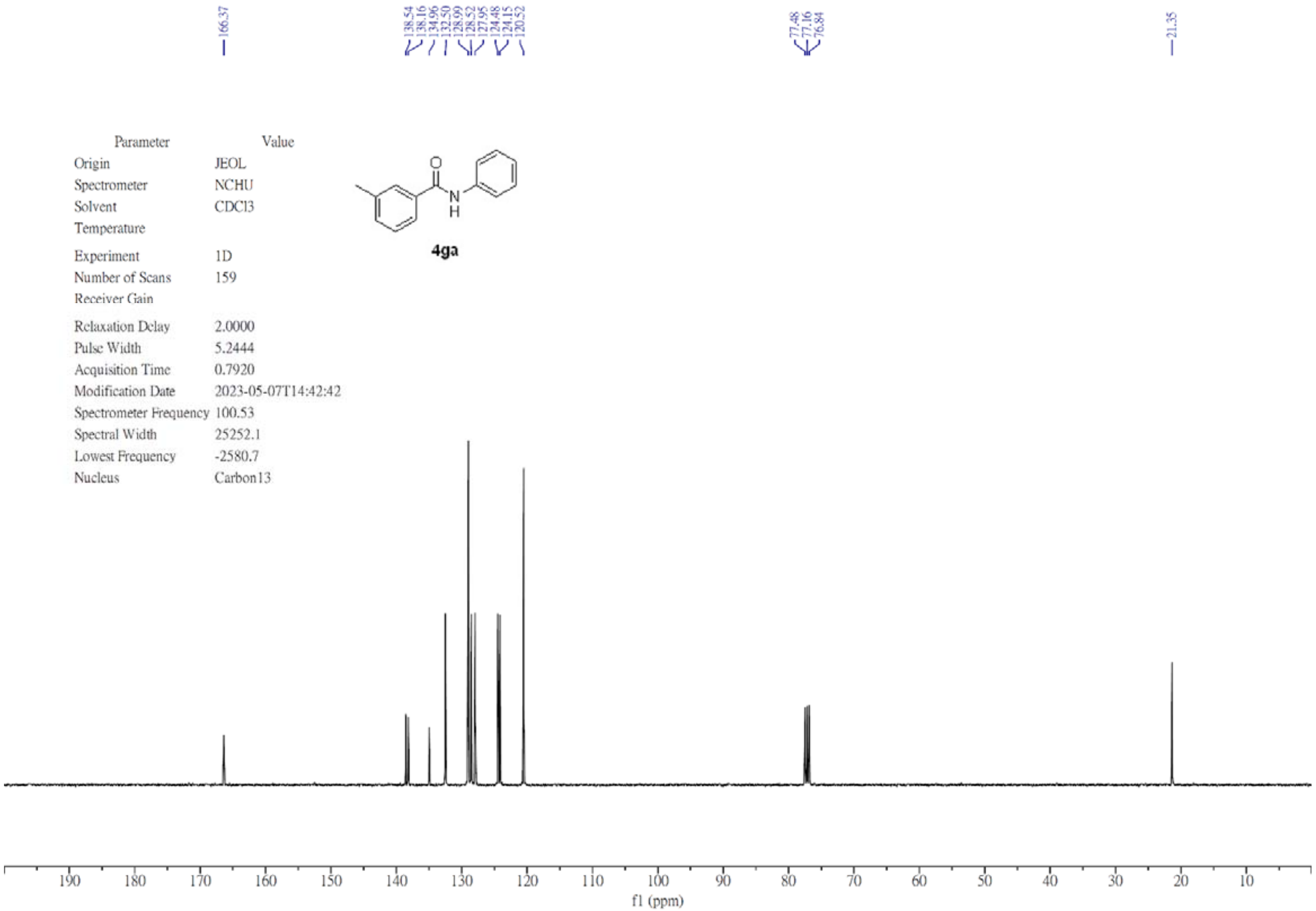

4ga $^{13}C\{^{1}H\}$ NMR spectrum (100 MHz in $CDCl_3$)

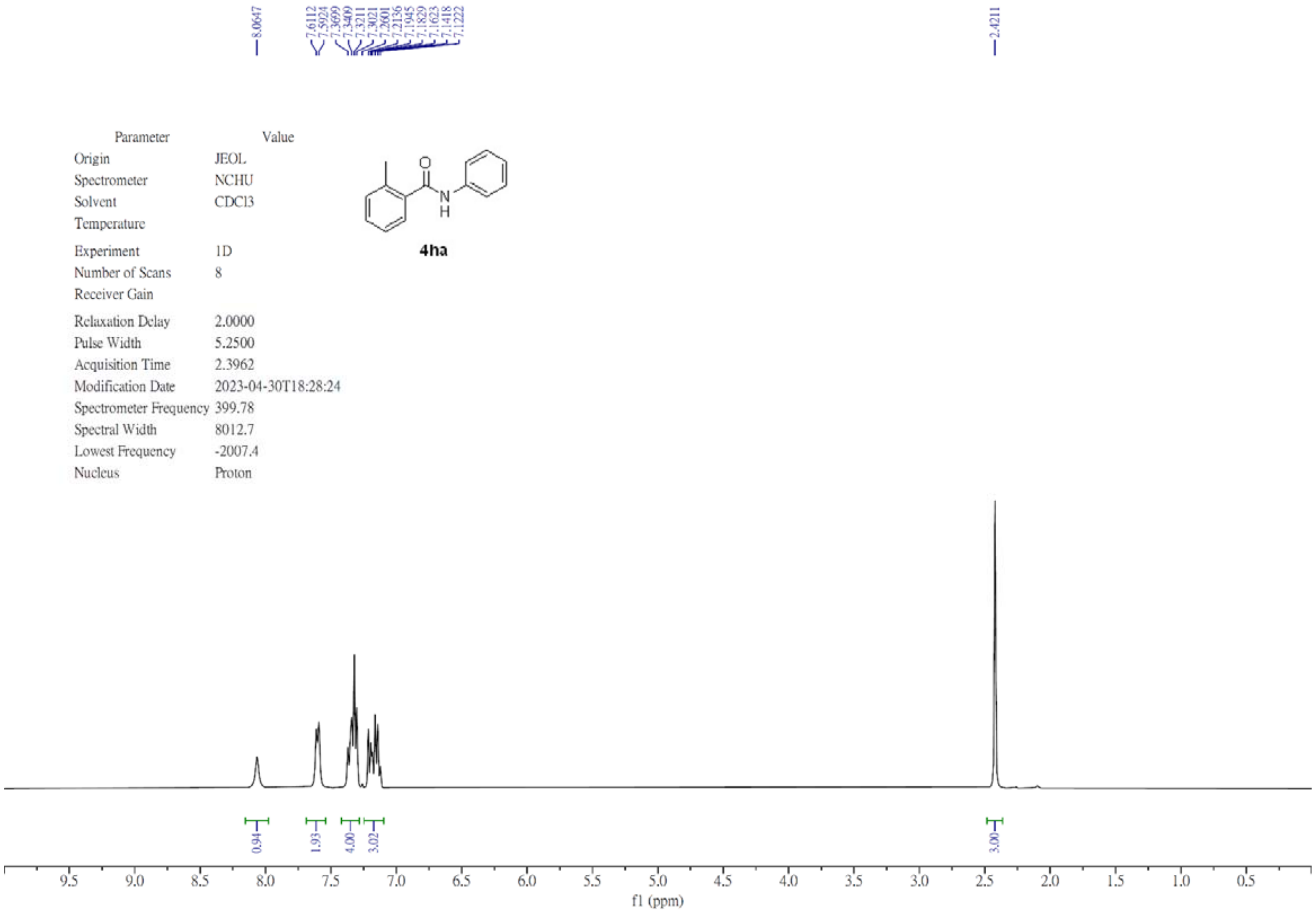

4ha ^{1}H NMR spectrum (400 MHz in $CDCl_3$)

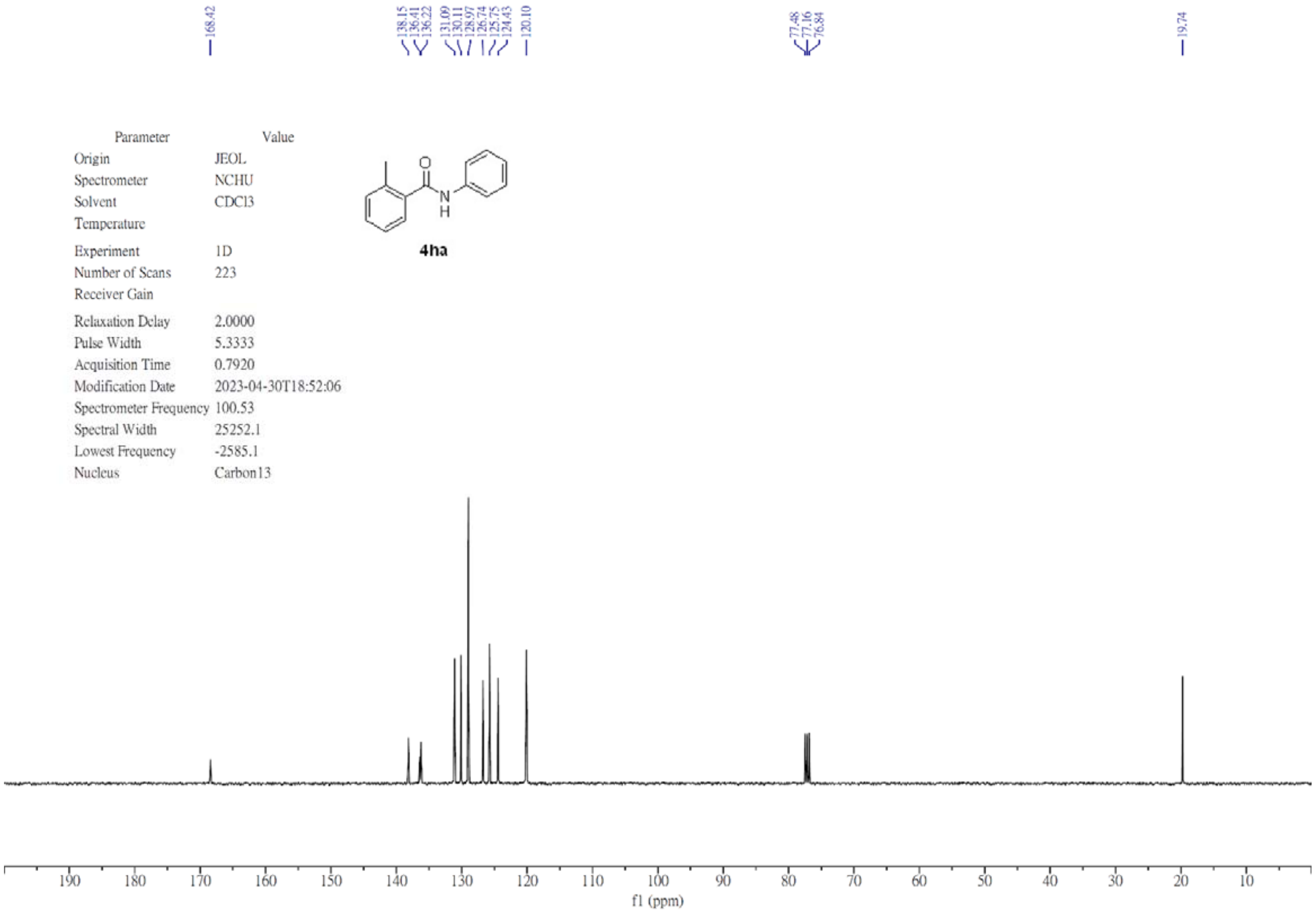

4ha $^{13}C\{^{1}H\}$ NMR spectrum (100 MHz in $(CD_3)_2SO$)

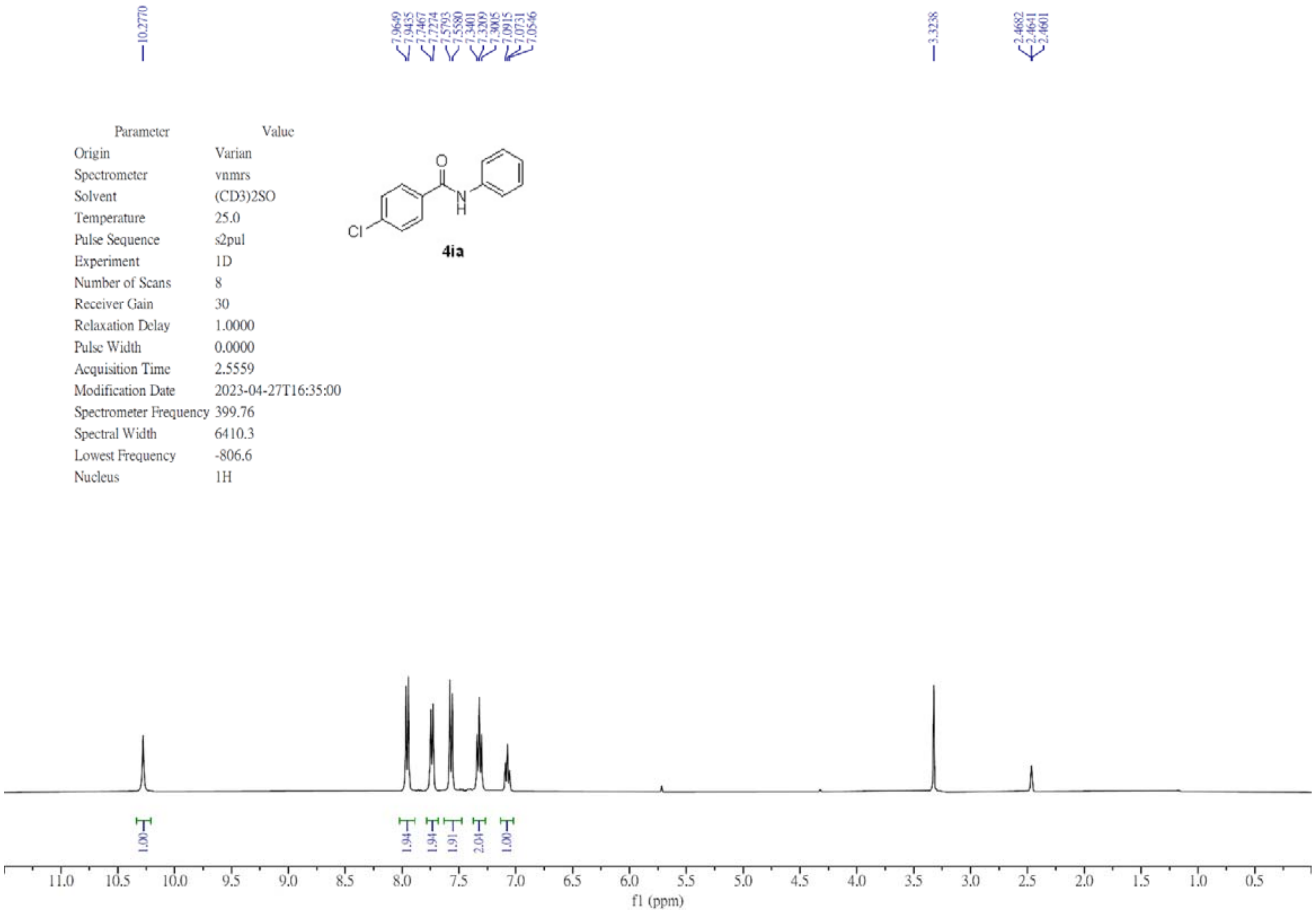

4ia ^{1}H NMR spectrum (400 MHz in $CDCl_3$)

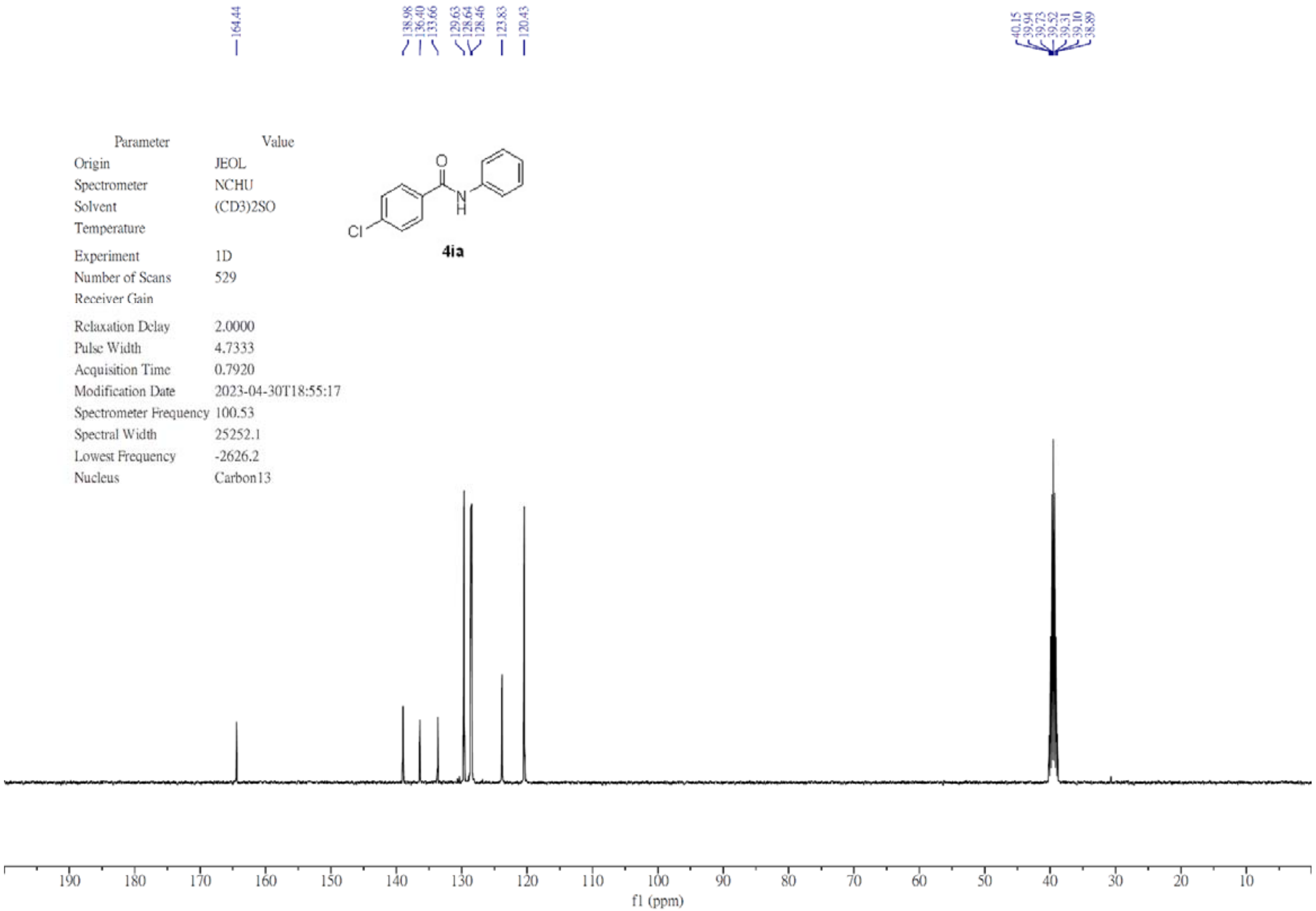

4ia $^{13}C\{^{1}H\}$ NMR spectrum (100 MHz in $CDCl_3$)

Parameter	Value
Origin	JEOL
Spectrometer	NCHU
Solvent	CDCl3
Temperature	
Experiment	1D
Number of Scans	8
Receiver Gain	
Relaxation Delay	2.0000
Pulse Width	5.2500
Acquisition Time	2.3962
Modification Date	2023-04-30T18:26:31
Spectrometer Frequency	399.78
Spectral Width	8012.7
Lowest Frequency	-2007.4
Nucleus	Proton

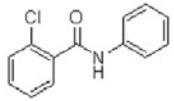

4ja

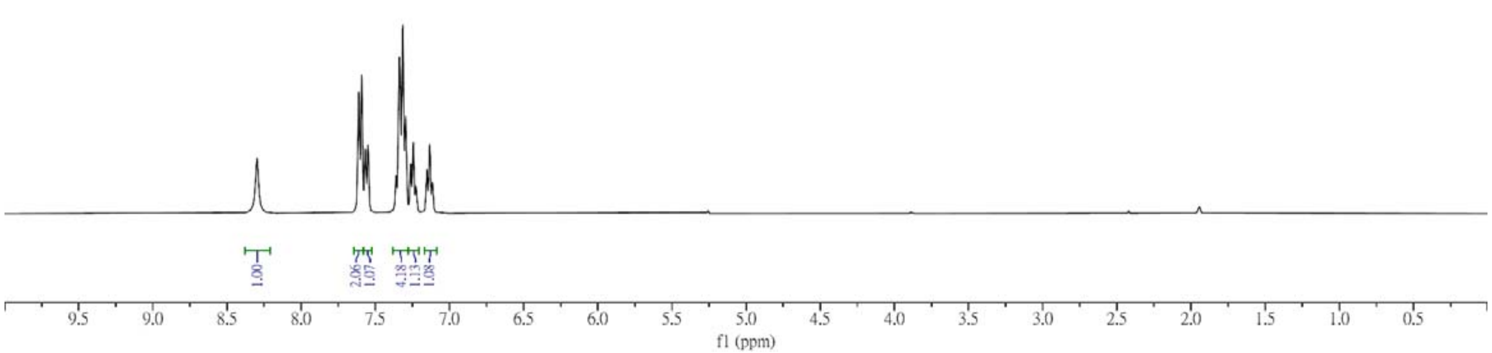

4ja ^{1}H NMR spectrum (400 MHz in $CDCl_3$)

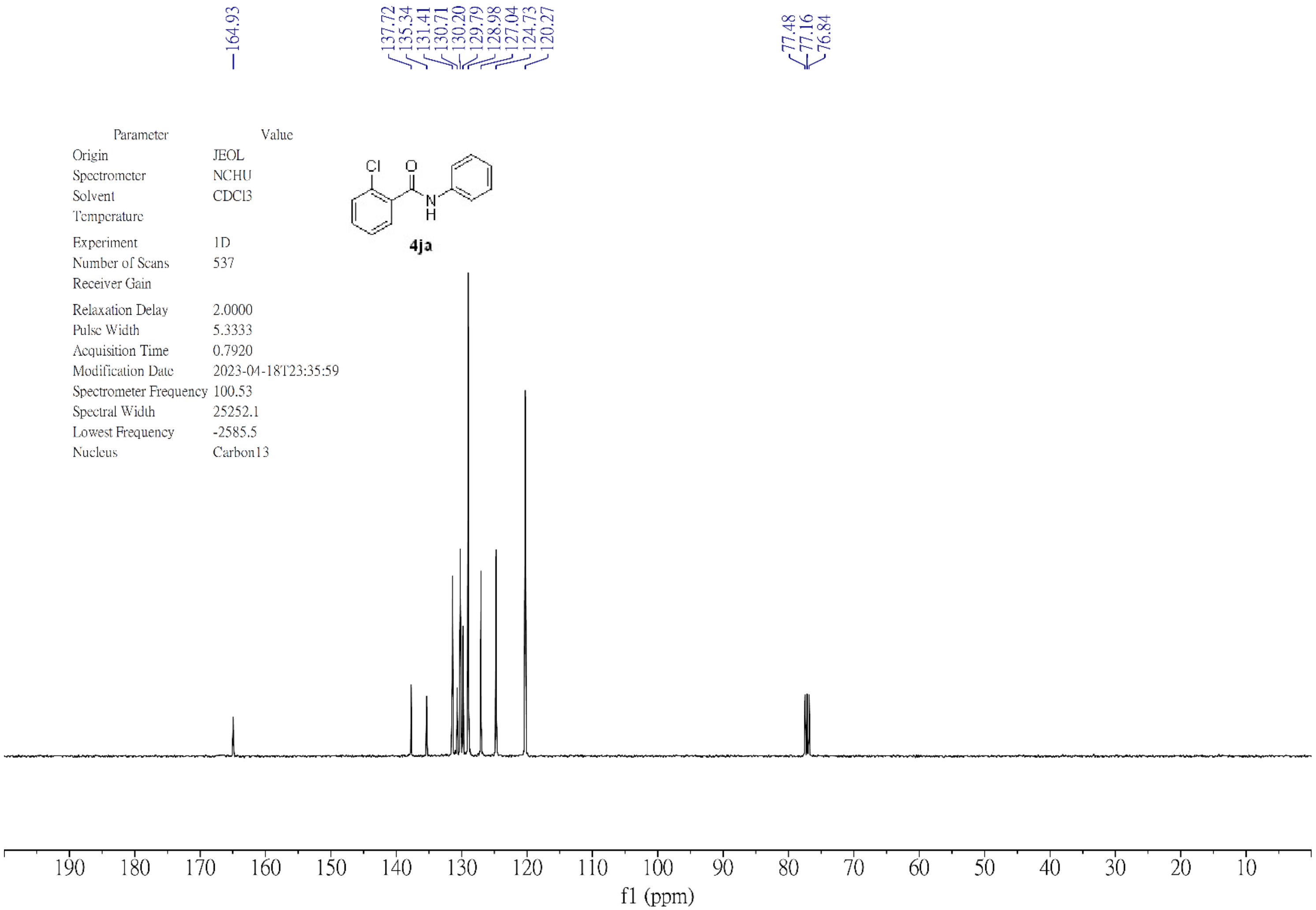

4ja $^{13}C\{^{1}H\}$ NMR spectrum (100 MHz in $CDCl_3$)

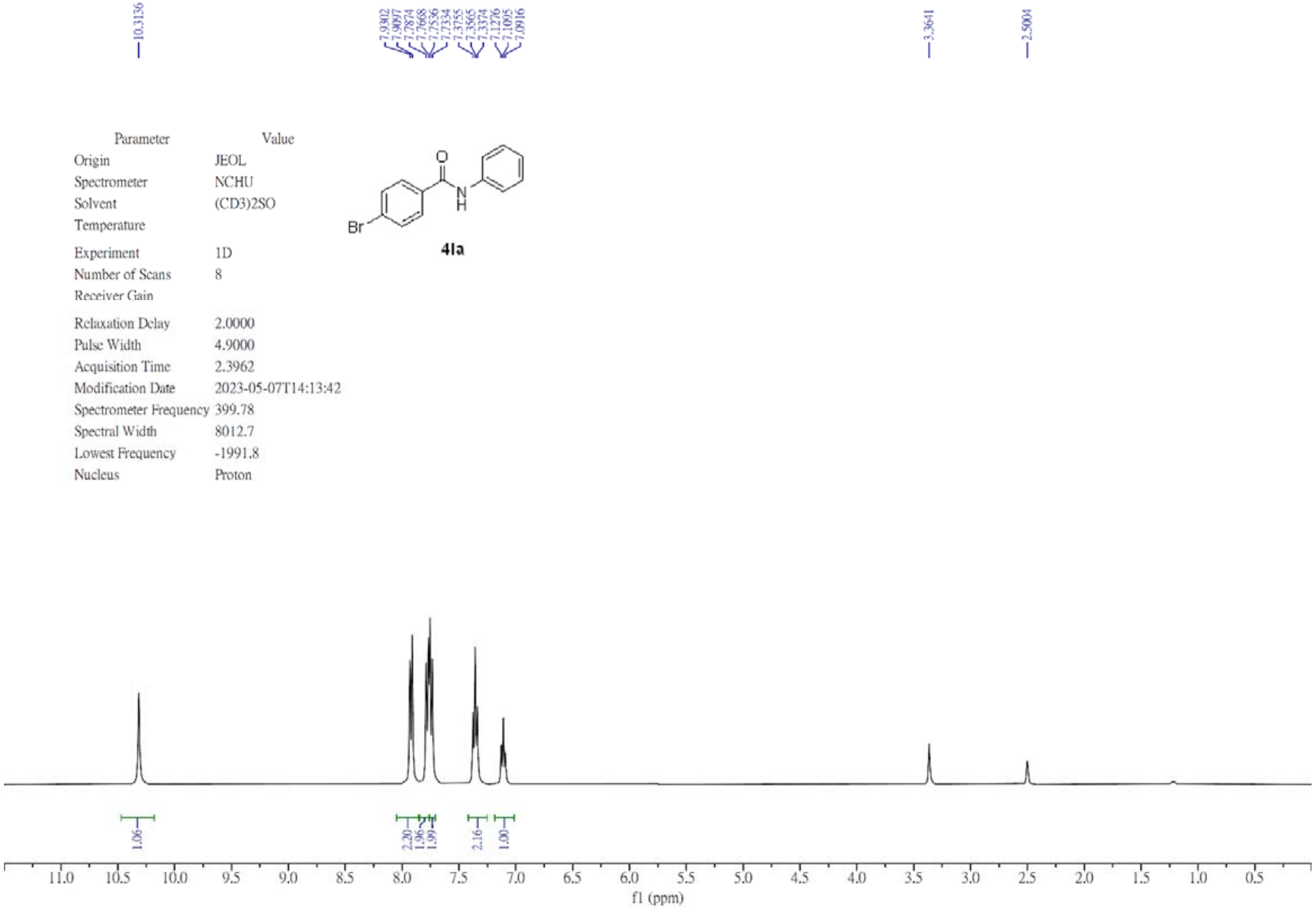

4la ^{1}H NMR spectrum (400 MHz in $CDCl_3$)

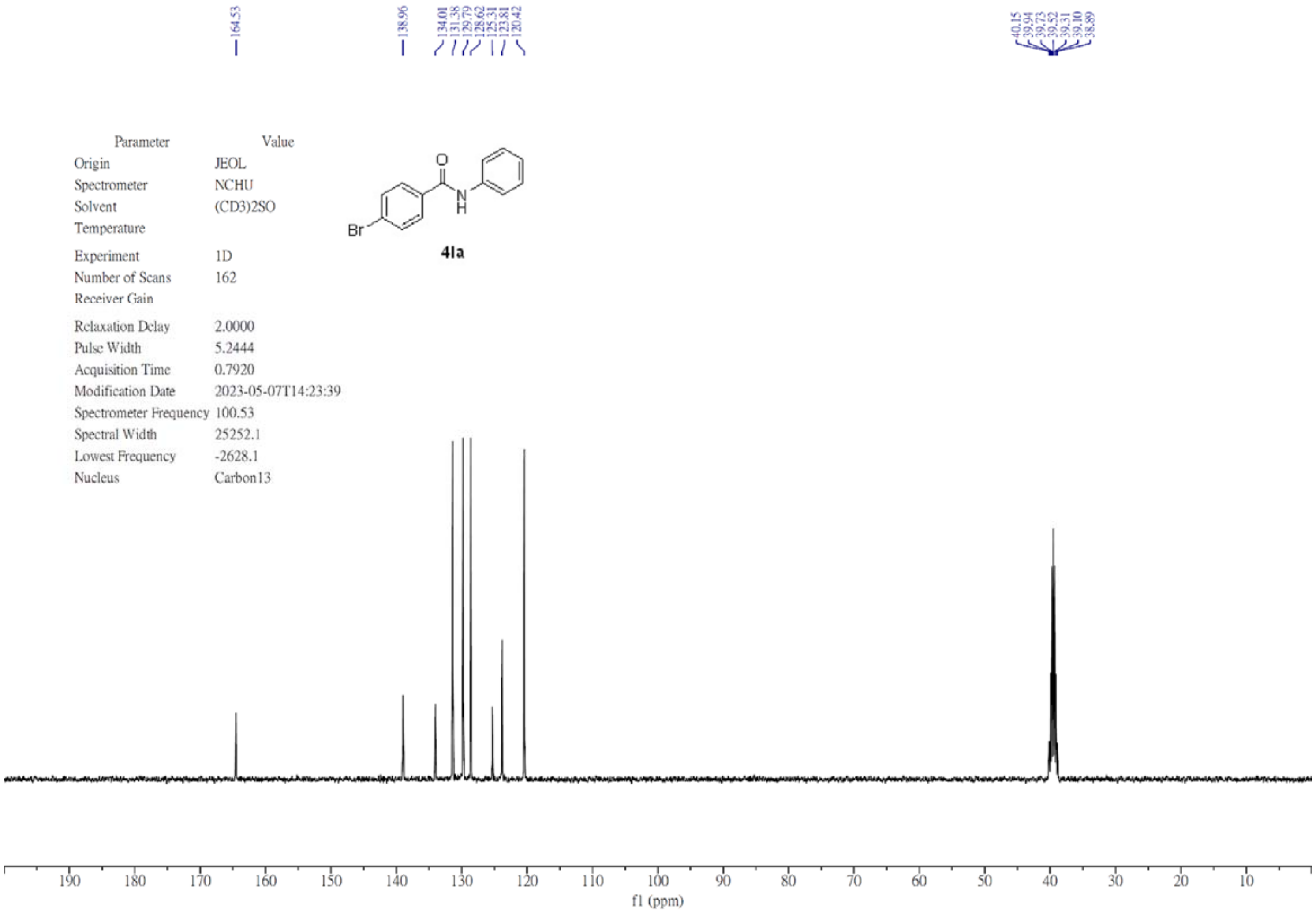

4la $^{13}C\{^{1}H\}$ NMR spectrum (100 MHz in $CDCl_3$)

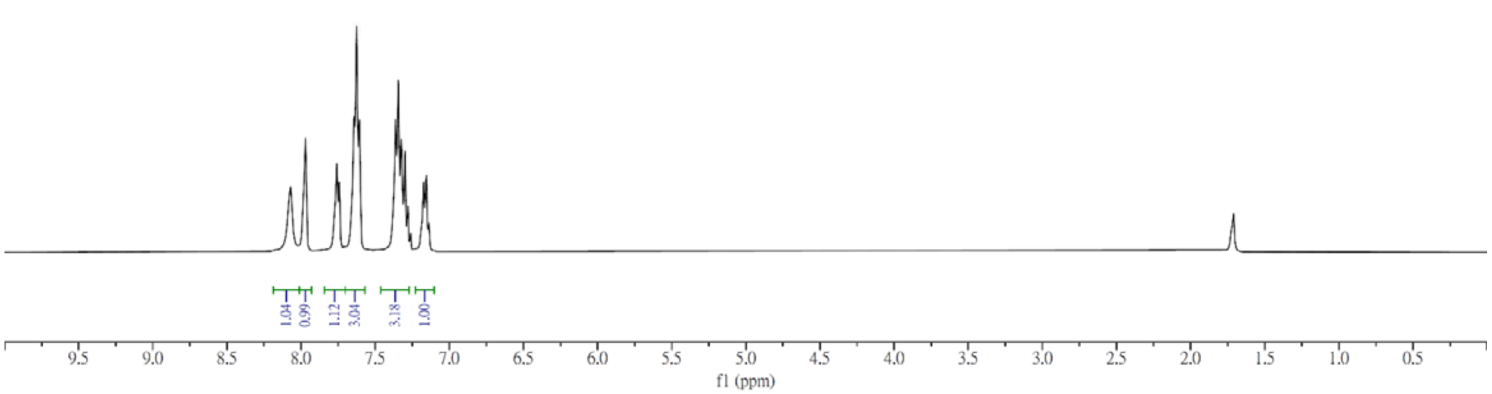

Parameter	Value
Origin	JEOL
Spectrometer	NCHU
Solvent	CDCl3
Temperature	
Experiment	1D
Number of Scans	8
Receiver Gain	
Relaxation Delay	2.0000
Pulse Width	4.9000
Acquisition Time	2.3962
Modification Date	2023-05-07T14:25:28
Spectrometer Frequency	399.78
Spectral Width	8012.7
Lowest Frequency	-2000.6
Nucleus	Proton

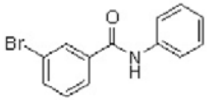

4ma

4ma ^{1}H NMR spectrum (400 MHz in $CDCl_3$)

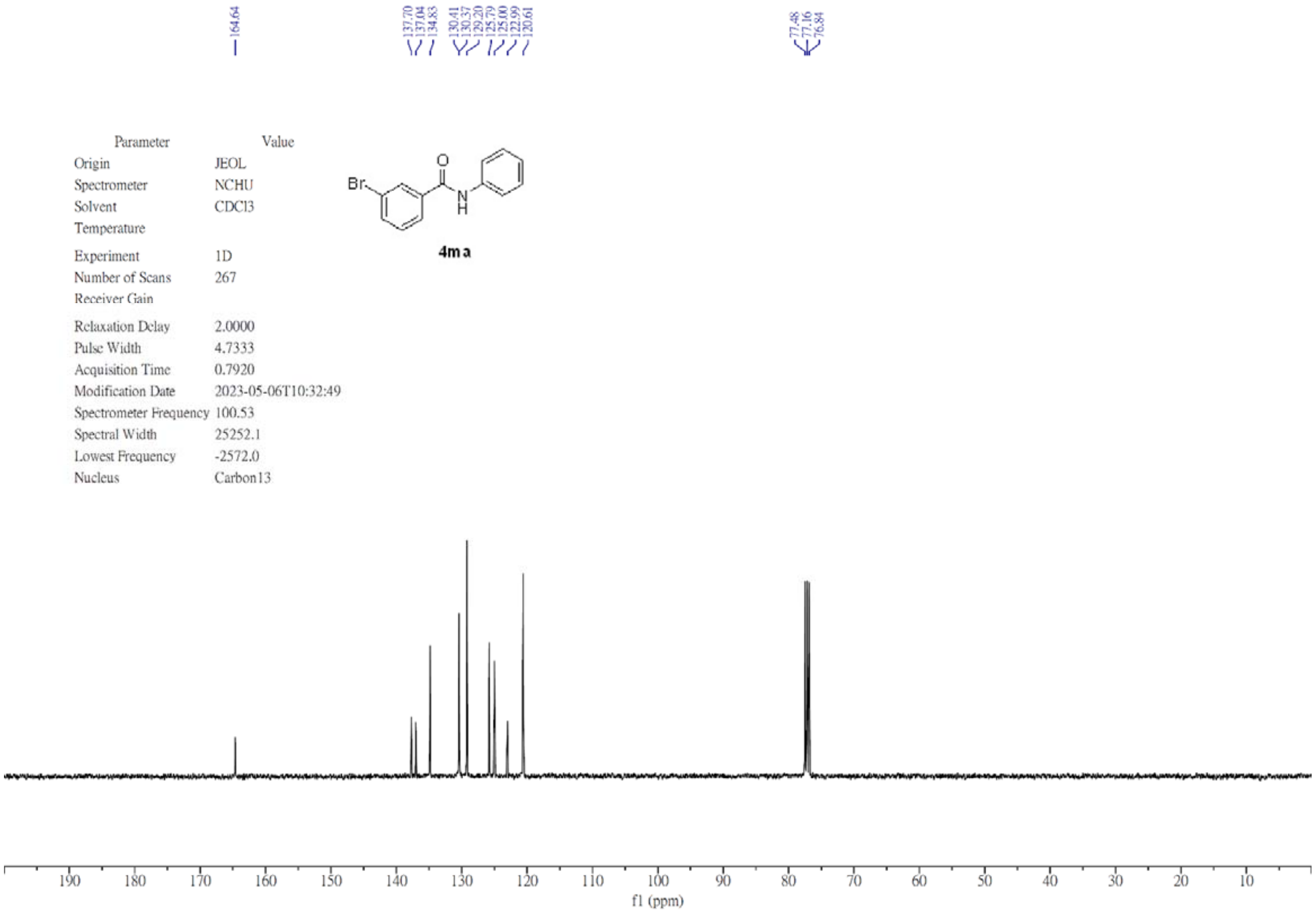

4ma $^{13}C\{^{1}H\}$ NMR spectrum (100 MHz in $CDCl_3$)

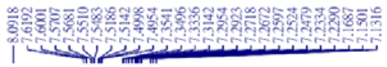

Parameter	Value
Origin	JEOL
Spectrometer	NCHU
Solvent	CDCl3
Temperature	
Experiment	1D
Number of Scans	8
Receiver Gain	
Relaxation Delay	2.0000
Pulse Width	4.9000
Acquisition Time	2.3962
Modification Date	2023-05-19T18:27:17
Spectrometer Frequency	399.78
Spectral Width	8012.7
Lowest Frequency	-2002.6
Nucleus	Proton

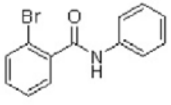

4na

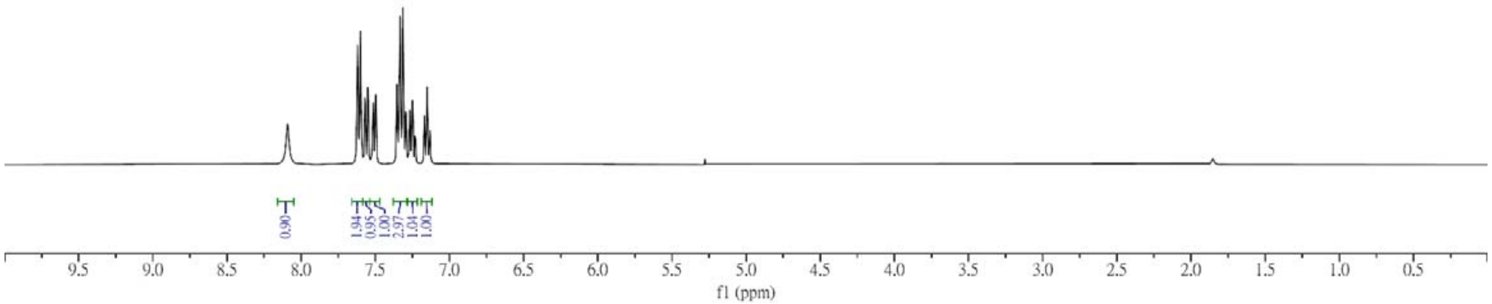

4na ^{1}H NMR spectrum (400 MHz in $CDCl_3$)

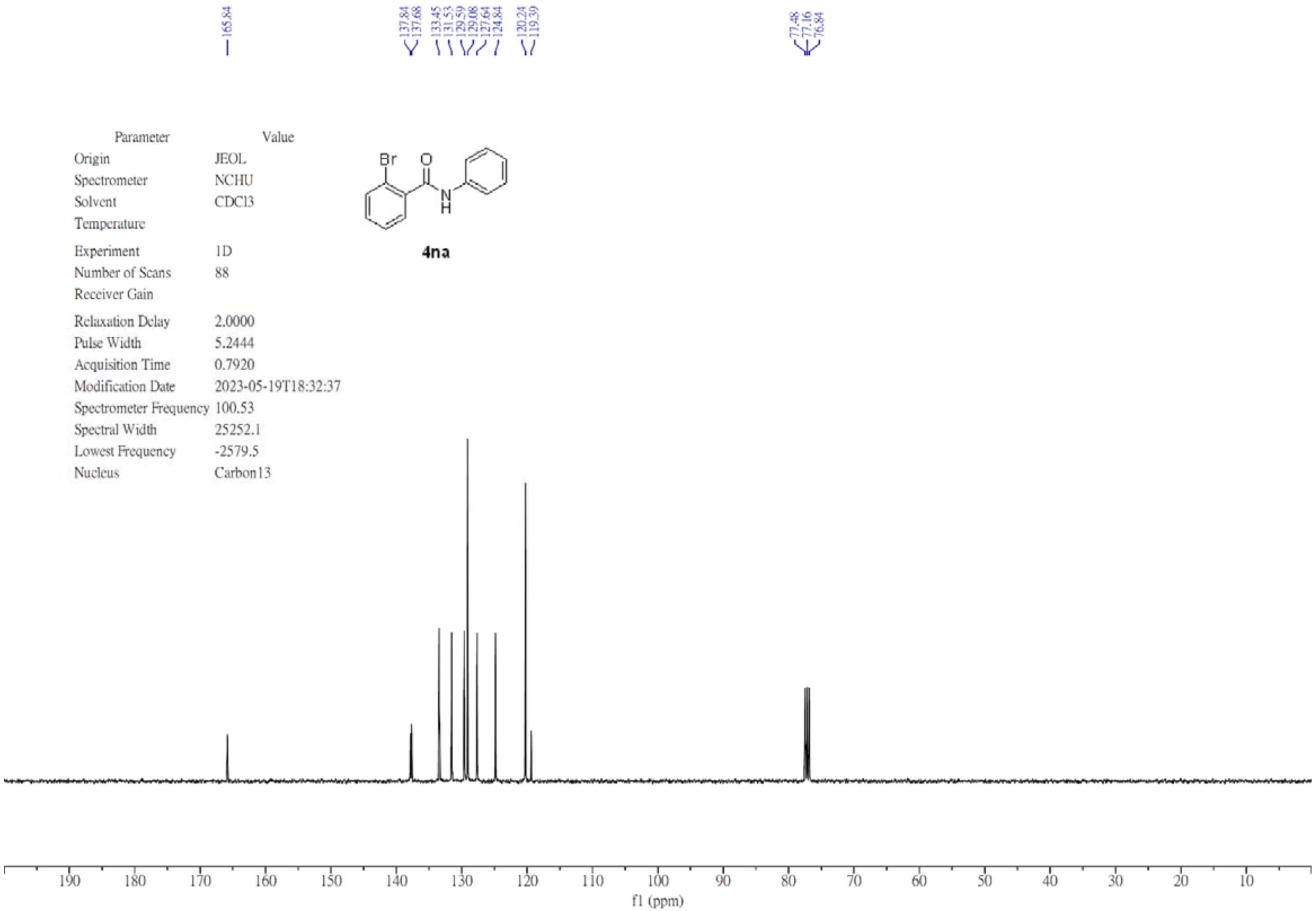

4na $^{13}C\{^{1}H\}$ NMR spectrum (100 MHz in $CDCl_3$)

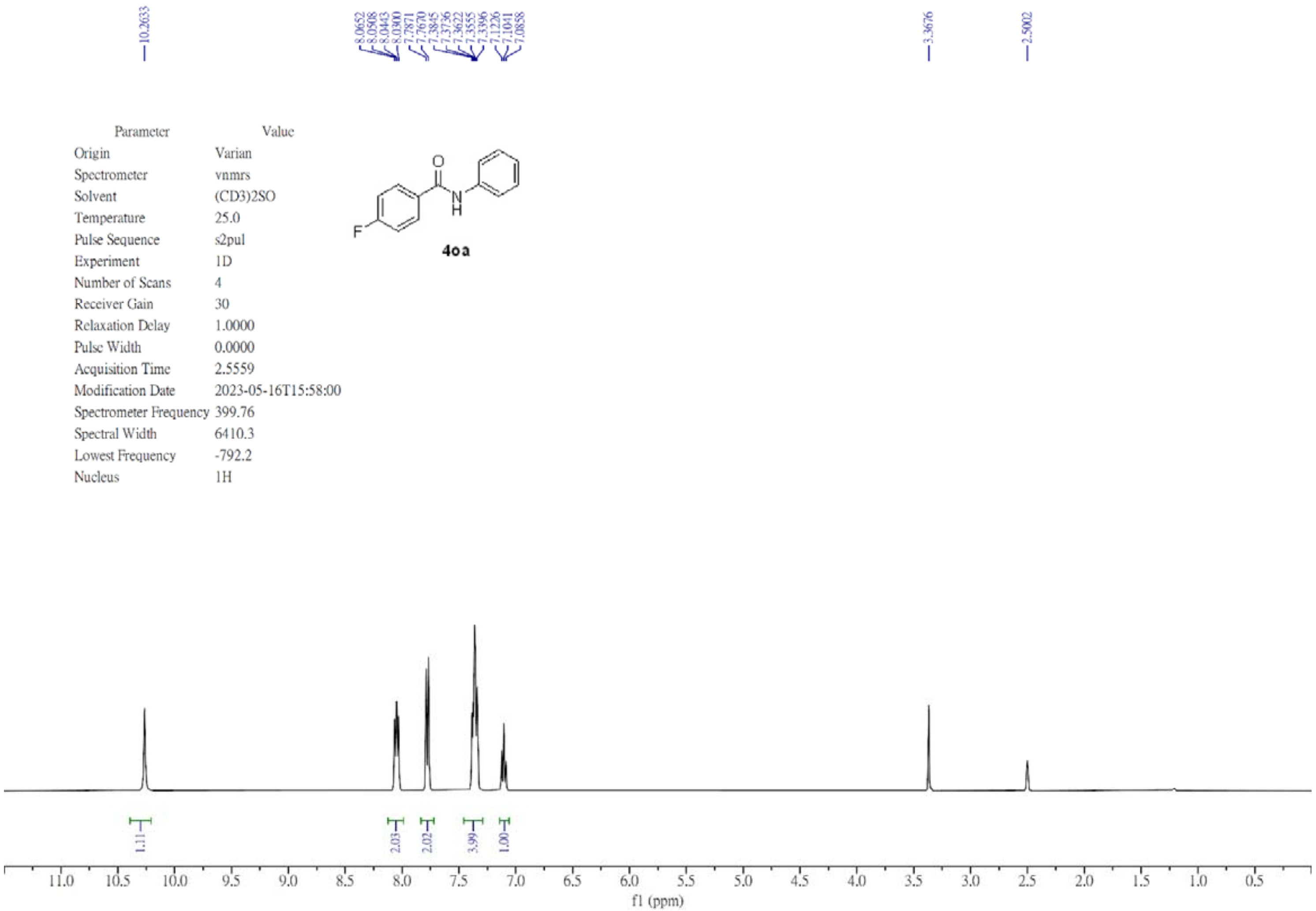

4oa ^{1}H NMR spectrum (400 MHz in $CDCl_3$)

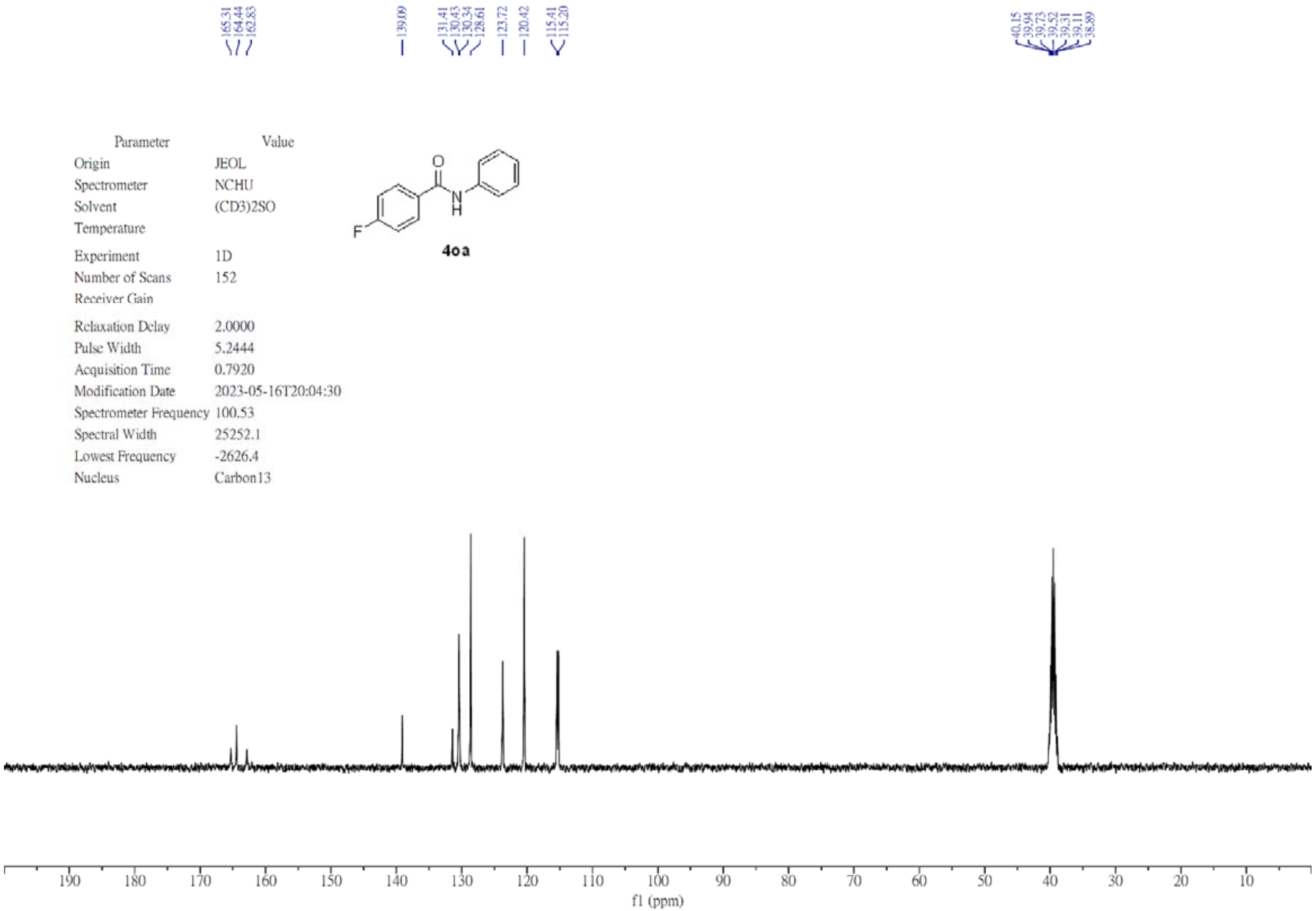

4oa $^{13}C\{^{1}H\}$ NMR spectrum (100 MHz in $(CD_3)_2SO$)

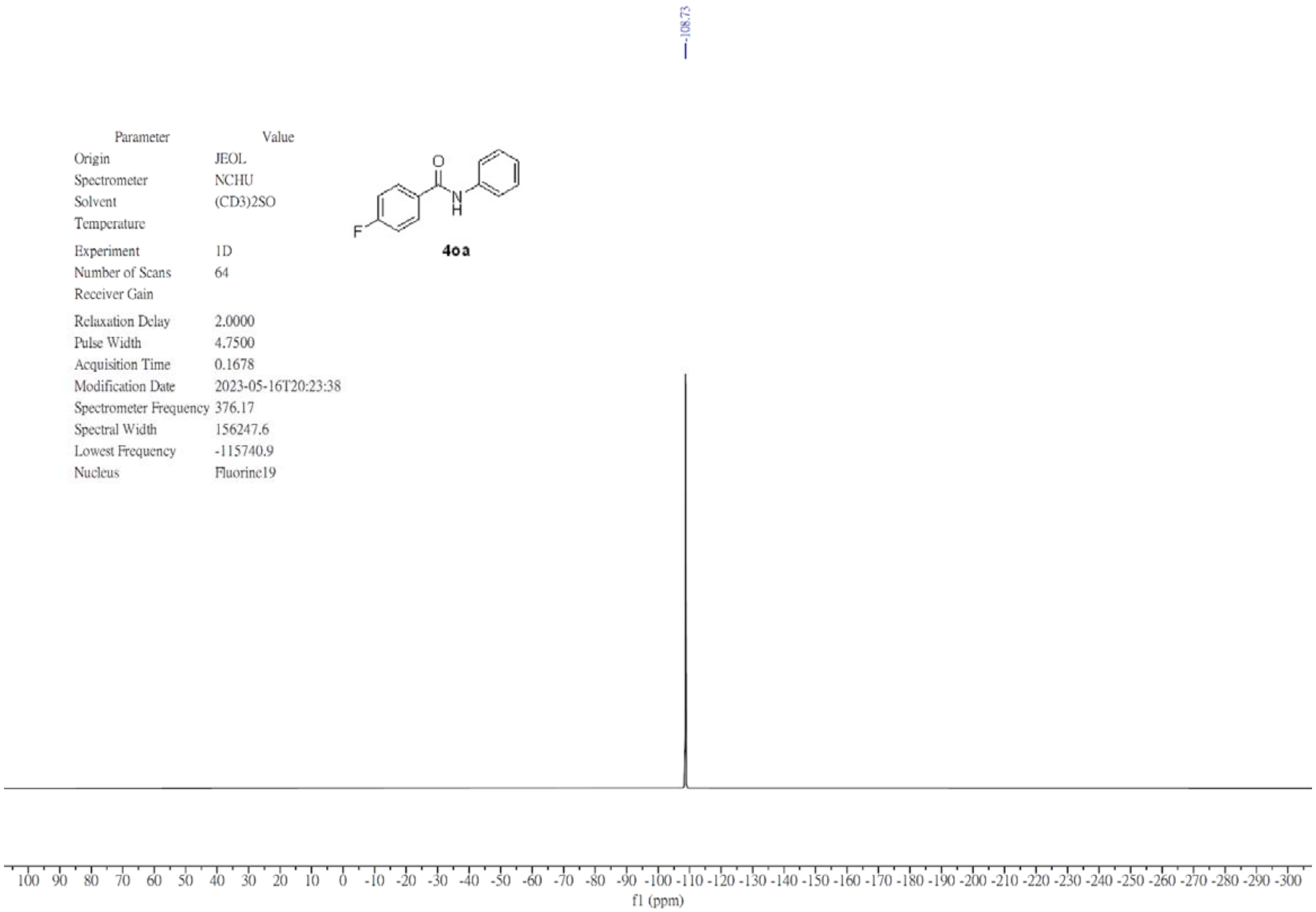

4oa ^{19}F NMR spectrum (376 MHz in $CDCl_3$)

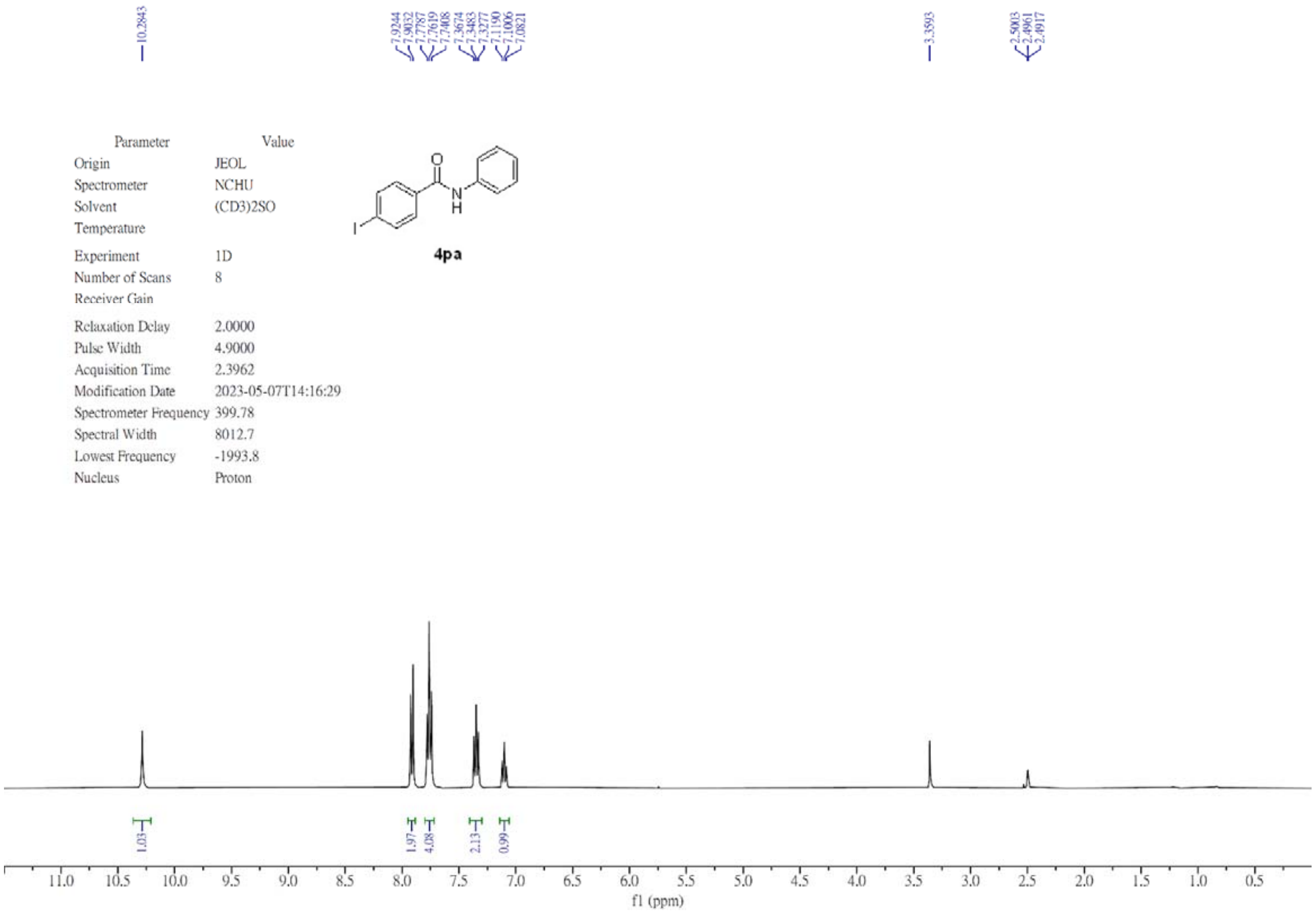

4pa ^{1}H NMR spectrum (400 MHz in $CDCl_3$)

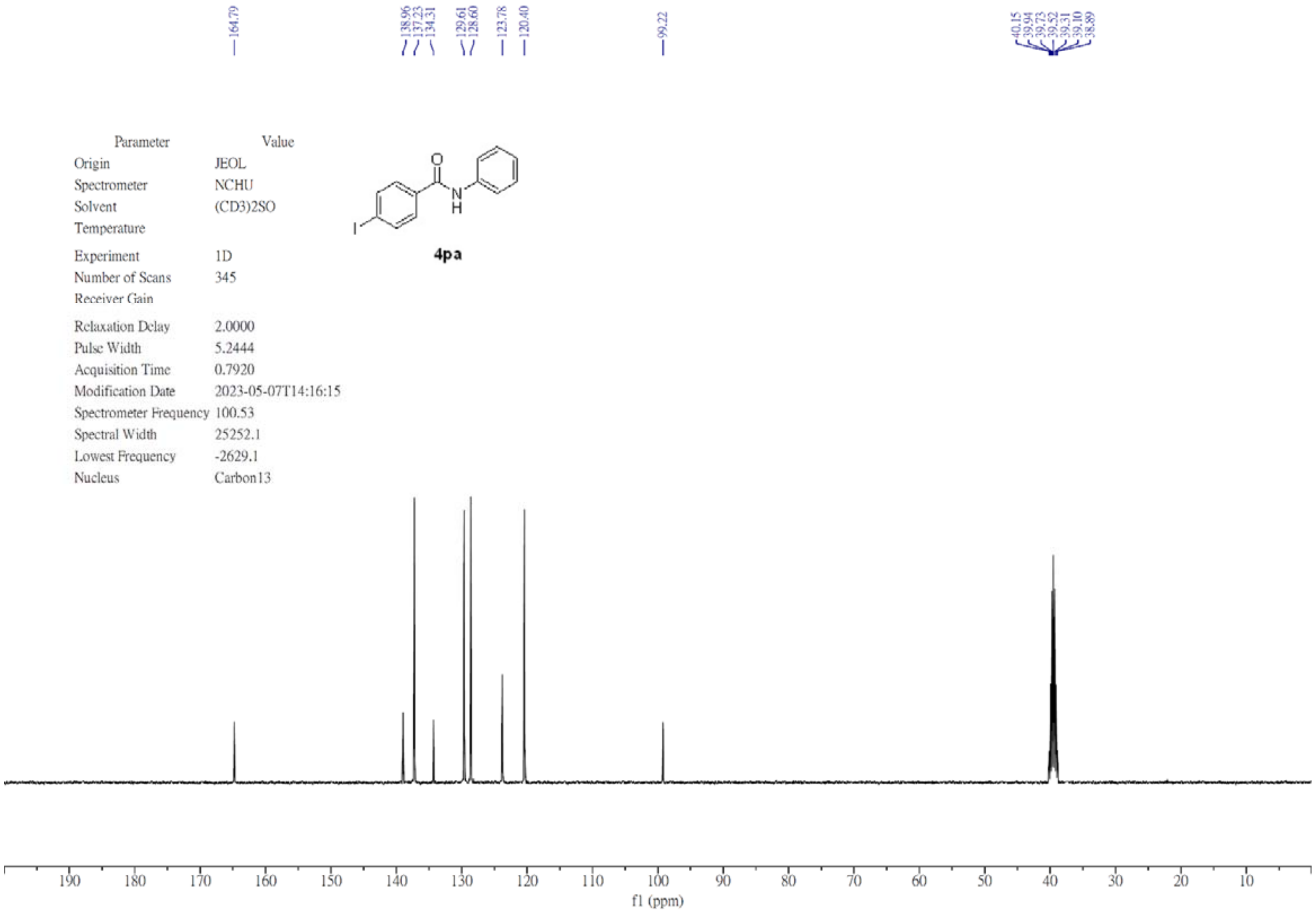

4pa $^{13}C\{^{1}H\}$ NMR spectrum (100 MHz in $CDCl_3$)

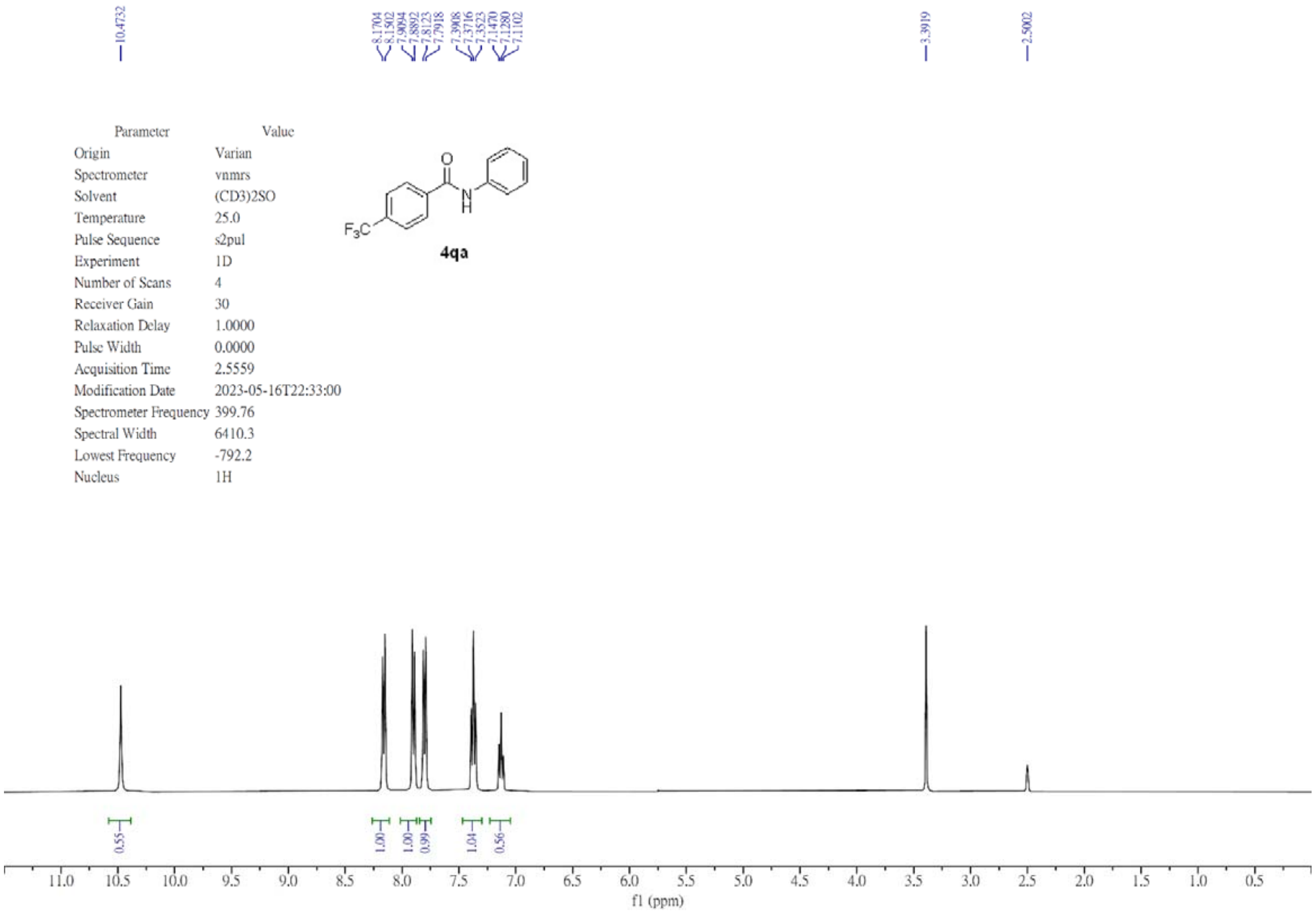

4qa ^{1}H NMR spectrum (400 MHz in $CDCl_3$)

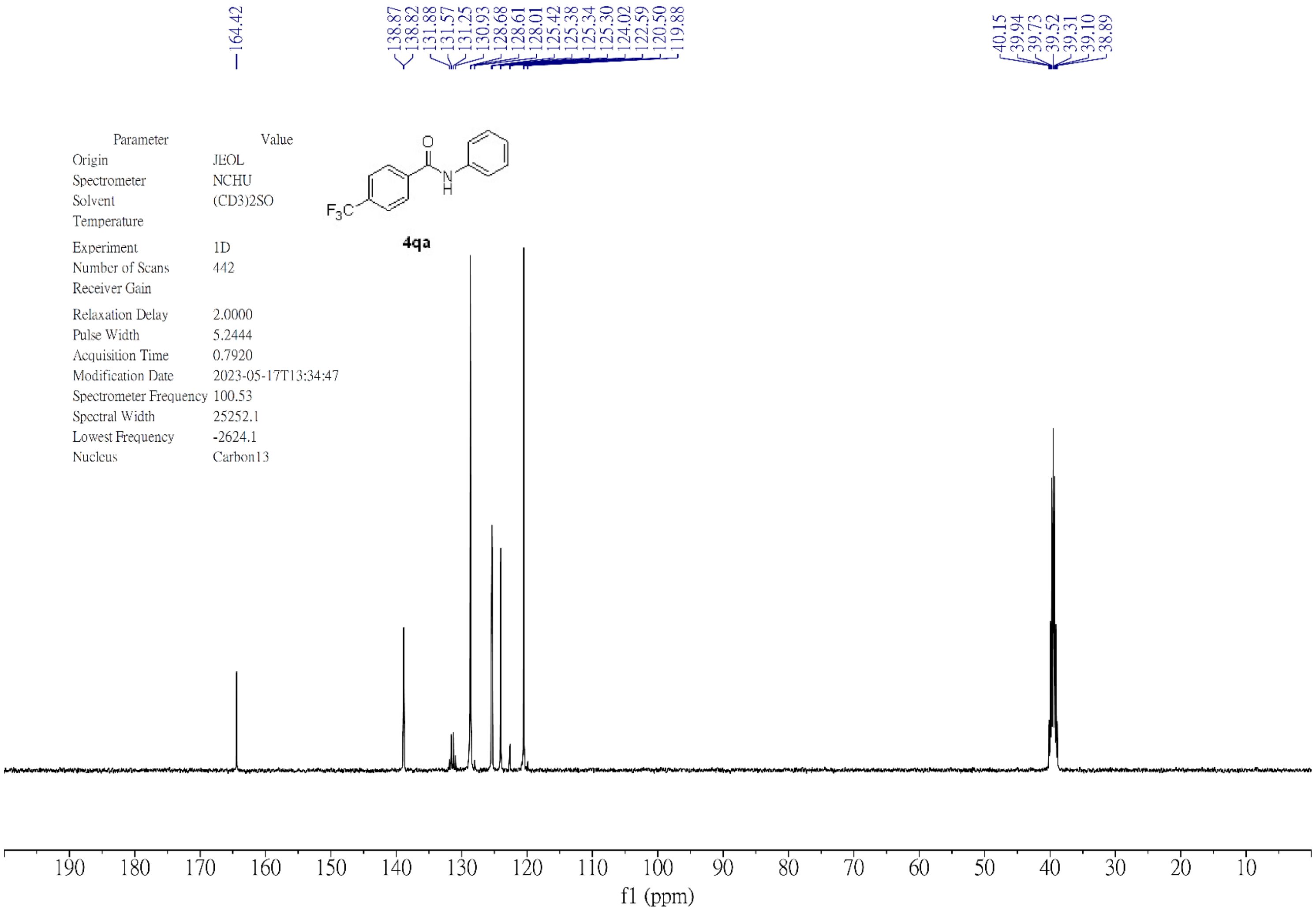

4qa $^{13}C\{^{1}H\}$ NMR spectrum (100 MHz in $CDCl_3$)

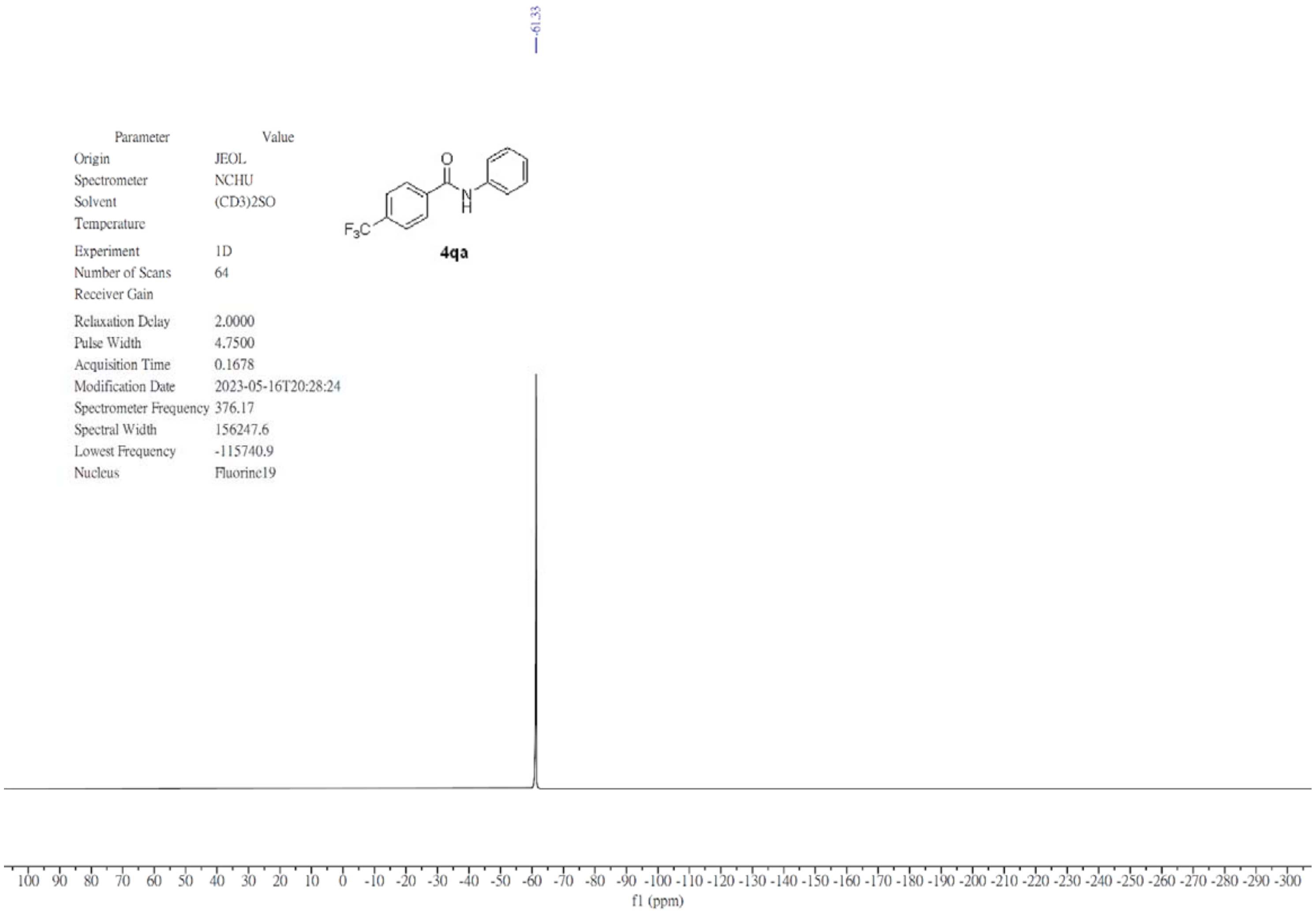

4qa ^{19}F NMR spectrum (376 MHz in $CDCl_3$)

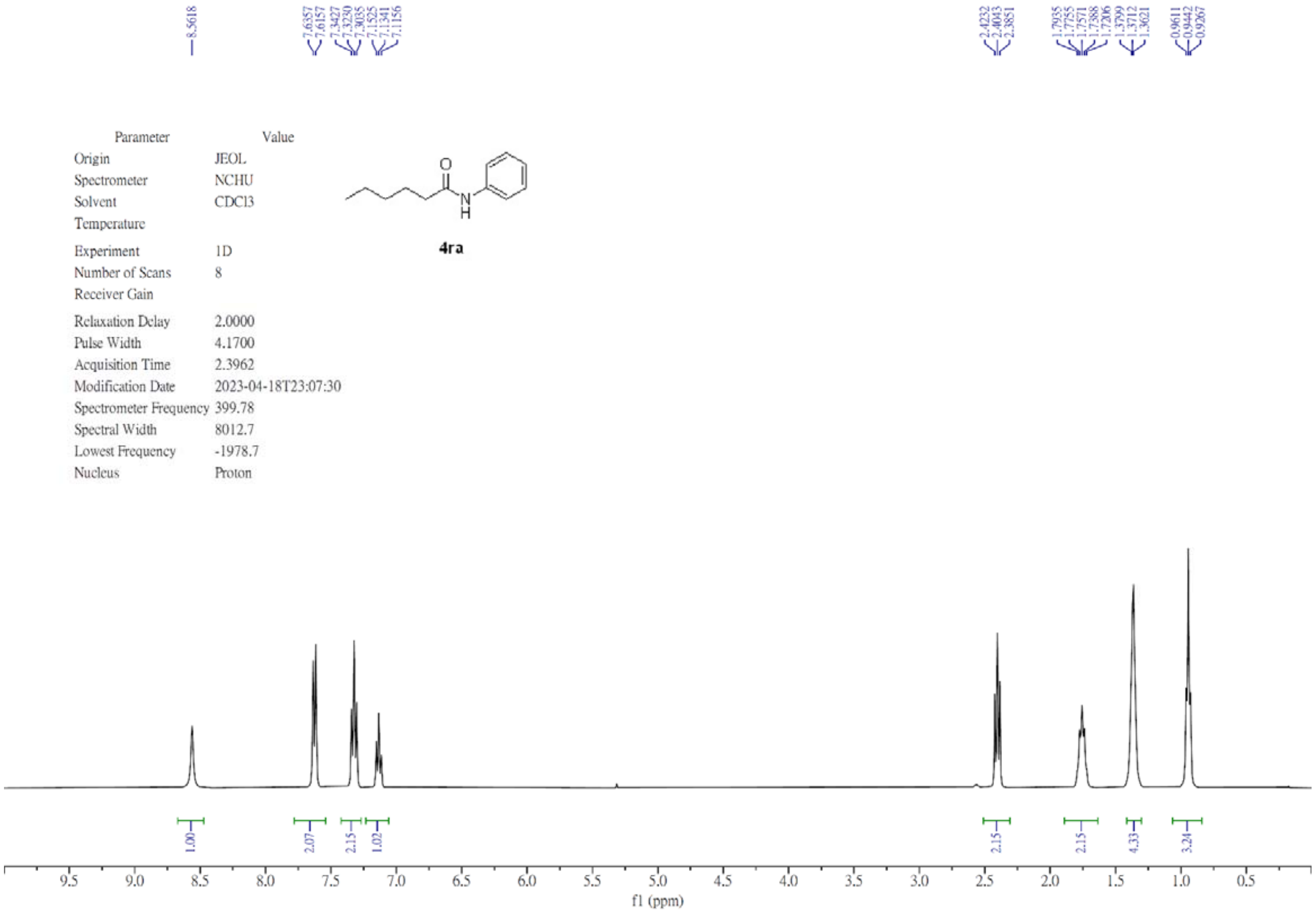

4ra ^{1}H NMR spectrum (400 MHz in $(CD_3)_2SO$)

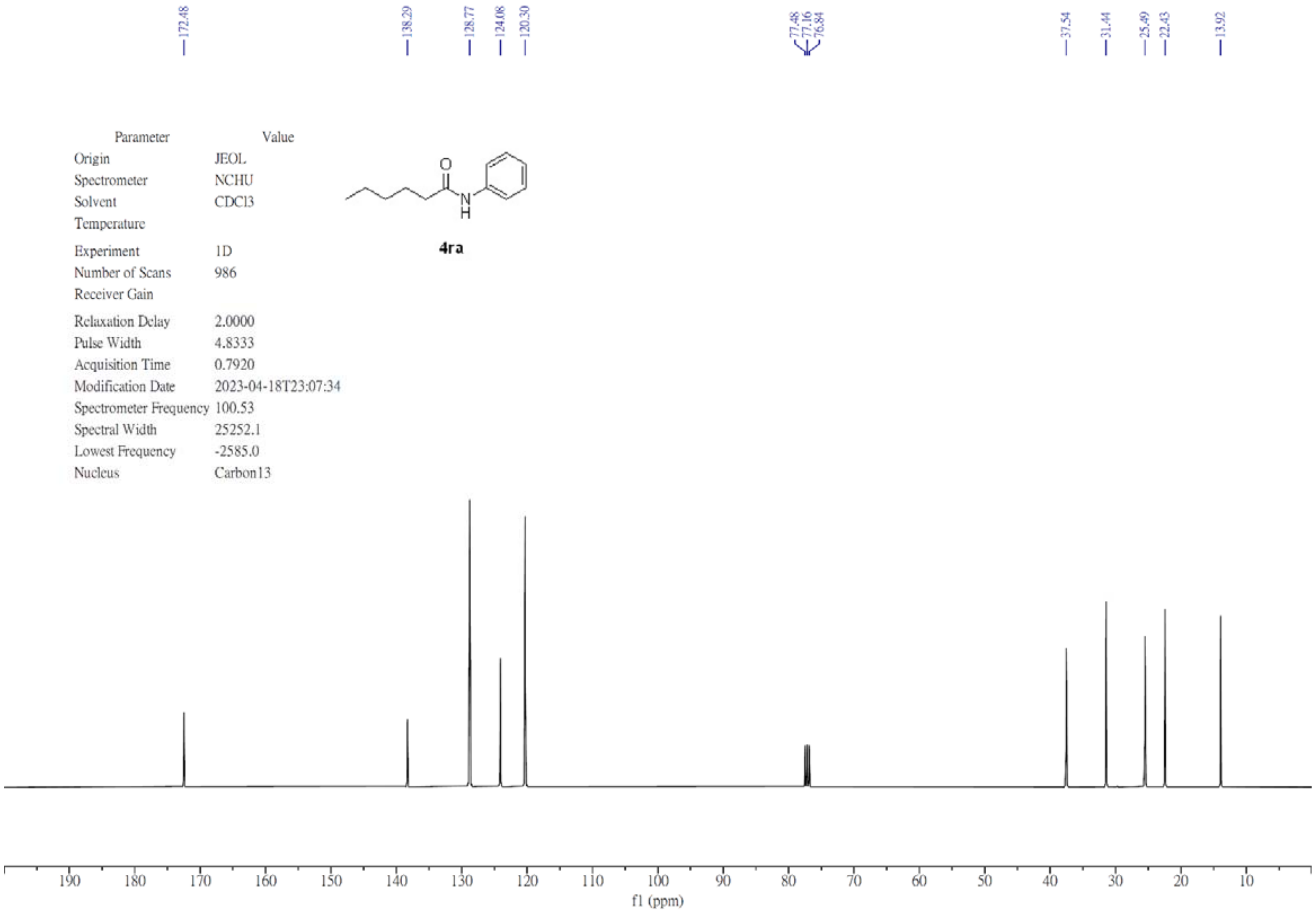

4ra $^{13}C\{^{1}H\}$ NMR spectrum (100 MHz in $(CD_3)_2SO$)

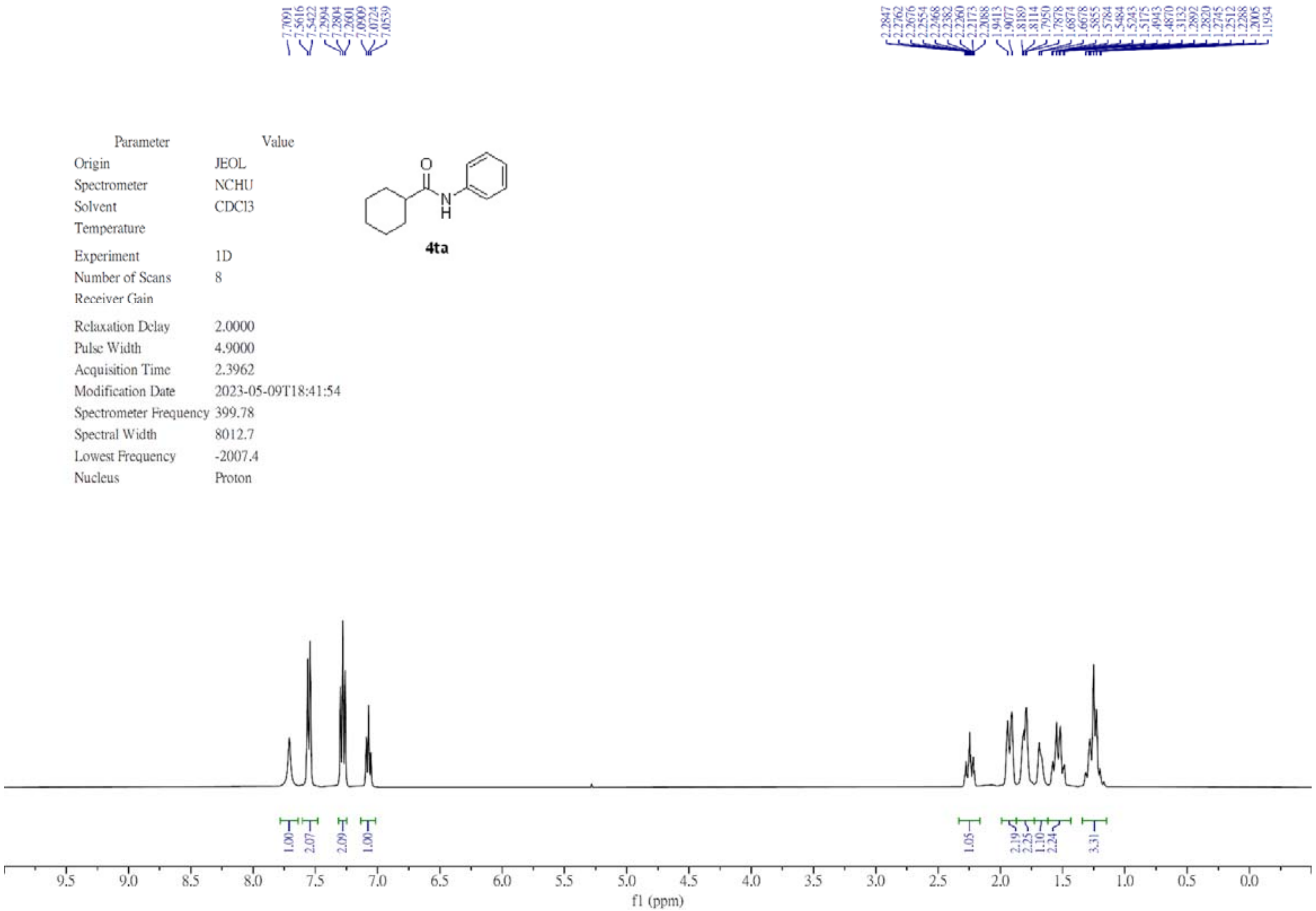

4ta ^{1}H NMR spectrum (400 MHz in $(CD_3)_2SO$)

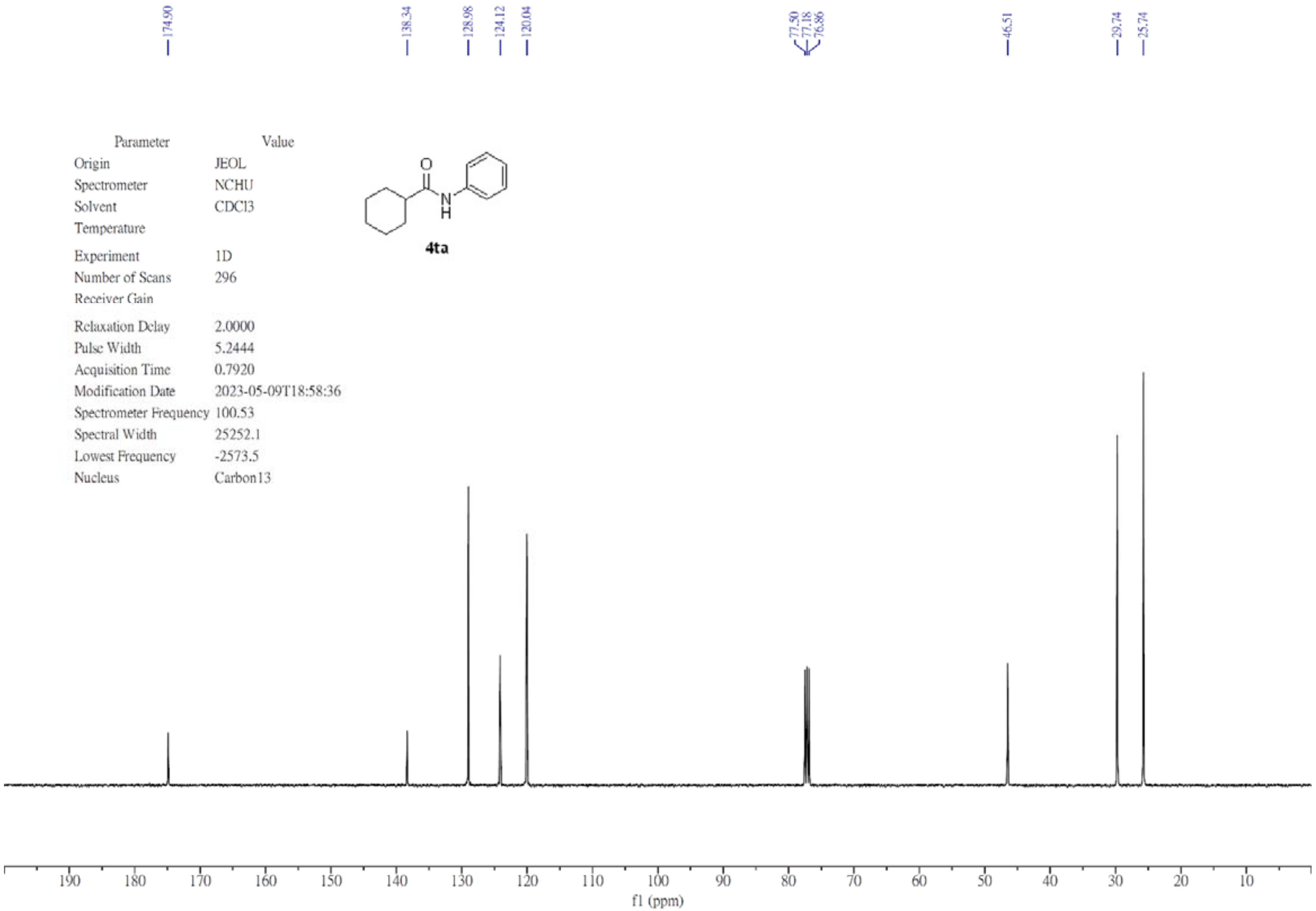

4ta $^{13}C\{^{1}H\}$ NMR spectrum (100 MHz in $(CD_3)_2SO$)

Parameter	Value
Origin	Varian
Spectrometer	vnmrs
Solvent	CDCl3
Temperature	25.0
Pulse Sequence	s2pul
Experiment	1D
Number of Scans	32
Receiver Gain	30
Relaxation Delay	1.0000
Pulse Width	0.0000
Acquisition Time	2.5559
Modification Date	2023-05-02T16:24:00
Spectrometer Frequency	399.76
Spectral Width	6410.3
Lowest Frequency	-799.0
Nucleus	1H

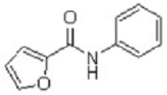

4ua

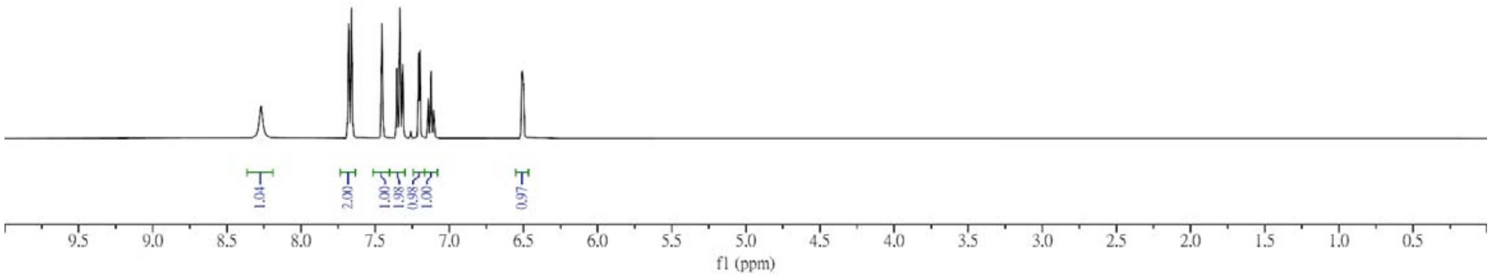

4ua ^{1}H NMR spectrum (400 MHz in $CDCl_3$)

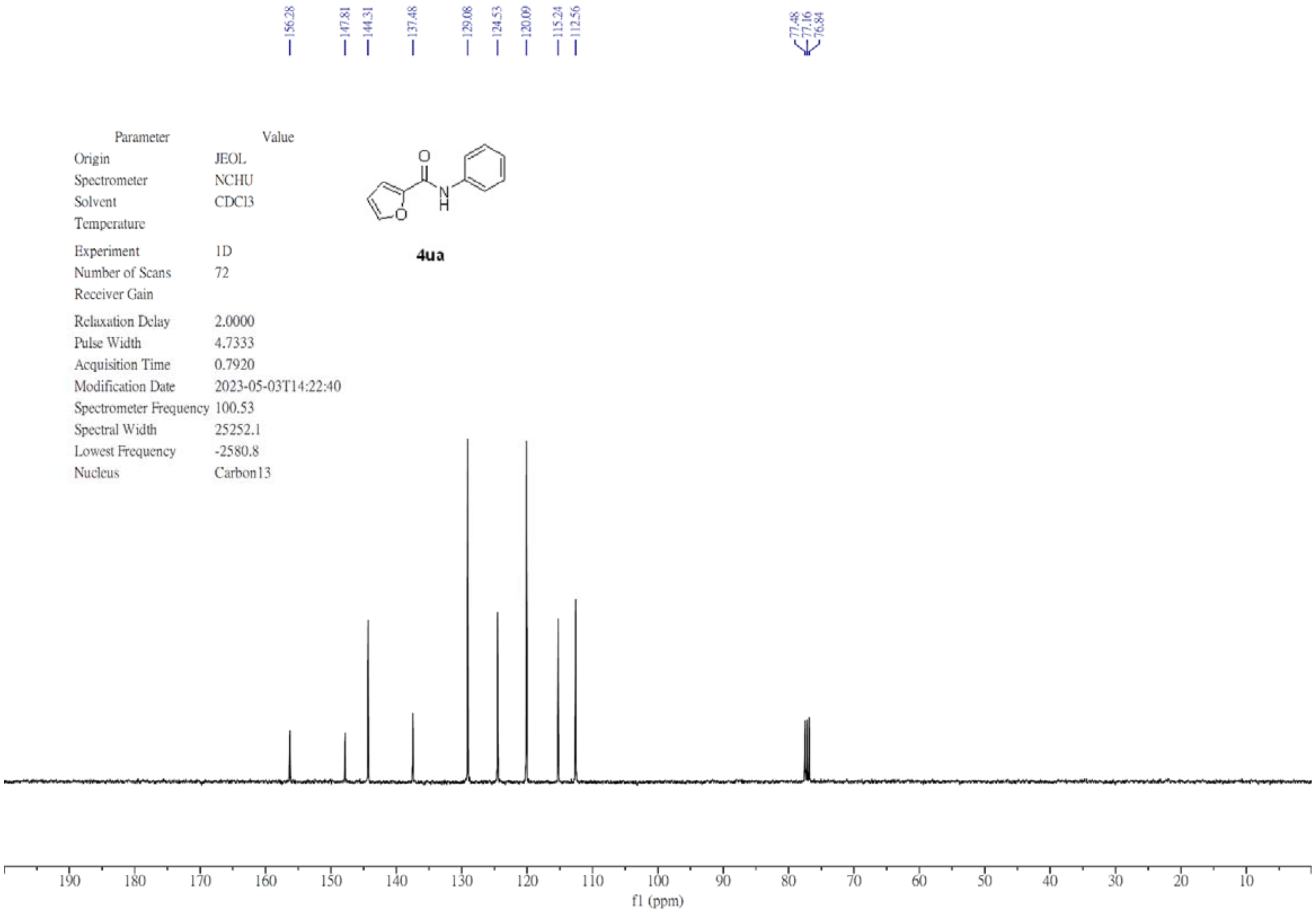

4ua $^{13}C\{^{1}H\}$ NMR spectrum (100 MHz in $CDCl_3$)

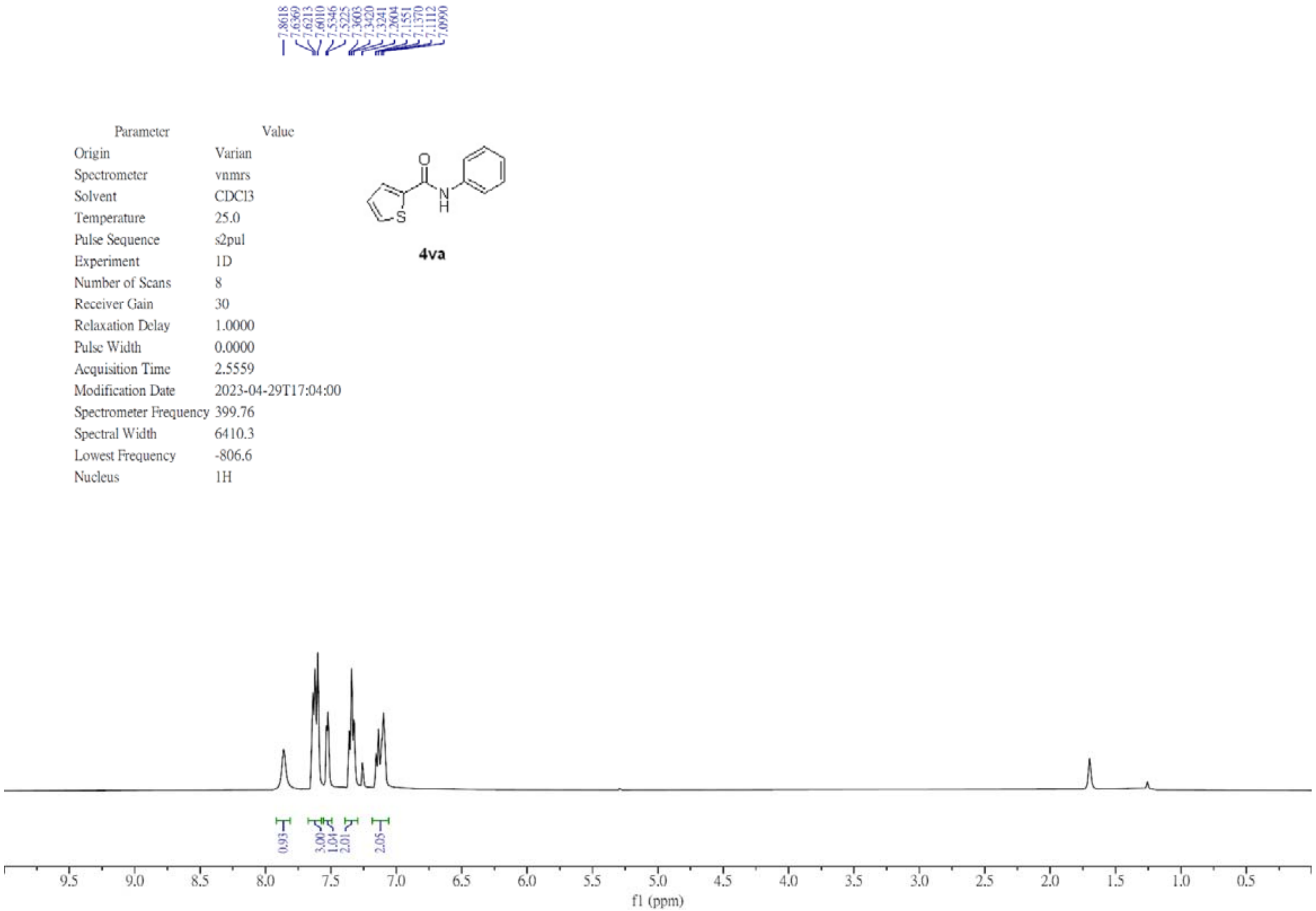

4va ^{1}H NMR spectrum (400 MHz in $CDCl_3$)

Parameter	Value
Origin	JEOL
Spectrometer	NCHU
Solvent	CDCl3
Temperature	
Experiment	1D
Number of Scans	560
Receiver Gain	
Relaxation Delay	2.0000
Pulse Width	4.7333
Acquisition Time	0.7920
Modification Date	2023-04-30T19:00:37
Spectrometer Frequency	100.53
Spectral Width	25252.1
Lowest Frequency	-2570.5
Nucleus	Carbon13

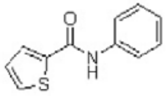

4va

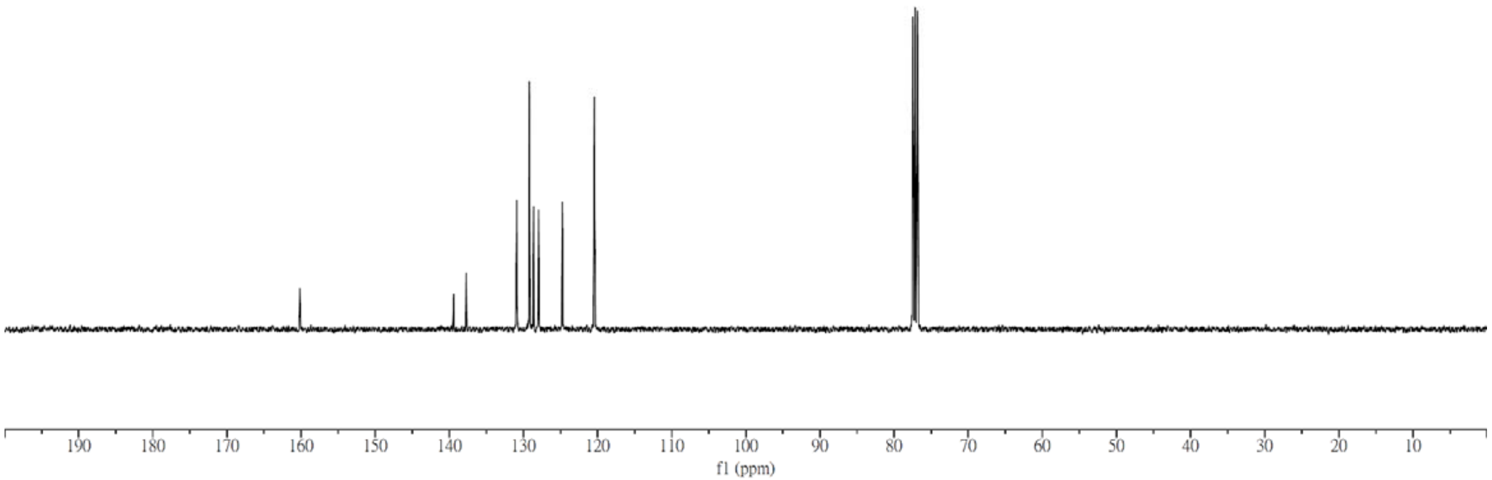

4va $^{13}C\{^{1}H\}$ NMR spectrum (100 MHz in $CDCl_3$)

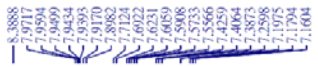

Parameter	Value
Origin	Varian
Spectrometer	vnmrs
Solvent	CDCl3
Temperature	25.0
Pulse Sequence	s2pul
Experiment	1D
Number of Scans	24
Receiver Gain	52
Relaxation Delay	1.0000
Pulse Width	0.0000
Acquisition Time	2.5559
Modification Date	2023-05-16T15:25:00
Spectrometer Frequency	399.76
Spectral Width	6410.3
Lowest Frequency	-799.4
Nucleus	1H

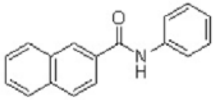

4wa

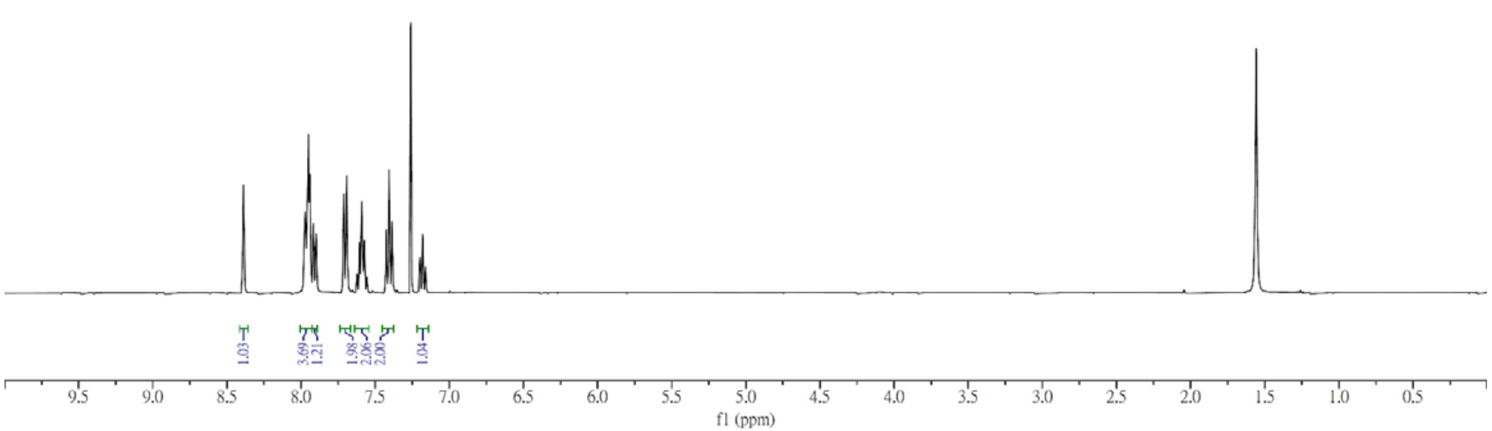

4wa ^{1}H NMR spectrum (400 MHz in $CDCl_3$)

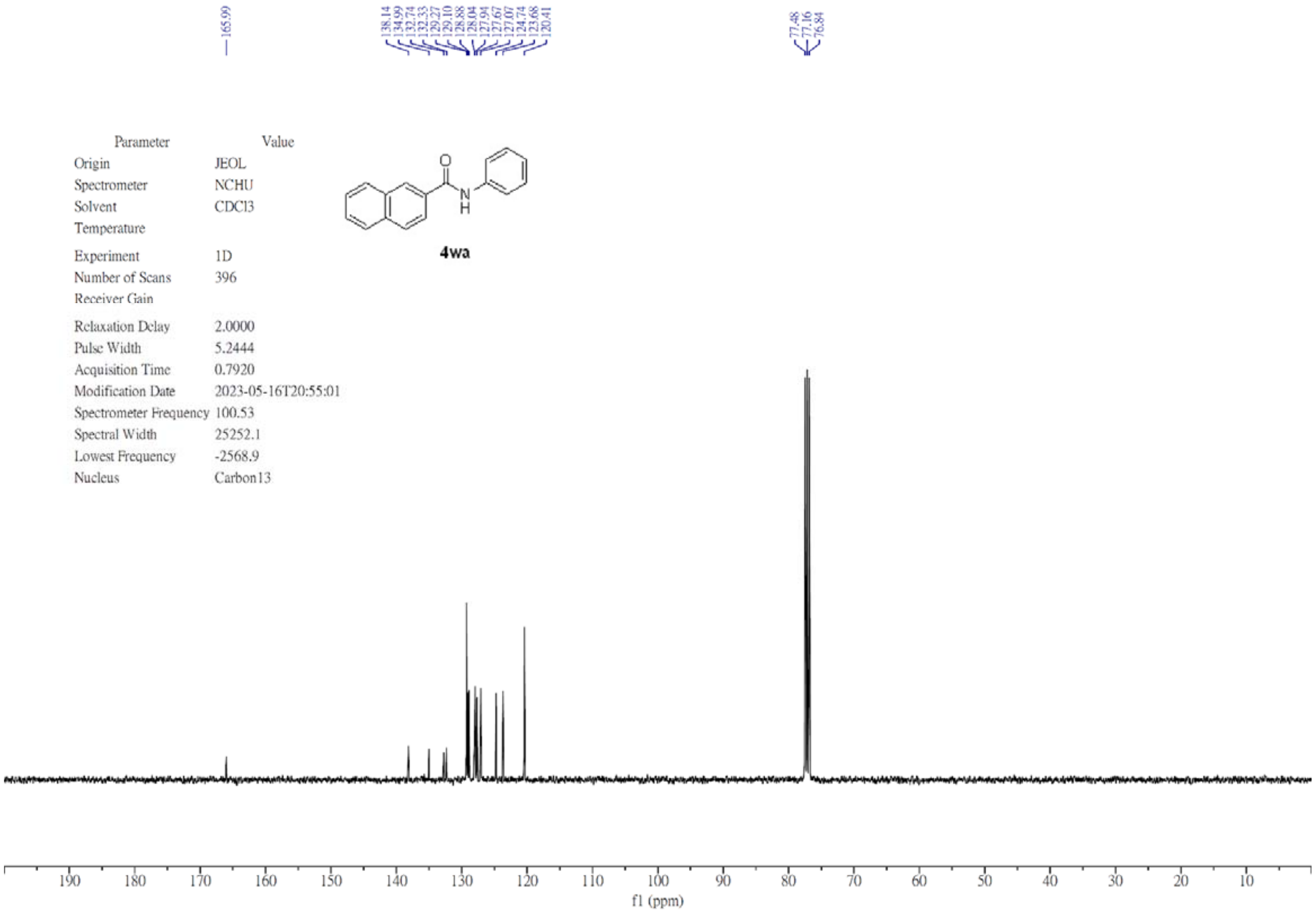

4wa $^{13}C\{^{1}H\}$ NMR spectrum (100 MHz in $CDCl_3$)

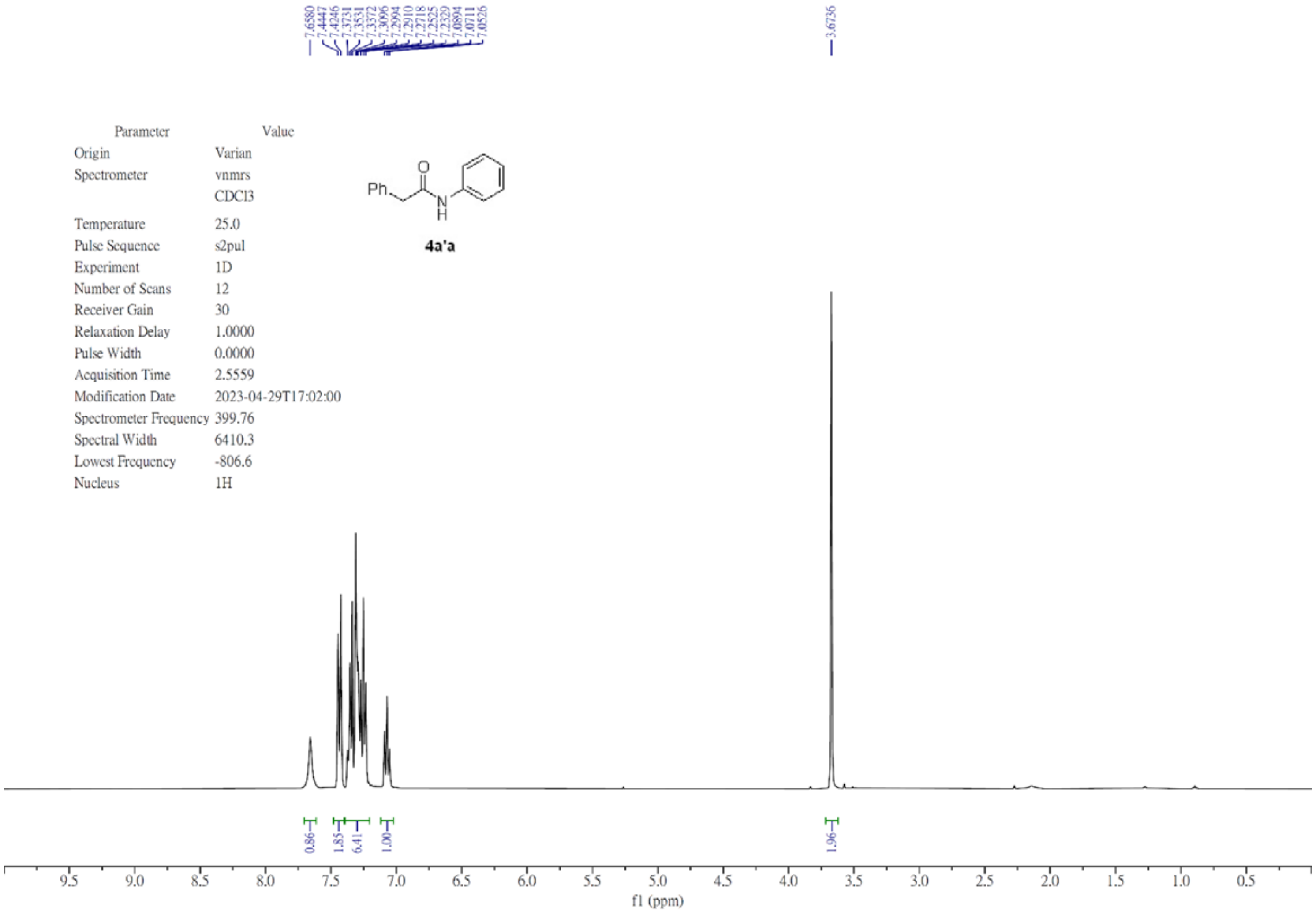

4a'a ^{1}H NMR spectrum (400 MHz in $CDCl_3$)

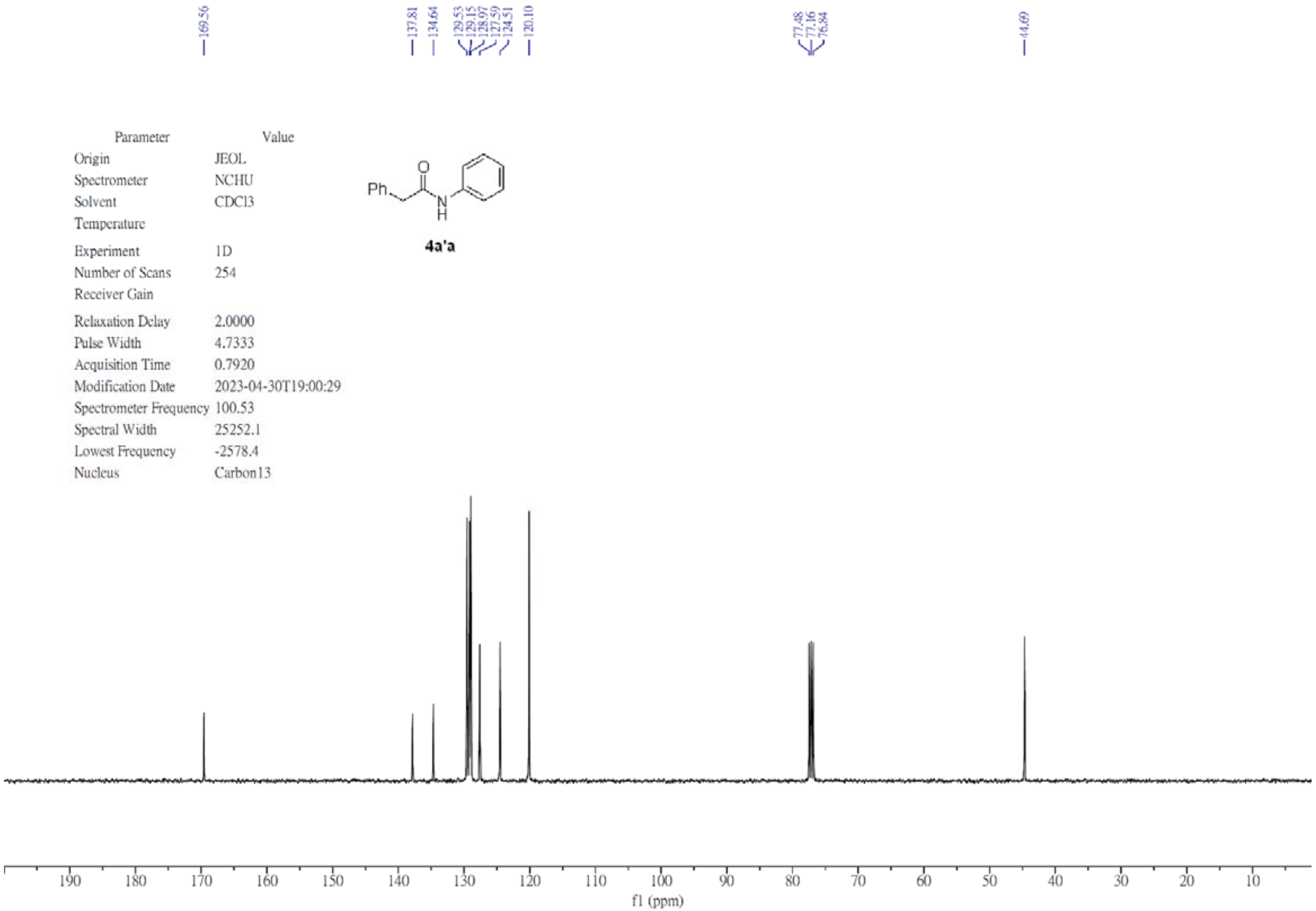

4a'a $^{13}C\{^1H\}$ NMR spectrum (100 MHz in $CDCl_3$)

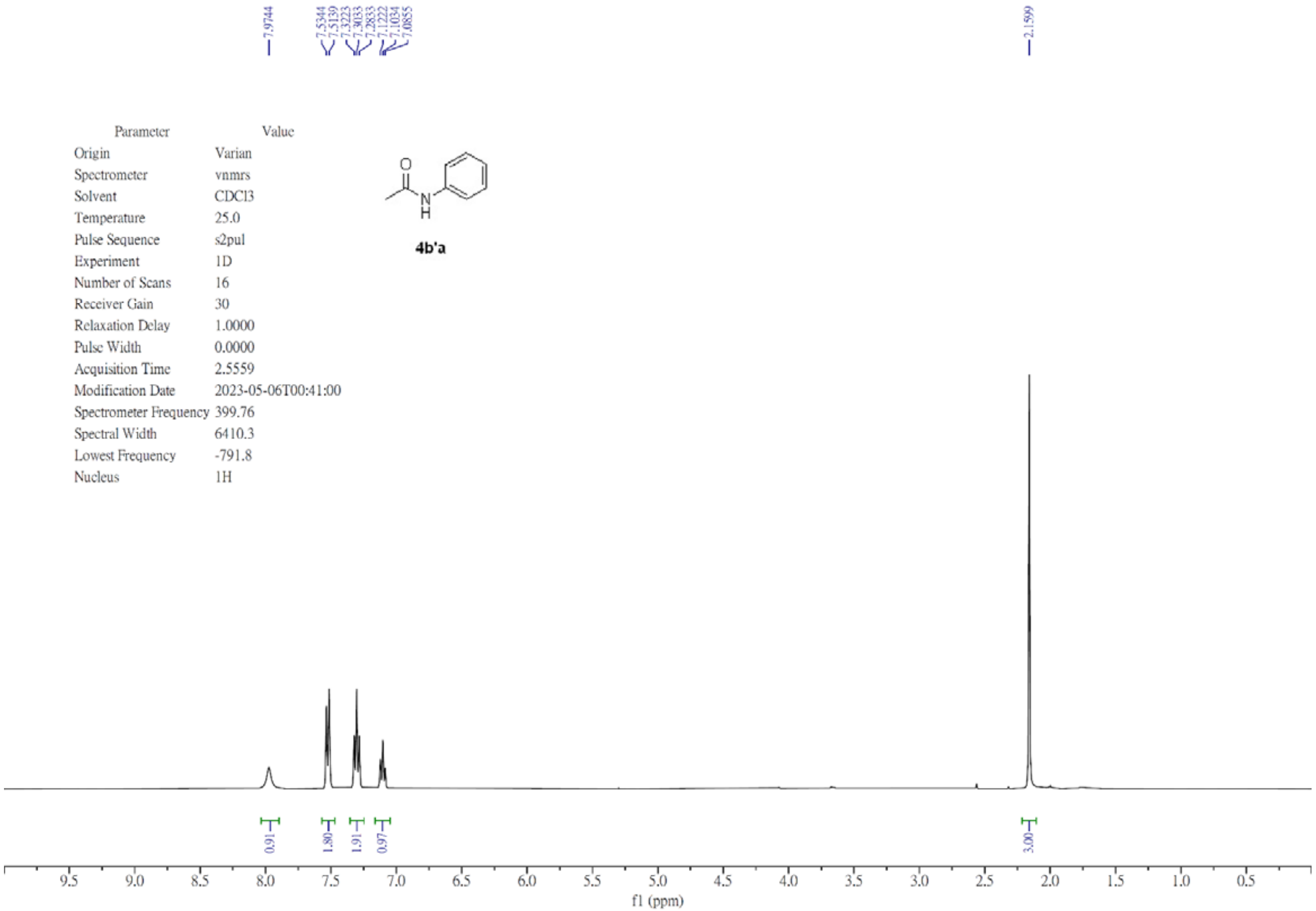

4b'a ^{1}H NMR spectrum (400 MHz in $CDCl_3$)

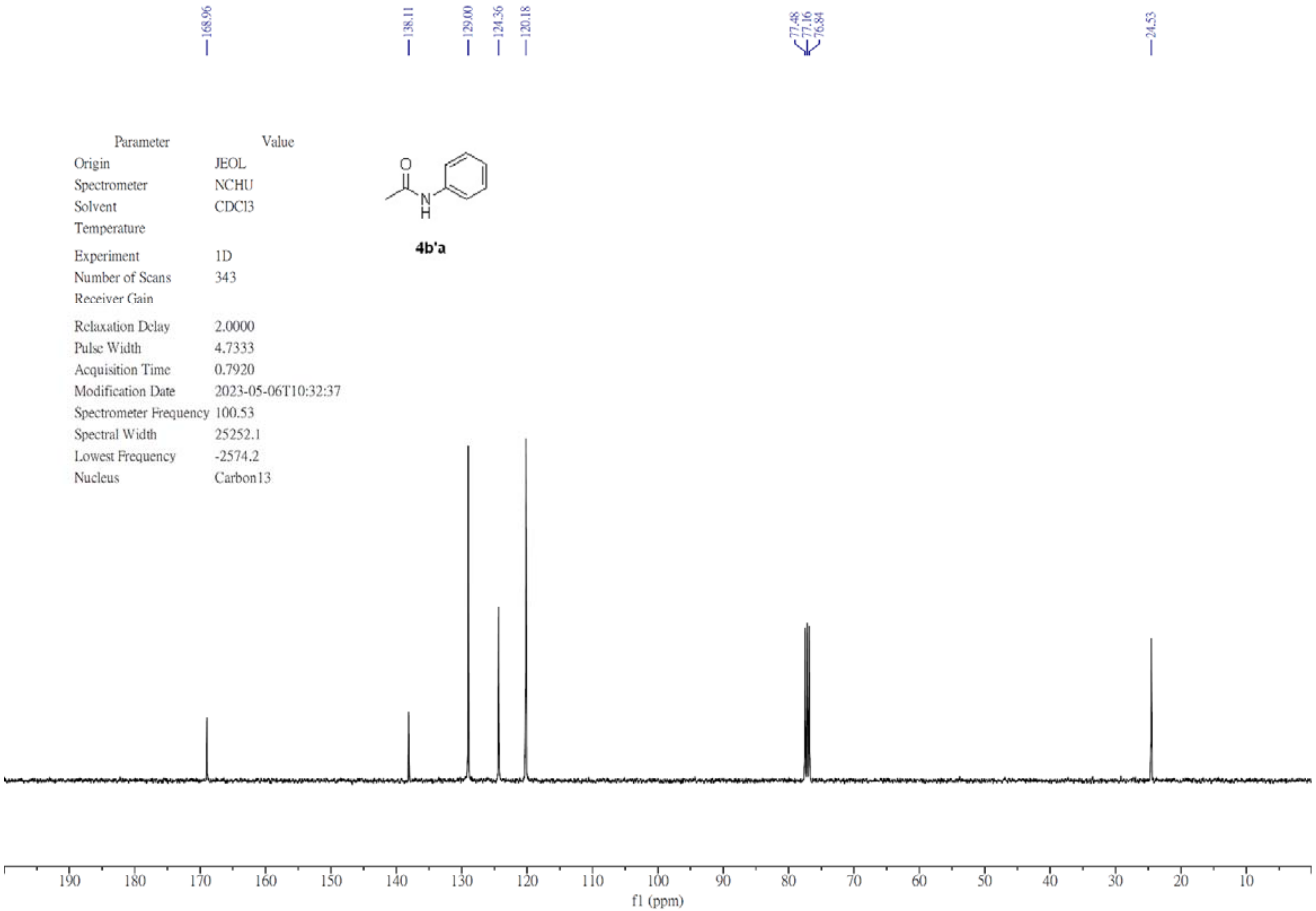

4b'a $^{13}C\{^{1}H\}$ NMR spectrum (100 MHz in $CDCl_3$)

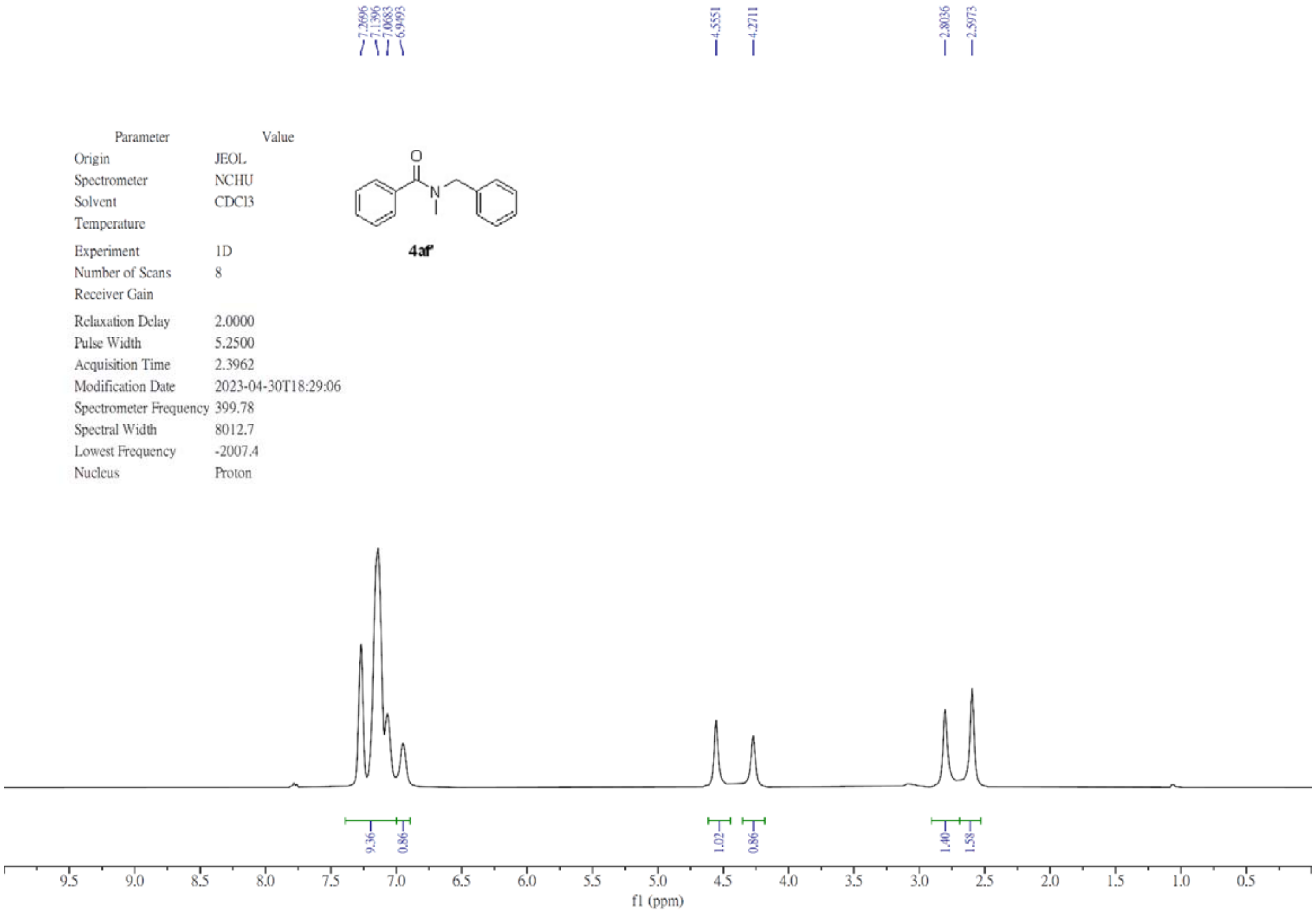

4af' ^{1}H NMR spectrum (400 MHz in $CDCl_3$)

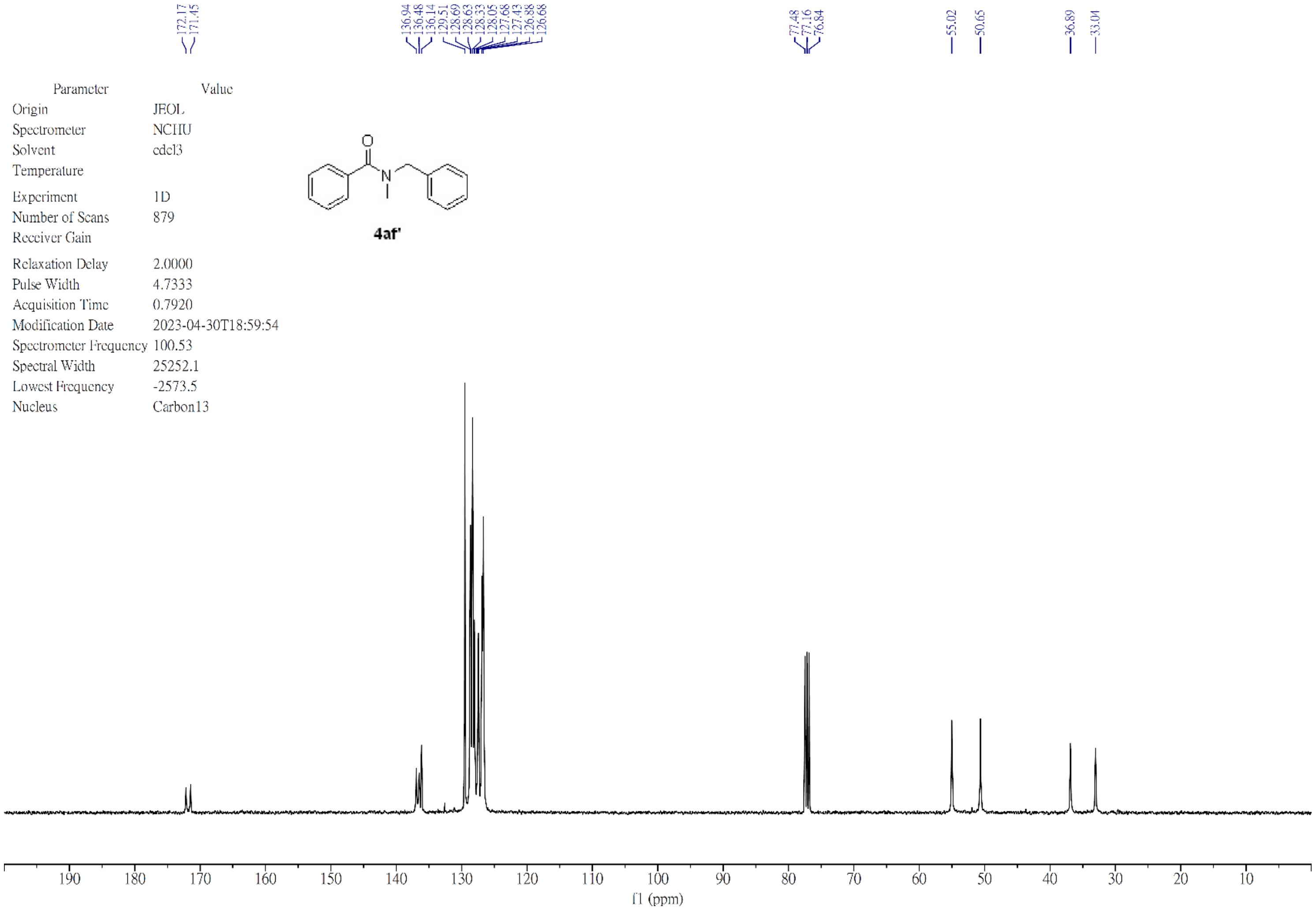

4af' $^{13}C\{^{1}H\}$ NMR spectrum (100 MHz in $CDCl_3$)

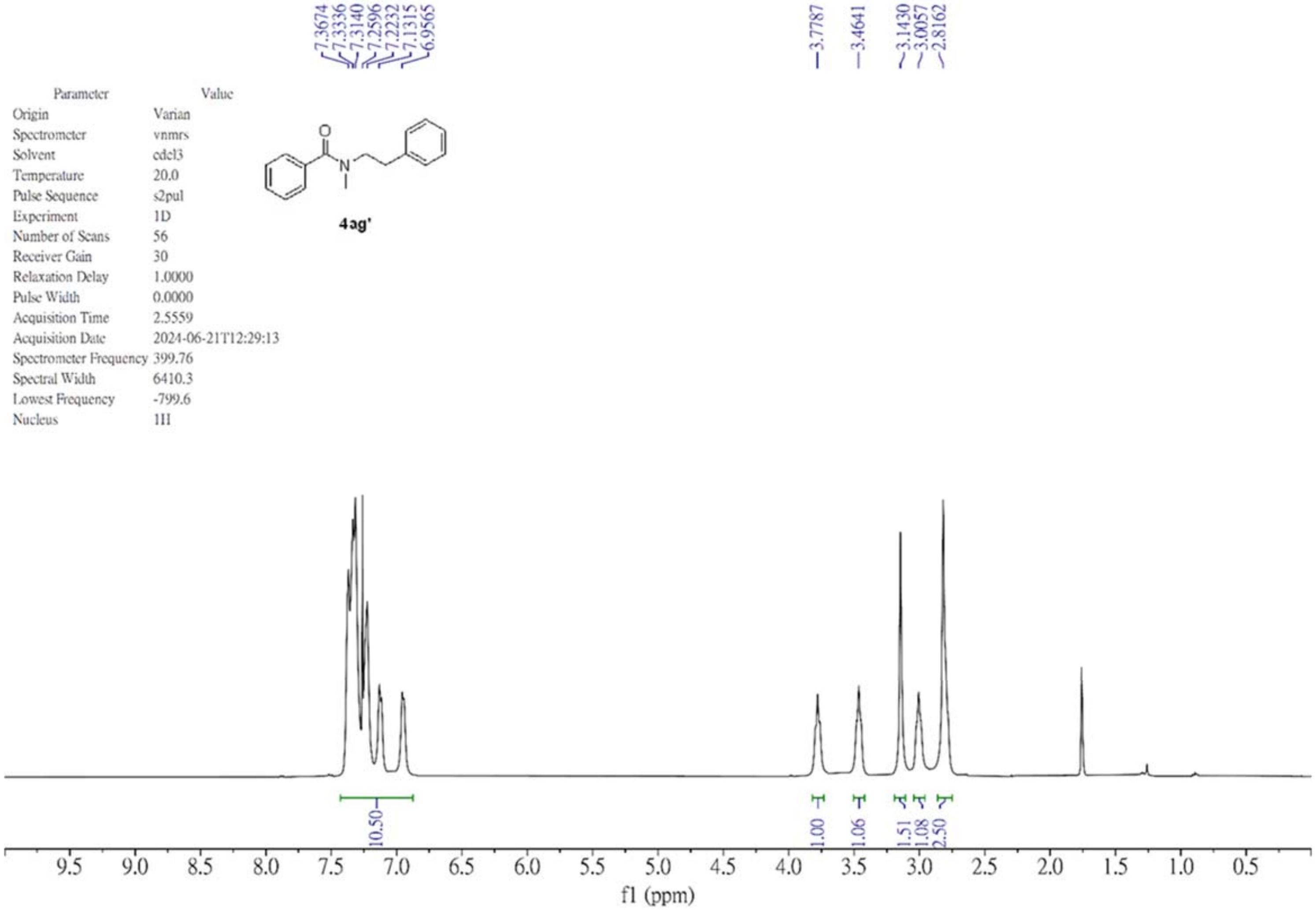

4ag' ^{1}H NMR spectrum (400 MHz in $CDCl_3$)

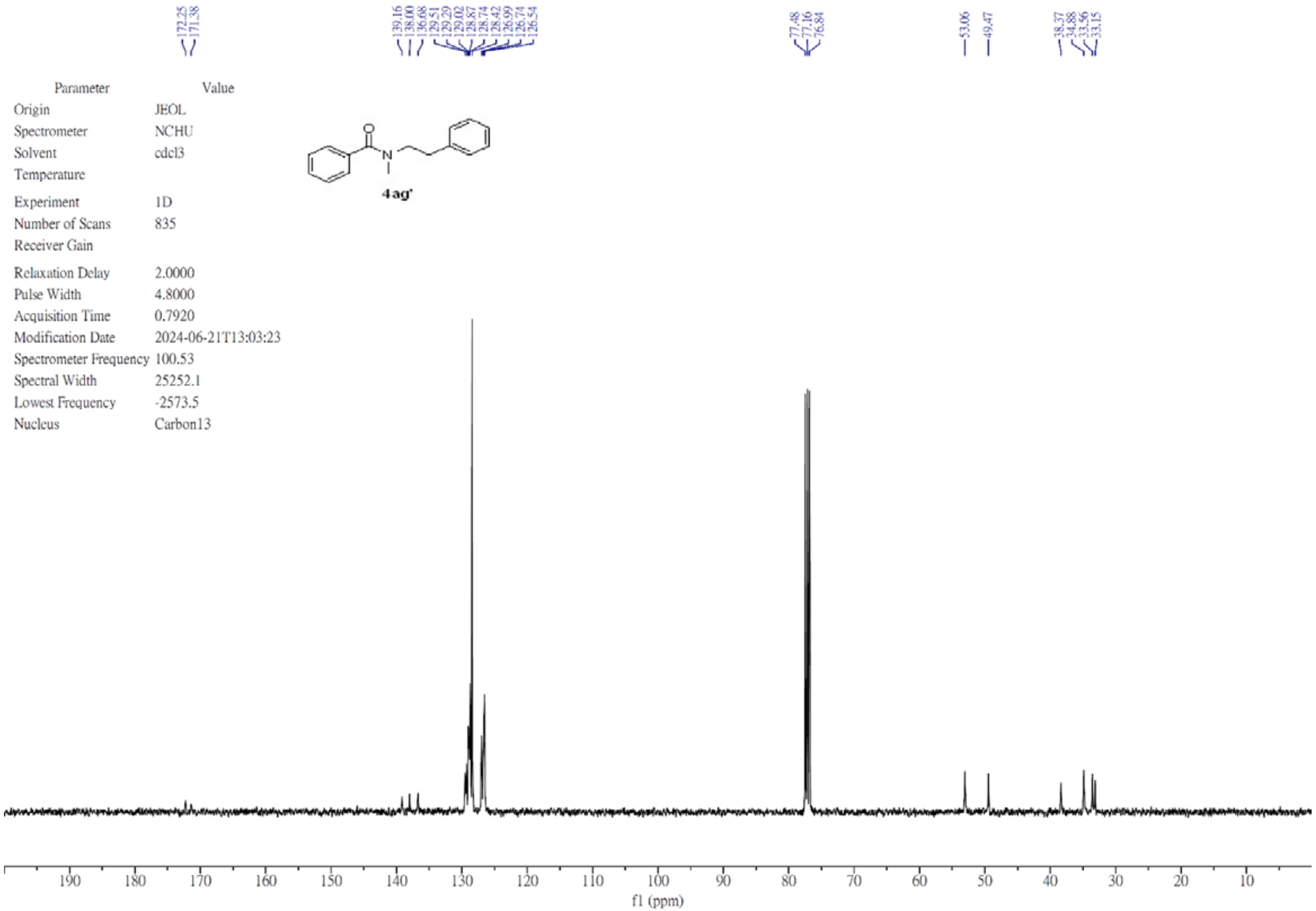

4ag' $^{13}C\{^{1}H\}$ NMR spectrum (100 MHz in $CDCl_3$)

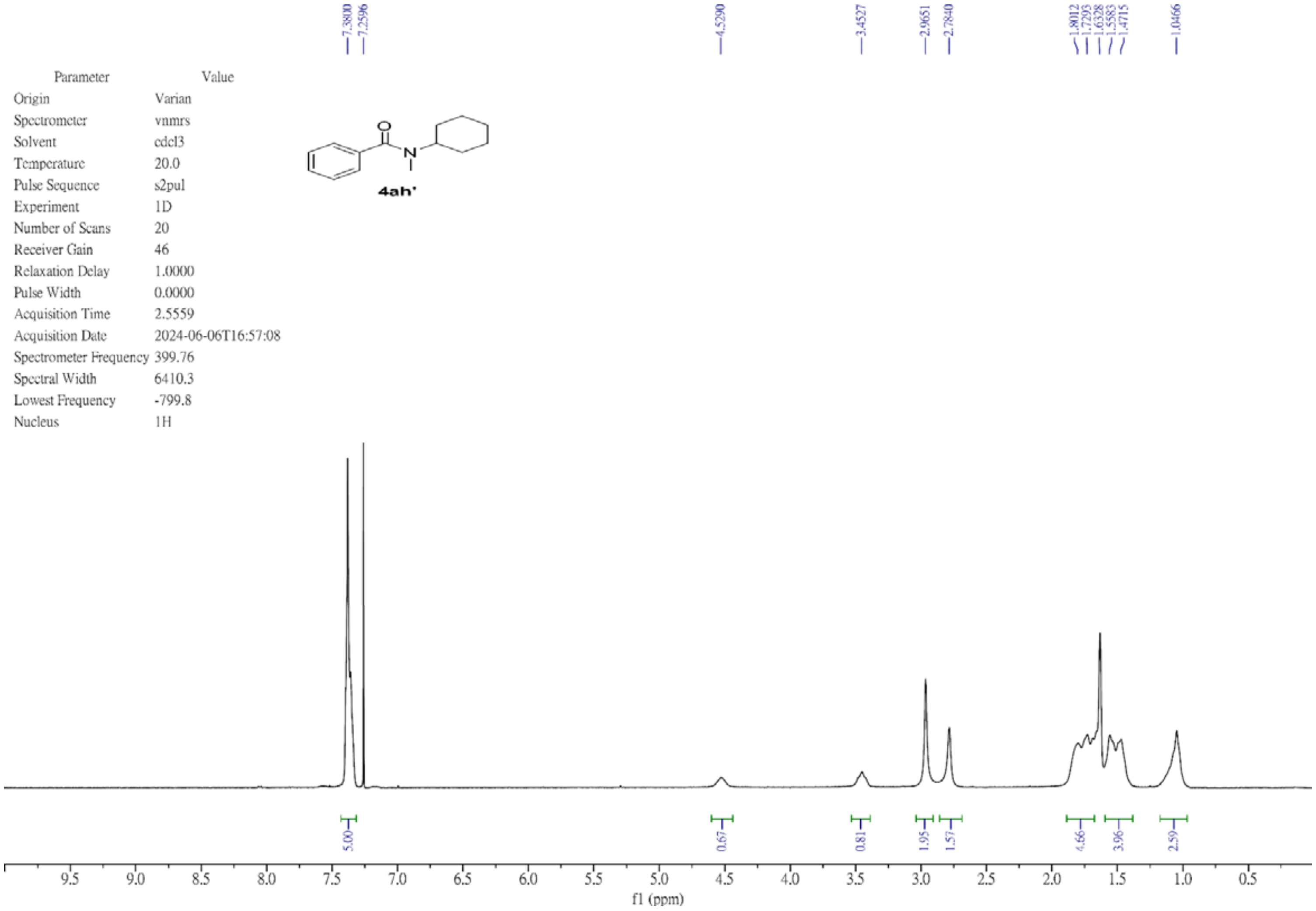

Parameter	Value
Origin	Varian
Spectrometer	vnmrs
Solvent	cdcl3
Temperature	20.0
Pulse Sequence	s2pul
Experiment	1D
Number of Scans	20
Receiver Gain	46
Relaxation Delay	1.0000
Pulse Width	0.0000
Acquisition Time	2.5559
Acquisition Date	2024-06-06T16:57:08
Spectrometer Frequency	399.76
Spectral Width	6410.3
Lowest Frequency	-799.8
Nucleus	1H

4ah' ^{1}H NMR spectrum (400 MHz in $CDCl_3$)

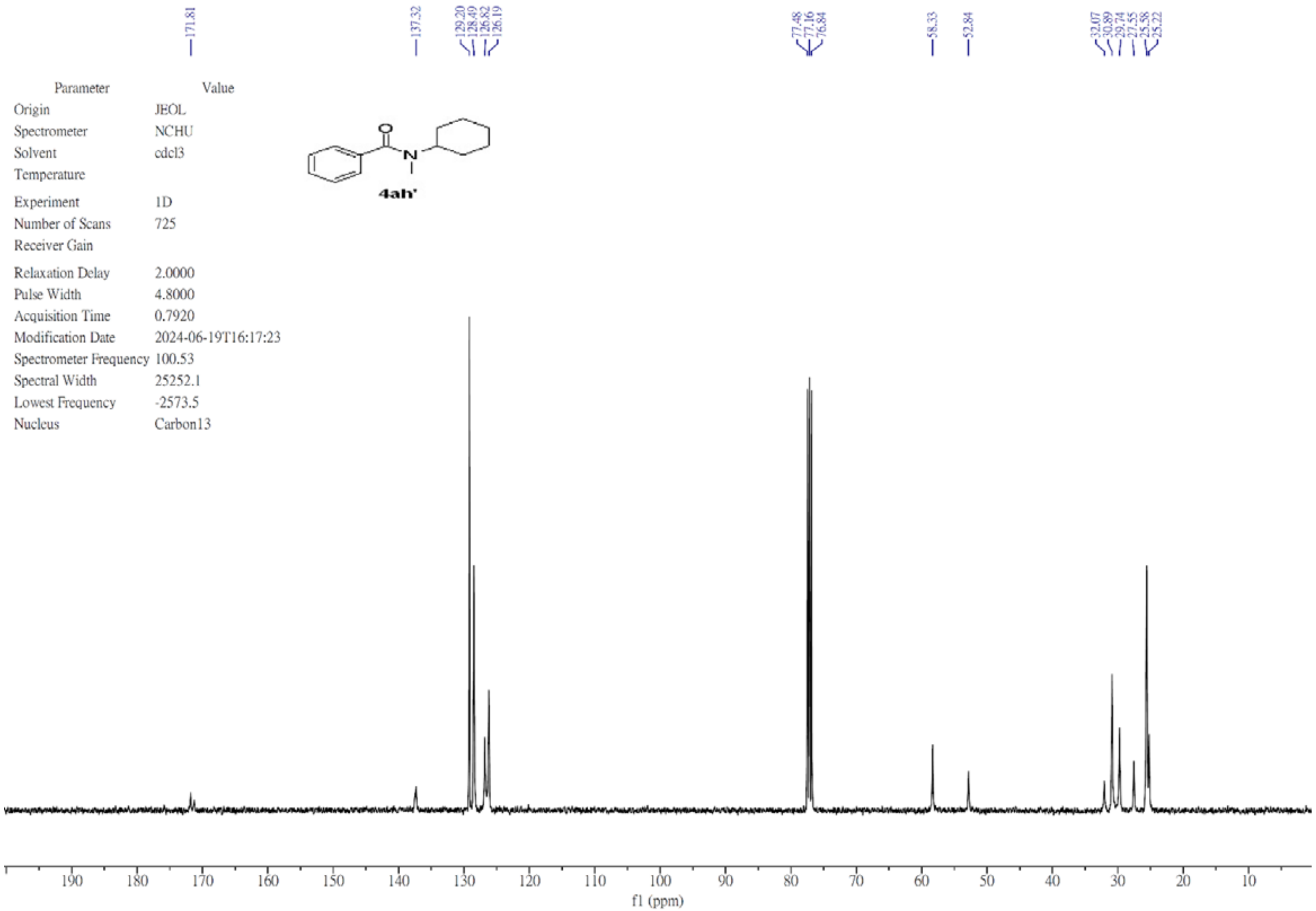

4ah' $^{13}C\{^{1}H\}$ NMR spectrum (100 MHz in $CDCl_3$)

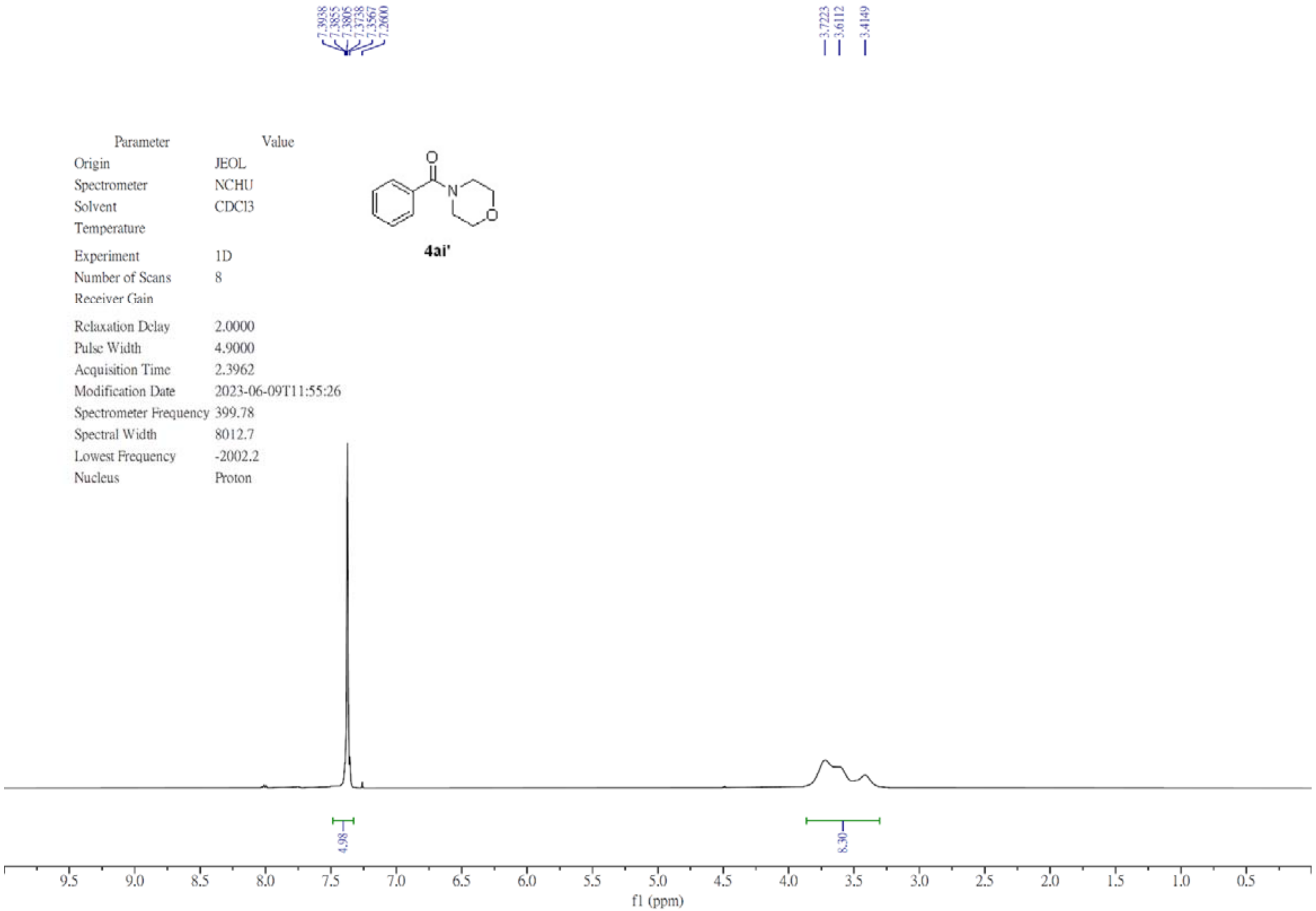

4ai' ^{1}H NMR spectrum (400 MHz in $CDCl_3$)

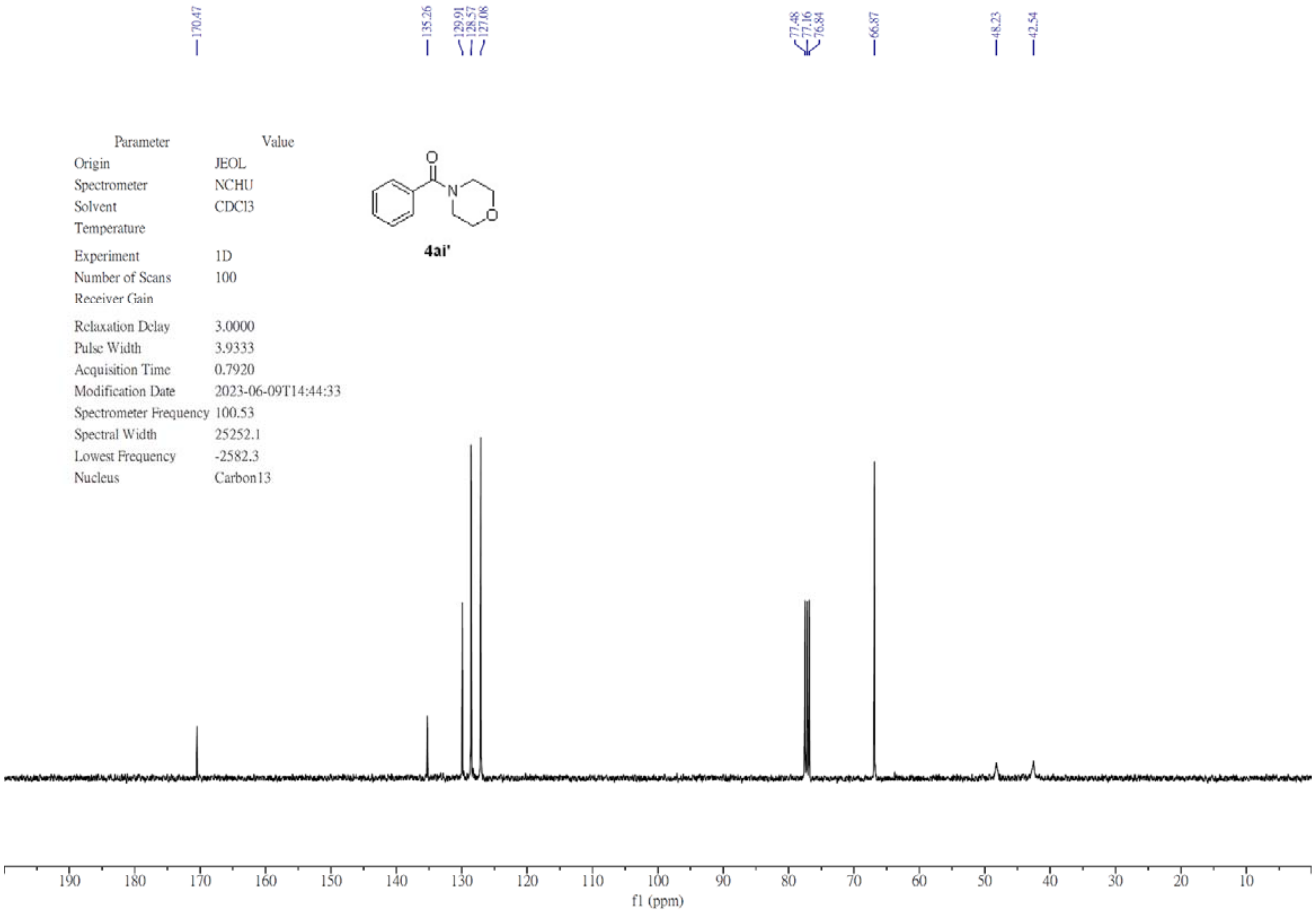

4ai' $^{13}C\{^{1}H\}$ NMR spectrum (100 MHz in $CDCl_3$)

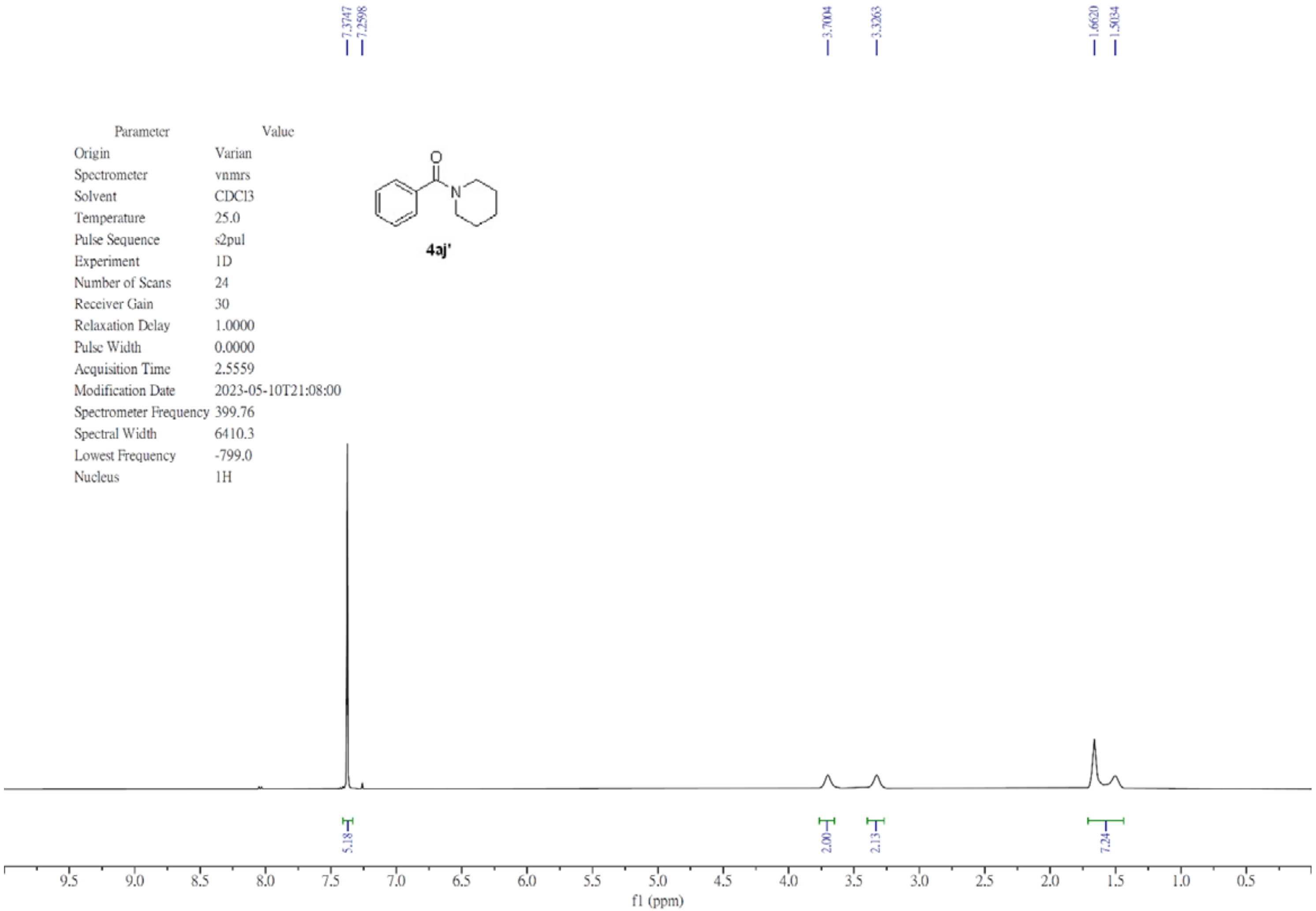

4aj' ^{1}H NMR spectrum (400 MHz in $CDCl_3$)

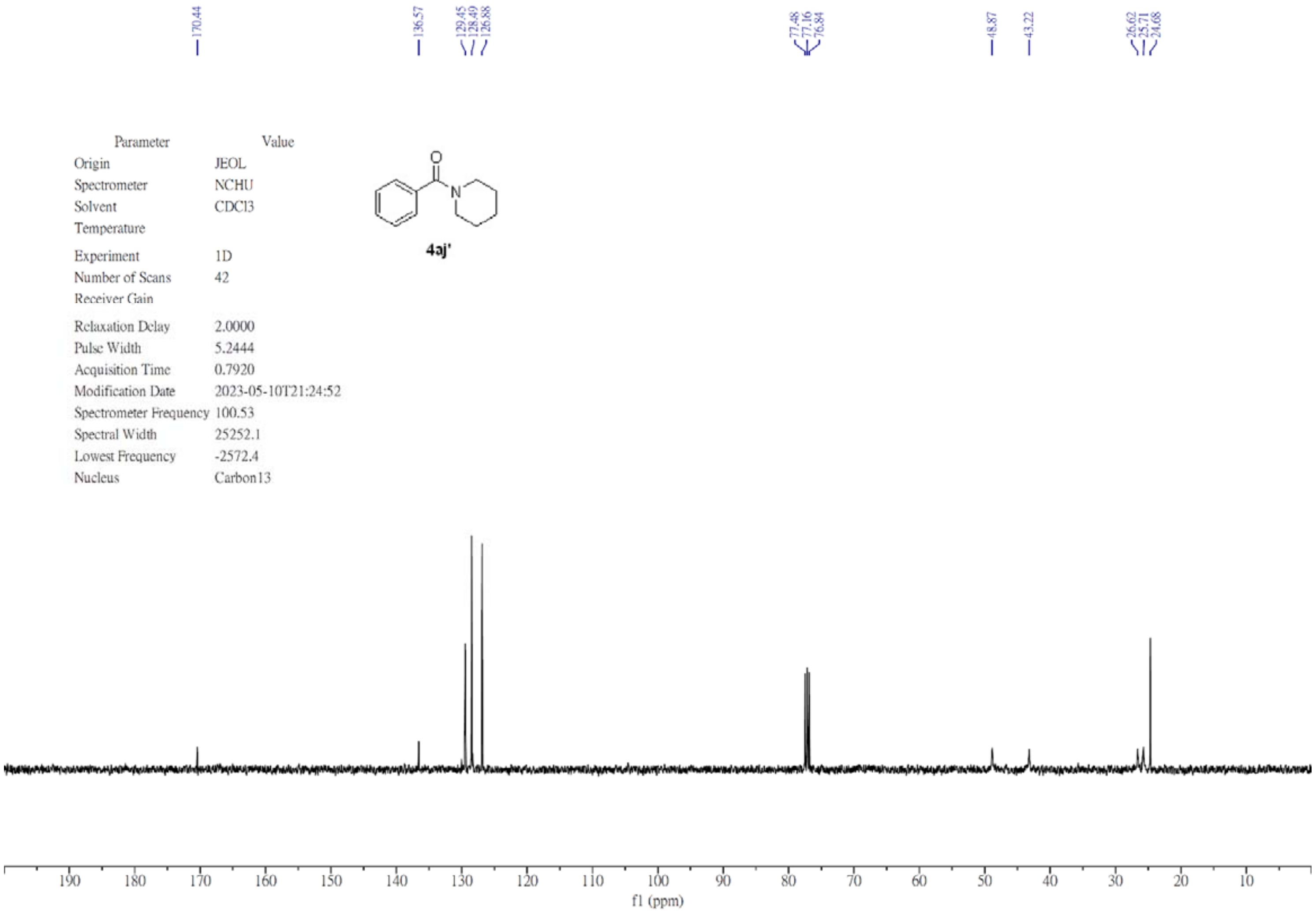

4aj' $^{13}C\{^{1}H\}$ NMR spectrum (100 MHz in $CDCl_3$)

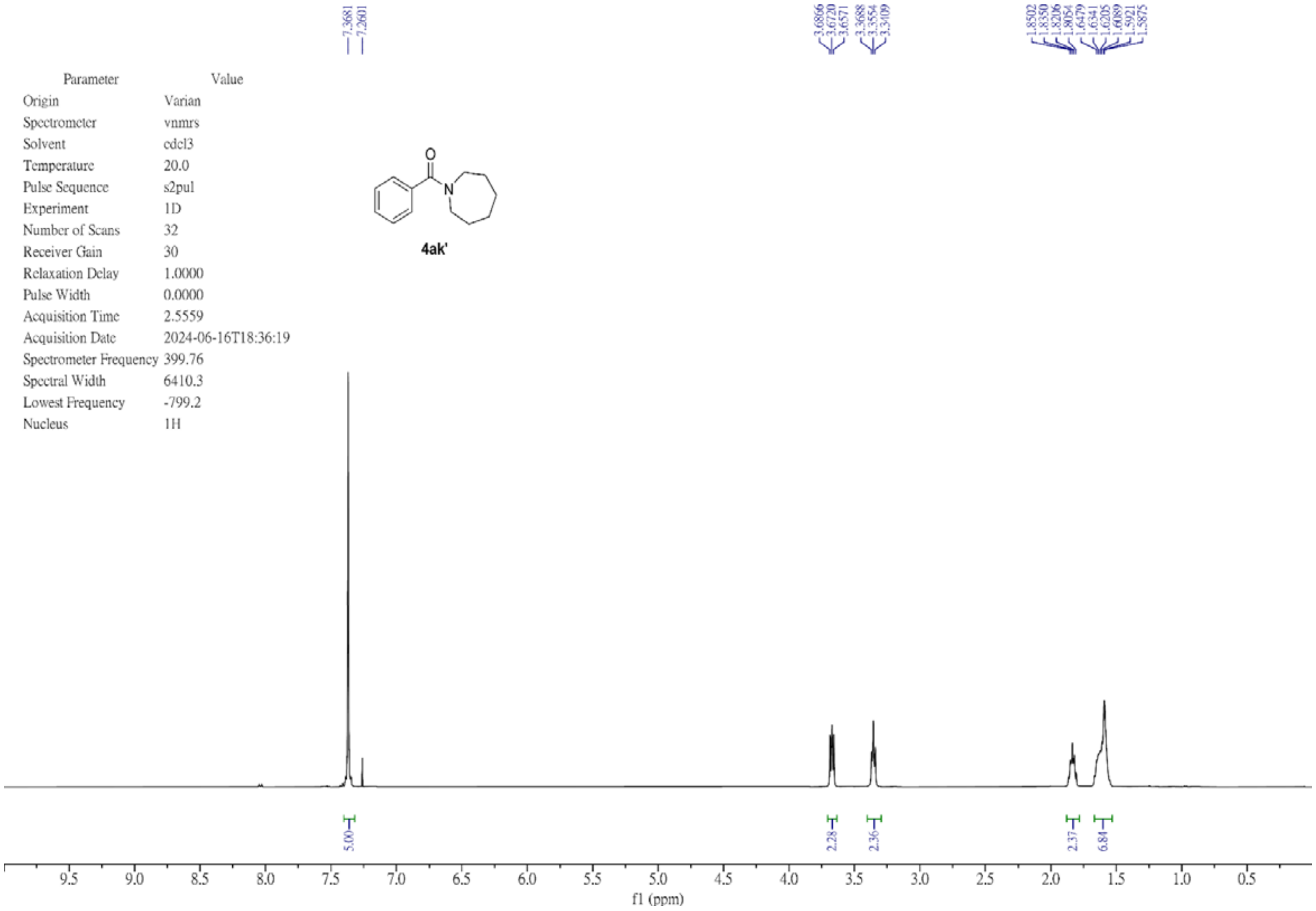

4ak' ^{1}H NMR spectrum (400 MHz in $CDCl_3$)

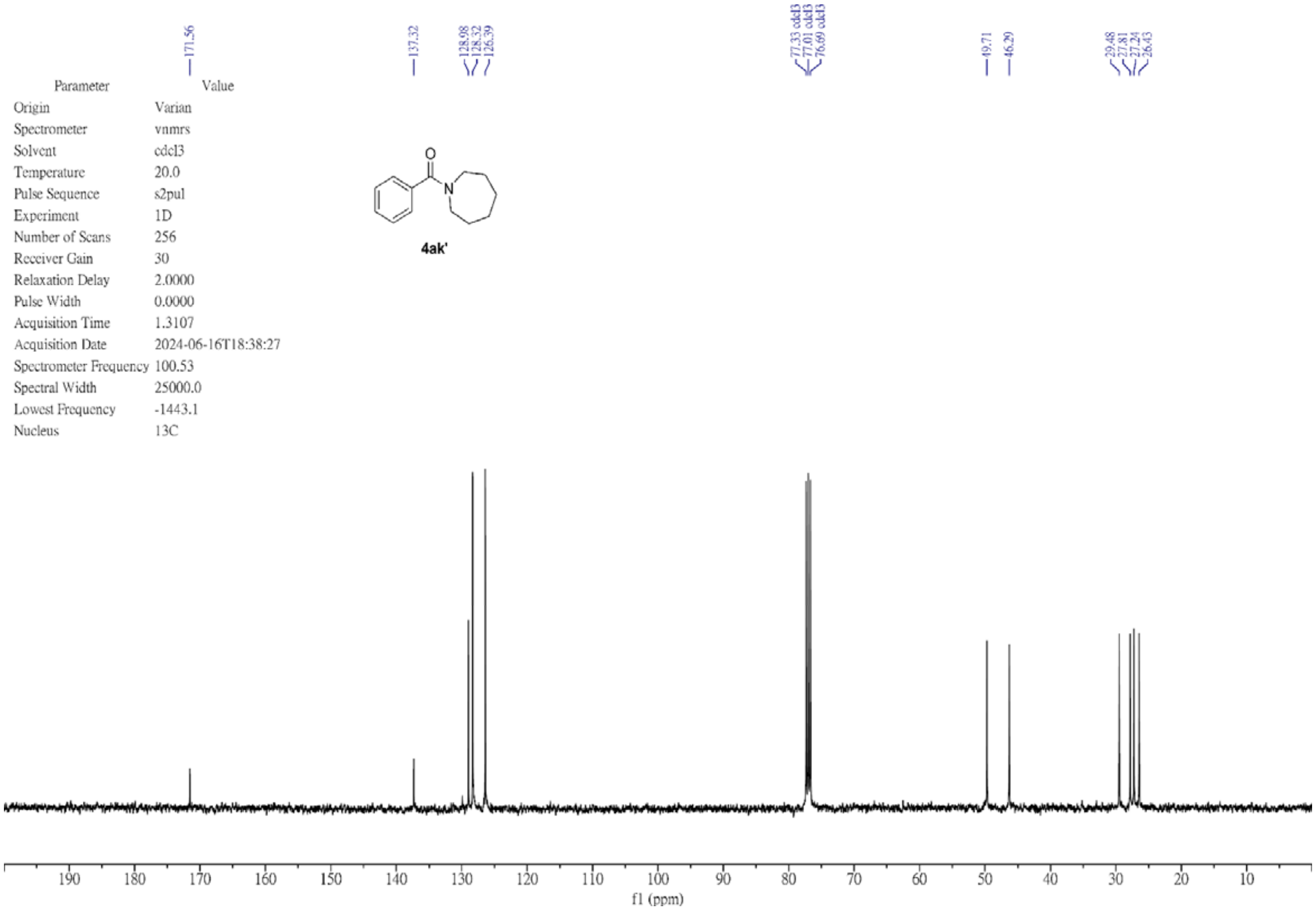

4ak' $^{13}C\{^{1}H\}$ NMR spectrum (100 MHz in $CDCl_3$)

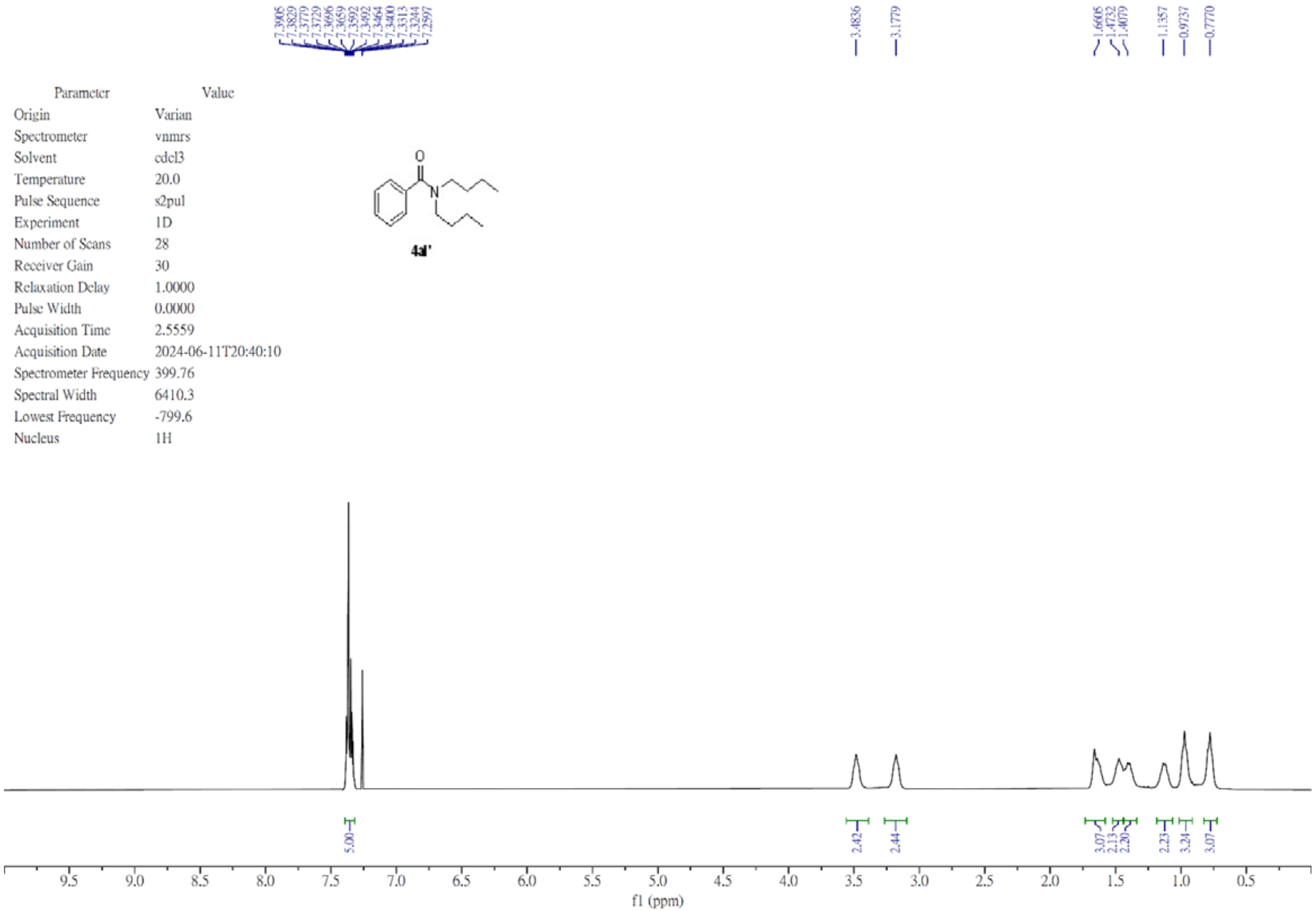

4al' ^{1}H NMR spectrum (400 MHz in $CDCl_3$)

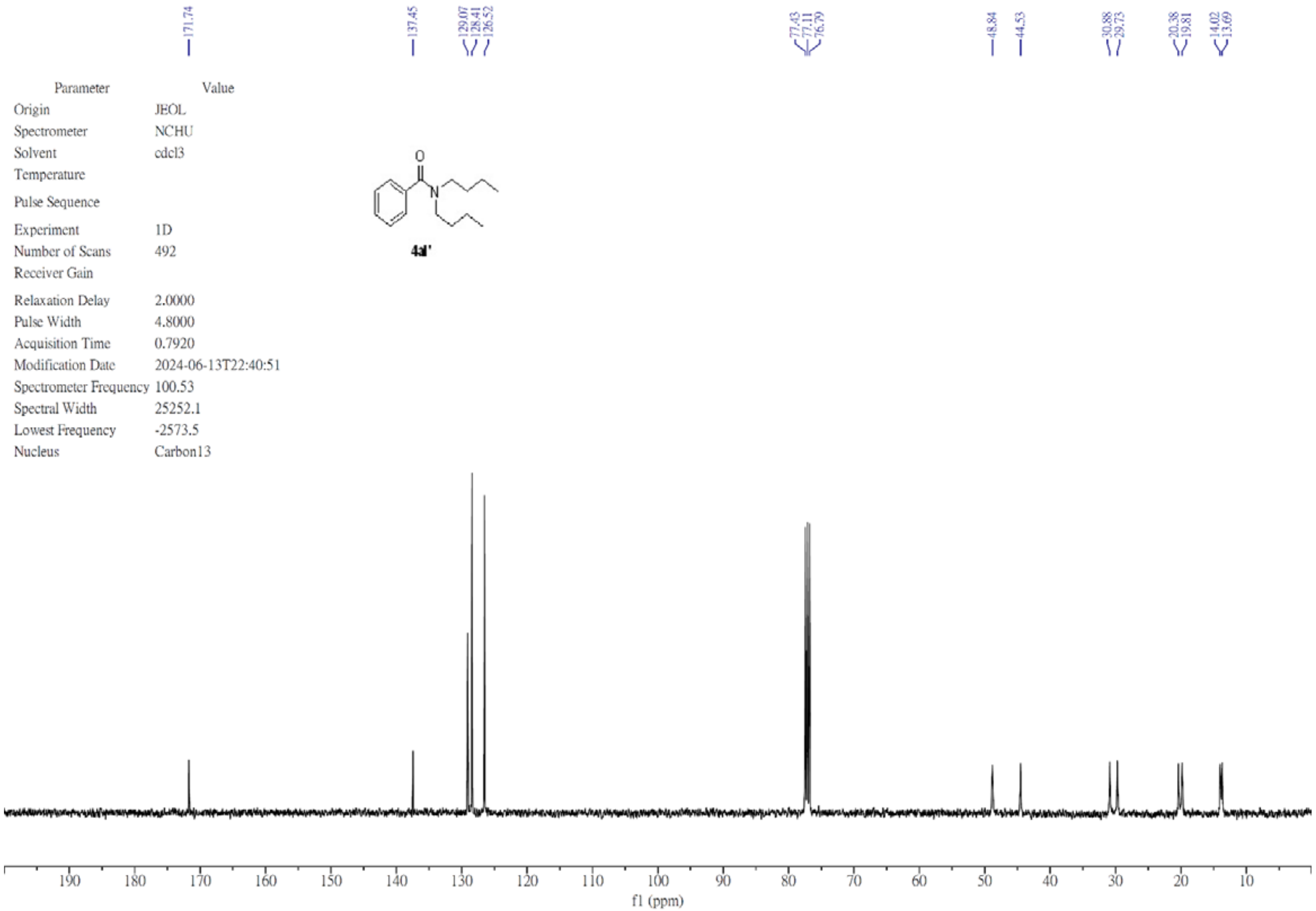

4al' $^{13}C\{^{1}H\}$ NMR spectrum (100 MHz in $CDCl_3$)

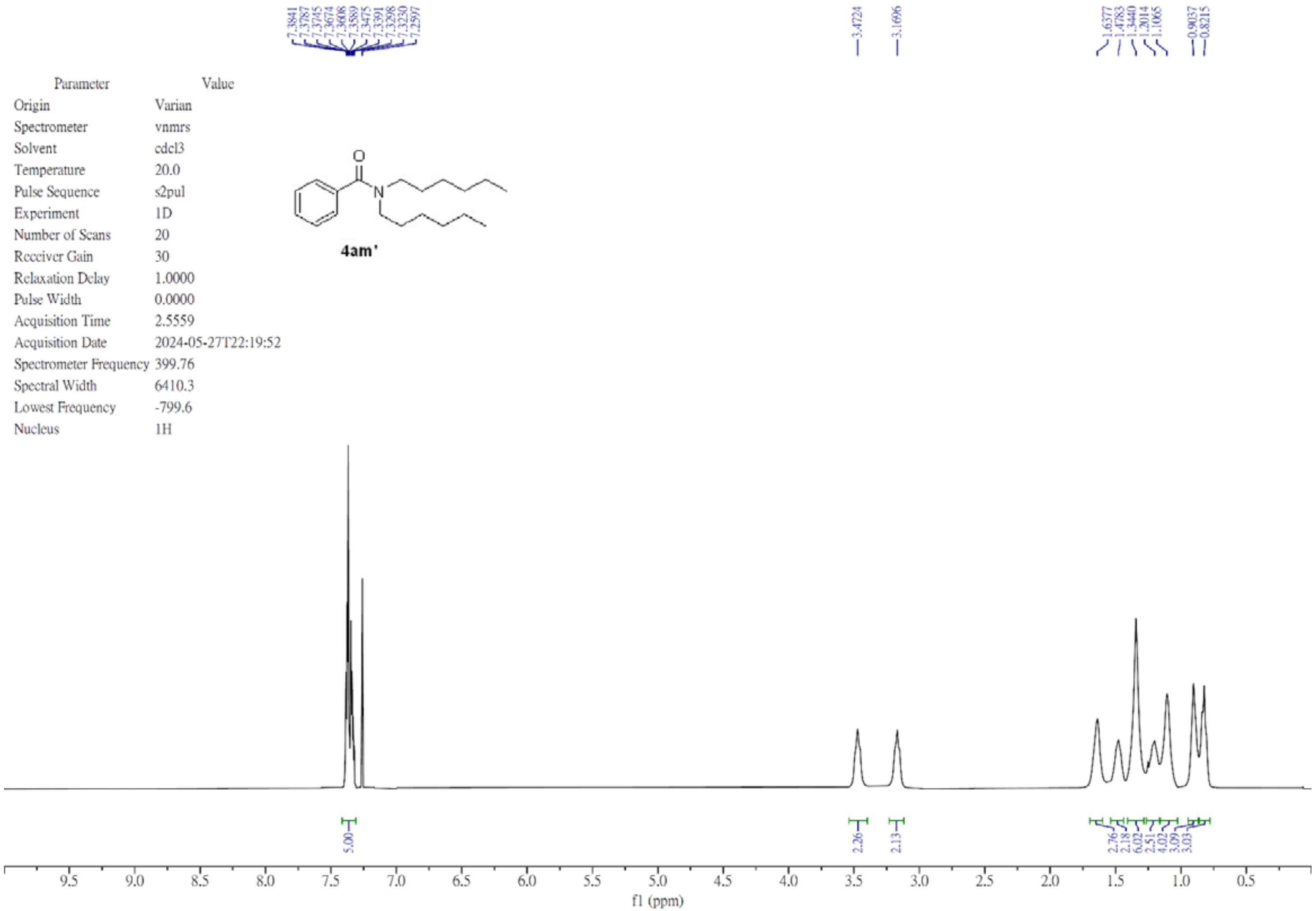

4am' ^{1}H NMR spectrum (400 MHz in $CDCl_3$)

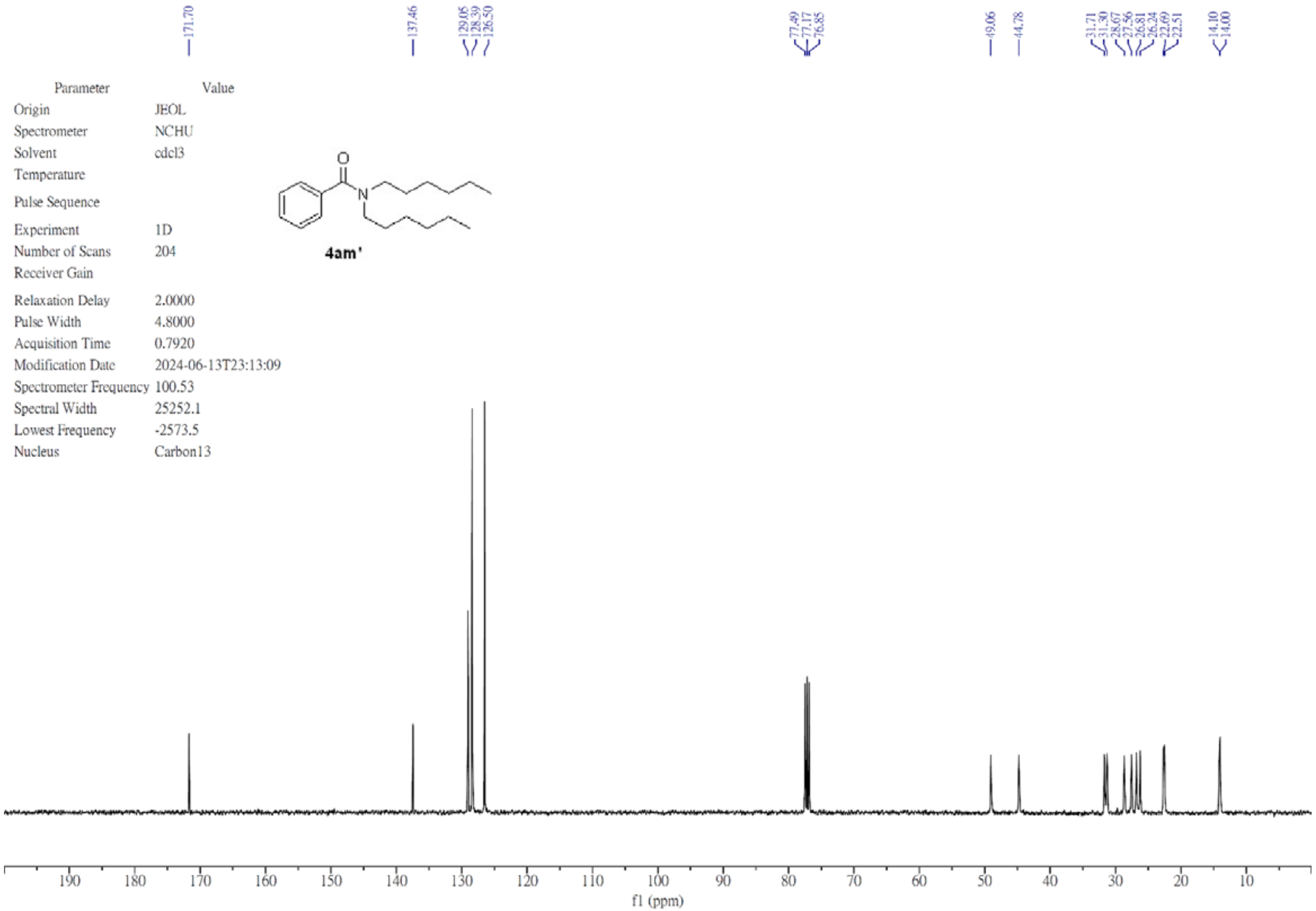

4am' $^{13}C\{^{1}H\}$ NMR spectrum (100 MHz in $CDCl_3$)

Parameter	Value
Origin	Varian
Spectrometer	vnmrs
Solvent	cdcl3
Temperature	20.0
Pulse Sequence	s2pul
Experiment	1D
Number of Scans	8
Receiver Gain	30
Relaxation Delay	1.0000
Pulse Width	0.0000
Acquisition Time	2.5559
Modification Date	2025-06-24T15:10:00
Spectrometer Frequency	399.76
Spectral Width	6410.3
Lowest Frequency	-799.4
Nucleus	1H

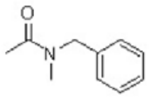

4b'f'

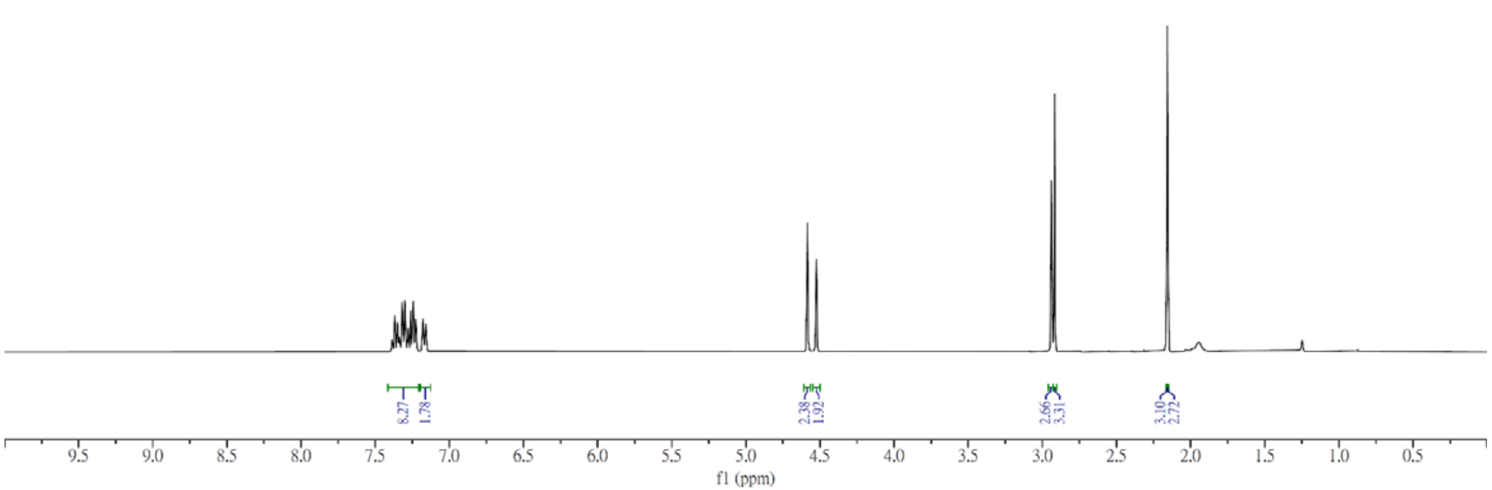

4b'f' ^{1}H NMR spectrum (400 MHz in $CDCl_3$)

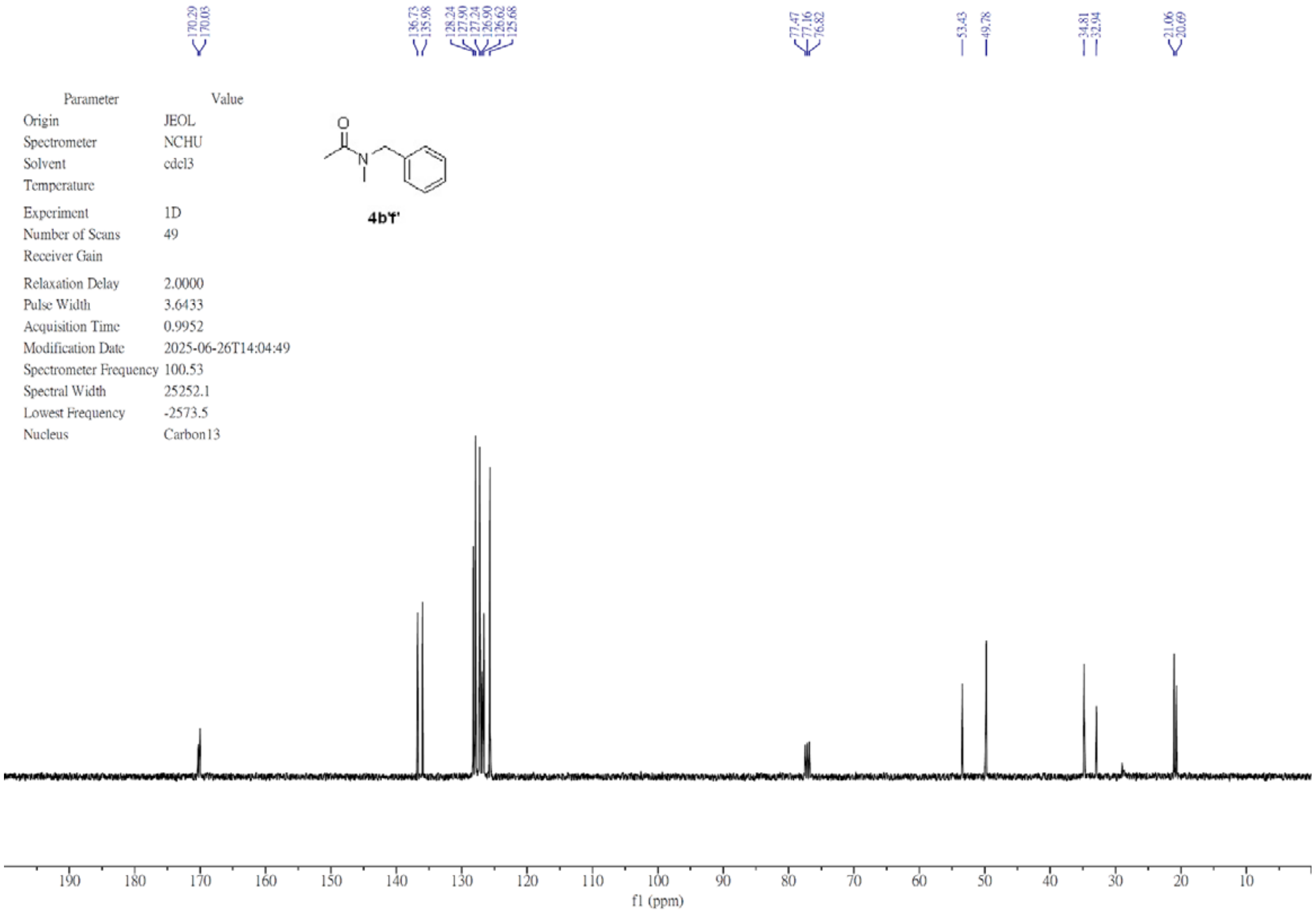

4b'f' $^{13}C\{^{1}H\}$ NMR spectrum (100 MHz in $CDCl_3$)

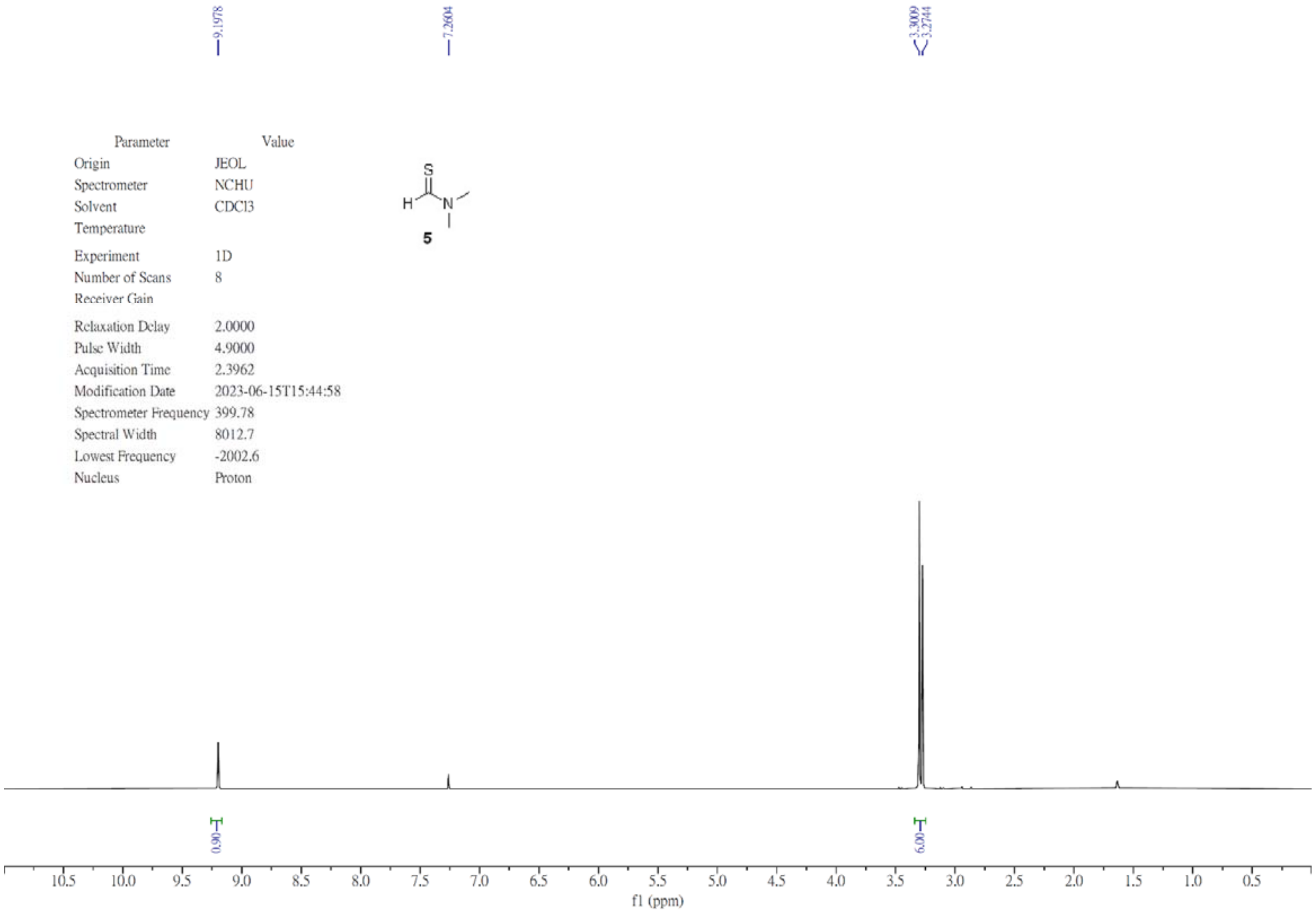

5 ^{1}H NMR spectrum (400 MHz in $CDCl_3$)

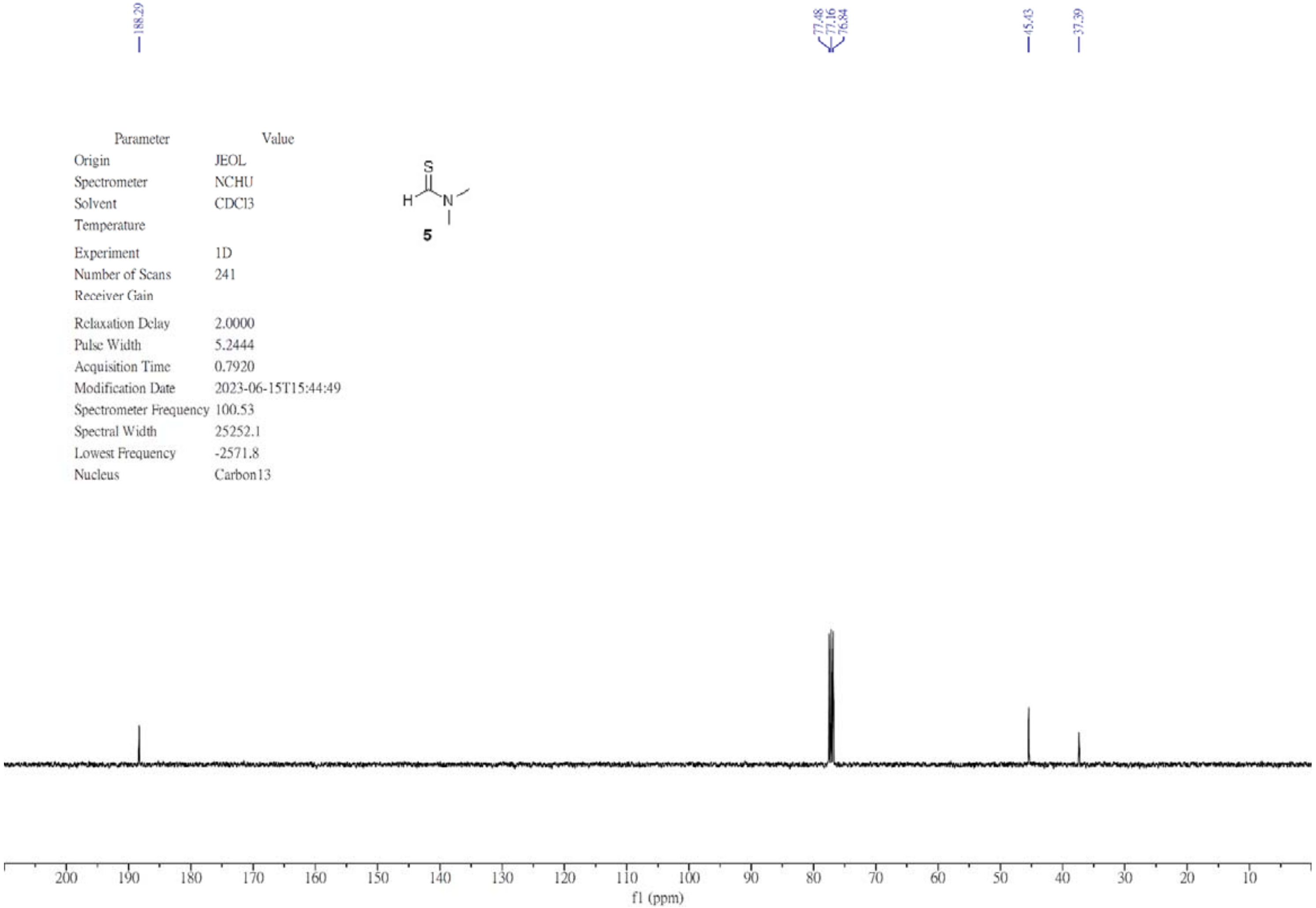

5 $^{13}C\{^{1}H\}$ NMR spectrum (100 MHz in $CDCl_3$)

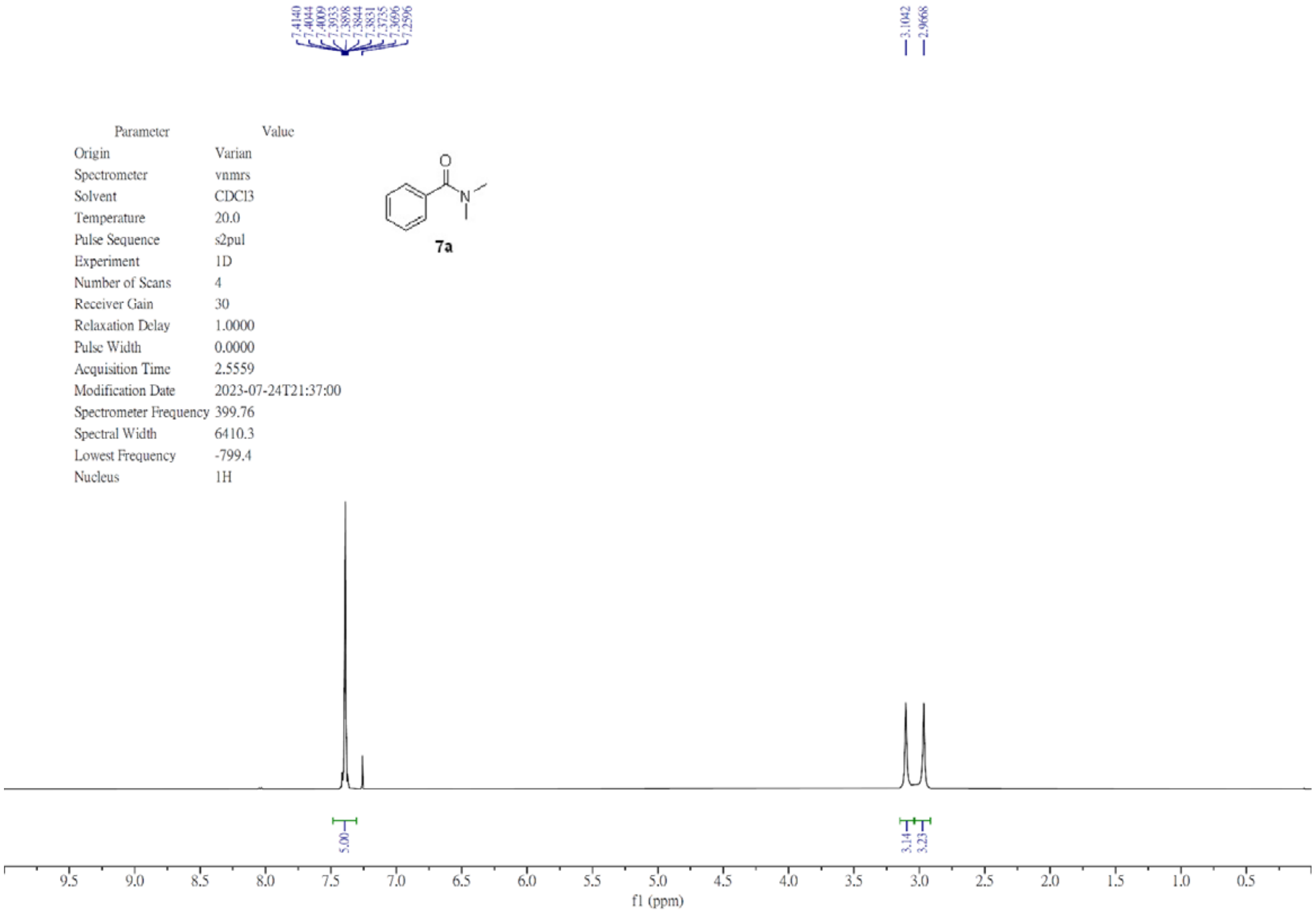

7a ^{1}H NMR spectrum (400 MHz in $CDCl_3$)

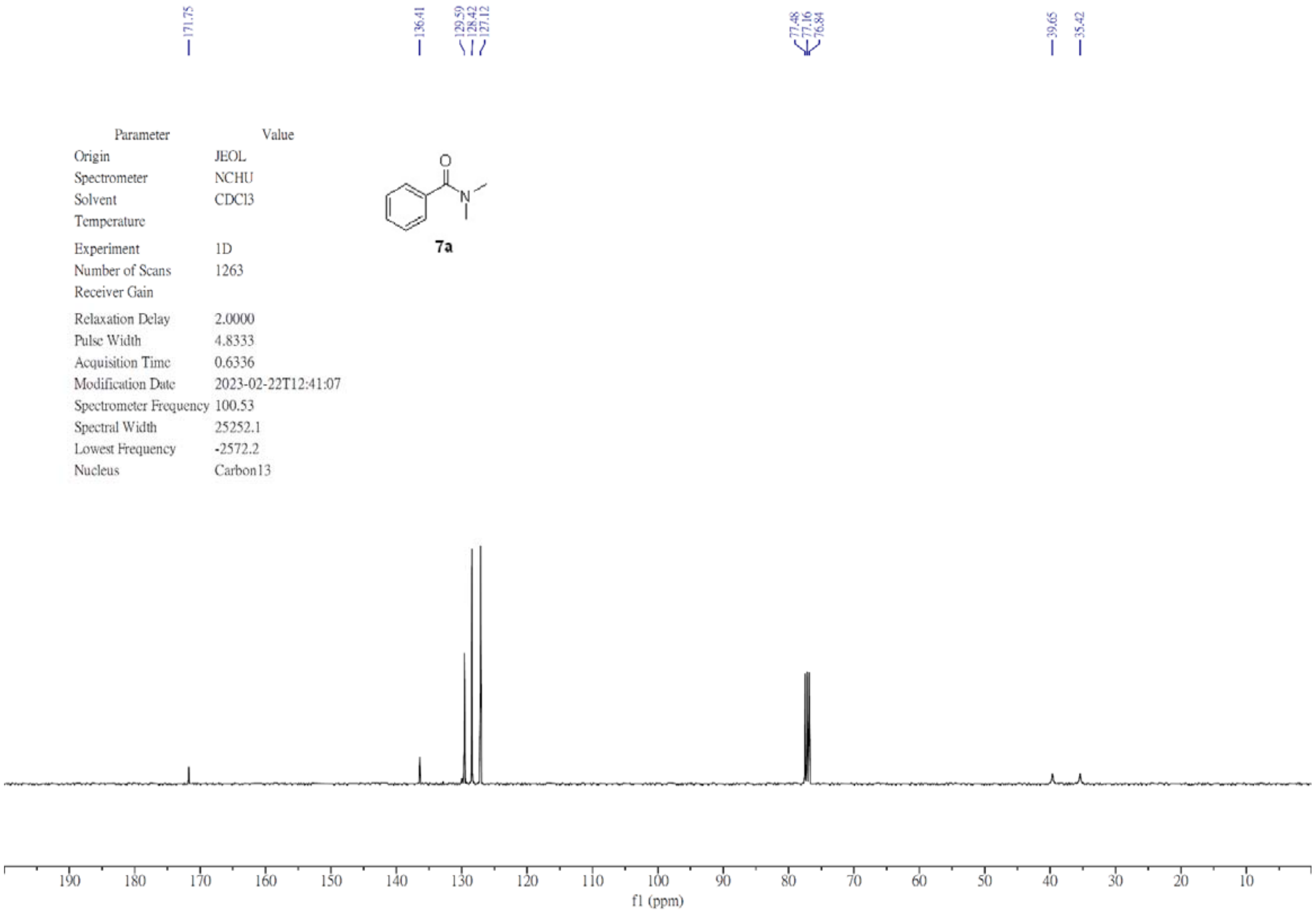

7a

7a $^{13}C\{^1H\}$ NMR spectrum (100 MHz in $CDCl_3$)

Parameter	Value
Origin	JEOL
Spectrometer	NCHU
Solvent	CDCl3
Temperature	
Experiment	1D
Number of Scans	8
Receiver Gain	
Relaxation Delay	2.0000
Pulse Width	5.2500
Acquisition Time	2.3962
Modification Date	2023-05-11T00:07:27
Spectrometer Frequency	399.78
Spectral Width	8012.7
Lowest Frequency	-2003.0
Nucleus	Proton

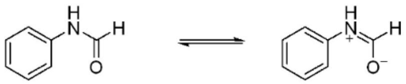

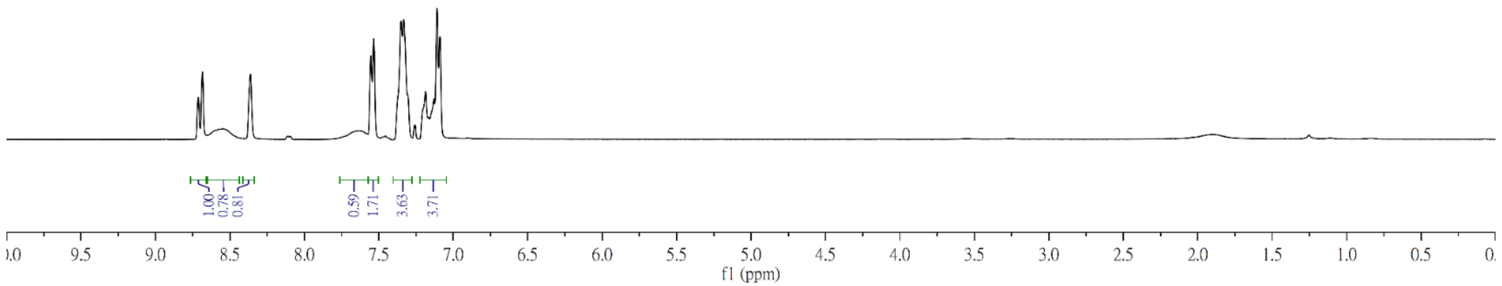

9 ^{1}H NMR spectrum (400 MHz in $CDCl_3$)

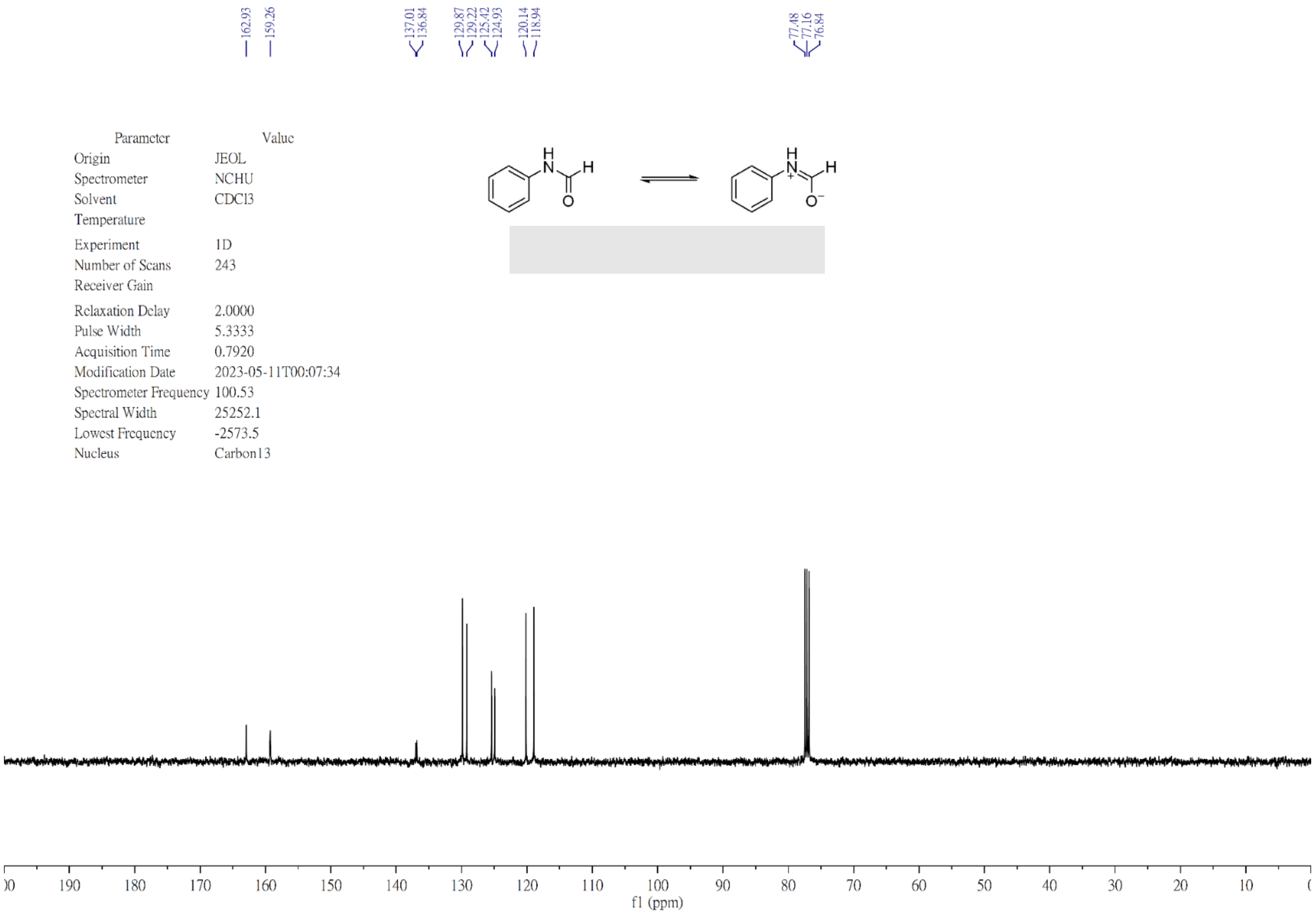

9 $^{13}C\{^{1}H\}$ NMR spectrum (100 MHz in $CDCl_3$)

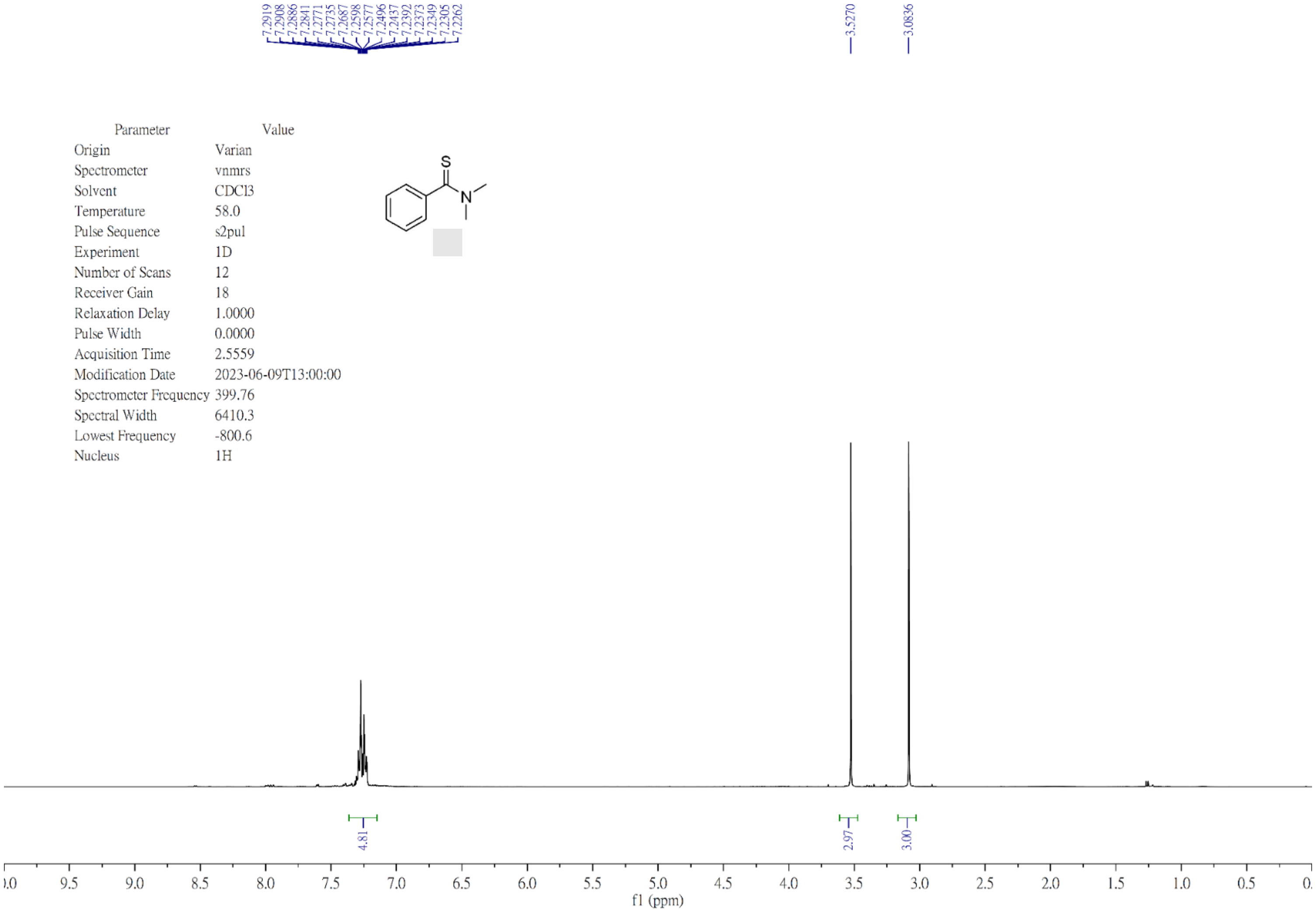

10 ^{1}H NMR spectrum (400 MHz in $CDCl_3$)

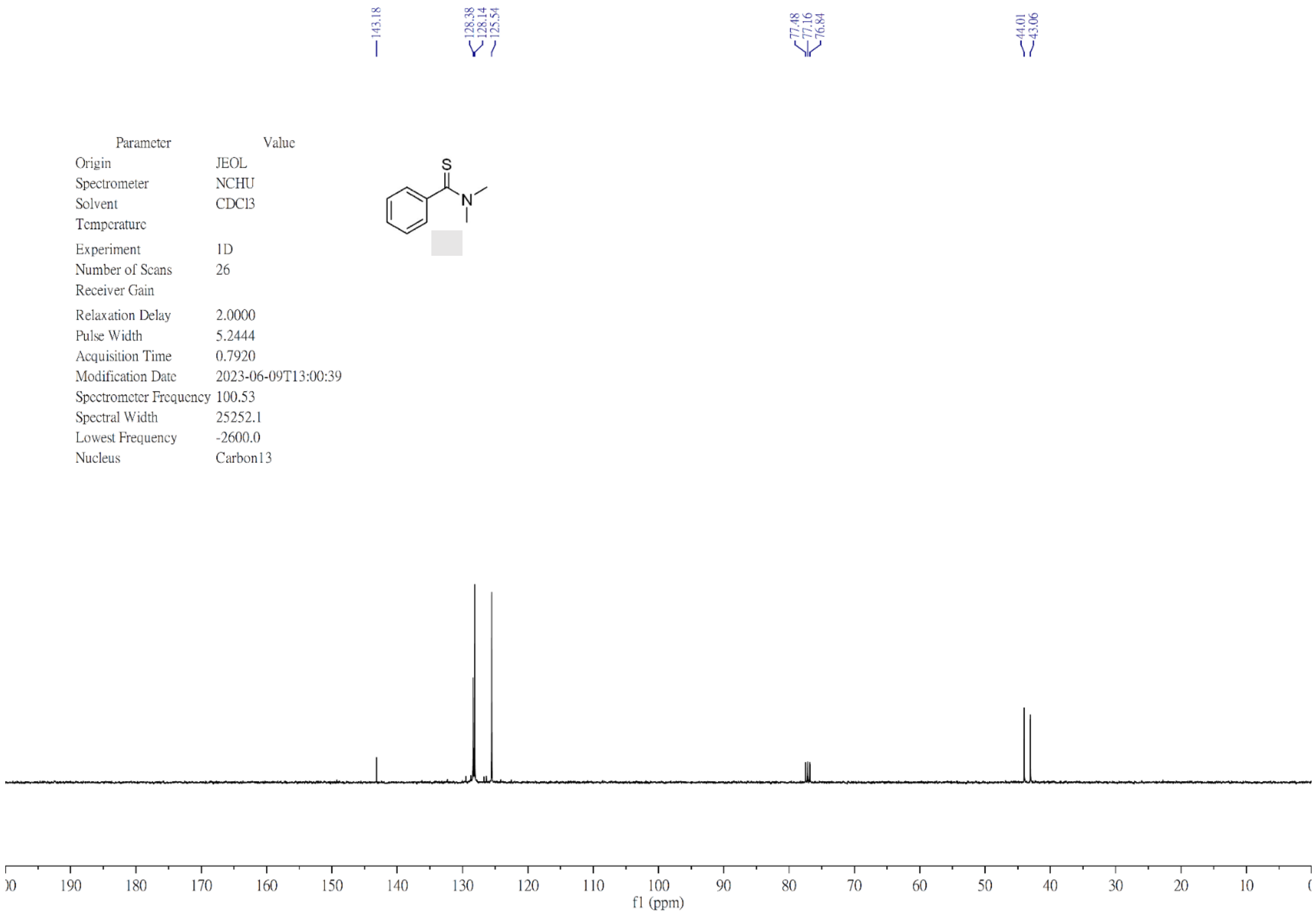

10 $^{13}C\{^{1}H\}$ NMR spectrum (100 MHz in $CDCl_3$)
